# Ontological Differences in First Compared to Third Trimester Human Fetal Placental Chorionic Stem Cells

**DOI:** 10.1371/journal.pone.0043395

**Published:** 2012-09-04

**Authors:** Gemma N. Jones, Dafni Moschidou, Tamara-Isabel Puga-Iglesias, Katarzyna Kuleszewicz, Maximilien Vanleene, Sandra J. Shefelbine, George Bou-Gharios, Nicholas M. Fisk, Anna L. David, Paolo De Coppi, Pascale V. Guillot

**Affiliations:** 1 Institute of Reproductive and Developmental Biology, Imperial College London, London, United Kingdom; 2 Department of Bioengineering, Imperial College London, London, United Kingdom; 3 Kennedy Institute of Rheumatology, University of Oxford, London, United Kingdom; 4 UQ Centre for Clinical Research, University of Queensland, Brisbane, Queensland, Australia; 5 Prenatal Cell and Gene Therapy Group, Institute for Women's Health, University College London, London, United Kingdom; 6 Surgery Unit, UCL Institute of Child Health, London, United Kingdom; Georgia Health Sciences University, United States of America

## Abstract

Human mesenchymal stromal/stem cells (MSC) isolated from fetal tissues hold promise for use in tissue engineering applications and cell-based therapies, but their collection is restricted ethically and technically. In contrast, the placenta is a potential source of readily-obtainable stem cells throughout pregnancy. In fetal tissues, early gestational stem cells are known to have advantageous characteristics over neonatal and adult stem cells. Accordingly, we investigated whether early fetal placental chorionic stem cells (e-CSC) were physiologically superior to their late gestation fetal chorionic counterparts (l-CSC). We showed that e-CSC shared a common phenotype with l-CSC, differentiating down the osteogenic, adipogenic and neurogenic pathways, and containing a subset of cells endogenously expressing *NANOG*, *SOX2*, *c-MYC*, and *KLF4*, as well as an array of genes expressed in pluripotent stem cells and primordial germ cells, including *CD24*, *NANOG*, *SSEA4*, *SSEA3*, *TRA-1-60*, *TRA-1-81*, *STELLA*, *FRAGILIS*, *NANOS3*, *DAZL* and *SSEA1*. However, we showed that e-CSC have characteristics of an earlier state of stemness compared to l-CSC, such as smaller size, faster kinetics, uniquely expressing *OCT4A* variant 1 and showing higher levels of expression of *NANOG*, *SOX2*, *c-MYC* and *KLF4* than l-CSC. Furthermore e-CSC, but not l-CSC, formed embryoid bodies containing cells from the three germ layer lineages. Finally, we showed that e-CSC demonstrate higher tissue repair *in vivo*; when transplanted in the *osteogenesis imperfecta* mice, e-CSC, but not l-CSC increased bone quality and plasticity; and when applied to a skin wound, e-CSC, but not l-CSC, accelerated healing compared to controls. Our results provide insight into the ontogeny of the stemness phenotype during fetal development and suggest that the more primitive characteristics of early compared to late gestation fetal chorionic stem cells may be translationally advantageous.

## Introduction

Mesenchymal stromal/stem cells (MSC), isolated from a range of adult and fetal tissues, have generated substantial interest for use in cell therapy and tissue engineering due to their ability to migrate to sites of injury and regenerate and repair damaged tissues [1], [2], [3]. We [4] and others [5], [6], [7] have shown that human first trimester fetal MSC present advantages for regenerative medicine over adult and perinatal MSC, such as faster kinetics, greater expansion potential, smaller size, unique adhesion molecule profile, greater telomerase activity and broader differentiation potential.

First trimester human fetal MSC have now been applied with significant effect in both hereditary and acquired disease paradigms. Human fetal blood MSC transplanted in a mouse model of osteogenesis imperfecta substantially reduced long bone fracture rates, with donor cells engrafting at sites of bone formation and differentiating into functional osteoblasts, which modified bone matrix and reduced bone brittleness [8], [9]. Similarly transplantation of fetal blood MSC in a mouse model of collagen type 1 deficiency led to improvement of glomerulopathy [10]. Finally tissue-engineered bone grafts seeded with first trimester bone marrow MSC resulted in closure of critical-sized femoral defects in rats by promoting woven and compact bone formation [11].

Collection of fetal blood and somatic tissues in the first trimester, however is technically challenging and usually requires pregnancy termination, an obstacle to autologous applications [12]. For allogeneic applications, the placenta in contrast is larger and easier to separate than other fetal tissues following pregnancy termination and hence a more realistic candidate for cell banking. In terms of supply, first trimester suction termination of pregnancy is the commonest operation in the world, but remains ethically contentious as a cell source. However fetal MSC can also be isolated in ongoing pregnancies from surplus tissues obtained during routine prenatal diagnostic procedures such as chorionic villous sampling [13], [14], [15] and amniocentesis [16], [17], [18]. Although amniotic fluid stem cells have therapeutic potential in the clinic, they are restricted to harvest in the mid-trimester, whereas the earlier gestational age of chorionic stem cells harvested at 10–12 weeks should be preferable for autologous prenatal cell therapy within the window of fetal immunological naivety. First trimester chorionic stem cells may also be developmentally-advantageous for allogenic and postnatal autologous use and have already been expanded large-scale in human serum with substantially more success than second trimester amniotic fluid stem cells [7].

Although there are substantial reports characterizing amniotic fluid stem cells, the literature on chorion-derived stem cells is insufficient with limited characterisation of immuno-phenotype and a focus primarily on isolation techniques and identification of their vascular niche within the placenta [13], [19], [20], [21], [22]. In addition many studies of term chorionic cells report cells of both fetal and maternal origin [22], [23], with some groups isolating pure maternal origin cells only [21], [24], [25], [26], and others failing to exclude maternal cell origin [19], [20], [27], [28], [29], [30]. In contrast work on first trimester chorionic stem cells has shown fetal origin by karyotyping [13], although not carried out in all studies [14], [15], [31]. Notwithstanding this problem of contaminating decidual maternal stem cells, comparisons of term placenta/chorionic stem cells with adult bone marrow MSC [22], [25] have indicated that these cells may be more primitive than adult MSC with evidence of greater self renewal [26], expression of some pluripotency markers [20], [27], [29] and potential to differentiate beyond mesodermal lineages to hepatogenic [28] and neurogenic lineages [15], [31].

We compared the phenotype of first trimester and term fetal placental chorionic stem cells (e-CSC and l-CSC respectively) to investigate their suitability for cell-based therapies and tissue engineering applications. Here, we show that compared to l-CSC, e-CSC are smaller cells with faster growth kinetics, and higher levels of pluripotency marker expression. We also found that e-CSC uniquely expressed *OCT4A variant 1* and had potential to differentiate into lineages of the three germ layers *in vitro*. In addition e-CSC and l-CSC express markers associated with primordial germ cells (PGC) and thus may share a developmental origin with these cells. Finally, we showed that e-CSC demonstrate higher tissue repair *in vivo*. Together our results suggest that e-CSC are more primitive than l-CSC and give insight into the developmental ontogeny of fetal chorionic stem cells.

## Results

### e-CSC and l-CSC are of fetal origin

**Figure 1:**
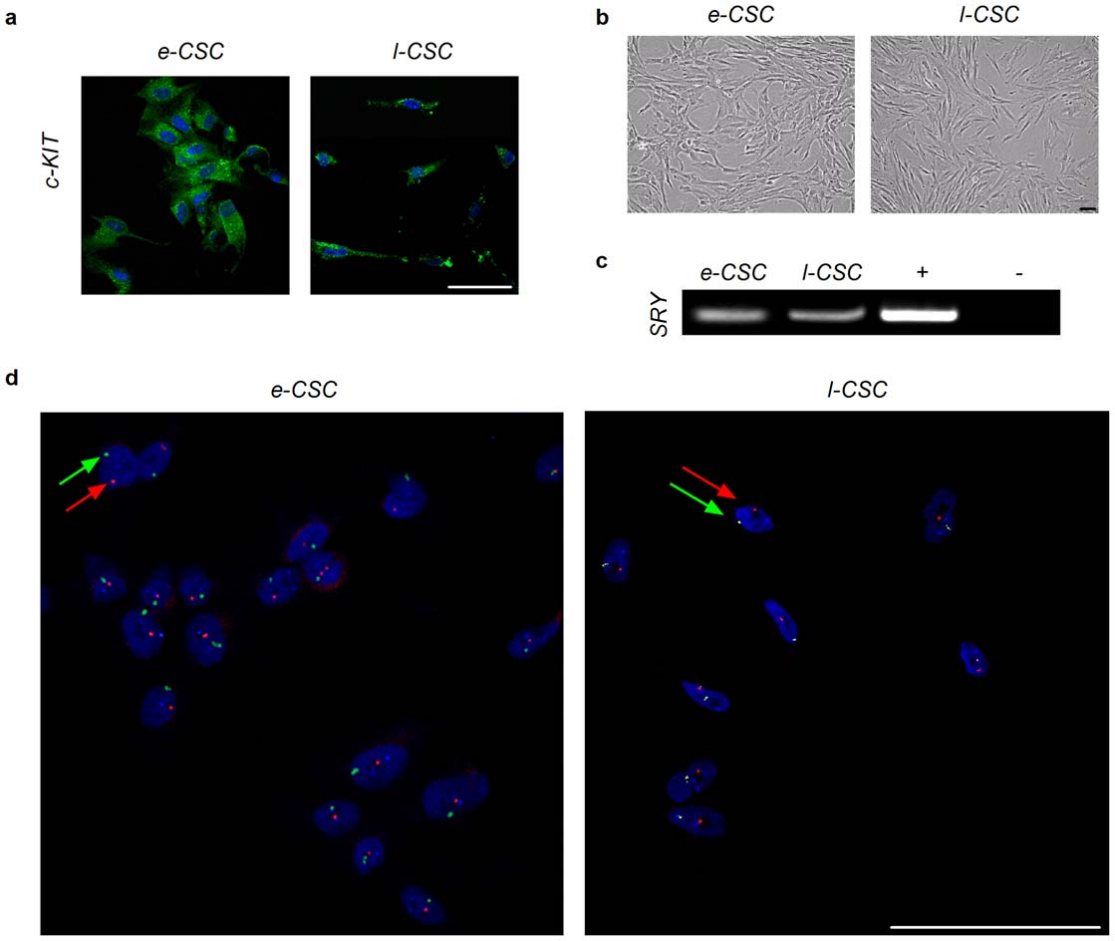
e-CSC and l-CSC are of fetal origin. (**a**) Confocal immuno-fluorescence for c-KIT (**b**) Cell morphology of early and late gestation chorionic stem cells (e-CSC and l-CSC respectively) passage 4–5. (**c**) PCR for Y chromosome specific SRY. Positive and negative controls shown. (**d**) FISH analysis for X (FITC) and Y (Texas Red) chromosomes indicated with green and red arrows respectively. 100 cells were counted. All scale bars 100 µm.

Both e-CSC and l-CSC were isolated based on plastic adherence and cultured in DMEM supplemented with 10% FBS. Both populations were c-KIT^+^ (Fig. 1a), grew in monolayers and showed a spindle shaped cytoplasm morphology, (Fig. 1b). The fetal origin of the cells was confirmed by the presence of the *SRY* gene in male samples (Fig. 1c). Fluorescence *in situ* hybridisation (FISH) analysis showed that all nuclei contained the X and Y chromosome in both e-CSC and l-CSC populations, identifying cells as being of fetal origin, with no contaminating maternal cells (Fig. 1d).

### e-CSC are smaller in size with faster growth kinetics than l-CSC

**Figure 2:**
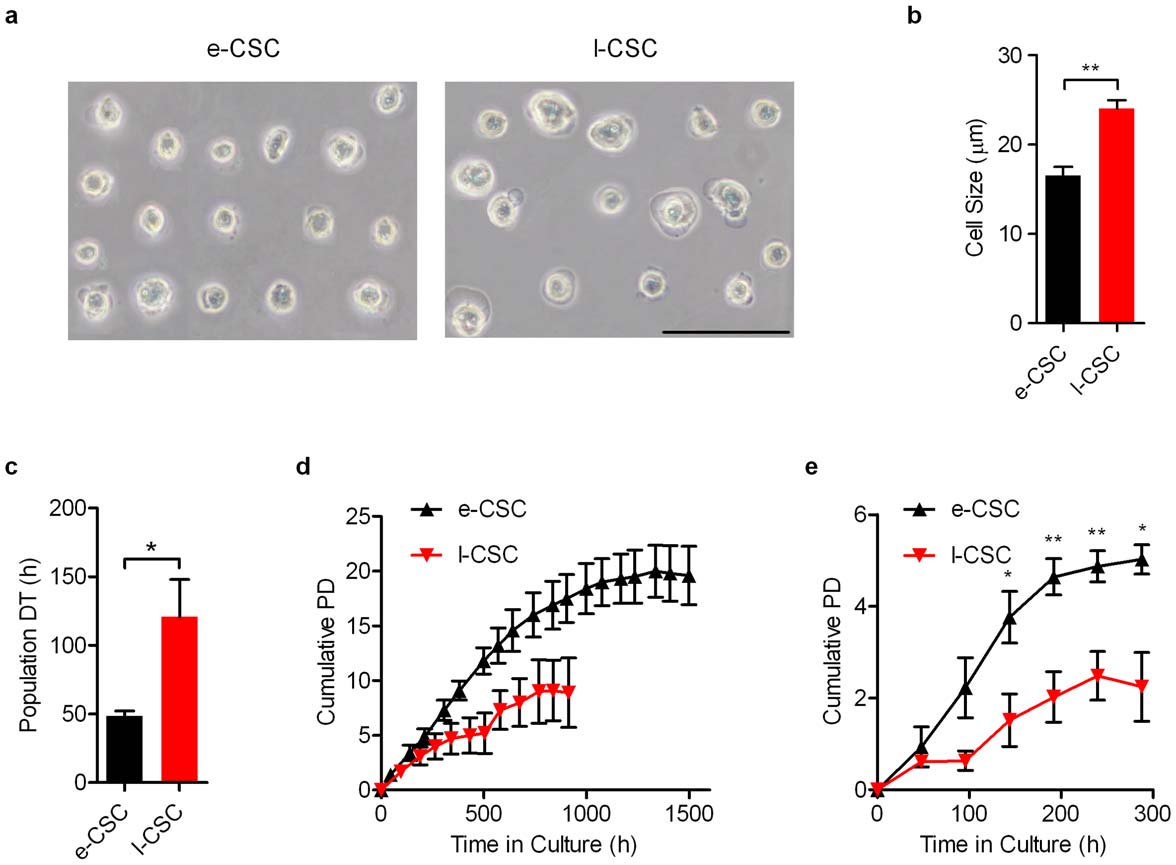
e-CSC are smaller in size and grow faster than l-CSC. (**a**) Image of cells in suspension used for cell size analysis. (**b**) Average cell size in µm of e-CSC (black) and l-CSC (red) when in suspension. (**c**) Average growth rate in hours taken for cell population to double during exponential growth phase; i.e. population doubling time (DT). e-CSC (black), l-CSC (red). (**d**) Cell expansion capacity over 1500 hours measured by average cumulative population doublings (Cumulative PD) of e-CSC (▴) and l-CSC (▾) when passaged at sub-confluence. (**e**) Cell kinetics measured by average cumulative population doublings of e-CSC (▴) and l-CSC (▾) when seeded at low density and grown beyond confluence for 288 hours. * P<0.05; ** P<0.01, Student's *t* test, *n* = 3 different samples per cell group. Mean ± s.e.m. All scale bars 100 µm.

On average e-CSC were smaller in size than l-CSC (16.5 µm±0.1 s.e.m *vs.* 24.0 µm±0.1, P<0.01; Fig. 2a and 2b). Kinetic analysis revealed that e-CSC grew faster than l-CSC (population doubling time 48.6 h±3.7 s.e.m *vs.* 120.7 h±27.2, P<0.05; Fig. 2c) over a longer period of time when maintained at sub-confluence (17 *vs.* 11 passages without reduced growth; Fig. 2d) and generated greater cell numbers when grown beyond confluence (5.0 PD±0.6 s.e.m *vs.* 2.3 PD±1.3 over 288 hours, P<0.05; Fig. 2e).

### e-CSC and l-CSC have osteogenic and adipogenic differentiation capacity

**Figure 3:**
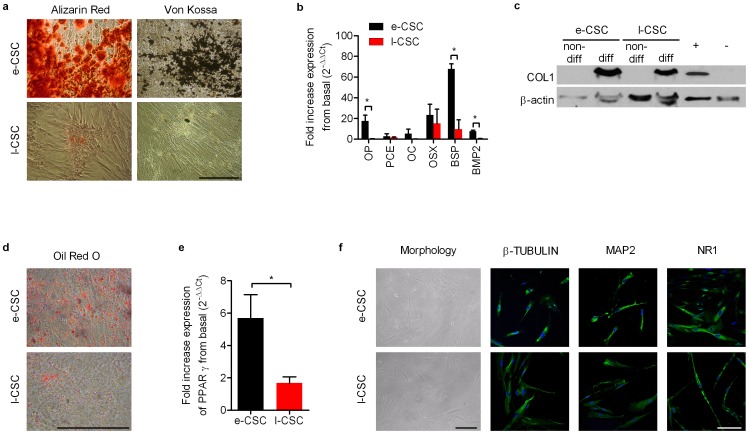
Osteogenic, adipogenic and neurogenic differentiation of e-CSC and l-CSC. (**a**) Alizarin red staining (calcium deposits) and von kossa staining (mineralisation) of cells grown in osteogenic permissive media for 2 weeks. (**b**) Quantitative real time PCR of fold increased expression of osteogenic genes after osteogenic differentiation; osteopontin (OP), procollagen endopeptidase enhancer (PCE), osteocalcin (OC), osterix (OSX), bone sialo-protein-II (BSP) and bone morphogenic protein-2 (BMP2). (**c**) Western blot of COL1 for e-CSC and l-CSC not differentiated (non-diff) and differentiated to bone for 2 weeks (diff). Positive and negative controls shown. Loading control is β-actin. (**d**) Oil Red O staining (lipid droplets) of e-CSC and l-CSC grown in adipogenic permissive media for 3 weeks. (**e**) Quantitative real time PCR of fold increased expression of adipocyte differentiation regulator peroxisome proliferator-activated receptor gamma (PPARγ). (**f**) Morphology and expression of neuronal markers β-TUBULIN, MAP2 and NMDA receptor NR1 shown with FITC (green) of e-CSC and l-CSC after 5 days neurogenic differentiation. Nuclei stained with DAPI (blue). All quantitative real time PCR data normalised to GAPDH and basal expression levels of differentiation genes (i.e. 2^−ΔΔCt^). e-CSC (black) and l-CSC (red). Data. * P<0.05; ** P<0.01, Student's *t* test, *n* = 3 per cell group. Mean ± s.e.m. All scale bars 100 µm.

The e-CSC population showed higher osteogenic potential than l-CSC, as shown qualitatively by increased intensity of alizarin red and von kossa staining after culture in osteogenic permissive conditions for 14 days (Fig. 3a). We used quantitative real time PCR (primers in Table S2) to semi-quantify the upregulation of late osteogenic genes and bone matrix related genes in e-CSC and l-CSC after 2 weeks *in vitro* differentiation. Relative expression of each gene was normalized by its expression in non-differentiating growth medium (basal level) after normalization to *GAPDH* (2^−ΔΔCt^). Results showed only differentiated e-CSC expressed osteocalcin (*OC*; 5.2%±4.6 s.e.m) and differentiated e-CSC compared to l-CSC had higher expression of osteopontin (*OP*; 17.5%±5.6 vs. 0.3%±0.3, P<0.05), bone sialo-protein-II (*BSP*; 67.8%±5.0 vs. 9.7%±11.1, P<0.05) and bone morphogenic protein-2 (*BMP2*; 7.5%±1.2 vs. 0.5%±0.3, P<0.01) (Fig. 3b). At the protein level, collagen type I (COL1) was absent in non-differentiated cells, but present in differentiated e-CSC and l-CSC samples, with e-CSC showing 3.2 times more protein (Fig. 3c).

Culture of the cells in adipogenic permissive medium for three weeks demonstrated that e-CSC produced considerably more Oil Red O-positive lipid vacuoles than l-CSC (Fig. 3d). Quantification of adipocyte differentiation regulator peroxisome proliferator-activated receptor gamma (*PPARγ*) using real time PCR analysis revealed greater expression in differentiated e-CSC than l-CSC (P<0.05) (Fig. 3e). Together, these results illustrate the higher adipogenic potential of e-CSC over l-CSC.

### e-CSC and l-CSC differentiate along the neurogenic but not the hepatogenic lineages

We next investigated the capacity of e-CSC and l-CSC to differentiate into non-mesodermal lineages. When co-cultured with C17.2 mouse neural progenitor cells in chemically defined medium supplemented with Baicalin for 5 days, e-CSC and l-CSC changed morphology and expressed the neuronal markers β-tubulin, the microtubule-associated protein 2 (MAP2) and the NMDA receptor NR1 (Fig. 3f, positive control in Fig. S2a), indicating that both cell populations have the potential to differentiate into neural-like cells.

However, culture of e-CSC and l-CSC in hepatic-permissive medium supplemented with hepatocyte growth factor over 21 days failed to induce expression of the hepatic markers albumin and alpha-fetoprotein (*AFP*) as visualized by confocal immunostaining (Fig. S1a), with the cells not producing urea in the culture medium (Fig. S1b).

### e-CSC and l-CSC share a common immunophenotype

**Figure 4:**
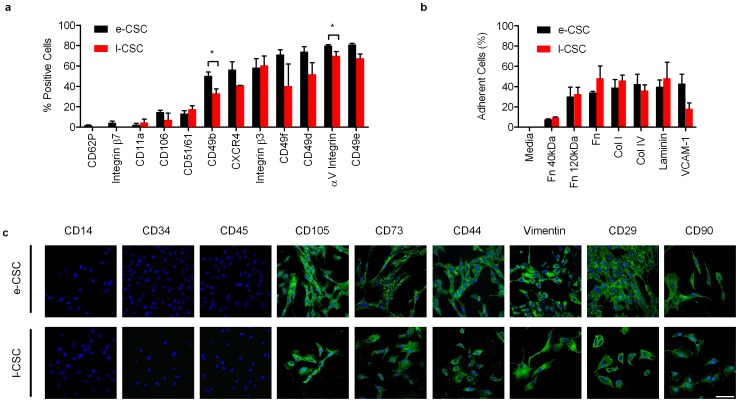
e-CSC and l-CSC have a similar immunophenotype. (**a**) Flow cytometry for percent of e-CSC (black) and l-CSC (red) populations positive for surface adhesion markers; CD62P, Integrin β7, CD11a, CD106, CD51/61, CD49b, CXCR4, Integrin β3, CD49f, CD49d, αV Integrin and CD49e. (**b**) Percentage of e-CSC (black) and l-CSC (red) populations that adhered to fibronectin a-chemotryptic 40 kDa (Fn 40 kDa), fibronectin a-chemotryptic 120 kDa (Fn 120 kDa), whole fibronectin (Fn), collagen I (col I), collagen IV (col IV), human placenta laminin (laminin) and vascular cell adhesion molecule-1 (VCAM-1). Negative control DMEM alone (media). Data. * P<0.05, Student's *t* test, *n* = 3 per cell group. Mean ± s.e.m. (**c**) Confocal immuno-fluorescence for endothelial marker (CD14), hematopoietic markers (CD34 and CD45), MSC-associated markers (CD105, CD73 and CD44), matrix protein (vimentin) and markers found in pluripotent cells as well as MSC (CD29 and CD90) stained with FITC (green). Nuclei stained with DAPI (blue). Scale bar 100 µm. Positive controls are shown in Fig. S3a.

Both e-CSC and l-CSC share a largely common adhesion molecule profile, expressing *CD11a*, *CD106*, *CD51/61*, *CXCR4*, integrin β3, *CD49f*, *CD49d* and αV integrin. However, only e-CSC expressed *CD62P* and integrin β7 and, when compared to l-CSC, e-CSC showed higher levels of *CD49b* (50.1%±3.9 s.e.m vs. 33.0%±4.5, P<0.05) and *CD49e* (81.1%±1.1 vs. 67.4%±4.2, P<0.05) (Fig. 4a). Adhesion assays showed that both e-CSC and l-CSC exhibited equal adherence to human fibronectin α-chymotryptic 40 kDa (7.5%±0.7 s.e.m vs. 9.6%±0.4) and 120 kDa fragments (30.3%±9.3 vs. 32.6%±6.6), human plasma fibronectin (34.2%±1.0 vs. 48.2%±12.2), collagen type I (38.8%±8.1 vs. 45.9%±5.4), collagen type IV (42.6%±9.7 vs. 36.1%±5.6), human placenta laminin (39.8%±6.7 vs. 48.1%±15.8) and human VCAM-1 (42.9%±9.4 vs. 18.2%±5.8) (Fig. 4b).

Both e-CSC and l-CSC share characteristics with MSC [32] with absence of endothelial (*CD14*) and hematopoietic markers (*CD34* and *CD45*), and expression of MSC-associated markers *CD105*, *CD73*, *CD44* and the matrix protein vimentin as well as *CD29* and *CD90*, although *CD29* and *CD90* are also found in human embryonic stem cells (hESC) [33], [34] (Fig. 4c). Positive controls are shown in Fig. S2b.

### e-CSC uniquely express *OCT4A* variant 1 and show higher levels of pluripotency-associated markers

**Figure 5:**
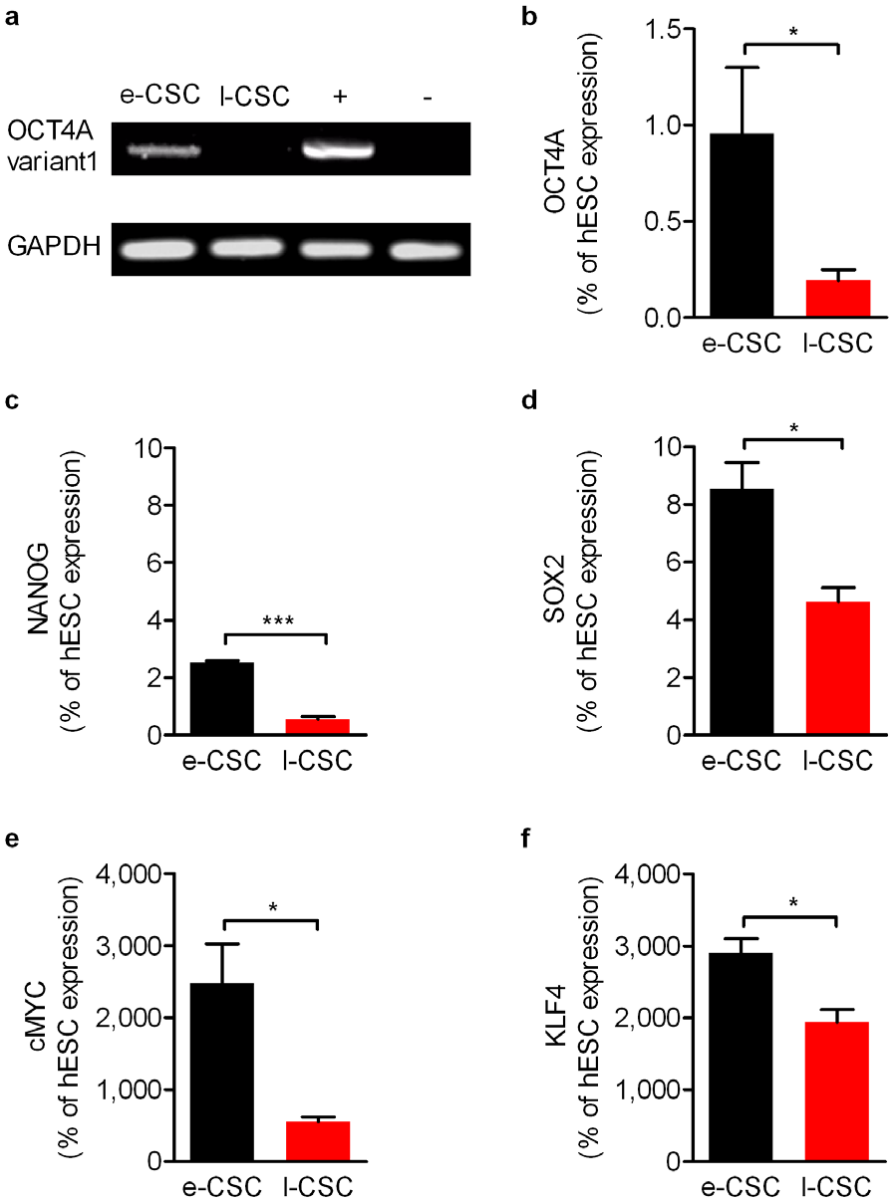
e-CSC have higher expression of pluripotency genes than l-CSC. (**a**) RT-PCR for OCT4A variant 1. GAPDH and positive and negative controls shown. (**b–f**) Quantitative real time PCR for (**b**) OCT4A all variants, (**c**) NANOG, (**d**) SOX2, (**e**) CMYC and (**f**) KLF4 for e-CSC (black) and l-CSC (red) as percentage expression of human embryonic stem cells (hESC). * P<0.05; *** P<0.001, Student's *t* test, *n* = 4 per cell group. Mean ± s.e.m.

To avoid false positive detection of *OCT4* pseudogenes not involved in maintaining pluripotency [35], [36], [37], [38], RT-PCR was performed with *OCT4A* variant 1 specific primers [35] and results showed expression in e-CSC, but not l-CSC (Fig. 5a). However, expression of the other variants of *OCT4A* was shown in both e-CSC and l-CSC when primers that also detected *OCT4A* variants 2 and 3 were used (Fig. 5b). Transcript levels of the other reprogramming factors *SOX2*, *c-MYC*, *KLF4* as well as *NANOG* were subsequently quantified and showed expression levels were considerably lower relative to hESC for *SOX2* (8.5%±1.0 s.e.m and 4.6%±0.5; Fig. 5b) and *NANOG* (2.5%±0.1 and 0.6%±0.1; Fig. 5c). However, expression levels were markedly higher in both e-CSC and l-CSC respectively relative to those found in hESC for *c-MYC* (2,472%±785.9 vs. 556.4%±106.7; Fig. 5d) and *KLF4* (2,906%±277.3 vs. 1,940%±305.8; Fig. 5e). When we compared e-CSC to l-CSC, all five pluripotency markers were expressed at greater relative levels in e-CSC; *OCT4A* (P<0.05), *SOX2* (P<0.05), *c-MYC* (P<0.05), *KLF4* (P<0.05) and *NANOG* (P<0.001).

We next used flow cytometry to determine whether lower expression in l-CSC was associated with fewer positive cells in the population than e-CSC. However, both populations contained a similar sized subset of CD24 positive cells (34.2%±3.5 s.e.m vs. 36.0%±10.0), a marker of undifferentiated hESC. In addition CD24 is not found expressed in MSC and hence we have not referred to the chorionic stem cells as MSC [33], [39]. As well as CD24, a subpopulation of cells positive for OCT4A (63.1%±5.1 s.e.m vs. 70.2%±6.6) were identified in e-CSC and l-CSC respectively (Fig. 5b).

**Figure 6:**
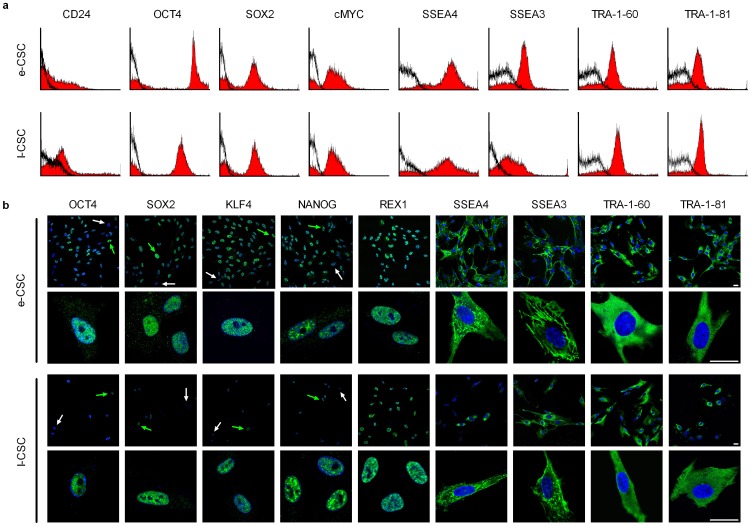
e-CSC and l-CSC contain a subpopulation positive for pluripotency markers. (**a**) Representative flow cytometry (*n* = 3) for percent of cells positive for CD24, OCT4, SOX2, CMYC, SSEA4, SSEA3, TRA-1-60 and TRA-1-81 in e-CSC and l-CSC whole populations (isotype control in black). (**b**) Representative confocal immunofluorescence images for OCT4, SOX2, KLF4, NANOG, REX1, SSEA4, SSEA3, TRA-1-60 and TRA-1-81 stained with FITC (green). Positive cells indicated with green arrow, negative cells with white arrow. Nuclei stained with DAPI (blue). Scale bar 25 µm. Positive controls are shown in Fig. S3b.

Both e-CSC and l-CSC also contained a similar sized subpopulation of cells expressing other markers typical of undifferentiated hESC [40], [41]; SOX2 (62.2%±4.2 s.e.m vs. 66.1%±3.3), c-MYC (52.3%±11.2 vs. 61.6%±11.4), SSEA4 (83.1%±8.2 vs. 69.6%±22.3), SSEA3 (63.7%±6.6 vs. 66.1%±3.3), TRA-1-60 (59.4%±10.6 vs. 62.8%±12.1), TRA-1-81 (61.8%±3.2 vs. 62.8%±12.1) (Fig. 6a). Expression and heterogeneity of the population for OCT4A, SOX2, c-MYC, SSEA4, SSEA3, TRA-1-60, and TRA-1-81 was confirmed by confocal immuno-fluorescence. Both e-CSC and l-CSC also contained a subset of cells expressing KLF4, NANOG and REX1 (Fig. 6b). Positive controls are shown in Fig. S2c.

### e-CSC uniquely form embryoid bodies

**Figure 7:**
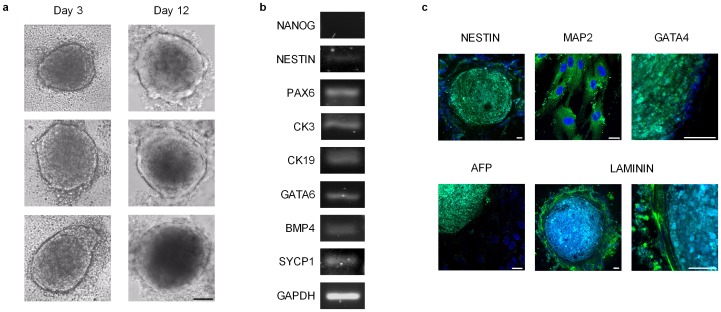
e-CSC uniquely form embryoid bodies (EBs). (**a**) Phase contrast image of e-CSC EBs at day 3 and day 12. Scale bar 100 µm. (**b**) RT-PCR for expression of NANOG, NESTIN, PAX6, CK3, CK19, GATA6, BMP4, SYCP1 and GAPDH in e-CSC EBs. (**c**) Confocal immuno-fluorescence of e-CSC EBs for expression of NESTIN, MAP2, GATA4, AFP and LAMININ stained with FITC (green). Nuclei stained with DAPI (blue). Scale bars 25 µm. Positive controls are shown in Fig. S3c.

To investigate if endogenous expression of pluripotency markers translates to an ability to differentiate beyond mesodermal lineages, we tested the capacity of e-CSC and l-CSC to form embryoid bodies (EBs) *in vitro* with differentiation to cells from the three germ layers. We showed that only e-CSC, but not l-CSC, formed EBs when grown in EB-permissive media, which may be linked to lack of *OCT4A* variant 1 expression in l-CSC. The e-CSC EBs began to form at 3 days and by 12 days were larger in size, more complex and compact in morphology and presented a defined peripheral lining (Fig. 7a). EBs from e-CSC expressed differentiation markers representative of all three germ layers: Nestin and *PAX6* (ectoderm), *CK3*, *CK19* and *GATA6* (endoderm), *BMP4* (mesoderm), and *SYCP1* (testis) with down regulation of undifferentiated stem cell marker *NANOG* (Fig.7b). This was confirmed by confocal microscopy for Nestin and MAP2 (ectoderm), GATA4 (mesoderm), AFP (endoderm) and laminin, which was expressed in the peripheral lining of the EBs (Fig. 7c). Positive controls are shown in Fig. S2d.

### e-CSC and l-CSC express markers found in primordial germ cells

**Figure 8:**
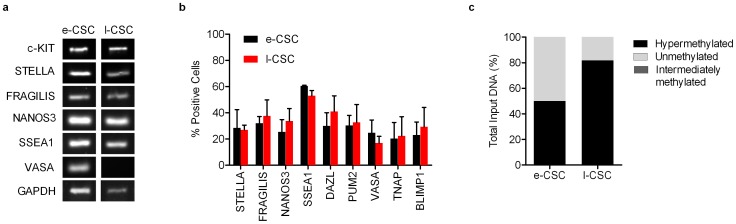
e-CSC and l-CSC express markers found in primordial germ cells. (**a**) RT-PCR for expression of c-KIT, STELLA, FRAGILIS, NANOS3, SSEA1, VASA and GAPDH. (**b**) Flow cytometry for percent of e-CSC (black) and l-CSC (red) populations positive for STELLA, FRAGILIS, NANOS3, SSEA1, DAZL, PUM2, VASA, TNAP and BLIMP1. (**c**) DNA methylation status of imprinted gene *H19* as percent total input DNA hypermethylated (black), unmethylated (light grey) and intermediately methylated (dark grey). *n* = 3 per cell group.

We next investigated the ontogeny of e-CSC and l-CSC populations. At the transcript level both e-CSC and l-CSC expressed markers found in primordial germ cells (PGC). These included *c-KIT*, a regulator of PGC survival and growth [42], [43], which is expressed in PGC, neural crest stem cells and haematopoietic stem cells during embryogenesis [44]. However, e-CSC and l-CSC also expressed other markers which commonly define PGC: Stella, which is thought to maintain pluripotentiality [45], [46], Fragilis and Nanos3, which have roles in PGC migration [47], [48] as well as SSEA1, an early marker found in PGC and human embryonic germ (hEG) cells but not hESC [49], [50] (Fig. 8a). Immuno-fluorescence and flow cytometry supported these findings (positive controls are shown in Fig. S3a). Flow cytometry showed at the protein level a subpopulation of cells in both e-CSC and l-CSC respectively positive for STELLA (29%±14 and 27%±4), FRAGILIS (32%±5 and 38%±12), NANOS3 (25%±9 and 34%±10), SSEA1 (61%±1 and 53%±4), as well as DAZL (30%±10 and 41%±12) and PUM2 (30%±8 and 33%±14), which colocalise in hEG cells [51] (Fig. 8b) Representative flow cytometry images are shown in Fig. S2b. Whereas VASA, a marker uniquely specific to the germ cell lineage [52], was only detected in e-CSC and not l-CSC at the transcript level (Fig. 8b), flow cytometry showed a small proportion of cells expressed VASA in both e-CSC and l-CSC (25%±10 and 17%±5 respectively). Also only a small proportion of e-CSC and l-CSC respectively expressed TNAP, used to denote early PGC [53], [54] (20%±12 and 22%±14) and BLIMP1, not found in hEG cells [55], but a key marker in PGC that prevents dedifferentiation into pluripotent stem cells [56] (13%±1 and 15%±1). Down-regulation of Blimp1 in e-CSC and l-CSC may be responsible for the high expression levels of its targets *c-MYC* and *KLF4*, which were at least five times higher than expressed by hESC (Fig.5). PGC reside in extra-embryonic tissues prior to migration along the genital ridge where they undergo epigenetic reprogramming including erasure of methylation on *H19* [57], [58]. To determine to which developmental stage of PGC e-CSC and l-CSC are most similar, we investigated the methylation status of *H19*. We found *H19* was both hypermethylated and unmethylated in both e-CSC (50%±0 hypermethylated and 50%±0 unmethylated, n = 3) and l-CSC (70%±8 hypermethylated and 30%±8 unmethylated, n = 3) (Fig. 8c), indicating that e-CSC and l-CSC may contain cells or primordial origin which have been retained in placental tissue prior to the start of genital ridge migration, at a time when imprint erasure has not yet started.

### e-CSC showed greater tissue repair capacity *in vivo* compared to l-CSC

**Figure 9:**
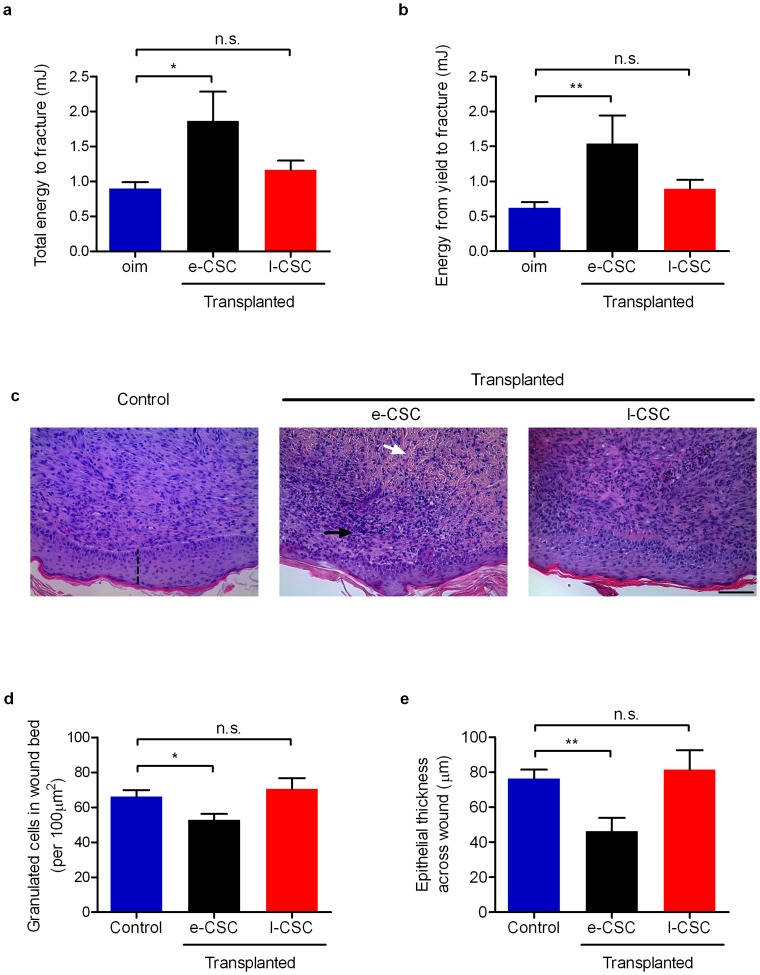
e-CSC showed better tissue repair capacity *in vivo* compared to l-CSC. (**a**) Mechanical 3-point bending data in a mouse model of bone brittleness (*oim*) for overall bone quality shown by total energy input in millijoules to fracture and for (**b**) femoral plasticity shown by energy input in millijoules from yield to fracture. Results shown per mouse for non-transplanted *oim* (blue) and *oim* transplanted with e-CSC (black) and l-CSC (red). (**c**) Cross-section of skin wounds 7 days after 4 mm dermal biopsy in mice treated with either PBS alone (Control) or transplanted with e-CSC or l-CSC; stained with haematoxylin and eosin. Indicated is collagen (white arrow), granulated cells (black arrow) and epithelial thickness (dashed line). In the same model of wound repair, (**d**) number of granulated cells in the wound bed (per 100 µm^2^) and (**e**) epithelial thickness across wound in µm. Data. * P<0.05; ** P<0.01, n.s. (not significant), Student's *t* test. Mean ± s.e.m.

We first compared the capacity of e-CSC and l-CSC to improve bone pathology in a mouse model of osteogenesis imperfecta (*oim* mice). *Oim* mice suffer from bone brittleness in response to a mutation in the collagen Type I alpha 2 gene [8], [9]; e-CSC and l-CSC (10^6^ cells in PBS) were transplanted intraperitoneally into *oim* neonates (n = 15 per transplanted group and n = 30 for non-transplanted *oim*) and culled 8 weeks later for analysis. Transplantation of e-CSC led to an improvement of overall bone quality, i.e. total work (0.90 mJ±0.09 s.e.m for non-transplanted *oim* mice vs. 1.86 mJ±0.42 for e-CSC-transplanted *oim* mice, P<0.01) (Fig. 9a) and bone plasticity, i.e. work from yield to fracture (0.62 mJ±0.08 s.e.m for non-transplanted *oim* mice vs. 1.54 mJ±0.40 for e-CSC-transplanted *oim* mice, P<0.01) (Fig. 9b), whilst *oim* mice transplanted with l-CSC did not show any significant improvements (1.16 mJ±0.14 for total work, and 0.89 mJ±0.12 for work from yield to fracture) (Fig. 9a and 9b). The differences in bone parameters between *oim* mice transplanted with e-CSC and l-CSC were likely to result from differences in intrinsic donor cell properties, as we observed no difference in donor cell engraftment between e-CSC and l-CSC for both bone marrow (BM) and bone (Fig. S4).

We next compared the tissue repair capacity of e-CSC and l-CSC during the skin wound healing process. Full thickness dermal excision skin wounds were treated with local application of either e-CSC or l-CSC in PBS or PBS alone (n = 8 per group). Morphological analysis of H&E sections of the wound beds seven days later showed that the healing process for the e-CSC-treated wounds was more advanced than for either PBS or l-CSC-treated wounds. It was evident by a lower number of infiltrated cells in the e-CSC-treated wounds compared to PBS-treated wounds (53 cells ± 3 s.e.m vs. 66 cells ± 4, respectively, calculated in a 100 µm^2^ surface, P<0.05). In contrast, l-CSC-treated wounds did not significantly differ from the controls (70 cells ± 6) (Fig. 9c and 9d). Similarly, the epithelial thickness of the wound beds was thinner for the wounds covered with e-CSC compared to controls (46 µm ± 7 s.e.m vs. 76 µm ± 5, respectively, P<0.01), whilst l-CSC-treated wounds did not significantly differ from the controls (81 µm±11) (Fig. 9c and 9e).

## Discussion

Identifying a safe, suitable and readily obtainable supply of therapeutic cells is a major challenge in regenerative medicine. Our work compares two potential candidates; first trimester and term human placental chorionic stem cells (e-CSC and l-CSC respectively), available from discarded tissues after first trimester chorionic villous sampling or termination of pregnancy, or after delivery. We derived pure populations of cells of fetal not maternal origin to show that e-CSC offer advantages relevant to the clinic when compared with their term counterparts.

Here we showed that e-CSC and l-CSC could differentiate down the osteogenic and adipogenic pathways, as well as neurogenic pathways, as previously reported [31], although they failed to differentiate down the hepatogenic lineage. Both populations shared a common immunophenotype and adhesion molecule profile, both expressing pluripotency markers as well as markers associated with PGC at the transcript and protein levels. However, e-CSC had a smaller size, higher kinetics and longer self-renewal potential, and showed greater osteogenic and adipogenic differentiation qualitatively at the whole population level. e-CSC also expressed pluripotency markers at higher levels than l-CSC, formed embryoid bodies *in vitro*, and expressed the variant 1 transcript of the *OCT4A* isoform, critical for pluripotency [35], [36], [37], [38]. In addition, relevant to cell-based therapy, we showed that e-CSC have higher tissue repair potential *in vivo* when transplanted neonatally into *osteogenesis imperfecta* mice, or applied locally to a dermal excision skin wound. This might be due to superior lineage specific differentiation and/or greater paracrine effects of e-CSC compared to l-CSC. This suggests e-CSC are more primitive than l-CSC and we therefore postulate that the phenotype of stem cells isolated from placental chorionic tissue evolves during pregnancy, with a decrease in plasticity paralleling a down regulation of expression levels of early “stemness” genes.

Our findings parallel our previous work, which showed fetal bone marrow MSC in contrast to their adult counterparts were smaller, grew faster and senesced later, despite sharing a common immunophenotype with expression of mesenchymal, adhesion and lineage markers in the whole population [4]. Others have previously reported that early placental MSC or e-CSC [13] have increased proliferative potential when compared to adult bone marrow MSC. Our data indicate that human fetal chorionic stem cells retain their immunophenotype with age, but lose associated primitive characteristics, not only from fetal to adult, but also from fetal to neonatal.

The absence of *OCT4A* variant 1 expression in l-CSC at the transcript level and downregulation of other pluripotency markers may be pivotal to the decline of primative characteristics seen in these cells when compared to their early gestational counterparts. *OCT4A* variant 1 transcript is expressed in the nuclei of undifferentiated hESC, embryonal carcinoma (EC) cells and PGC and is responsible for maintaining “stemness”, whilst the other pseudogenes are found in the cytoplasm and have no known function [36], [37], [38]. In particular the lack of *OCT4A* variant 1 in l-CSC may explain the inability of l-CSC to form embryoid bodies (EBs) as *OCT4A* variant 1 is critical to retain pluripotentiality [35], [36]. From a technical view, OCT4A was detected at the protein level in the nuclei of both e-CSC and l-CSC using OCT4A-specific antibodies; however the available antibodies may not be specific for variant 1, potentially also binding to variants 2 and 3 [35]. This was confirmed through investigation of the gene expression of all OCT4A variants, which were found expressed in both e-CSC and l-CSC. Therefore the difference between both populations is only at the level of *OCT4A* variant 1 expression.

Although *OCT4A* variant 1 was not detectable in l-CSC, other pluripotency associated markers were found in both e-CSC and l-CSC populations at both the transcript and protein level. Of particular, note both cell populations contained a similar proportion of cells expressing these markers, indicating down regulation with increased gestational age rather than a reduced number of cells expressing these early developmental markers. A similar result has been shown in amniotic epithelium stem cells, whereby expression of *NANOG* and *SOX2* was higher in first trimester amnion than term, although levels of *OCT4* were unchanged [6]. While there is little literature on this in human chorionic stem cells [59], work to date on whole chorion tissues indicates a down regulation of telomerase activity over gestation, supporting the decline of primative stem cell characteristics with age in this tissue [60].

Despite both e-CSC and l-CSC expressing early developmental markers, only e-CSC formed EBs, which we validated with typical morphology and expression of markers from the three germ layers. However, the lack of EB formation by l-CSC does not rule out the potential of these cells to differentiate beyond mesodermal lineages. Here we showed both e-CSC and l-CSC differentiated down the neurogenic lineage, but failed to differentiate down the hepatogenic lineage in our study. Others have shown stem cells isolated from both early and term placental tissue can form neural progenitors [13], [15], [31], cardiomyocytes [61], [62], [63], hepatocytes [28], [64] and pancreatic cells [65]. Results in a number of studies, mainly those on term placental stem cells, may be confounded by contamination with or overgrowth of maternal/decidual cells [23], as verified in some by characterising the cells [21], [22], [24], [25], [26] and not tested for in others [14], [15], [19], [20], [27], [28], [29], [30], [31]. Here we confirmed cells were of fetal origin with no contaminating maternal cells.

The potential of fetal derived MSC for use in bone tissue engineering is promising [3] as is their use for cell therapy in bone related disorders such as brittle bone disease [8], [66]. There is however, limited work on placenta-derived stem cells. Surbek's group showed both first trimester and term chorionic stem cells were capable of generating osteogenic grafts [67], indicating the potential of these cells in regenerative medicine. Furthermore, another study showed engraftment of term placental adherent cells inhibited growth of multiple myeloma in bone and stimulated bone formation in an immune-deficient model of medullary myeloma bone disease [68]. Notwithstanding this, neither study excluded a maternal origin for the cell populations used.

This work offers support for Kucia et al's [69], [70] hypothesis that cells isolated from adult and fetal tissues that have pluripotency characteristics may share an origin with PGC. This has been shown for murine fetal liver and adult bone marrow derived very small embryonic-like (VSEL) cells [71], and may also be true for umbilical cord blood VSEL cells [72] as well as human fetal bone marrow and lung stem cells [73], [74]. During fetal development PGC reside at the junction between embryonic and extra-embryonic tissues, then migrate through the primitive streak to give rise to extra-embryonic mesoderm [75]. Erasure of *H19* methylation occurs during PGC migration. As we have demonstrated e-CSC and l-CSC maintained both methylated and unmethylated regions on *H19* and expressed a wide array of markers associated with pre-migratory PGCs (*OCT4*, *c-KIT*, *STELLA*, *FRAGILIS*, *NANOS3*) [76], [77]. The cells may orginate from PGCs deposited in the extra-embryonic tissues before migration and hence before methylation was erased.

Notwithstanding this, we also showed that e-CSC and l-CSC contain a subfraction of cells expressing post-migratory PGC markers (*VASA*, *DAZL*) [76], [77], although *VASA* transcripts were not detected in l-CSC. Placental stem cells are known to reside in a vascular niche [19], therefore these cells may have been deposited later in PGC development following their mobilisation into the peripheral blood as shown for VSELs [78].

Human chorionic stem cells are relatively easy to isolate, can be sourced from surplus tissues at routine prenatal diagnostic procedures or after term delivery and thus used without ethical restrictions in allogeneic and autologous applications [79]. Steps towards establishing the safety of e-CSC and l-CSC for use in the clinic are already underway. No adverse clinical signs were seen after administration of allogeneic term placenta derived stem cells in NOD/SCID mice [80] or when delivered by intramuscular injection to mice with critical limb ischemia [81]. Furthermore term placenta cells were safely infused in a patient with acute myeloid leukemia [25], and are currently in Phase III clinical trials by Israel's Pluristem Therapeutics for patients with critical limb ischemia and peripheral artery disease [82], although maternal origin of these cells is possible. Transplantation of e-CSC into immunocompromised mice did not cause tumour formation and cells repopulated depleted hematopoietic tissues [14], while neural progenitors derived from e-CSC engrafted in the brain of an animal model with hypoxia-ischaemia and improved motor activity [83]. Consequently both e-CSC and l-CSC are emerging as a safe cell source for allogeneic or autologous cell therapy and tissue engineering with potential for cell or tissue banking [13], [15].

Here we have shown e-CSC have a comparable immunophenotype and developmental origin to l-CSC, but have advantages for use in regenerative medicine, which may be linked to their earlier state of stemness. Accordingly e-CSC represent a promsing cell choice for experimental and clinical applications.

## Materials and Methods

### Cell isolation and culture

Human early and term chorionic stem cells (e-CSC and l-CSC respectively) were isolated fresh after first trimester chorionic villous sampling (n = 5, 8^+3^ to 12^+0^ weeks gestational age) and term placenta from caesarean section deliveries (n = 5). The chorionic tissue (5 cm^2^ for l-CSC and the totality of the sample for e-CSC) was separated from the amnion, minced mechanically, and trypsinized using 0.05% trypsin EDTA (Invitrogen) for 20 min. Cells were subsequently centrifuged (5 min at 1500 rpm), counted and replated (10^4^ cells per cm^2^) in Dulbecco's modified Eagle's medium (DMEM-LG) (Invitrogen)+10% fetal bovine serum (BioSera). Isolated cells were plastic adherent and cKIT^+^. All cells were used at passage 4–8. Ethical approval was given by the Research Ethics Committees of Hammersmith & Queen Charlotte's Hospitals and the joint University College London Hospitals. Committees on the Ethics of Human Research (08/H0714/87).

### Bone repair mouse model

All experimental protocols complied with UK Home Office guidelines. Heterozygous male and female (B6C3Fe a/a-Col1a2*^oim^*/Col1a2*^oim^*) mice (Jackson Laboratory) were housed in individual ventilated cages (IVC) in 12∶12-hour light dark cycle (21°C) with water and chow. Offspring were genotyped by sequencing the *oim* fragment then homozygous and wild type colonies established. Homozygous *oim* mice showed multiple fractures and brittle bones [8], [9], [84]. Progeny were weaned at 30±1 day and culled at 8 weeks of age. Human e-CSC and l-CSC were injected intraperitoneally (10^6^ cells in 10 µl PBS) in homozygous *oim* neonates 3–4 days after birth. The mice were weighed and culled at 8 weeks of age for bone analysis. Three-point bending tests were performed on 8 week old unfractured frozen and thawed femurs in a materials testing machine (5866 Instron) at a loading rate of 50 µm/s on two supports 9 mm apart until fracture at mid-diaphysis. Force deflection curves were analyzed with a custom program (Matlab, MathWorks) to measure the bending stiffness (slope of the linear elastic deformation), the yield force (limit between the elastic and plastic deformation), the ultimate force (maximum force sustained), the total work to fracture (total area under the curve) and the plastic work to fracture (area under the curve from the yield point to fracture).

Donor cell engraftment: Femurs from transplanted 8 week old *oim* were dissected and bone marrow flushed. Human donor cell engraftment was determined as previously published [8], RNA was extracted using TRIzol (Invitrogen) followed by cDNA synthesis and qRT-PCR (protocol as above) then amplification of both human specific and human-mouse unspecific sequences of β-actin gene (See Table S2 for primers). Human:mouse chimerism was estimated as a ratio using 2^−ΔCt^. Samples were considered positive for engraftment with a human-specific β-actin Ct above 35 at a threshold of 0.13 ΔRn (primer specificity was confirmed by absence of amplification of non-transplanted mouse cDNA).

### Wound healing model

All experimental protocols complied with UK Home Office guidelines. C57/Bl6 mice were housed in individual ventilated cages (IVC) in 12∶12-hour light dark cycle (21°C) with water and chow. Mice were given a dermal excision as previously described by us [85]. After hair removal from the dorsal surface, 4-mm full-thickness excisional dermal wounds were made under anaesthesia. Each mouse was given two dermal excisions on each dorsal side. Stem cells (e-CSC and l-CSC, 10^6^ cells in 30 µl PBS) were applied to the left-side wounds and PBS alone applied to the right-side wounds, to avoid inter-mouse variability. Seven days later, the mice were culled and the dermal tissues excised, cut across the middle of the wound and fixed overnight in 10% neutral buffered formalin at 4°C and embedded in paraffin, sectioned and subjected to hematoxylin and eosin staining for histological analysis.

### DNA extraction and PCR

Male gender of cells was determined by DNA extraction using TRIzol (Invitrogen), followed by ethanol precipitation, washing and DNA dissolution in 8 mM NaOH. Then PCR was performed in the presence of dNTPs (Promega) and Taq polymerase (Bioline) with *SRY* specific primers (see Supplementary Table S2), followed by gel electrophoresis on 1.5% agarose with ethidium bromide (Sigma).

### FISH analysis

No contamination by maternal cells was confirmed at passages 3–9 for *SRY* positive samples using XY FISH analysis. Cells were fixed on slides as previously described [4], then digested with proteinase K before hybridization with human specific centromeric mixture probe (CEP X/Y/18) according to manufacturer's protocol (Vysis). Nuclei were counterstained with DAPI and viewed on a fluorescence confocal laser scanning microscope Leica TCS SP5 (X600 PL APO oil objective). A total of 100 cells per group were analysed.

### Cell Size

Size of passage 4–5 cells in suspension was determined using Image J software and calibrated with a haemocytometer.

### Growth Kinetics

Cells were plated in triplicate at 1×10^4^/cm^2^ and counted in trypan blue to exclude dead cells in a haemocytometer either twice weekly over 60 days with passaging to maintain sub-confluence or every 2 days for 12 days without passaging. Doubling time (DT) was calculated via the formula: DT = t/(Log_2_[y/m]), with t = time in culture, y = number of cells at end of culture, m = number of cells at beginning of culture.

### Flow cytometry

For intracellular FACS staining cells were detached, blocked with 1% bovine serum albumin (BSA; Sigma) in phosphate-buffered saline (PBS; Invitrogen) then fixed with 0.01% paraformaldehyde (PFA) (Sigma) and permeabilized with 0.5% Triton X-100 (Sigma). For cell surface and intracellular FACS staining detached cells were blocked with 1% BSA and incubated with specific anti-human primary antibodies (see Table S1), either conjugated with PE, FITC or PE-Cy5 or unconjugated. For unconjugated antibodies, cells were subsequently washed with 1% BSA and incubated with secondary goat anti-murine IgM PE (Santa Cruz). All samples were analyzed in triplicate by FACScalibur flow cytometry (Becton Dickinson). Positive controls were hESC and negative controls were IgG or IgM primary antibody-specific isotype controls.

### RNA extraction and cDNA synthesis

Total RNA was extracted from cells using RNeasy mini RNA isolation kit (Qiagen) followed by cDNA synthesis using Pd(N)6 random hexamers and M-MLV reverse transcriptase in the presence of dNTPs (Promega) (10 min, 25°C; 60 min, 42°C and 10 min, 75°C).

### RT-PCR

Reverse-transcriptase PCR (RT-PCR) was performed with cDNA using specific primers (see Table S2), dNTPs, ×10 buffer and Taq polymerase for 40 cycles according to the manufacturer's protocol (Qiagen).

### qRT-PCR

Quantitative real time polymerase chain reaction (qRT-PCR) was performed using SYBR green dye (Applied Biosystem) and analysed either with MJ-opticon or ABI Prism 7700 Sequence Detection system (Applied Biosystem). Samples were normalized against the internal control (*GAPDH*) and the 2^−ΔCt^ of each sample plotted (log_10_ scale) relative to the expression level in human embryonic stem cells (hESC) line H9 (from Wei Cui), arbitrarily set to 100. Primers supplied in Table S2.

### Fluorescence immunostaining and confocal microscopy

Cells were fixed and stained as previously described [4] and mounted in VectaShield labelled with DAPI (Vector Labs). Fluorescence confocal laser scanning microscopy images were collected on a Leica TCS SP5 (×400 PL APO oil objective) and transferred to Adobe Photoshop (Adobe Systems). Primary antibodies are listed in Table S1. Secondary antibodies were donkey anti-mouse or anti-rabbit IgG (Jackson ImmunoResearch Laboratories). Positive controls were hESC and negative controls differentiated cells.

### Adhesion Assay

Human plasma Fn (Sigma-Aldrich) or 40 and 120 kDa human Fn α-chymotryptic fragments (Chemicon ITL), calf skin collagen type I and human placenta collagen IV (Sigma-Aldrich) were adsorbed at room temperature (2 hr) at 10 µg/cm^2^ in PBS. Recombinant human VCAM-1 (ADP5, R&D Systems) and human placenta laminin (Sigma-Aldrich) were adsorbed at 1 µg/cm^2^ in PBS at 4°C similarly. Control plates were coated with 2% BSA. Coating solutions were aspirated and plates were incubated with 2% BSA in PBS (30 min) to block non-specific binding. Plates were washed with PBS before 50,000 cells/cm^2^ were plated in serum-free medium (DMEM) for 1 hr at 37°C. Non-adherent cells were removed with PBS and adherent cells were trypsinised and counted. Percentage of adhesion was calculated as follows: number of adherent cells/(number of adherent cells+number of non-adherent cells)*100 [86].

### Analysis of *H19* methylation

Genomic DNA was extracted using DNeasy Blood and Tissue Kit (Qiagen) and made free from RNA using RNase A (Invitrogen). The EpiTect Methyl qPCR assay (Qiagen) was used to digest DNA with methylation-sensitive and methylation-dependent enzymes to obtain fractions of unmethylated, partially methylated and hypermethylated DNA. The methylation status of *H19* was then quantified using qRT-PCR and specific primers (see Table S2), data were then normalised to the total amount of DNA.

### Embryoid body formation and analysis

Undifferentiated e-CSC and l-CSC were induced to differentiate *in vitro* into EBs by incubating confluent cells with differentiation medium containing knockout Dulbecco's modified eagle's medium (Gibco BRL Life Technologies) supplemented with 1 mM L-mercaptoethanol, 1% non-essential amino acids stock (all Gibco BRL Life Technologies) and 20% FBS (Biosera). At confluence, cells were incubated with 1 mg/ml type IV collagenase (Invitrogen) before being dissociated into small clumps on low attachment Petri dish in differentiating medium and allowed to develop into EBs for 12 days. EB suspensions were either pelleted and used for RNA extraction and RT-PCR of lineage specific markers (see Table S2 for primers) or transferred to gelatin-coated coverslips before fixation in 4% PFA and analysis under confocal microscopy for expression of lineage markers from all three germ layers (see Table S1 for primary antibodies used). Positive controls were pluripotent-induced human amniotic fluid stem cell-derived EBs.

### Osteogenic Differentiation

Cells were cultured in DMEM supplemented with 10 mM β-glycerophosphate, 0.2 mM ascorbic acid and 10−8 M dexamethasone for 14 days. Then cells were fixed with 10% formalin and stained with von kossa or alizarin red, or pelleted and extracted for RNA or protein for qRT-PCR or western blot analysis respectively. All analysis was compared to undifferentiated cells. qRT-PCR reactions were carried out as described above for osteogenic specific primers (given in Table S2) and normalised to *GAPDH* and compared to basal expression levels in non-differentiated cells.

### Adipogenic differentiation

Cells were seeded at a concentration of 5000 cells/cm^2^ on tissue culture plastic plates and coverslips and cultured in high glucose DMEM supplemented with 1% penicillin/streptomycin (Invitrogen), 2 mM L-glutamine (Invitrogen) and 10% FBS (Biosera Ltd., East Sussex, UK) for 1 day. The media were then changed to high glucose DMEM supplemented with 0.5 µM hydrocortisone, 0.5 µM isobutyl methylxanthine and 60 µM indomethacin (all Sigma-Aldrich) for 2 weeks. Lipid droplets were subsequently stained with oil-red-O (Sigma-Aldrich). Oil-red-O was prepared at a 1% concentration in isopropanol (BDH Laboratory Supplies, Dorset, UK) then diluted in a 3∶2 ratio of stock solution to distilled water. Cells were washed in PBS, fixed with 10% formalin, rinsed with 60% isopropanol (BDH Laboratory Supplies), followed by staining for 10 minutes. The cells were then washed 4 times with distilled water and photographed using an Olympus CK40-SLP microscope and an Olympus C3040-ADL camera (Olympus).

### Hepatogenic differentiation

Cells were seeded at a concentration of 5000 cells/cm^2^ on tissue culture plastic plates and coverslips coated with Matrigel (BD Biosciences) and cultured in high glucose DMEM supplemented with 1% penicillin/streptomycin (Invitrogen), 2 mM L-Glutamine (Invitrogen) and 10% FBS for 3 days. The media were then changed to high glucose DMEM supplemented with 15% FBS, 1% penicillin/streptomycin, 2 mM L-glutamine, 300 µM monothioglycerol (Sigma), 20 ng/ml hepatocyte growth factor (Peprotech), 10 ng/ml oncostatin M (Peprotech), 10^−7^ dexamethasone (Sigma), 100 ng/ml FGF4 (Peprotech) and 1X ITS (insulin, transferrin, selenium, Sigma). The cells were allowed to differentiate for 21 days and then fixed and stored in PBS for immunofluorescence. The differentiation media were collected and analysed for the presence of urea secreted by the differentiated cells. Urea was subsequently measured using the urea/ammonia determination kit (R-Biopharm AG, Darmstadt, Germany) according to the manufacturers instructions, with positive control being urea (as per manufacturer's instructions) and negative control undifferentiated e-CSC and l-CSC. Cells were subsquently assessed for expression of the hepatocyte markers ALBUMIN and ALPHA FETO-PROTEIN (AFP) using confocal microscopy. Positive control were HepG2 cells (gift from Wei Cui) and negative controls were undifferentiated e-CSC and l-CSC cells.

### Neurogenic differentiation

Cells were seeded at a concentration of 5000 cells/cm^2^ on tissue culture plastic plates and coverslips and cultured in high glucose DMEM supplemented with 1% penicillin/streptomycin (Invitrogen), 2 mM L-glutamine (Invitrogen) and 10% FBS (Biosera Ltd., East Sussex, UK) for 1 day. The media were then changed to high glucose DMEM supplemented with 0.5% FBS, 1% penicillin/streptomycin, 2 mM L-glutamine and 0.1% baicalin (Sigma), freshly made. C17.2 mouse neural progenitor cells (from Henrik Hagberg and Pierre Gressens) were added in a co-culture setting to promote differentiation, using co-culture membrane inserts (Thermo Scientific Nunc, Loughborough, UK). The cells were allowed to differentiate for 5 days and assessed for expression of the neuronal markers β-tubulin and MAP2 and for the NMDA receptor NR1 using confocal immunostaining. Positive controls were differentiated neural progenitors (gift Henrik Hagberg and from Pierre Gressens) and negative controls were undifferentiated e-CSC and l-CSC cells.

### Protein Extraction and Western Blot

Total protein was extracted using RIPAE buffer containing protease inhibitor cocktail and PMSF (Sigma). Protein concentrations were determined using the BCA protein assay (Thermo-scientific), with bovine serum albumin as the standard. Proteins were run on an 8% SDS-PAGE, transferred to nitrocellulose, blocked with milk and incubated with primary anitbody COL1A2 (123 kDa) (Abcam). Secondary HRP-linked anti-rabbit IgG (GE healthcare), followed by enhanced chemiluminescence (Thermo scientific) was used for detection. Loading control used was β-actin (Abcam).

### Statistical analysis

Data were expressed as mean ± s.d (standard deviation) or mean ± s.e.m (standard error). Parametric statistics were applied after confirming normal distributions on histograms and unpaired two-tailed Student's *t*-test was used for comparison between groups. P<0.05 was considered significant.

## Supporting Information

Figure S1 e-CSC and l-CSC hepatogenic differentiation. (**a**) Confocal immuno-fluorescence for ALBUMIN and AFP in e-CSC and l-CSC grown in hepatogenic permissive media for 3 weeks. HepG3 cells used for positive control. All genes stained with FITC (green). Nuclei stained with DAPI (blue). Scale bars 100 µm. (**b**) Presence of urea (mmol/l) in hepatogenic differentiation media (diff) of e-CSC and l-CSC. Positive (urea; according to the manufacturer's instructions) and negative controls; non-differentiated (non-diff) e-CSC and l-CSC are shown. *n* = 4 per cell group. Data. n.s. (not significant), Student's *t* test. Mean ± s.e.m.
(TIF)

Figure S2 Positive controls for confocal immuno-fluorescence. (**a**) Positive controls (differentiated neural progenitors) of neuronal differentiation for expression of β-TUBULIN, MAP2 and NR1. (**b**) Positive controls (human first trimester fetal bone marrow mesenchymal stem cells, hfMSC) CD105, CD73, CD44, CD29 and CD90 (**c**) Positive controls (hES cells line H9) OCT4A, SOX2, KLF4, NANOG, REX1, SSEA4, SSEA3, TRA-1-60, and TRA-1-81 (**d**) Positive controls (pluripotent-induced human amniotic fluid stem cells-derived EBs) NESTIN, MAP2, AFP, GATA4 and LAMININ. All genes stained with FITC (green). Nuclei stained with DAPI (blue). Scale bars 50 µm.
(TIF)

Figure S3 e-CSC and l-CSC express PGC associated markers. (**a**) Representative confocal immuno-fluorescence for protein expression of FRAGILIS, SSEA1, TNAP, NANOS3, BLIMP1, PUM2, STELLA, DAZL and VASA stained with FITC (green). Nuclei stained with DAPI (blue). Scale bar 100 µm. (**b**) Representative flow cytometry for PGC markers; STELLA, FRAGILIS, NANOS3, SSEA1, DAZL, PUM2, VASA, TNAP and BLIMP1 (isotype control in black).
(TIF)

Figure S4 Engraftment of e-CSC and l-CSC in 8 week-old transplanted *oim* mice. Donor cell engraftment in bone marrow and bone was calculated as the 2^−ΔCt^ of human specific β-actin normalised to human-mouse non-specific β-actin and using quantitative real time PCR in *oim* mice transplanted with e-CSC (black) or l-CSC (red).
(TIF)

Table S1 Antibodies used. List of antibodies used for immuno-fluorescence (IF), flow cytometry (FC) and immuno-histochemistry (IH).
(DOC)

Table S2 Primers used. List of primers used for RT-PCR and quantitative real time PCR and EpiTect Methyl qPCR assay.
(DOC)

## References

[1] Phinney DG, Prockop DJ (2007) Concise review: mesenchymal stem/multipotent stromal cells: the state of transdifferentiation and modes of tissue repair–current views. Stem Cells 25: 2896–2902. 17901396 10.1634/stemcells.2007-0637

[2] Chavakis E, Urbich C, Dimmeler S (2008) Homing and engraftment of progenitor cells: a prerequisite for cell therapy. J Mol Cell Cardiol 45: 514–522. 18304573 10.1016/j.yjmcc.2008.01.004

[3] Zhang ZY, Teoh SH, Hui JH, Fisk NM, Choolani M, et al (2012) The potential of human fetal mesenchymal stem cells for off-the-shelf bone tissue engineering application. Biomaterials 10.1016/j.biomaterials.2011.12.025 22217806

[4] Guillot PV, Gotherstrom C, Chan J, Kurata H, Fisk NM (2007) Human first-trimester fetal MSC express pluripotency markers and grow faster and have longer telomeres than adult MSC. Stem Cells 25: 646–654. 17124009 10.1634/stemcells.2006-0208

[5] Zhang ZY, Teoh SH, Chong MS, Schantz JT, Fisk NM, et al (2009) Superior osteogenic capacity for bone tissue engineering of fetal compared with perinatal and adult mesenchymal stem cells. Stem Cells 27: 126–137. 18832592 10.1634/stemcells.2008-0456

[6] Izumi M, Pazin BJ, Minervini CF, Gerlach J, Ross MA, et al (2009) Quantitative comparison of stem cell marker-positive cells in fetal and term human amnion. J Reprod Immunol 81: 39–43. 19501410 10.1016/j.jri.2009.02.007

[7] Poloni A, Maurizi G, Serrani F, Mancini S, Discepoli G, et al (2012) Human AB serum for generation of mesenchymal stem cells from human chorionic villi: comparison with other source and other media including platelet lysate. Cell Prolif 45: 66–75. 22168227 10.1111/j.1365-2184.2011.00799.x PMC6496523

[8] Guillot PV, Abass O, Bassett JH, Shefelbine SJ, Bou-Gharios G, et al (2008) Intrauterine transplantation of human fetal mesenchymal stem cells from first-trimester blood repairs bone and reduces fractures in osteogenesis imperfecta mice. Blood 111: 1717–1725. 17967940 10.1182/blood-2007-08-105809

[9] Vanleene M, Saldanha Z, Cloyd KL, Jell G, Bou-Gharios G, et al (2011) Transplantation of human fetal blood stem cells in the osteogenesis imperfecta mouse leads to improvement in multiscale tissue properties. Blood 117: 1053–1060. 21088133 10.1182/blood-2010-05-287565

[10] Guillot PV, Cook HT, Pusey CD, Fisk NM, Harten S, et al (2008) Transplantation of human fetal mesenchymal stem cells improves glomerulopathy in a collagen type I alpha 2-deficient mouse. J Pathol 214: 627–636. 18266309 10.1002/path.2325

[11] Zhang ZY, Teoh SH, Chong MS, Lee ES, Tan LG, et al (2010) Neo-vascularization and bone formation mediated by fetal mesenchymal stem cell tissue-engineered bone grafts in critical-size femoral defects. Biomaterials 31: 608–620. 19836073 10.1016/j.biomaterials.2009.09.078

[12] Chan J, Kumar S, Fisk NM (2008) First trimester embryo-fetoscopic and ultrasound-guided fetal blood sampling for ex vivo viral transduction of cultured human fetal mesenchymal stem cells. Hum Reprod 23: 2427–2437. 18687673 10.1093/humrep/den302

[13] Poloni A, Rosini V, Mondini E, Maurizi G, Mancini S, et al (2008) Characterization and expansion of mesenchymal progenitor cells from first-trimester chorionic villi of human placenta. Cytotherapy 10: 690–697. 18985476 10.1080/14653240802419310

[14] Spitalieri P, Cortese G, Pietropolli A, Filareto A, Dolci S, et al (2009) Identification of multipotent cytotrophoblast cells from human first trimester chorionic villi. Cloning Stem Cells 11: 535–556. 20025524 10.1089/clo.2009.0046

[15] Portmann-Lanz CB, Schoeberlein A, Huber A, Sager R, Malek A, et al (2006) Placental mesenchymal stem cells as potential autologous graft for pre- and perinatal neuroregeneration. Am J Obstet Gynecol 194: 664–673. 16522395 10.1016/j.ajog.2006.01.101

[16] De Coppi P, Bartsch G Jr, Siddiqui MM, Xu T, Santos CC, et al (2007) Isolation of amniotic stem cell lines with potential for therapy. Nat Biotechnol 25: 100–106. 17206138 10.1038/nbt1274

[17] Tsai MS, Lee JL, Chang YJ, Hwang SM (2004) Isolation of human multipotent mesenchymal stem cells from second-trimester amniotic fluid using a novel two-stage culture protocol. Hum Reprod 19: 1450–1456. 15105397 10.1093/humrep/deh279

[18] Prusa AR, Marton E, Rosner M, Bernaschek G, Hengstschlager M (2003) Oct-4-expressing cells in human amniotic fluid: a new source for stem cell research? Hum Reprod 18: 1489–1493. 12832377 10.1093/humrep/deg279

[19] Castrechini NM, Murthi P, Gude NM, Erwich JJ, Gronthos S, et al (2010) Mesenchymal stem cells in human placental chorionic villi reside in a vascular Niche. Placenta 31: 203–212. 20060164 10.1016/j.placenta.2009.12.006

[20] Bacenkova D, Rosocha J, Tothova T, Rosocha L, Sarissky M (2011) Isolation and basic characterization of human term amnion and chorion mesenchymal stromal cells. Cytotherapy 13: 1047–1056. 21916779 10.3109/14653249.2011.592522

[21] Rus Ciuca D, Soritau O, Susman S, Pop VI, Mihu CM (2011) Isolation and characterization of chorionic mesenchyal stem cells from the placenta. Rom J Morphol Embryol 52: 803–808. 21892522

[22] Soncini M, Vertua E, Gibelli L, Zorzi F, Denegri M, et al (2007) Isolation and characterization of mesenchymal cells from human fetal membranes. J Tissue Eng Regen Med 1: 296–305. 18038420 10.1002/term.40

[23] In't Anker PS, Scherjon SA, Kleijburg-van der Keur C, de Groot-Swings GM, Claas FH, et al (2004) Isolation of mesenchymal stem cells of fetal or maternal origin from human placenta. Stem Cells 22: 1338–1345. 15579651 10.1634/stemcells.2004-0058

[24] Semenov OV, Koestenbauer S, Riegel M, Zech N, Zimmermann R, et al (2010) Multipotent mesenchymal stem cells from human placenta: critical parameters for isolation and maintenance of stemness after isolation. Am J Obstet Gynecol 202: 193 e191–193 e113. 20035913 10.1016/j.ajog.2009.10.869

[25] Brooke G, Rossetti T, Pelekanos R, Ilic N, Murray P, et al (2009) Manufacturing of human placenta-derived mesenchymal stem cells for clinical trials. Br J Haematol 144: 571–579. 19077161 10.1111/j.1365-2141.2008.07492.x

[26] Barlow S, Brooke G, Chatterjee K, Price G, Pelekanos R, et al (2008) Comparison of human placenta- and bone marrow-derived multipotent mesenchymal stem cells. Stem Cells Dev 17: 1095–1107. 19006451 10.1089/scd.2007.0154

[27] Yen BL, Huang HI, Chien CC, Jui HY, Ko BS, et al (2005) Isolation of multipotent cells from human term placenta. Stem Cells 23: 3–9. 15625118 10.1634/stemcells.2004-0098

[28] Kim MJ, Shin KS, Jeon JH, Lee DR, Shim SH, et al (2011) Human chorionic-plate-derived mesenchymal stem cells and Wharton's jelly-derived mesenchymal stem cells: a comparative analysis of their potential as placenta-derived stem cells. Cell Tissue Res 346: 53–64. 21987220 10.1007/s00441-011-1249-8

[29] Fariha MM, Chua KH, Tan GC, Tan AE, Hayati AR (2011) Human chorion-derived stem cells: changes in stem cell properties during serial passage. Cytotherapy 13: 582–593. 21231803 10.3109/14653249.2010.549121

[30] Miao Z, Jin J, Chen L, Zhu J, Huang W, et al (2006) Isolation of mesenchymal stem cells from human placenta: comparison with human bone marrow mesenchymal stem cells. Cell Biol Int 30: 681–687. 16870478 10.1016/j.cellbi.2006.03.009

[31] Portmann-Lanz CB, Schoeberlein A, Portmann R, Mohr S, Rollini P, et al (2010) Turning placenta into brain: placental mesenchymal stem cells differentiate into neurons and oligodendrocytes. Am J Obstet Gynecol 202: 294 e291–294 e211. 20060088 10.1016/j.ajog.2009.10.893

[32] Dominici M, Le Blanc K, Mueller I, Slaper-Cortenbach I, Marini F, et al (2006) Minimal criteria for defining multipotent mesenchymal stromal cells. The International Society for Cellular Therapy position statement. Cytotherapy 8: 315–317. 16923606 10.1080/14653240600855905

[33] Lian Q, Lye E, Suan Yeo K, Khia Way Tan E, Salto-Tellez M, et al (2007) Derivation of clinically compliant MSCs from CD105+, CD24− differentiated human ESCs. Stem Cells 25: 425–436. 17053208 10.1634/stemcells.2006-0420

[34] Carpenter MK, Rosler ES, Fisk GJ, Brandenberger R, Ares X, et al (2004) Properties of four human embryonic stem cell lines maintained in a feeder-free culture system. Dev Dyn 229: 243–258. 14745950 10.1002/dvdy.10431

[35] Ryan JM, Pettit AR, Guillot PV, Chan JK, Fisk NM (2011) Unravelling the Pluripotency Paradox in Fetal and Placental Mesenchymal Stem Cells: Oct-4 Expression and the Case of the Emperor's New Clothes. Stem Cell Rev 10.1007/s12015-011-9336-5 22161644

[36] Atlasi Y, Mowla SJ, Ziaee SA, Gokhale PJ, Andrews PW (2008) OCT4 spliced variants are differentially expressed in human pluripotent and nonpluripotent cells. Stem Cells 26: 3068–3074. 18787205 10.1634/stemcells.2008-0530

[37] Lee J, Kim HK, Rho JY, Han YM, Kim J (2006) The human OCT-4 isoforms differ in their ability to confer self-renewal. J Biol Chem 281: 33554–33565. 16951404 10.1074/jbc.M603937200

[38] Pesce M, Scholer HR (2000) Oct-4: control of totipotency and germline determination. Mol Reprod Dev 55: 452–457. 10694754 10.1002/(SICI)1098-2795(200004)55:4<452::AID-MRD14>3.0.CO;2-S

[39] Assou S, Le Carrour T, Tondeur S, Strom S, Gabelle A, et al (2007) A meta-analysis of human embryonic stem cells transcriptome integrated into a web-based expression atlas. Stem Cells 25: 961–973. 17204602 10.1634/stemcells.2006-0352 PMC1906587

[40] Adewumi O, Aflatoonian B, Ahrlund-Richter L, Amit M, Andrews PW, et al (2007) Characterization of human embryonic stem cell lines by the International Stem Cell Initiative. Nat Biotechnol 25: 803–816. 17572666 10.1038/nbt1318

[41] Skottman H, Mikkola M, Lundin K, Olsson C, Stromberg AM, et al (2005) Gene expression signatures of seven individual human embryonic stem cell lines. Stem Cells 23: 1343–1356. 16081666 10.1634/stemcells.2004-0341

[42] De Miguel MP, Cheng L, Holland EC, Federspiel MJ, Donovan PJ (2002) Dissection of the c-Kit signaling pathway in mouse primordial germ cells by retroviral-mediated gene transfer. Proc Natl Acad Sci U S A 99: 10458–10463. 12140361 10.1073/pnas.122249399 PMC124938

[43] Hoyer PE, Byskov AG, Mollgard K (2005) Stem cell factor and c-Kit in human primordial germ cells and fetal ovaries. Mol Cell Endocrinol 234: 1–10. 15836947 10.1016/j.mce.2004.09.012

[44] Motohashi T, Aoki H, Chiba K, Yoshimura N, Kunisada T (2007) Multipotent cell fate of neural crest-like cells derived from embryonic stem cells. Stem Cells 25: 402–410. 17038669 10.1634/stemcells.2006-0323

[45] Bowles J, Teasdale RP, James K, Koopman P (2003) Dppa3 is a marker of pluripotency and has a human homologue that is expressed in germ cell tumours. Cytogenet Genome Res 101: 261–265. 14684992 10.1159/000074346

[46] Payer B, Saitou M, Barton SC, Thresher R, Dixon JP, et al (2003) Stella is a maternal effect gene required for normal early development in mice. Curr Biol 13: 2110–2117. 14654002 10.1016/j.cub.2003.11.026

[47] Tanaka SS, Yamaguchi YL, Tsoi B, Lickert H, Tam PP (2005) IFITM/Mil/fragilis family proteins IFITM1 and IFITM3 play distinct roles in mouse primordial germ cell homing and repulsion. Dev Cell 9: 745–756. 16326387 10.1016/j.devcel.2005.10.010

[48] Tsuda M, Sasaoka Y, Kiso M, Abe K, Haraguchi S, et al (2003) Conserved role of nanos proteins in germ cell development. Science 301: 1239–1241. 12947200 10.1126/science.1085222

[49] Henderson JK, Draper JS, Baillie HS, Fishel S, Thomson JA, et al (2002) Preimplantation human embryos and embryonic stem cells show comparable expression of stage-specific embryonic antigens. Stem Cells 20: 329–337. 12110702 10.1634/stemcells.20-4-329

[50] Park JH, Kim SJ, Lee JB, Song JM, Kim CG, et al (2004) Establishment of a human embryonic germ cell line and comparison with mouse and human embryonic stem cells. Mol Cells 17: 309–315. 15179047

[51] Moore FL, Jaruzelska J, Fox MS, Urano J, Firpo MT, et al (2003) Human Pumilio-2 is expressed in embryonic stem cells and germ cells and interacts with DAZ (Deleted in AZoospermia) and DAZ-like proteins. Proc Natl Acad Sci U S A 100: 538–543. 12511597 10.1073/pnas.0234478100 PMC141031

[52] Castrillon DH, Quade BJ, Wang TY, Quigley C, Crum CP (2000) The human VASA gene is specifically expressed in the germ cell lineage. Proc Natl Acad Sci U S A 97: 9585–9590. 10920202 10.1073/pnas.160274797 PMC16908

[53] MacGregor GR, Zambrowicz BP, Soriano P (1995) Tissue non-specific alkaline phosphatase is expressed in both embryonic and extraembryonic lineages during mouse embryogenesis but is not required for migration of primordial germ cells. Development 121: 1487–1496. 7789278 10.1242/dev.121.5.1487

[54] McLaren A (2003) Primordial germ cells in the mouse. Dev Biol 262: 1–15. 14512014 10.1016/s0012-1606(03)00214-8

[55] Durcova-Hills G, Tang F, Doody G, Tooze R, Surani MA (2008) Reprogramming primordial germ cells into pluripotent stem cells. PLoS One 3: e3531. 18953407 10.1371/journal.pone.0003531 PMC2567847

[56] Ohinata Y, Payer B, O'Carroll D, Ancelin K, Ono Y, et al (2005) Blimp1 is a critical determinant of the germ cell lineage in mice. Nature 436: 207–213. 15937476 10.1038/nature03813

[57] Arnaud P (2010) Genomic imprinting in germ cells: imprints are under control. Reproduction 140: 411–423. 20501788 10.1530/REP-10-0173

[58] Hajkova P, Erhardt S, Lane N, Haaf T, El-Maarri O, et al (2002) Epigenetic reprogramming in mouse primordial germ cells. Mech Dev 117: 15–23. 12204247 10.1016/s0925-4773(02)00181-8

[59] Sung HJ, Hong SC, Yoo JH, Oh JH, Shin HJ, et al (2010) Stemness evaluation of mesenchymal stem cells from placentas according to developmental stage: comparison to those from adult bone marrow. J Korean Med Sci 25: 1418–1426. 20890420 10.3346/jkms.2010.25.10.1418 PMC2946649

[60] Kyo S, Takakura M, Tanaka M, Kanaya T, Sagawa T, et al (1997) Expression of telomerase activity in human chorion. Biochem Biophys Res Commun 241: 498–503. 9425299 10.1006/bbrc.1997.7767

[61] Schmidt D, Mol A, Breymann C, Achermann J, Odermatt B, et al (2006) Living autologous heart valves engineered from human prenatally harvested progenitors. Circulation 114: I125–131. 16820561 10.1161/CIRCULATIONAHA.105.001040

[62] Weber B, Zeisberger SM, Hoerstrup SP (2011) Prenatally harvested cells for cardiovascular tissue engineering: fabrication of autologous implants prior to birth. Placenta 32 Suppl 4: S316–319. 21575988 10.1016/j.placenta.2011.04.001

[63] Okamoto K, Miyoshi S, Toyoda M, Hida N, Ikegami Y, et al (2007) ‘Working’ cardiomyocytes exhibiting plateau action potentials from human placenta-derived extraembryonic mesodermal cells. Exp Cell Res 313: 2550–2562. 17544394 10.1016/j.yexcr.2007.04.028

[64] Shin KS, Lee HJ, Jung J, Cha DH, Kim GJ (2010) Culture and in vitro hepatogenic differentiation of placenta-derived stem cells, using placental extract as an alternative to serum. Cell Prolif 43: 435–444. 20887550 10.1111/j.1365-2184.2010.00693.x PMC6496597

[65] Susman S, Soritau O, Rus-Ciuca D, Tomuleasa C, Pop VI, et al (2010) Placental stem cell differentiation into islets of Langerhans-like glucagon-secreting cells. Rom J Morphol Embryol 51: 733–738. 21103634

[66] Le Blanc K, Gotherstrom C, Ringden O, Hassan M, McMahon R, et al (2005) Fetal mesenchymal stem-cell engraftment in bone after in utero transplantation in a patient with severe osteogenesis imperfecta. Transplantation 79: 1607–1614. 15940052 10.1097/01.tp.0000159029.48678.93

[67] Mohr S, Portmann-Lanz CB, Schoeberlein A, Sager R, Surbek DV (2010) Generation of an osteogenic graft from human placenta and placenta-derived mesenchymal stem cells. Reprod Sci 17: 1006–1015. 20940246 10.1177/1933719110377471

[68] Li X, Ling W, Pennisi A, Wang Y, Khan S, et al (2011) Human placenta-derived adherent cells prevent bone loss, stimulate bone formation, and suppress growth of multiple myeloma in bone. Stem Cells 29: 263–273. 21732484 10.1002/stem.572 PMC3175303

[69] Kucia M, Machalinski B, Ratajczak MZ (2006) The developmental deposition of epiblast/germ cell-line derived cells in various organs as a hypothetical explanation of stem cell plasticity? Acta Neurobiol Exp (Wars) 66: 331–341. 17265694 10.55782/ane-2006-1622

[70] Kucia M, Wu W, Ratajczak MZ (2007) Bone marrow-derived very small embryonic-like stem cells: their developmental origin and biological significance. Dev Dyn 236: 3309–3320. 17497671 10.1002/dvdy.21180

[71] Shin DM, Liu R, Klich I, Wu W, Ratajczak J, et al (2010) Molecular signature of adult bone marrow-purified very small embryonic-like stem cells supports their developmental epiblast/germ line origin. Leukemia 24: 1450–1461. 20508611 10.1038/leu.2010.121

[72] Kucia M, Halasa M, Wysoczynski M, Baskiewicz-Masiuk M, Moldenhawer S, et al (2007) Morphological and molecular characterization of novel population of CXCR4+ SSEA-4+ Oct-4+ very small embryonic-like cells purified from human cord blood: preliminary report. Leukemia 21: 297–303. 17136117 10.1038/sj.leu.2404470

[73] Hua J, Yu H, Dong W, Yang C, Gao Z, et al (2009) Characterization of mesenchymal stem cells (MSCs) from human fetal lung: potential differentiation of germ cells. Tissue Cell 41: 448–455. 19651422 10.1016/j.tice.2009.05.004

[74] Hua J, Pan S, Yang C, Dong W, Dou Z, et al (2009) Derivation of male germ cell-like lineage from human fetal bone marrow stem cells. Reprod Biomed Online 19: 99–105. 19573297 10.1016/s1472-6483(10)60052-1

[75] Donovan PJ (1998) The germ cell–the mother of all stem cells. Int J Dev Biol 42: 1043–1050. 9853836

[76] Saiti D, Lacham-Kaplan O (2007) Mouse Germ Cell Development in-vivo and in-vitro. Biomark Insights 2: 241–252. 19662207 PMC2717835

[77] Hayashi K, de Sousa Lopes SM, Surani MA (2007) Germ cell specification in mice. Science 316: 394–396. 17446386 10.1126/science.1137545

[78] Ratajczak MZ, Liu R, Marlicz W, Blogowski W, Starzynska T, et al (2011) Identification of very small embryonic/epiblast-like stem cells (VSELs) circulating in peripheral blood during organ/tissue injuries. Methods Cell Biol 103: 31–54. 21722799 10.1016/B978-0-12-385493-3.00003-6

[79] Abdulrazzak H, Moschidou D, Jones G, Guillot PV (2010) Biological characteristics of stem cells from foetal, cord blood and extraembryonic tissues. J R Soc Interface 7 Suppl 6: S689–706. 20739312 10.1098/rsif.2010.0347.focus PMC2988276

[80] Ramot Y, Meiron M, Toren A, Steiner M, Nyska A (2009) Safety and biodistribution profile of placental-derived mesenchymal stromal cells (PLX-PAD) following intramuscular delivery. Toxicol Pathol 37: 606–616. 19478280 10.1177/0192623309338383

[81] Prather WR, Toren A, Meiron M, Ofir R, Tschope C, et al (2009) The role of placental-derived adherent stromal cell (PLX-PAD) in the treatment of critical limb ischemia. Cytotherapy 11: 427–434. 19526389 10.1080/14653240902849762

[82] Trounson A, Thakar RG, Lomax G, Gibbons D (2011) Clinical trials for stem cell therapies. BMC Med 9: 52. 21569277 10.1186/1741-7015-9-52 PMC3098796

[83] Park S, Koh SE, Maeng S, Lee WD, Lim J, et al (2011) Neural progenitors generated from the mesenchymal stem cells of first-trimester human placenta matured in the hypoxic-ischemic rat brain and mediated restoration of locomotor activity. Placenta 32: 269–276. 21300404 10.1016/j.placenta.2010.12.027

[84] Jones GN, Moschidou D, Lay K, Abdulrazzak H, Vanleene M, et al (2012) Upregulating CXCR4 in human fetal mesenchymal stem cells enhances engraftment and bone mechanics in a mouse model of osteogenesis imperfecta. Stem Cells Translational Medicine 1: 70–78. 23197643 10.5966/sctm.2011-0007 PMC3727689

[85] Rajkumar VS, Shiwen X, Bostrom M, Leoni P, Muddle J, et al (2006) Platelet-derived growth factor-beta receptor activation is essential for fibroblast and pericyte recruitment during cutaneous wound healing. Am J Pathol 169: 2254–2265. 17148686 10.2353/ajpath.2006.060196 PMC1762470

[86] Huygen S, Giet O, Artisien V, Di Stefano I, Beguin Y, et al (2002) Adhesion of synchronized human hematopoietic progenitor cells to fibronectin and vascular cell adhesion molecule-1 fluctuates reversibly during cell cycle transit in ex vivo culture. Blood 100: 2744–2752. 12351381 10.1182/blood.V100.8.2744

