# Expression of Concern: Ontological Differences in First Compared to Third Trimester Human Fetal Placental Chorionic Stem Cells

**DOI:** 10.1371/journal.pone.0329484

**Published:** 2025-08-01

**Authors:** The PLOS One Editors

Following the publication of this article [1], concerns were raised regarding results presented in Figs 1, 2, and 6. Specifically,

**Fig 2:**
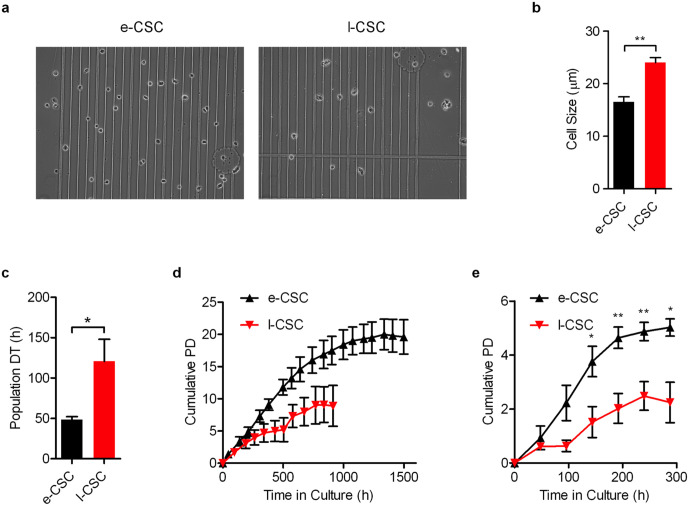
e-CSC are smaller in size and grow faster than l-CSC. (a) Image of cells in suspension used for cell size analysis. (b) Average cell size in µm of e-CSC (black) and l-CSC (red) when in suspension. (c) Average growth rate in hours taken for cell population to double during exponential growth phase; i.e., population doubling time (DT). e-CSC (black), l-CSC (red). (d) Cell expansion capacity over 1500 hours measured by average cumulative population doublings (Cumulative PD) of e-CSC (▴) and l-CSC (▾) when passaged at sub-confluence. (e) Cell kinetics measured by average cumulative population doublings of e-CSC (▴) and l-CSC (▾) when seeded at low density and grown beyond confluence for 288 hours. * P < 0.05; ** P < 0.01, Student’s *t* test, *n* = 3 different samples per cell group. Mean ± s.e.m. All scale bars 100 µm.

**Fig 6:**
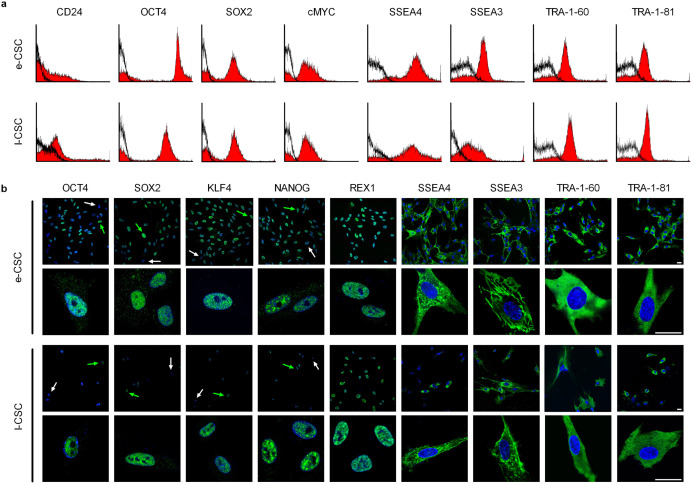
e-CSC and l-CSC contain a subpopulation positive for pluripotency markers. (a) Representative flow cytometry (*n* = 3) for percent of cells positive for CD24, OCT4, SOX2, CMYC, SSEA4, SSEA3, TRA-1–60 and TRA-1–81 in e-CSC and l-CSC whole populations (isotype control in black). (b) Representative confocal immunofluorescence images for OCT4, SOX2, KLF4, NANOG, REX1, SSEA4, SSEA3, TRA-1–60 and TRA-1–81 stained with FITC (green). Positive cells indicated with green arrow, negative cells with white arrow. Nuclei stained with DAPI (blue). Scale bar 25 µm. Positive controls are shown in Fig. S3b.

- When adjusting the color levels of the Fig 1a I-CSC panel, the background noise of the top of the panel does not appear to match the background noise of the bottom of the panel.
- Repetitive elements are observed within the Fig 2a e-CSC and the Fig 2a I-CSC panels.
- The top Fig 6b I-CSC TRA-1–60 panel of this article [1] appears similar to the Fig 2c AFSC Tra-1–60 panel of [2,3] despite representing results from different cell lines.

Regarding the concerns in Figs 1a and 2a, the corresponding author commented that the Fig 1a I-CSC panel and the Fig 2a panels present composite images. The individual image data used to compile these panels are provided in the S1–6 Files. The corresponding author clarified that the quantified results presented in Fig 2b were not measured directly from the composite image, but from original raw images of these cells (S4–6 Files). The authors provided an updated Fig 2 presenting representative images used for the cell size quantification. Composite images presenting unmarked grouping of image elements obtained from different fields or different parts of the same image is a breach of the *PLOS One* Image Manipulation guidelines in place at the time this article was submitted.

Regarding the concern with Fig 6B, the corresponding author stated that an incorrect panel was used during the preparation of this figure and provided an updated Fig 6.

The originally published Fig 6b I-CSC TRA-1–60 panel reports material from [2,3] published in 2012 by Elsevier, which is not offered under a CC BY license and is therefore excluded from this article’s [1] license.

The *PLOS One* Editors issue this Expression of Concern to notify readers of the above concerns and to relay the available data provided by the corresponding author.

## Supporting information

S1 File Original image data underlying Figs 1a-d. (ZIP)

S2 File Underlying data Fig 2a - Individual cropped images eCSC (the Editors note that “Picture 3” and “Picture 13” appear similar). (ZIP)

S3 File Underlying data Fig 2a - Individual cropped images ICSC. (ZIP)

S4 File Underlying data Fig 2a - l-CSC full collection of raw images part 1. (ZIP)

S5 File Underlying data Fig 2a - l-CSC full collection of raw images part 2. (ZIP)

S6 File Underlying data Fig 2a - l-CSC full collection of raw images part 3. (ZIP)

S7 File Individual level data underlying Figs 2b–e. (ZIP)

S8 File Original image data underlying Fig 6b. (ZIP)
