# Supplementary figures and images for: Ontological Differences in First Compared to Third Trimester Human Fetal Placental Chorionic Stem Cells
Source: PLoS One. 2012 Sep 4;7(9):e43395. doi: 10.1371/journal.pone.0043395 (PMC3433473; doi:10.1371/journal.pone.0043395)

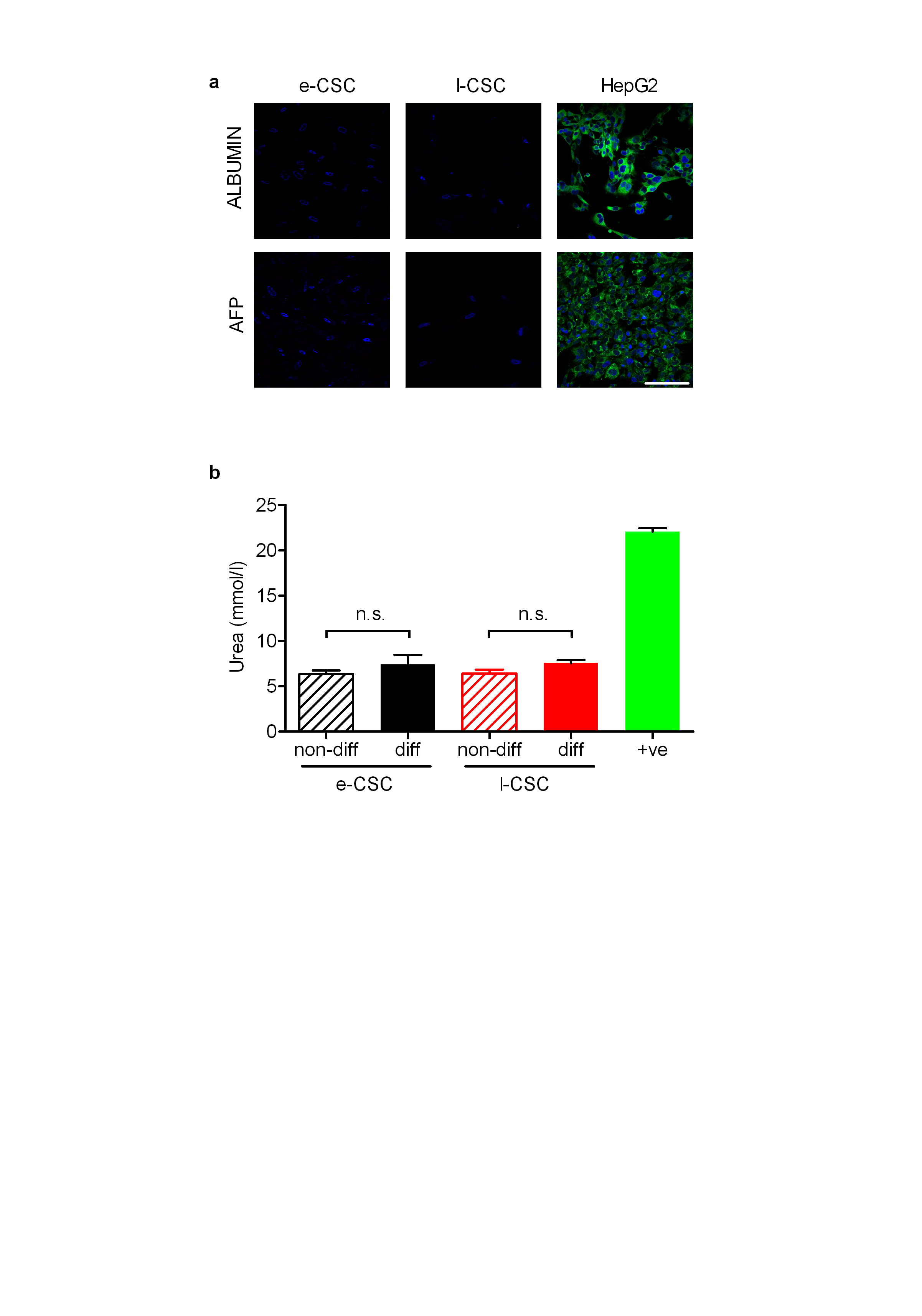

Supplement: Figure S1 — e-CSC and l-CSC hepatogenic differentiation. (a) Confocal immuno-fluorescence for ALBUMIN and AFP in e-CSC and l-CSC grown in hepatogenic permissive media for 3 weeks. HepG3 cells used for positive control. All genes stained with FITC (green). Nuclei stained with DAPI (blue). Scale bars 100 µm. (b) Presence of urea (mmol/l) in hepatogenic differentiation media (diff) of e-CSC and l-CSC. Positive (urea; according to the manufacturer's instructions) and negative controls; non-differentiated (non-diff) e-CSC and l-CSC are shown. n = 4 per cell group. Data. n.s. (not significant), Student's t test. Mean ± s.e.m. (TIF) [file pone.0043395.s001.tif]

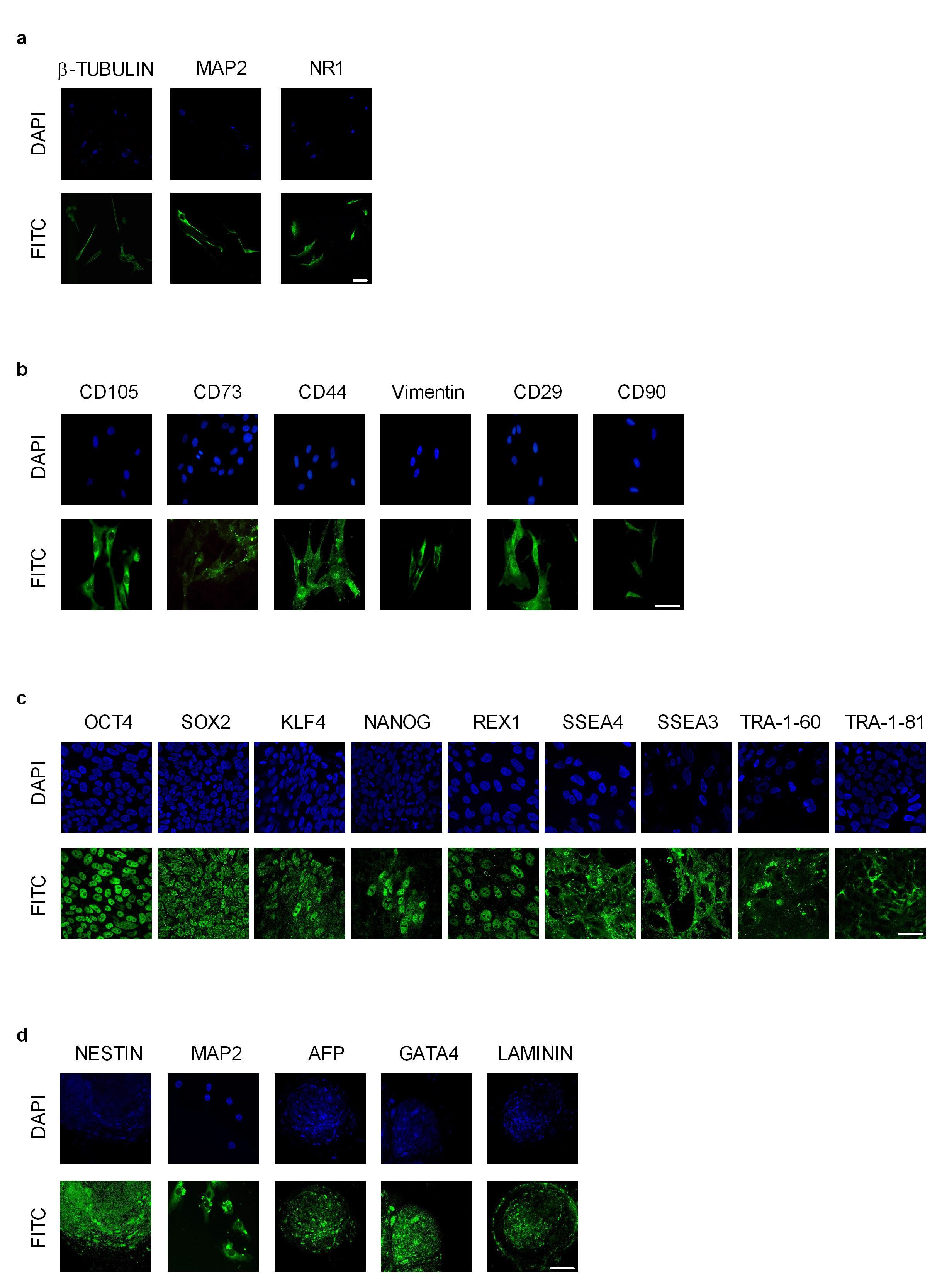

Supplement: Figure S2 — Positive controls for confocal immuno-fluorescence. (a) Positive controls (differentiated neural progenitors) of neuronal differentiation for expression of β-TUBULIN, MAP2 and NR1. (b) Positive controls (human first trimester fetal bone marrow mesenchymal stem cells, hfMSC) CD105, CD73, CD44, CD29 and CD90 (c) Positive controls (hES cells line H9) OCT4A, SOX2, KLF4, NANOG, REX1, SSEA4, SSEA3, TRA-1-60, and TRA-1-81 (d) Positive controls (pluripotent-induced human amniotic fluid stem cells-derived EBs) NESTIN, MAP2, AFP, GATA4 and LAMININ. All genes stained with FITC (green). Nuclei stained with DAPI (blue). Scale bars 50 µm. (TIF) [file pone.0043395.s002.tif]

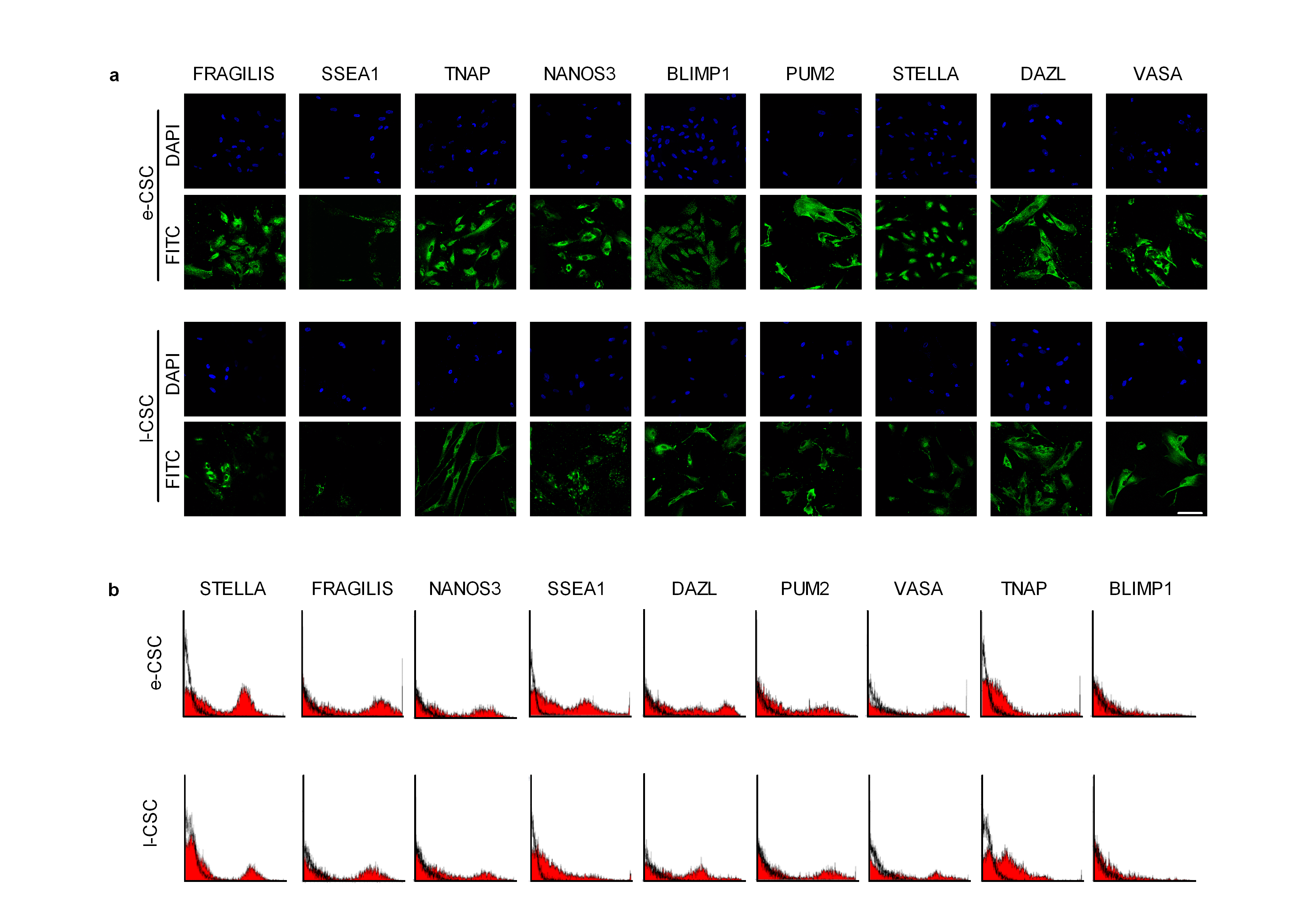

Supplement: Figure S3 — e-CSC and l-CSC express PGC associated markers. (a) Representative confocal immuno-fluorescence for protein expression of FRAGILIS, SSEA1, TNAP, NANOS3, BLIMP1, PUM2, STELLA, DAZL and VASA stained with FITC (green). Nuclei stained with DAPI (blue). Scale bar 100 µm. (b) Representative flow cytometry for PGC markers; STELLA, FRAGILIS, NANOS3, SSEA1, DAZL, PUM2, VASA, TNAP and BLIMP1 (isotype control in black). (TIF) [file pone.0043395.s003.tif]

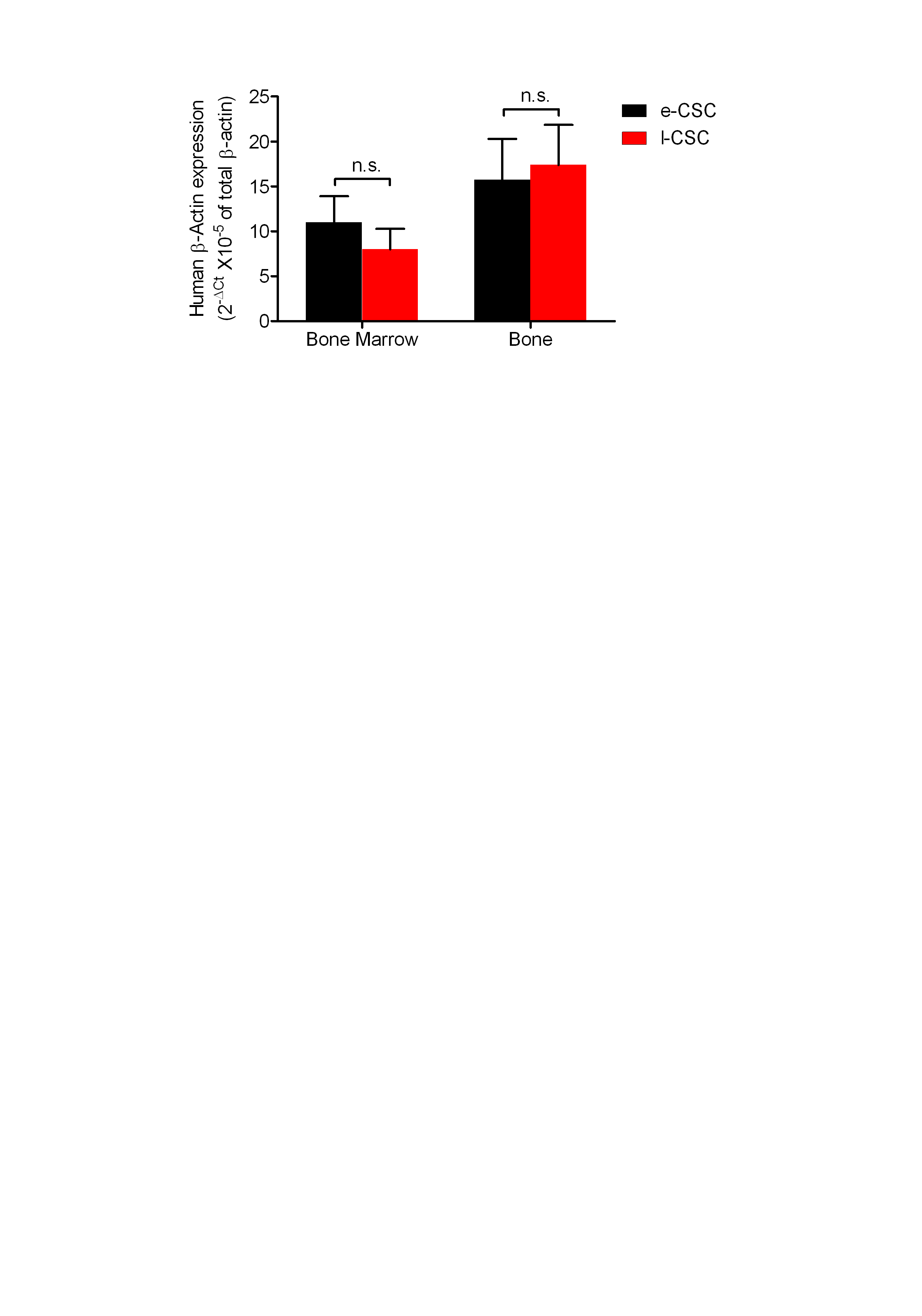

Supplement: Figure S4 — Engraftment of e-CSC and l-CSC in 8 week-old transplanted oim mice. Donor cell engraftment in bone marrow and bone was calculated as the 2−ΔCt of human specific β-actin normalised to human-mouse non-specific β-actin and using quantitative real time PCR in oim mice transplanted with e-CSC (black) or l-CSC (red). (TIF) [file pone.0043395.s004.tif]
