# Supplementary material for: Ontological Differences in First Compared to Third Trimester Human Fetal Placental Chorionic Stem Cells
Source: PLoS One. 2012 Sep 4;7(9):e43395. doi: 10.1371/journal.pone.0043395 (PMC3433473; doi:10.1371/journal.pone.0043395)
Supplement: Table S1 — Antibodies used. List of antibodies used for immuno-fluorescence (IF), flow cytometry (FC) and immuno-histochemistry (IH). (DOC) [file pone.0043395.s005.doc]

Table S1

| **Antibody** | **Host Species** | **Application** | **Dilution** | **Supplier** |
| --- | --- | --- | --- | --- |
| AFP | Mouse | IF | 1:100 | Abcam ([http://www.abcam.com](http://www.abcam.com/)) |
| ALBUMIN | Mouse | IF | 1:100 | Abcam |
| Β-TUBULIN | Mouse | IF | 1:100 | Chemicon ([http://www.millipore.com](http://www.millipore.com/)) |
| BLIMP1 | Goat | IF | 1:5 | Santa Cruz |
| CD105 | Mouse | IF | 1:50 | BD Biosciences |
| CD106-PE-Cy5 | Mouse | FC | 1:10 | BD Biosciences |
| CD11a-FITC | Mouse | FC | 1:10 | BD Biosciences |
| CD14 | Mouse | IF | 1:100 | Dako Cytomation |
| CD24-FITC | Mouse | FC | 1:10 | Abcam |
| CD29 | Mouse | IF | 1:100 | Abcam |
| CD29-PE | Mouse | FC | 1:10 | BD Biosciences |
| CD34 | Mouse | IF | 1:100 | Dako Cytomation |
| CD44 | Mouse | IF | 1:50 | Abcam |
| CD45 | Mouse | IF | 1:100 | Dako Cytomation |
| CD49b-FITC | Mouse | FC | 1:10 | BD Biosciences |
| CD49d-PE-Cy5 | Mouse | FC | 1:10 | BD Biosciences |
| CD49e-PE | Mouse | FC | 1:10 | BD Biosciences |
| CD49f-PE-Cy5 | Rat | FC | 1:10 | BD Biosciences |
| CD51/61-FITC | Mouse | FC | 1:10 | BD Biosciences |
| CD62P-PE | Mouse | FC | 1:10 | BD Biosciences |
| CD73 | Mouse | IF | 1:50 | BD Biosciences ([http://www.bdbiosciences.com](http://www.bdbiosciences.com/)) |
| CD90 | Mouse | IF | 1:100 | Abcam |
| C-KIT-PE | Mouse | FC | 1:10 | R&D Systems |
| C-MYC-PE | Mouse | FC | 1:20 | R&D Systems |
| COL1A2 | Rabbit | WB | 1:1000 | Abcam |
| CXCR4-PE | Mouse | FC | 1:10 | R&D Systems |
| DAZL | Goat | IF | 1:10 | Santa Cruz |
| FRAGILIS | Goat | IF | 1:20 | Santa Cruz |
| GATA4 | Rabbit | IF | 1:100 | Abcam |
| INTEGRIN β3-PE | Mouse | FC | 1:10 | R&D Systems |
| INTEGRIN β7-PE | Rat | FC | 1:10 | BD Biosciences |
| KLF4 | Mouse | IF | 1:2 | Stemgent ([http://www.stemgent.com](http://www.stemgent.com/)) |
| LAMININ | Rabbit | IF | 1:100 | Sigma ([http://www.sigmaaldrich.com](http://www.sigmaaldrich.com/)) |
| MAP2 | Mouse | IF | 1:100 | Abcam |
| NANOG | Rabbit | IF | 1:20 | Abcam |
| NANOS 3 | Goat | IF | 1:20 | Santa Cruz |
| NESTIN | Mouse | IF | 1:50 | Chemicon ([http://www.millipore.com](http://www.millipore.com/)) |
| NMDAR1 | Rabbit | IF | 1:50 | Abcam |
| OCT-4A | Mouse | IF | 1:2 | Santa Cruz (http://www.scbt.com) |
| OCT-4A-PE | Mouse | FC | 1:10 | R&D Systems (http://www.rndsystems.com) |
| PUM2 | Goat | IF | 1:20 | Santa Cruz |
| REX1 | Rabbit | IF | 1:100 | Stemgent |
| SOX2 | Rabbit | IF | 1:100 | Abcam |
| SOX2-PE | Mouse | FC | 1:10 | R&D Systems |
| SSEA1 | Mouse | IF | 1:50 | Chemicon |
| SSEA3 | Rat | IF | 1:50 | Abcam |
| SSEA3 -PE | Mouse | FC | 1:5 | Dev. Studies Hybridoma Bank (http://dshb.biology.uiowa.edu) |
| SSEA4 | Mouse | IF | 1:50 | Abcam |
| SSEA4-PE | Mouse | FC | 1:10 | R&D Systems |
| STELLA | Rabbit | IF | 1:50 | Santa Cruz |
| TNAP | Mouse | IF | 1:50 | Abcam |
| TRA-1-60 | Mouse | IF, FC | 1:10 | Santa Cruz |
| TRA-1-81 | Mouse | IF, FC | 1:10 | Santa Cruz |
| VASA (DDX4) | Rabbit | IF | 1:50 | Abcam |
| VIMENTIN | Mouse | IF | 1:100 | Dako Cytomation (http://www.dako.com) |
| αV INTEGRIN-PE | Mouse | FC | 1:10 | R&D Systems |
| Β-ACTIN | Rabbit | WB | 1:5000 | Abcam |
