# Supplementary material for: Ontological Differences in First Compared to Third Trimester Human Fetal Placental Chorionic Stem Cells
Source: PLoS One. 2012 Sep 4;7(9):e43395. doi: 10.1371/journal.pone.0043395 (PMC3433473; doi:10.1371/journal.pone.0043395)
Supplement: Table S2 — Primers used. List of primers used for RT-PCR and quantitative real time PCR and EpiTect Methyl qPCR assay. (DOC) [file pone.0043395.s006.doc]

**Table S2**

| **Gene** | **Forward** | **Reverse** | **Accession no.** |
| --- | --- | --- | --- |
| β-ACTIN  (Human) | 5’-CTGGAACGGTGAAGGTGACA-3’ | 5’-AAGGGACTTCCTGTAACAATGCA-3’ | NM_001101 |
| β-ACTIN  (Human-Mouse) | 5’-GCTCCTCCTGAGCGCAAGTA-3’ | 5’-GATGGAGGGGCCGGACT-3’ | NM_001101  NM_007393 |
| BMP2 | 5’-TTCCACCATGAAGAATCTTTGGA-3’ | 5’-CCTGAAGCTCTGCTGAGGTGAT-3’ | NM_001200 |
| BMP4 | 5’-GTGAGGAGCTTCCACCACGA-3’ | 5’-ACTGGTCCCTGGGATGTTCTC-3’ | NM_130850 |
| BSP | 5’-AAACGAAGAAAGCGAAGCAGAA-3’ | 5’-GCTGCCGTTGCCGTTTT-3’ | NM_004967 |
| CK19 | 5’-GGCCACACGGAGCAGCTCCA-3’ | 5’-CGCCTGGATATGCGCCAGCT-3’ | NM_002276 |
| CK3 | 5’-CGTACAGCTGCTGAGAATGA-3’ | 5’-CTGAGCGATATCCTCATACT-3’ | NM_057088 |
| c-KIT | 5’-CACCGAAGGAGGCACTTACAC-3’ | 5’-GGAATCCTGCTGCCACACA-3’ | NM_000222 |
| C-MYC | 5’-TTGTACCTGCAGGATCTGAG-3’ | 5’-CTTGTTCCTCCTCAGAGTCG-3’ | NM_002467 |
| FRAGILIS | 5’-CTCAAGGAGGAGCAGGAAGT-3’ | 5’-TGAAGAGGGTGTTGAACAGG-3’ | NM_006435 |
| GAPDH | 5’-TCTGCTCCTCCTGTTCGACA-3’ | 5’-AAAAGCAGCCCTGGTGACC-3’ | NM_002046 |
| GATA6 | 5’-ACCACCTTATGGCGCAGAAAC-3’ | 5’-TTTTTCATAGCAAGTGGTCTGGG-3’ | NM_005257 |
| H19 | Catalogue number: MePH28624-1B | Qiagen (http://www.qiagen.com) | NC_000011 |
| KLF4 | 5’-CACATTAATGAGGCAGCCACC-3’ | 5’-AAGTCGCTTCATGTGGGAGAG-3’ | NM_004235 |
| NANOG | 5’-CCAACATCCTGAACCTCAGCTAC-3’ | 5’-GCCTTCTGCGTCACACCATT-3’ | NM_024865 |
| NANOS3 | TaqMan Hs00928455_s1* | Applied Biosystems (http://www.appliedbiosystems.com) | NM_001098622 |
| NESTIN | 5’-TCCAGGAACGGAAAATCAAG-3’ | 5’-GCCTCCTCATCCCCTACTTC-3’ | NM_006617 |
| OC | 5’-CCTCACACTCCTCGCCCTATT-3’ | 5’-CCCTCCTGCTTGGACACAAA-3’ | NM_199173 |
| OCT4A | 5’-GTATTCAGCCAAACGACCATC-3’ | 5’-CTGGTTCGCTTTCTCTTTCG-3’ | NM_002701 |
| OCT4A variant1 | 5’- CCGCCGTATGAGTTCTGTGG-3’ | 5’- AGAGTGGTGACGGAGACAGG-3’ | NM_002701 |
| OP | 5’-GCCGACCAAGGAAAACTCACTA-3’ | 5’-CAGAACTTCCAGAATCAGCCTGTT-3’ | NM_001040058 |
| OSX | 5’-CCCCACCTCTTGCAACCA-3’ | 5’-GGCTCCACCACTCCCTTCTAG-3’ | NM_152860 |
| PAX6 | 5’-AGATTCAGATGAGGCTCAAA-3’ | 5’-AATTGGTTGGTAGACACTGG-3’ | NM_000280 |
| PCE | 5’-TTCGCTGGGTCTGGGACTT-3’ | 5’-CATCCTCAGGGTCACCTGGTT-3’ | NM_002593 |
| SOX2 | 5’- CACACTGCCCCTCTCACACAT-3’ | 5’- CATTTCCCTCGTTTTTCTTTGAA-3’ | NM_003106 |
| SRY | 5’-TGGCGATTAAGTCAAATTCGC-3’ | 5’-CCCCCTAGTACCCTGACAATGTATT-3’ | NM_003140 |
| SSEA1 | 5’-CACAACTGTTCCCGATTCAC-3’ | 5’-AGCCACAGTCCTTCCACTCT-3’ | NM_002033 |
| STELLA | TaqMan Hs01931905_g1* | Applied Biosystems | NM_199286 |
| SYCP1 | 5’-ACAGCGAAAAGCCATTCAGGA-3’ | 5’-GCCTGGTTTCTTCCCGTTCATA-3’ | NM_003176 |
| VASA | TaqMan Hs00987130_m1* | Applied Biosystems | NM_001166533 |
