# Supplementary figures and images for: Expression of Concern: Ontological Differences in First Compared to Third Trimester Human Fetal Placental Chorionic Stem Cells (part 1 of 2)
Source: PLoS One. 2025 Aug 1;20(8):e0329484. doi: 10.1371/journal.pone.0329484 (PMC12316261; doi:10.1371/journal.pone.0329484)

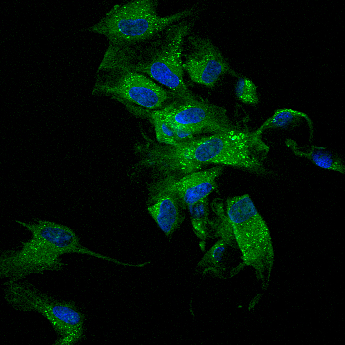

Supplement: S1 File — (ZIP) [file pone.0329484.s001.zip › S1 File/S1 File/Fig.1a/Fig1A. eCSC cKIT images/c-KIT eCSC.jpg]

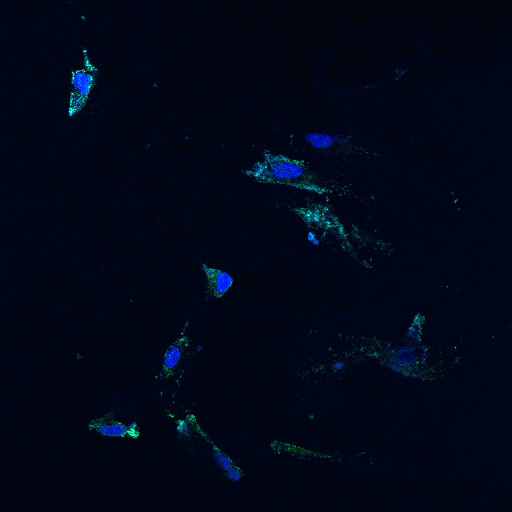

Supplement: S1 File — (ZIP) [file pone.0329484.s001.zip › S1 File/S1 File/Fig.1a/Fig1A. l-CSC cKIT images/Series035_z0.jpg]

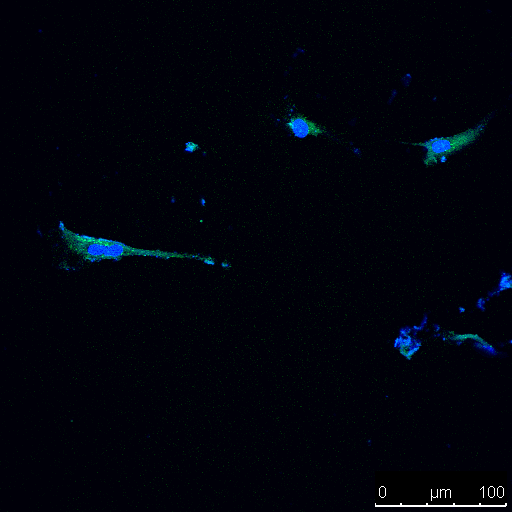

Supplement: S1 File — (ZIP) [file pone.0329484.s001.zip › S1 File/S1 File/Fig.1a/Fig1A. l-CSC cKIT images/Series078_z0.tif]

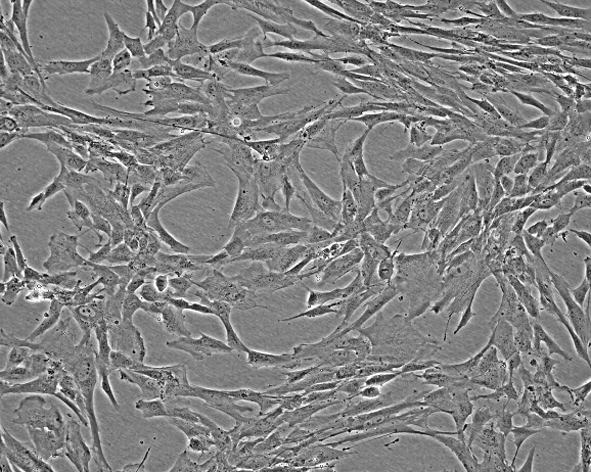

Supplement: S1 File — (ZIP) [file pone.0329484.s001.zip › S1 File/S1 File/Fig.1b/eCSC fig1b.tif]

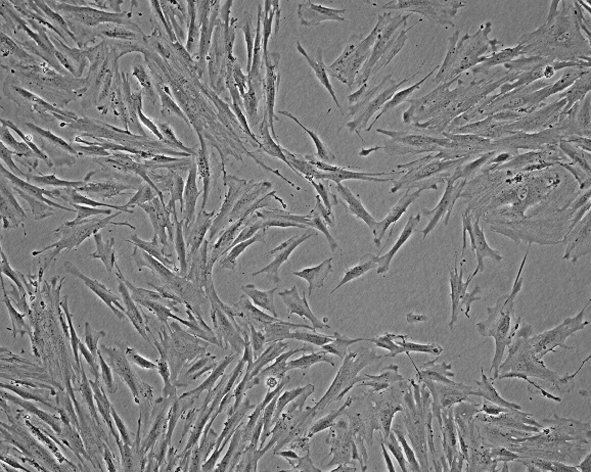

Supplement: S1 File — (ZIP) [file pone.0329484.s001.zip › S1 File/S1 File/Fig.1b/l-CSC fig1b.tif]

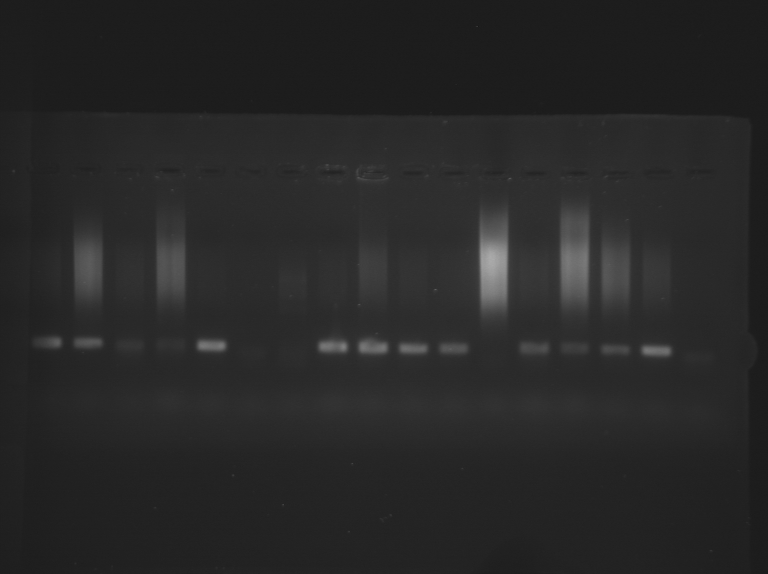

Supplement: S1 File — (ZIP) [file pone.0329484.s001.zip › S1 File/S1 File/Fig.1c/eCSC SRY.bmp]

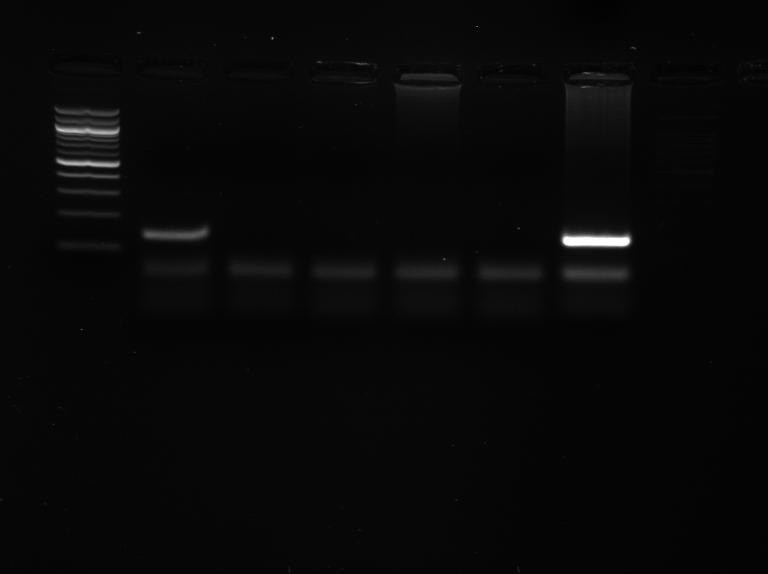

Supplement: S1 File — (ZIP) [file pone.0329484.s001.zip › S1 File/S1 File/Fig.1c/l-CSC SRY.TIF]

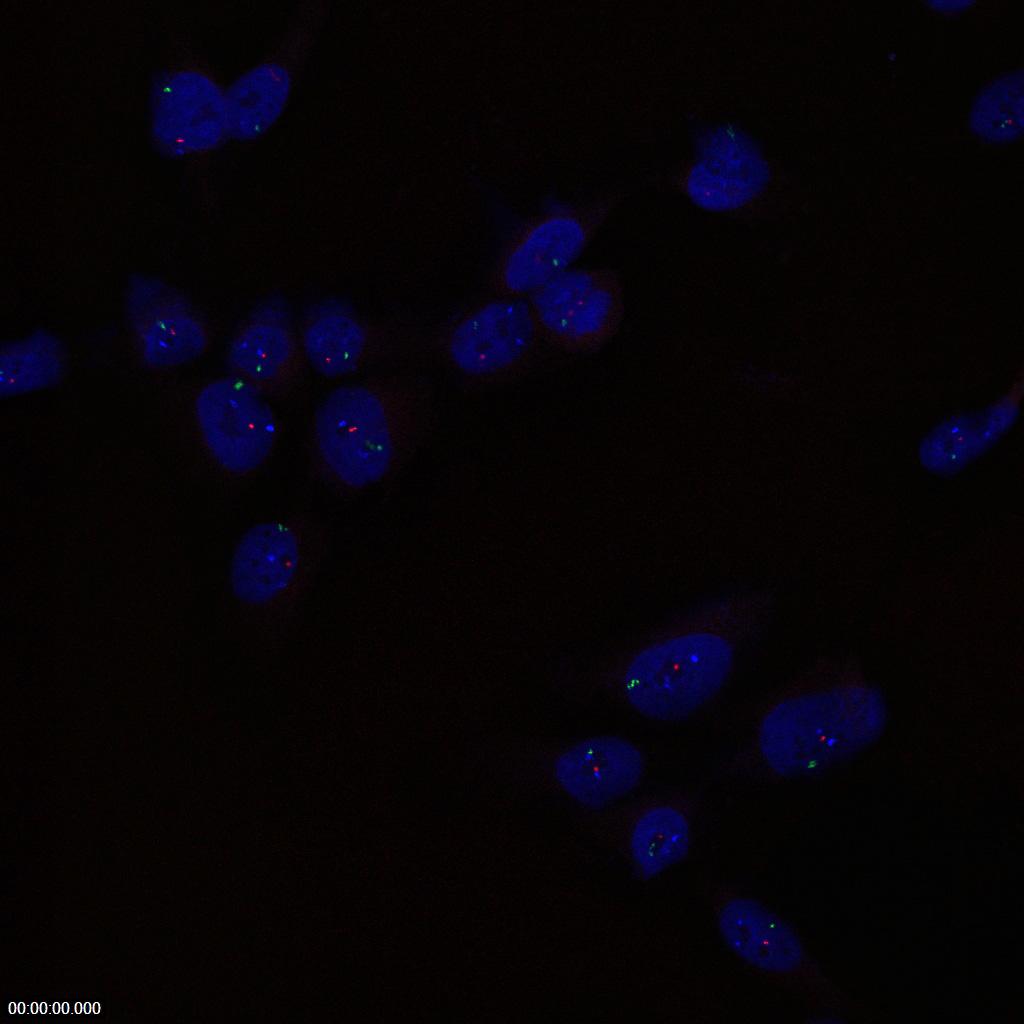

Supplement: S1 File — (ZIP) [file pone.0329484.s001.zip › S1 File/S1 File/Fig.1d/e-CSC/Series061_z0.jpg]

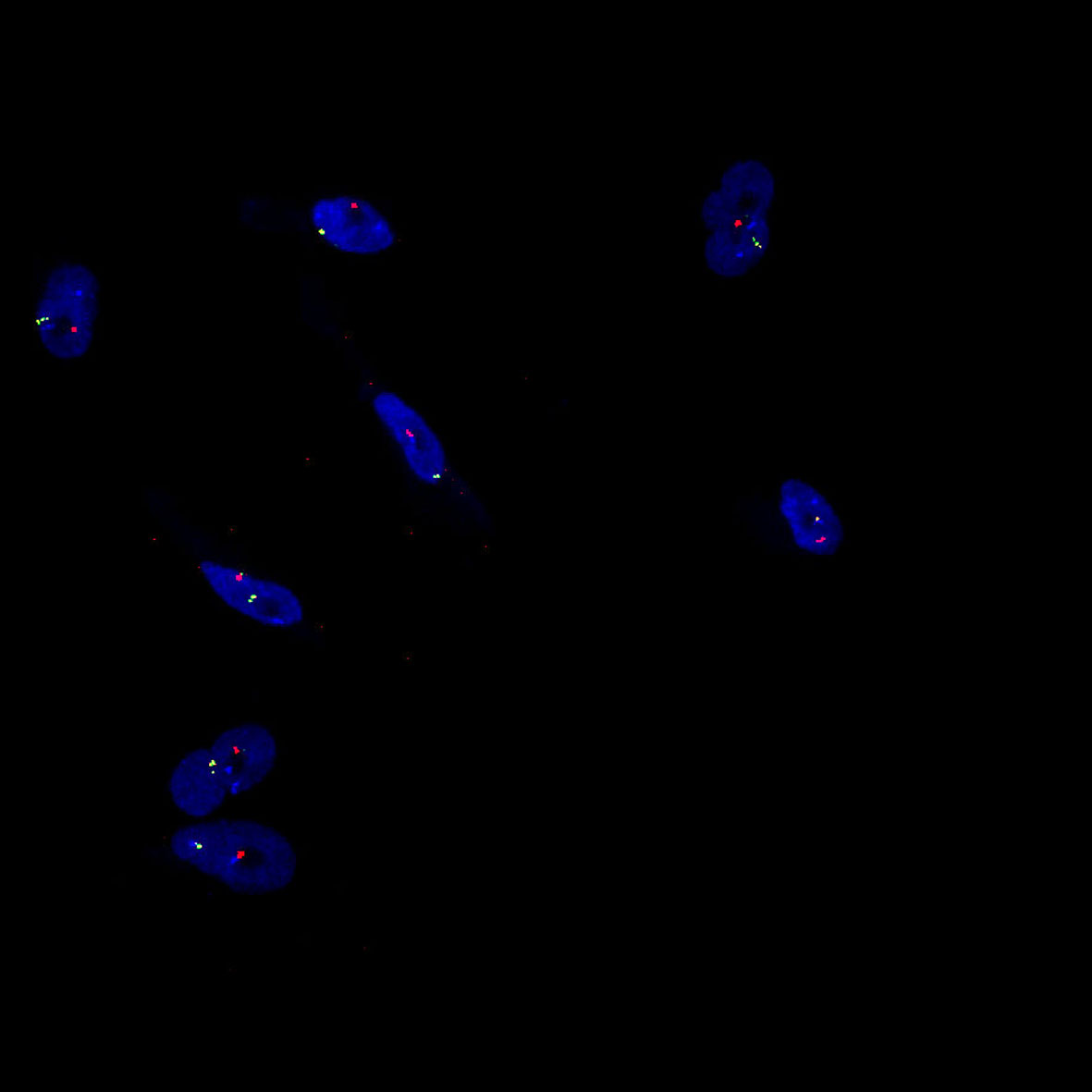

Supplement: S1 File — (ZIP) [file pone.0329484.s001.zip › S1 File/S1 File/Fig.1d/l-CSC/l-CSC FISH in publication.jpg]

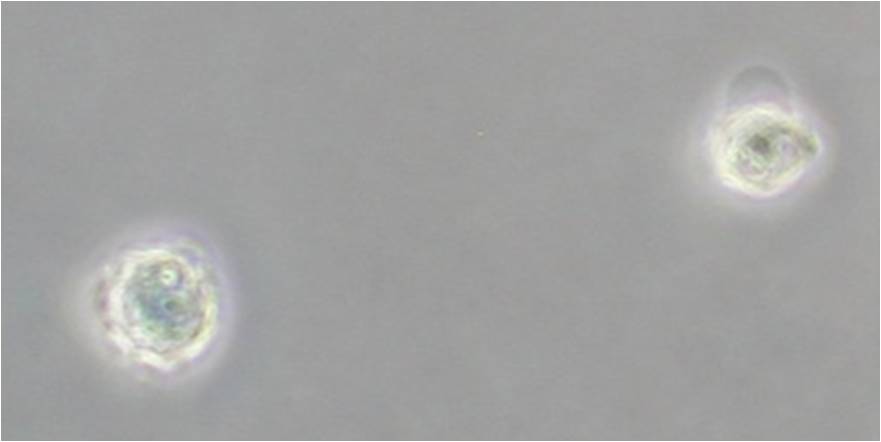

Supplement: S2 File — (ZIP) [file pone.0329484.s002.zip › S2 File - individual cropped images eCSC/individual cropped images eCSC/Picture1.jpg]

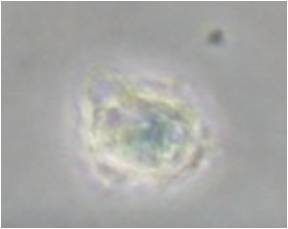

Supplement: S2 File — (ZIP) [file pone.0329484.s002.zip › S2 File - individual cropped images eCSC/individual cropped images eCSC/Picture11.jpg]

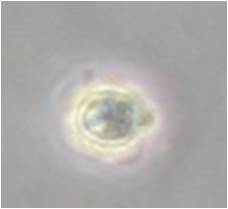

Supplement: S2 File — (ZIP) [file pone.0329484.s002.zip › S2 File - individual cropped images eCSC/individual cropped images eCSC/Picture12.jpg]

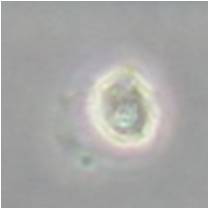

Supplement: S2 File — (ZIP) [file pone.0329484.s002.zip › S2 File - individual cropped images eCSC/individual cropped images eCSC/Picture13.jpg]

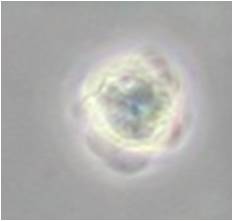

Supplement: S2 File — (ZIP) [file pone.0329484.s002.zip › S2 File - individual cropped images eCSC/individual cropped images eCSC/Picture14.jpg]

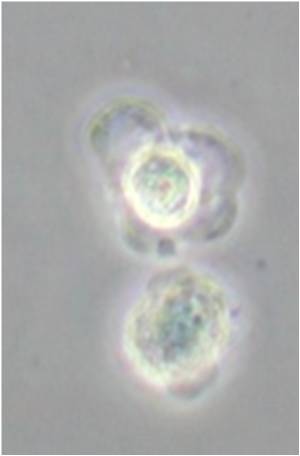

Supplement: S2 File — (ZIP) [file pone.0329484.s002.zip › S2 File - individual cropped images eCSC/individual cropped images eCSC/Picture15.jpg]

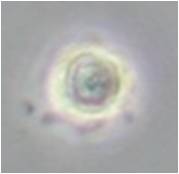

Supplement: S2 File — (ZIP) [file pone.0329484.s002.zip › S2 File - individual cropped images eCSC/individual cropped images eCSC/Picture16.jpg]

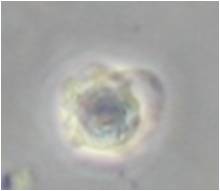

Supplement: S2 File — (ZIP) [file pone.0329484.s002.zip › S2 File - individual cropped images eCSC/individual cropped images eCSC/Picture17.jpg]

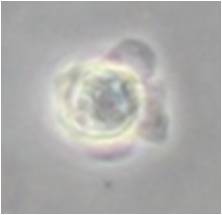

Supplement: S2 File — (ZIP) [file pone.0329484.s002.zip › S2 File - individual cropped images eCSC/individual cropped images eCSC/Picture20.jpg]

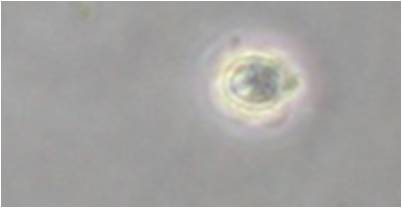

Supplement: S2 File — (ZIP) [file pone.0329484.s002.zip › S2 File - individual cropped images eCSC/individual cropped images eCSC/Picture21.jpg]

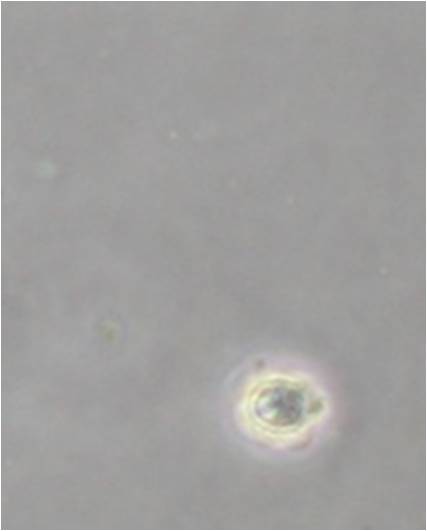

Supplement: S2 File — (ZIP) [file pone.0329484.s002.zip › S2 File - individual cropped images eCSC/individual cropped images eCSC/Picture22.jpg]

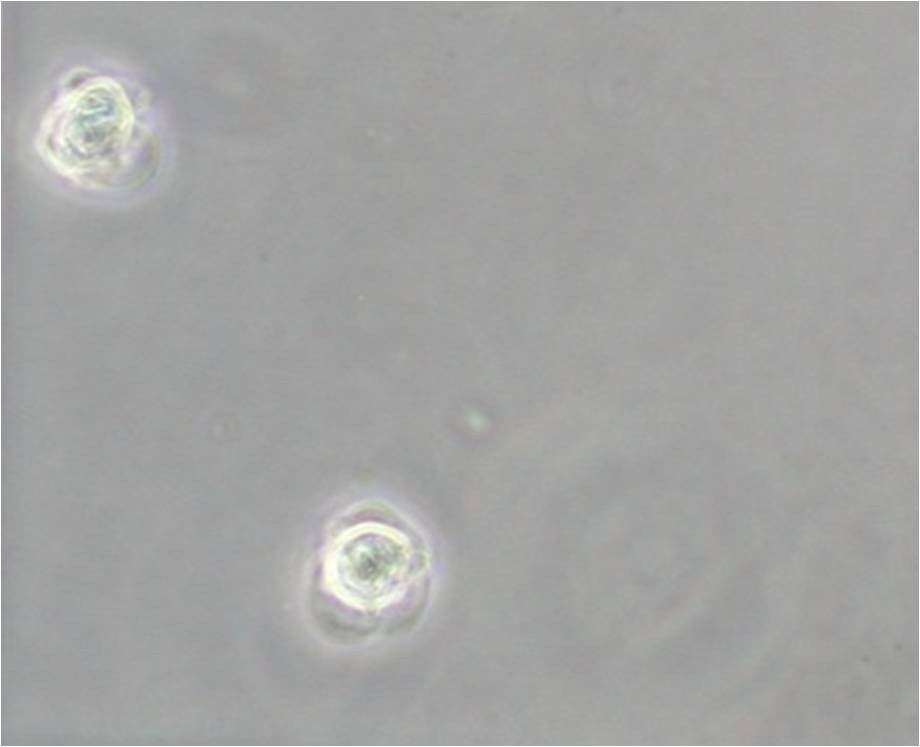

Supplement: S2 File — (ZIP) [file pone.0329484.s002.zip › S2 File - individual cropped images eCSC/individual cropped images eCSC/Picture3.jpg]

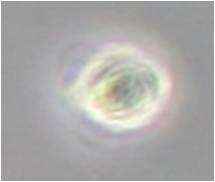

Supplement: S2 File — (ZIP) [file pone.0329484.s002.zip › S2 File - individual cropped images eCSC/individual cropped images eCSC/Picture4.jpg]

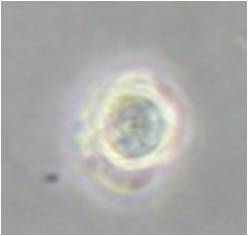

Supplement: S2 File — (ZIP) [file pone.0329484.s002.zip › S2 File - individual cropped images eCSC/individual cropped images eCSC/Picture5.jpg]

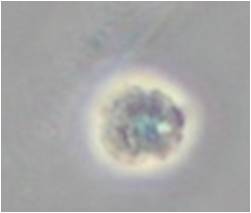

Supplement: S2 File — (ZIP) [file pone.0329484.s002.zip › S2 File - individual cropped images eCSC/individual cropped images eCSC/Picture6.jpg]

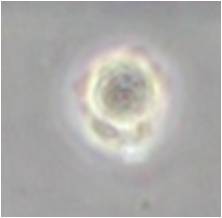

Supplement: S2 File — (ZIP) [file pone.0329484.s002.zip › S2 File - individual cropped images eCSC/individual cropped images eCSC/Picture7.jpg]

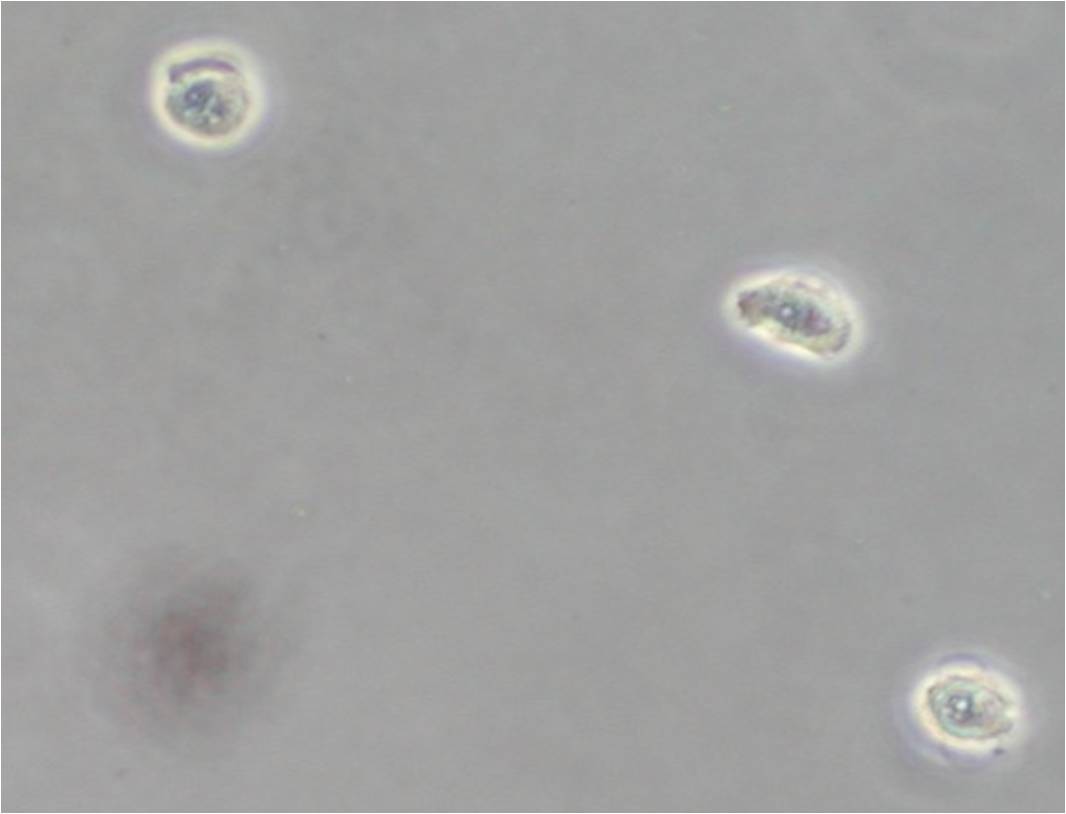

Supplement: S2 File — (ZIP) [file pone.0329484.s002.zip › S2 File - individual cropped images eCSC/individual cropped images eCSC/Picture8.jpg]

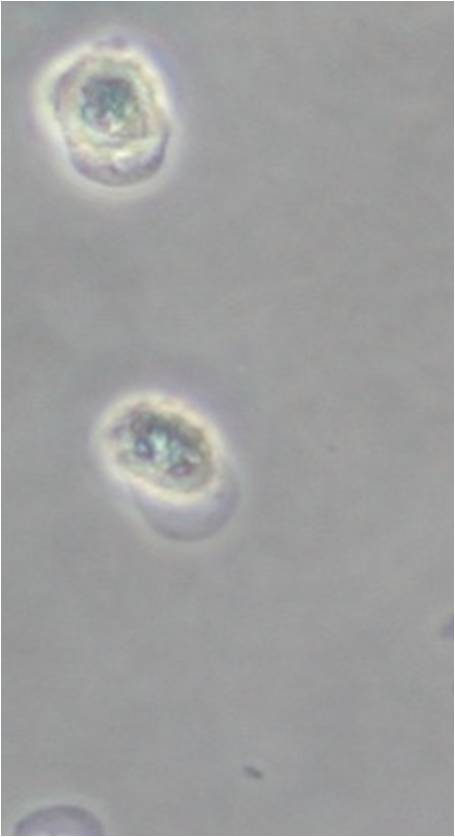

Supplement: S3 File — (ZIP) [file pone.0329484.s003.zip › S3 File - individual cropped images lCSC/individual cropped images lCSC/Picture1.jpg]

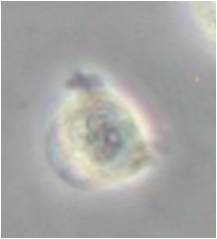

Supplement: S3 File — (ZIP) [file pone.0329484.s003.zip › S3 File - individual cropped images lCSC/individual cropped images lCSC/Picture10.jpg]

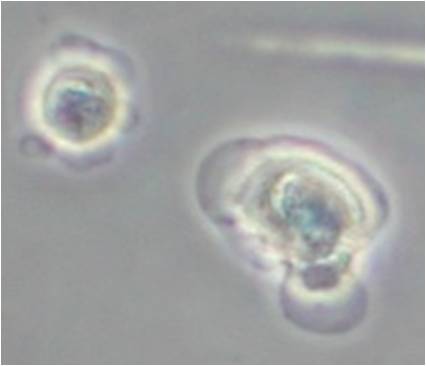

Supplement: S3 File — (ZIP) [file pone.0329484.s003.zip › S3 File - individual cropped images lCSC/individual cropped images lCSC/Picture11.jpg]

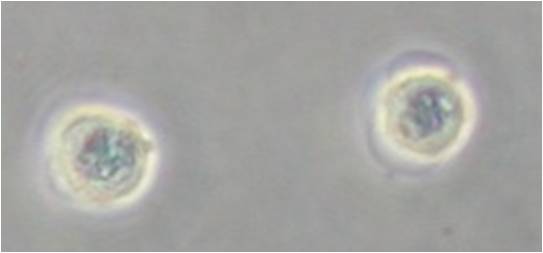

Supplement: S3 File — (ZIP) [file pone.0329484.s003.zip › S3 File - individual cropped images lCSC/individual cropped images lCSC/Picture12.jpg]

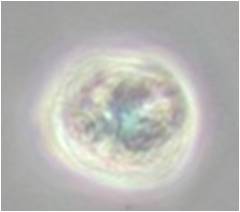

Supplement: S3 File — (ZIP) [file pone.0329484.s003.zip › S3 File - individual cropped images lCSC/individual cropped images lCSC/Picture13.jpg]

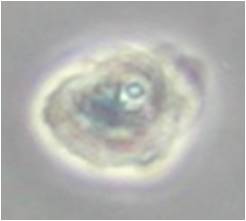

Supplement: S3 File — (ZIP) [file pone.0329484.s003.zip › S3 File - individual cropped images lCSC/individual cropped images lCSC/Picture14.jpg]

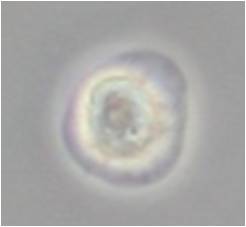

Supplement: S3 File — (ZIP) [file pone.0329484.s003.zip › S3 File - individual cropped images lCSC/individual cropped images lCSC/Picture2.jpg]

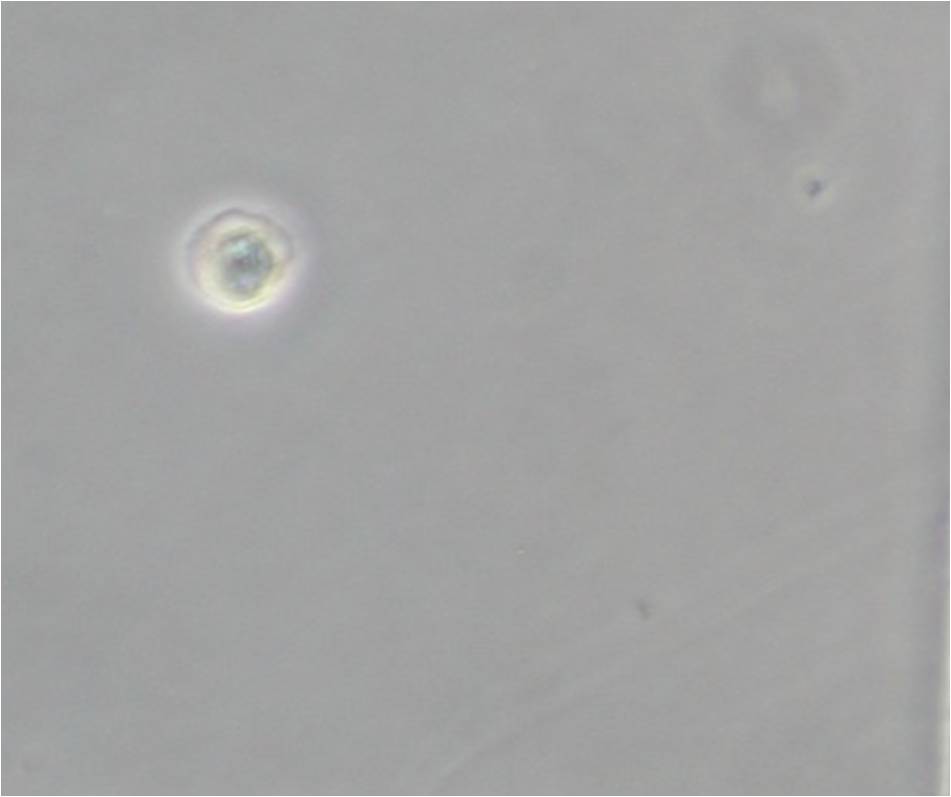

Supplement: S3 File — (ZIP) [file pone.0329484.s003.zip › S3 File - individual cropped images lCSC/individual cropped images lCSC/Picture3.jpg]

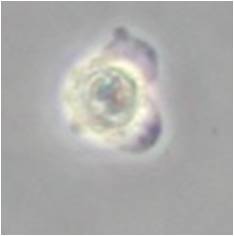

Supplement: S3 File — (ZIP) [file pone.0329484.s003.zip › S3 File - individual cropped images lCSC/individual cropped images lCSC/Picture4.jpg]

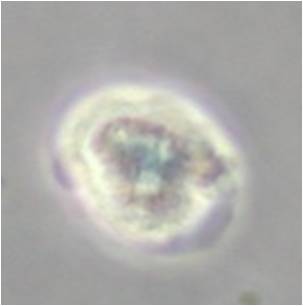

Supplement: S3 File — (ZIP) [file pone.0329484.s003.zip › S3 File - individual cropped images lCSC/individual cropped images lCSC/Picture5.jpg]

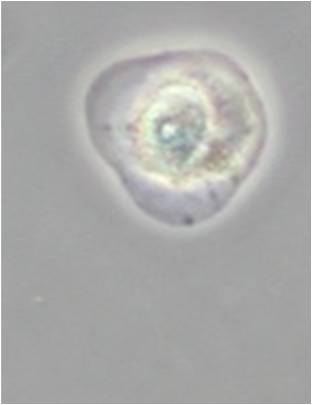

Supplement: S3 File — (ZIP) [file pone.0329484.s003.zip › S3 File - individual cropped images lCSC/individual cropped images lCSC/Picture6.jpg]

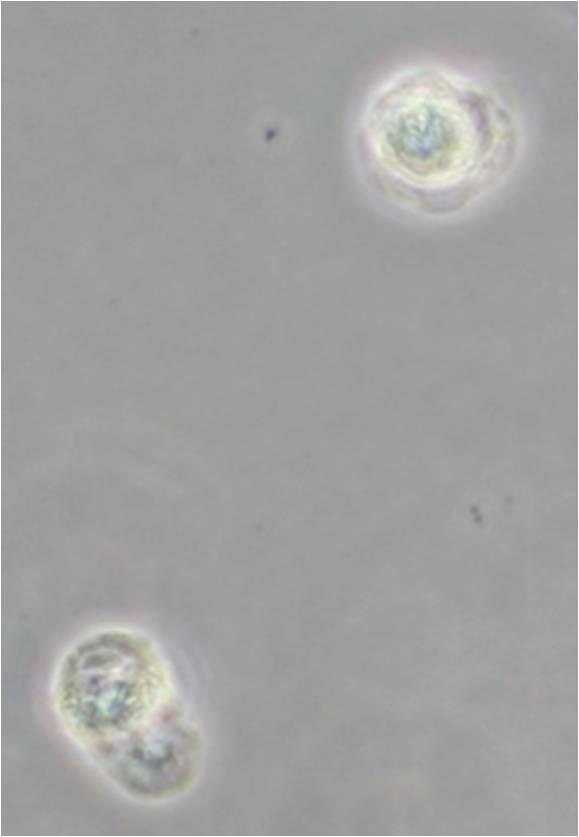

Supplement: S3 File — (ZIP) [file pone.0329484.s003.zip › S3 File - individual cropped images lCSC/individual cropped images lCSC/Picture7.jpg]

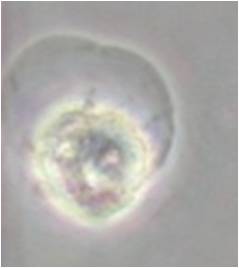

Supplement: S3 File — (ZIP) [file pone.0329484.s003.zip › S3 File - individual cropped images lCSC/individual cropped images lCSC/Picture8.jpg]

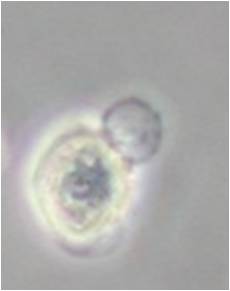

Supplement: S3 File — (ZIP) [file pone.0329484.s003.zip › S3 File - individual cropped images lCSC/individual cropped images lCSC/Picture9.jpg]

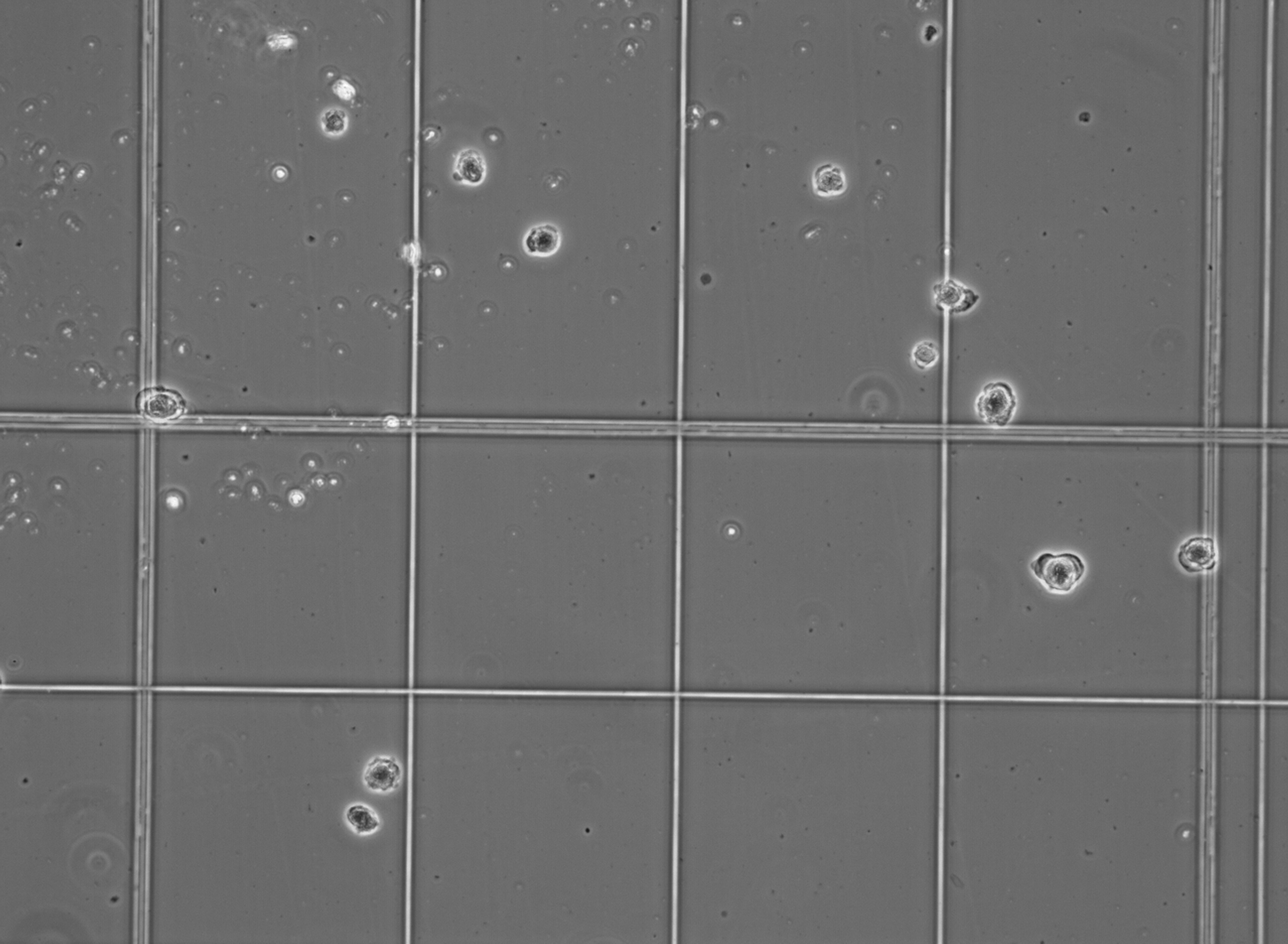

Supplement: S4 File — (ZIP) [file pone.0329484.s004.zip › S4 File - l-CSC 1/l-CSC 1/untitled034.tif]

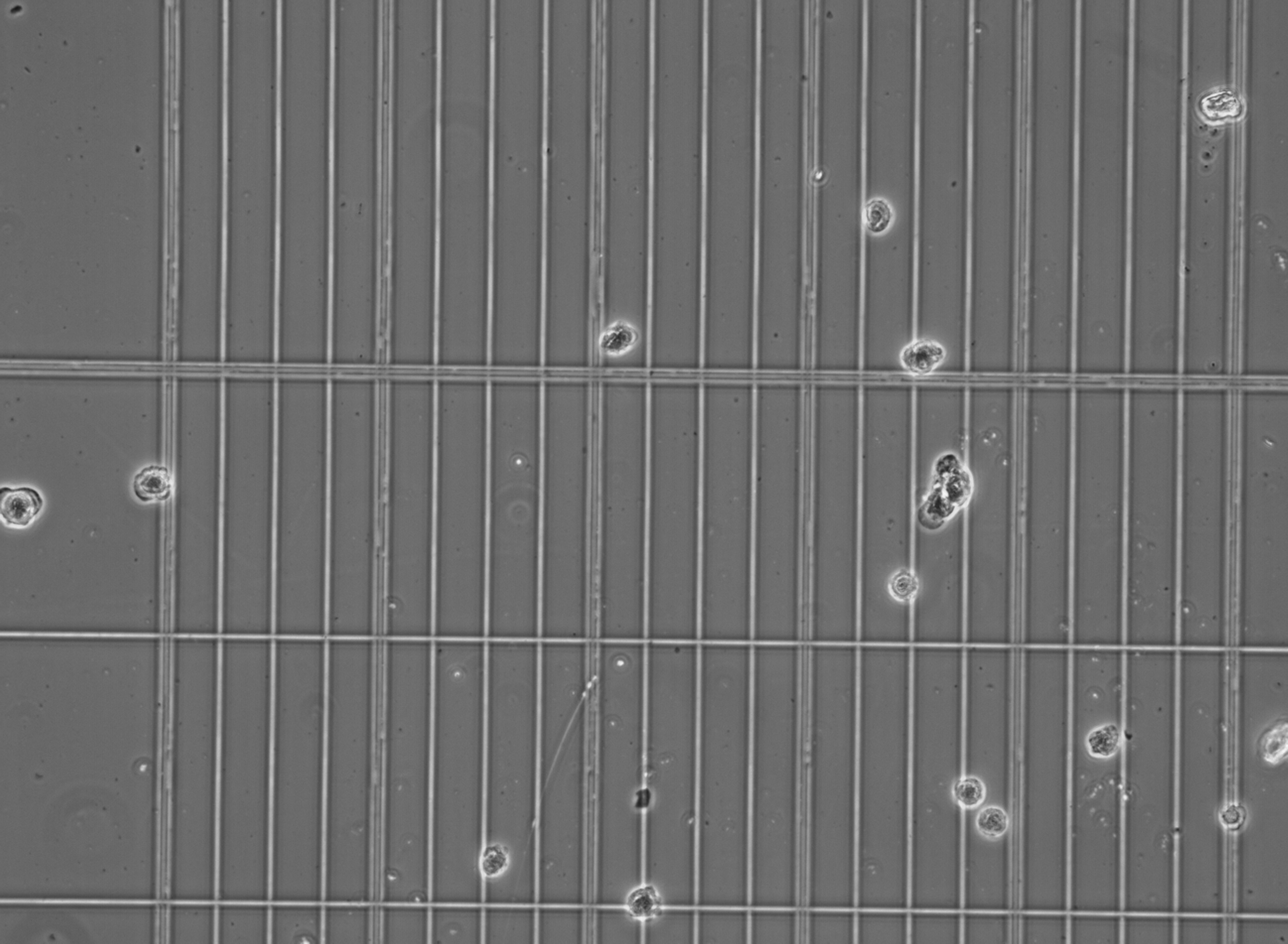

Supplement: S4 File — (ZIP) [file pone.0329484.s004.zip › S4 File - l-CSC 1/l-CSC 1/untitled035.tif]

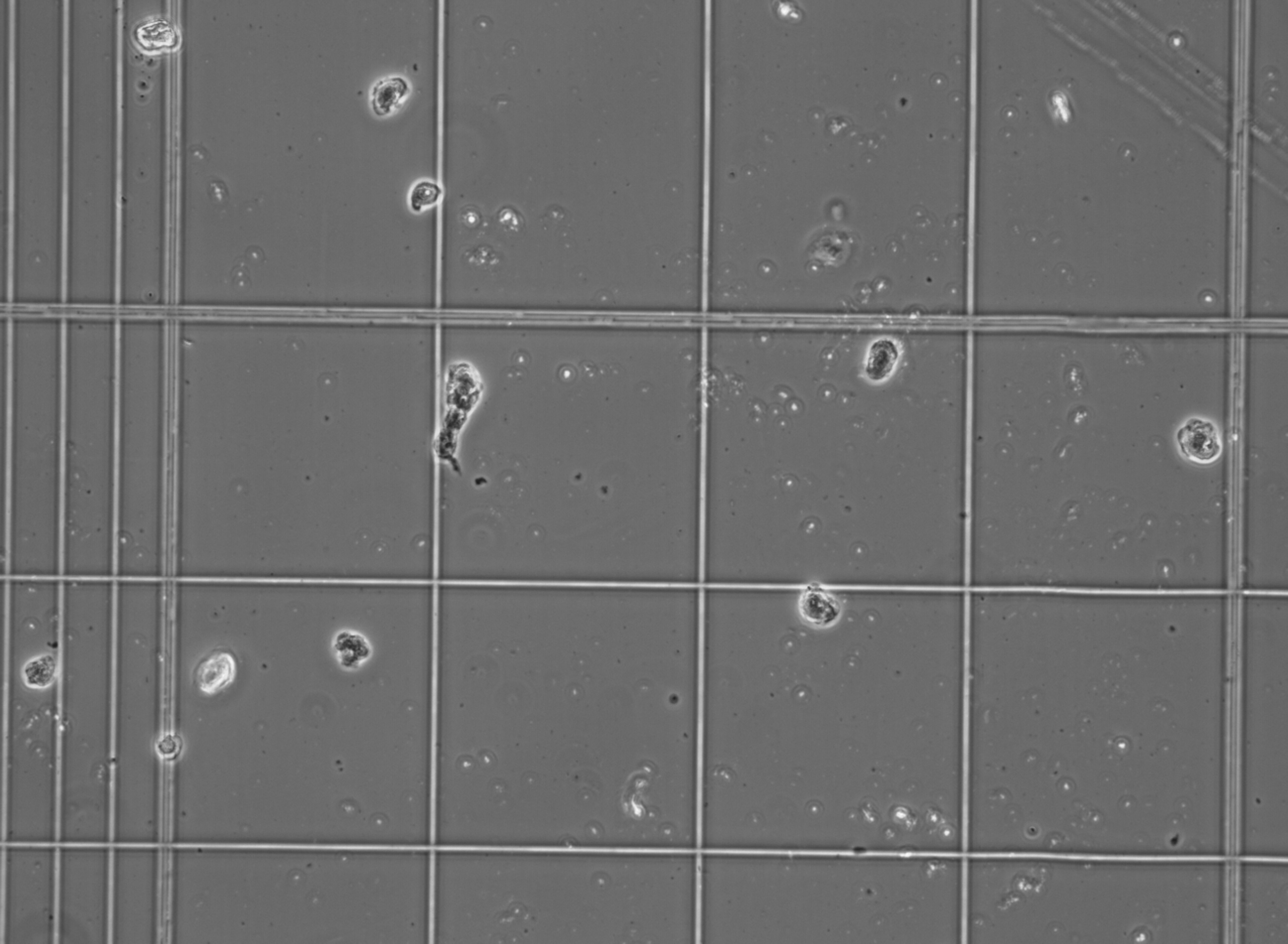

Supplement: S4 File — (ZIP) [file pone.0329484.s004.zip › S4 File - l-CSC 1/l-CSC 1/untitled036.tif]

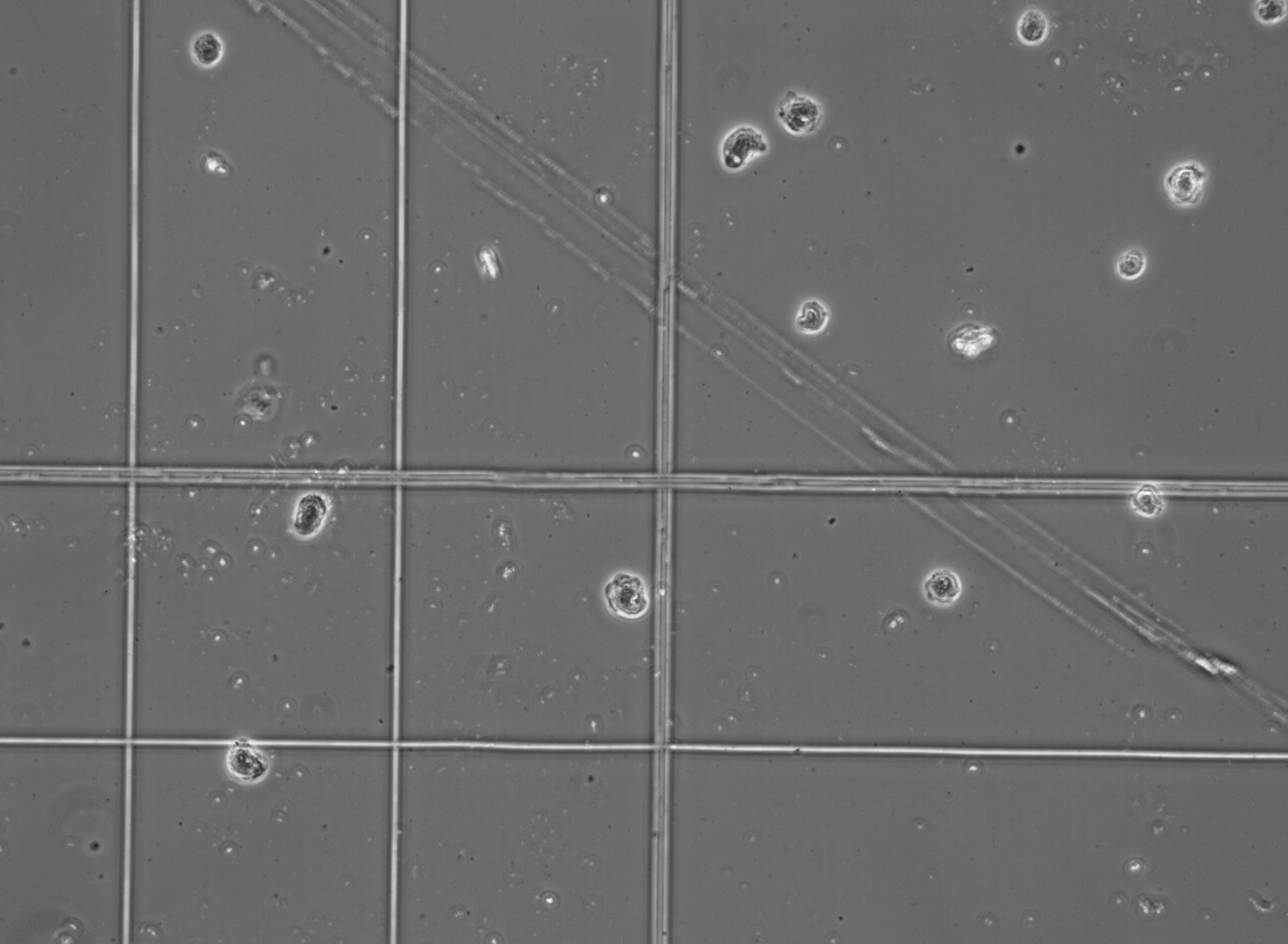

Supplement: S4 File — (ZIP) [file pone.0329484.s004.zip › S4 File - l-CSC 1/l-CSC 1/untitled037.tif]

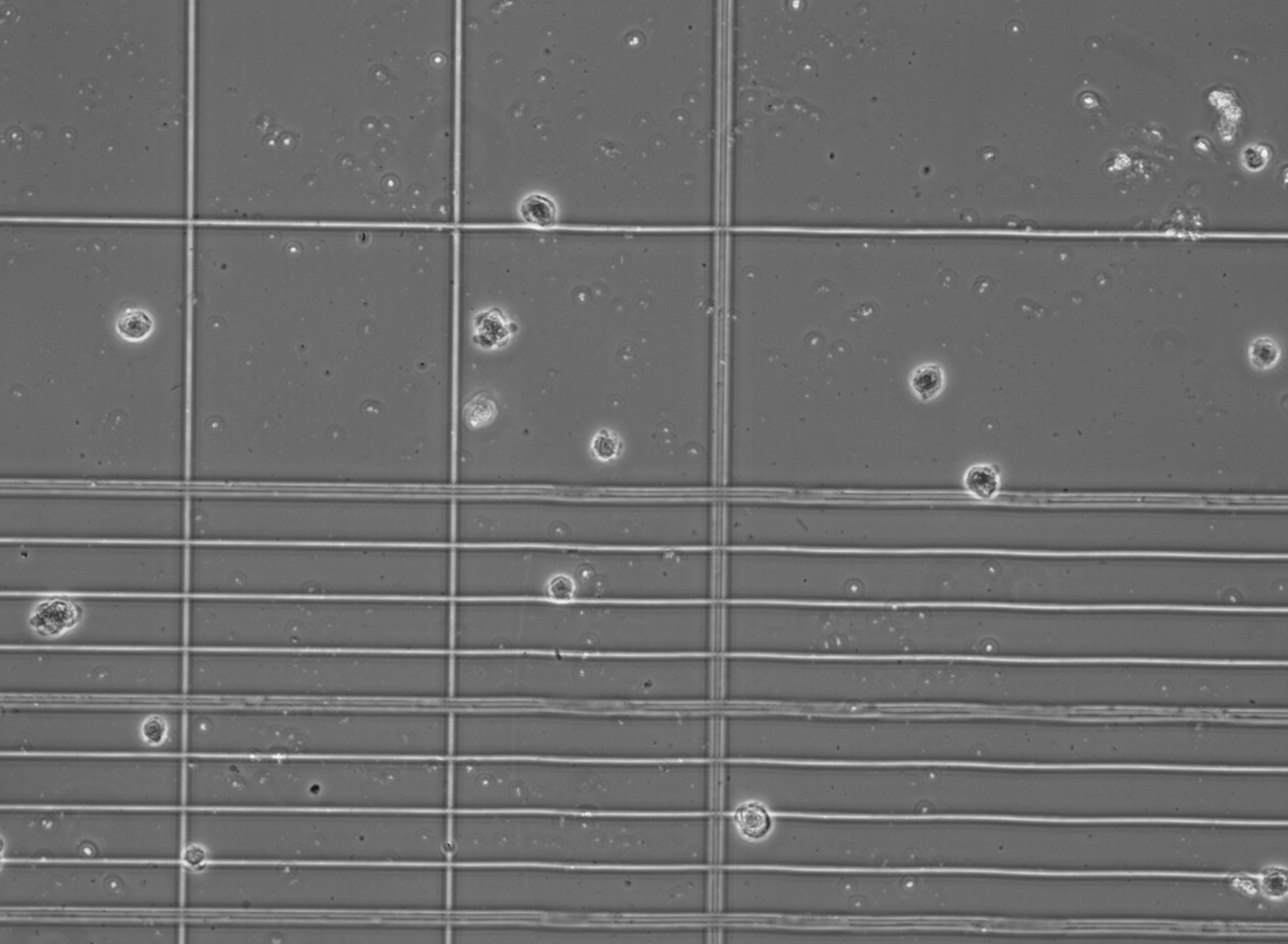

Supplement: S4 File — (ZIP) [file pone.0329484.s004.zip › S4 File - l-CSC 1/l-CSC 1/untitled038.tif]

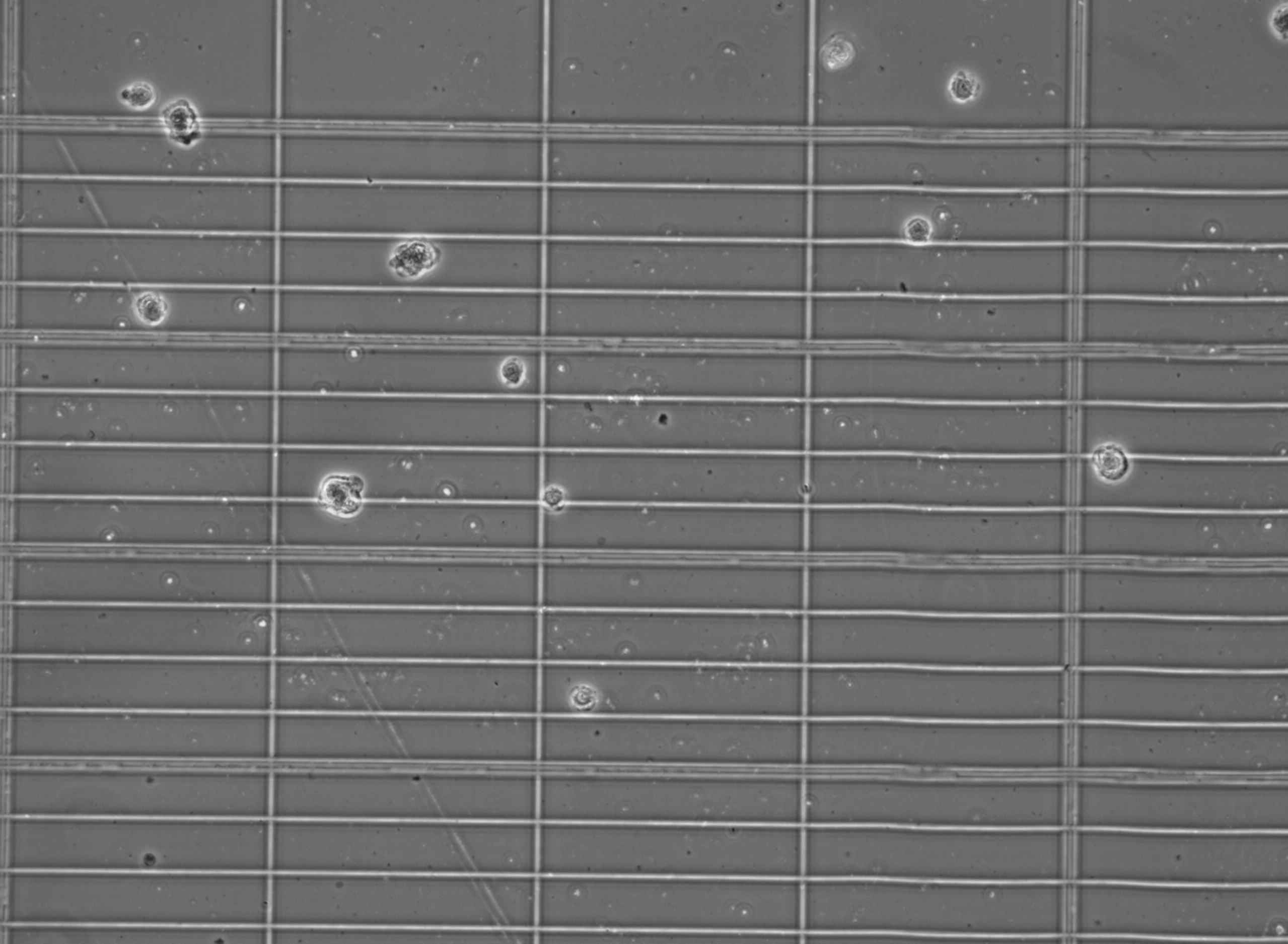

Supplement: S4 File — (ZIP) [file pone.0329484.s004.zip › S4 File - l-CSC 1/l-CSC 1/untitled039.tif]

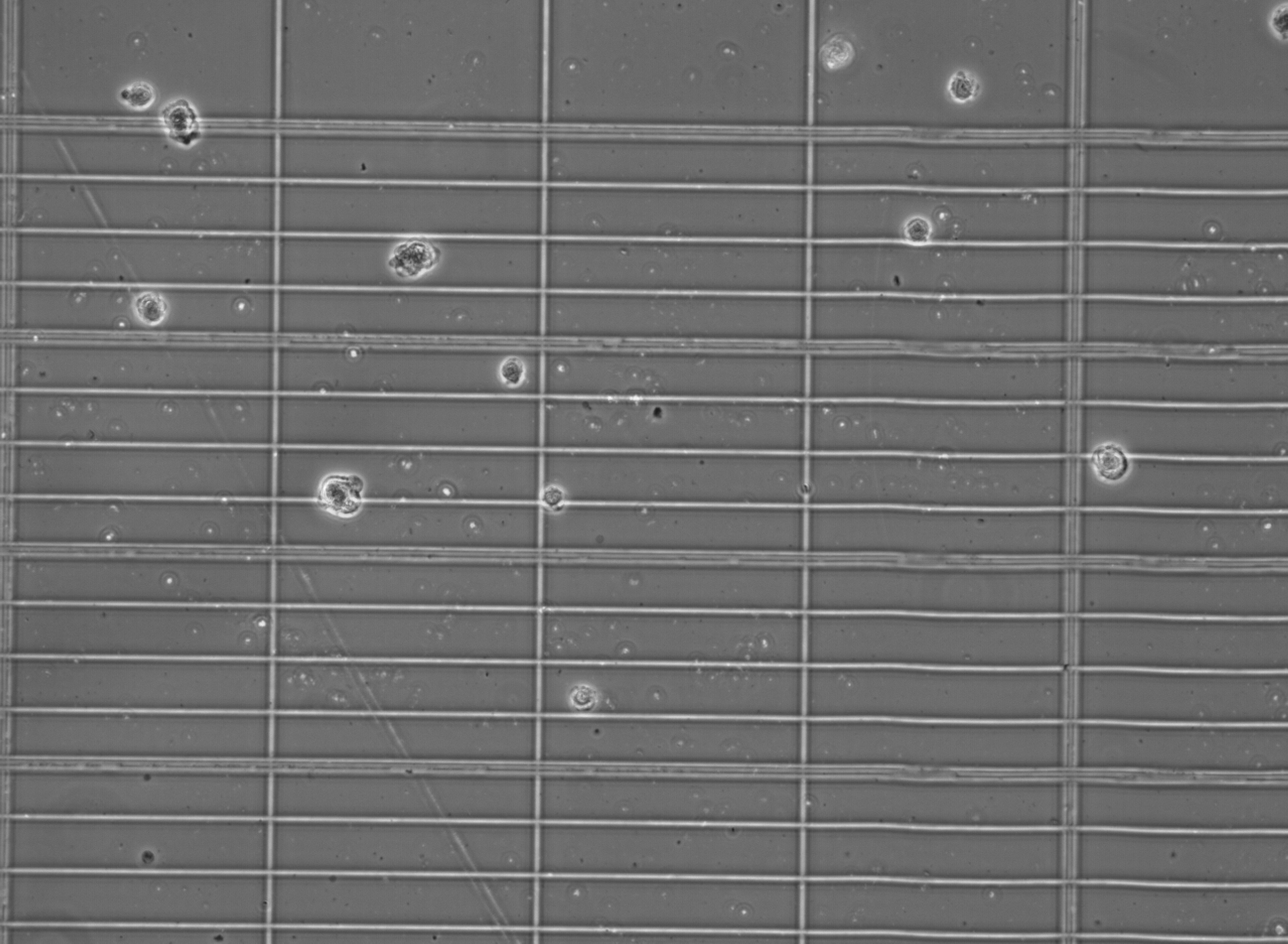

Supplement: S4 File — (ZIP) [file pone.0329484.s004.zip › S4 File - l-CSC 1/l-CSC 1/untitled040.tif]

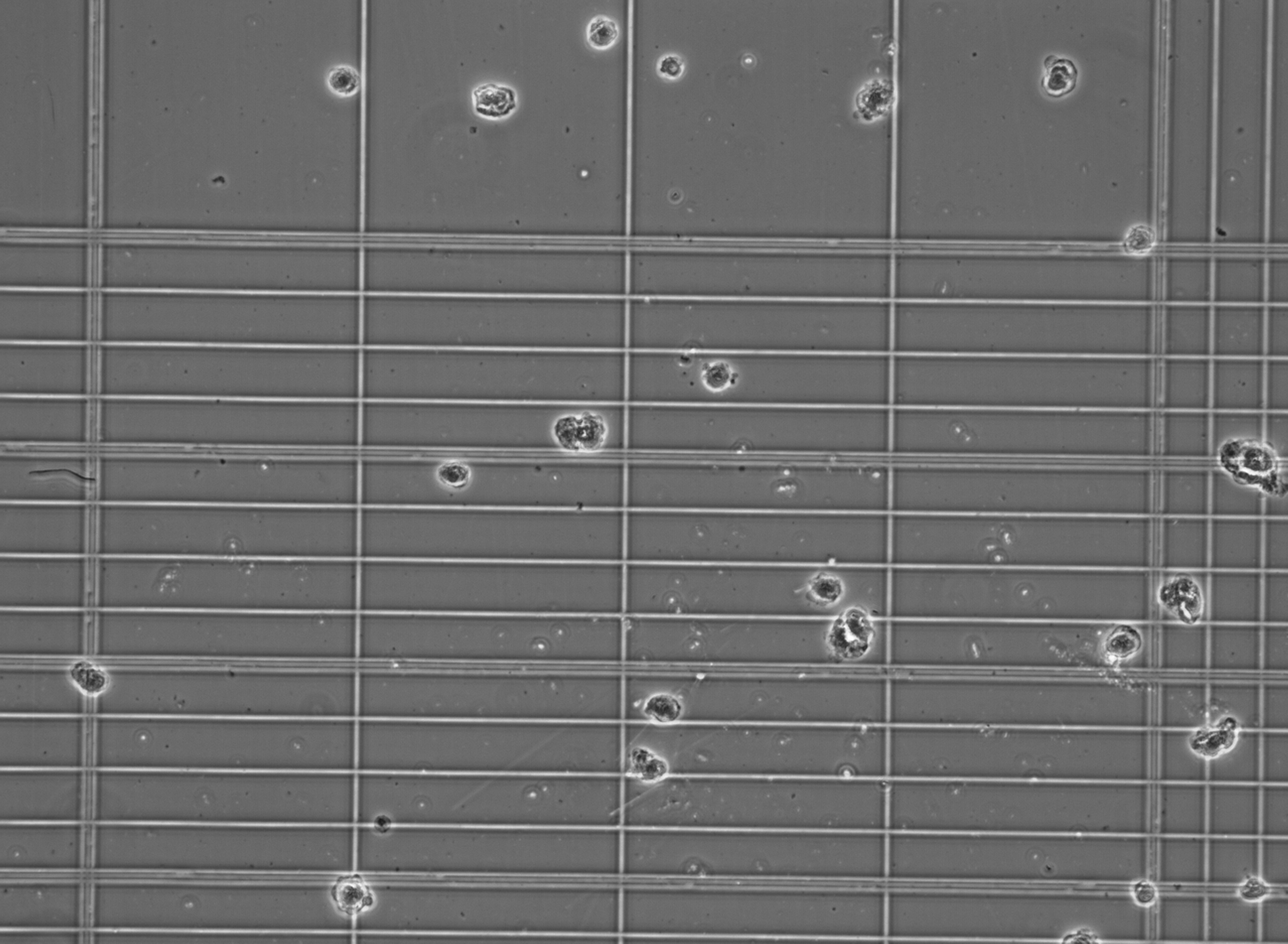

Supplement: S4 File — (ZIP) [file pone.0329484.s004.zip › S4 File - l-CSC 1/l-CSC 1/untitled041.tif]

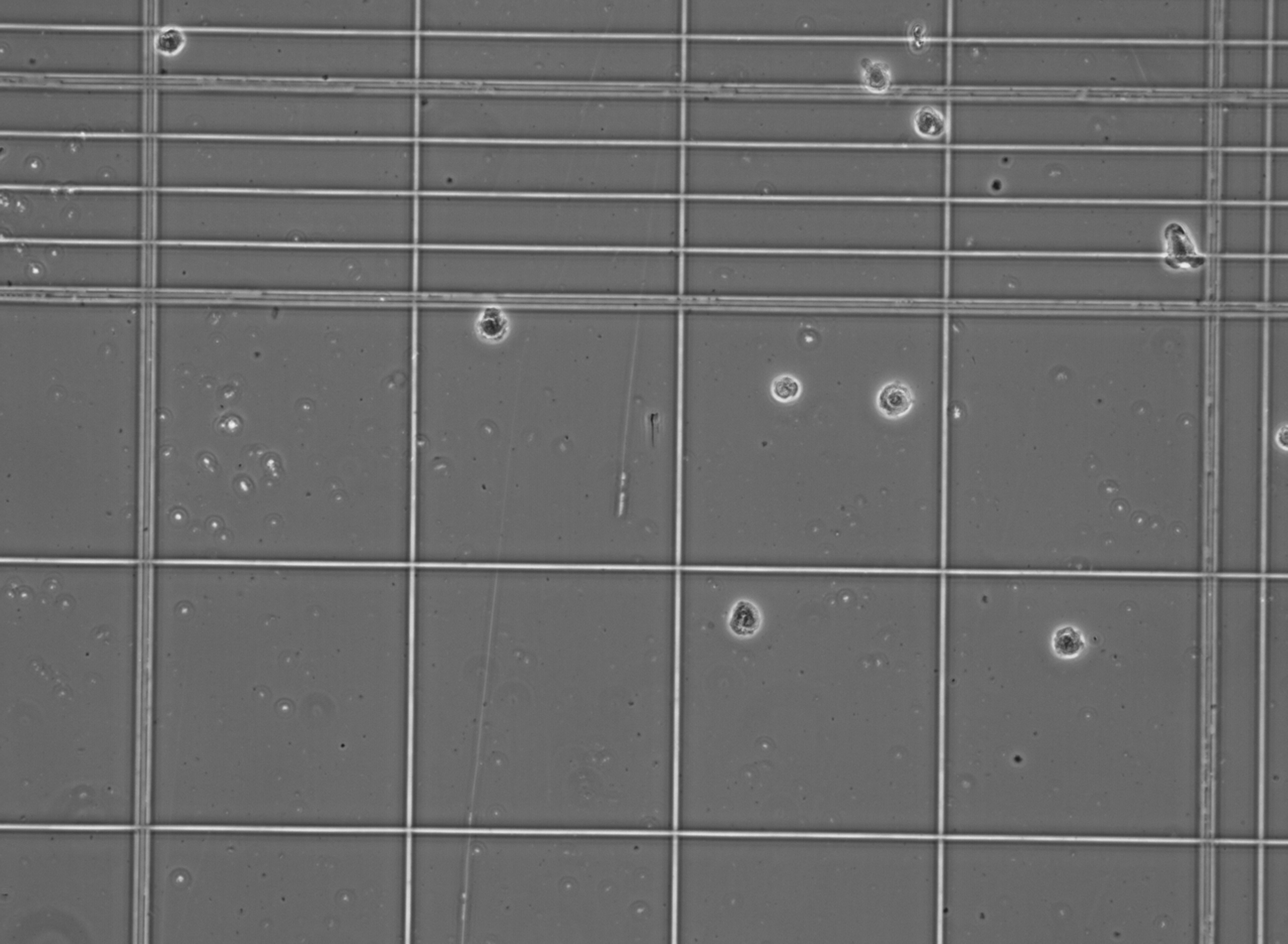

Supplement: S4 File — (ZIP) [file pone.0329484.s004.zip › S4 File - l-CSC 1/l-CSC 1/untitled042.tif]

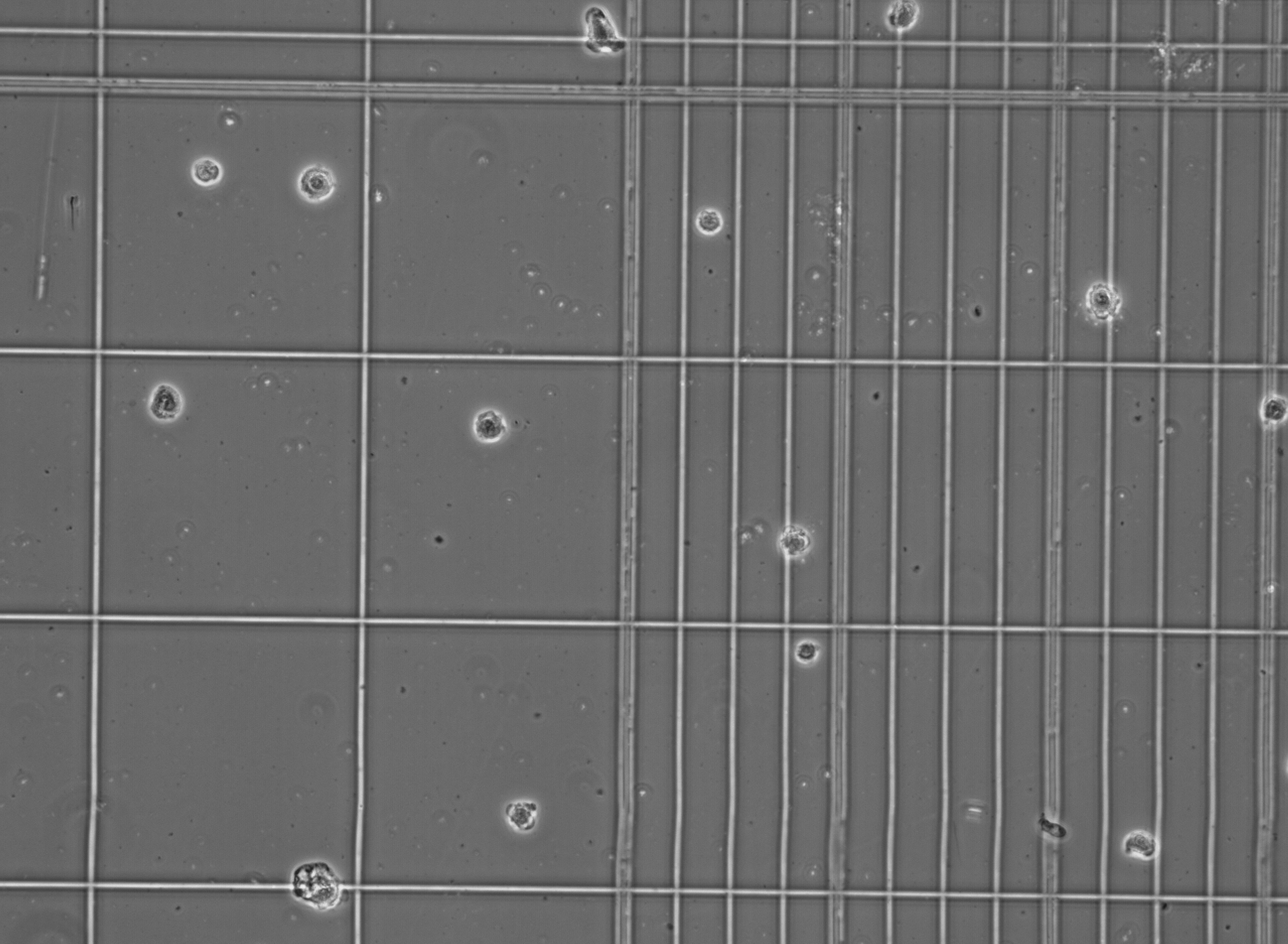

Supplement: S4 File — (ZIP) [file pone.0329484.s004.zip › S4 File - l-CSC 1/l-CSC 1/untitled043.tif]

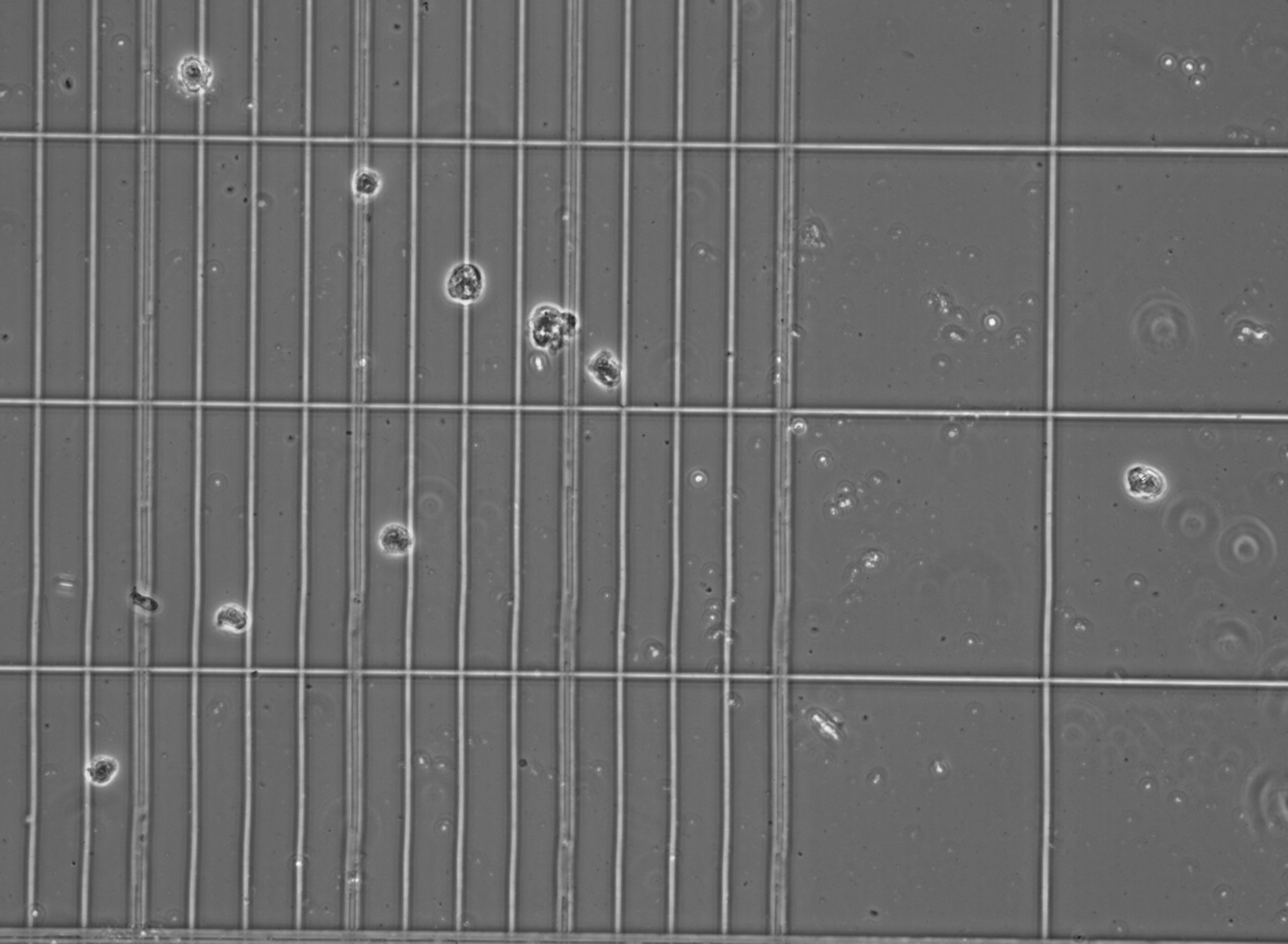

Supplement: S4 File — (ZIP) [file pone.0329484.s004.zip › S4 File - l-CSC 1/l-CSC 1/untitled044.tif]

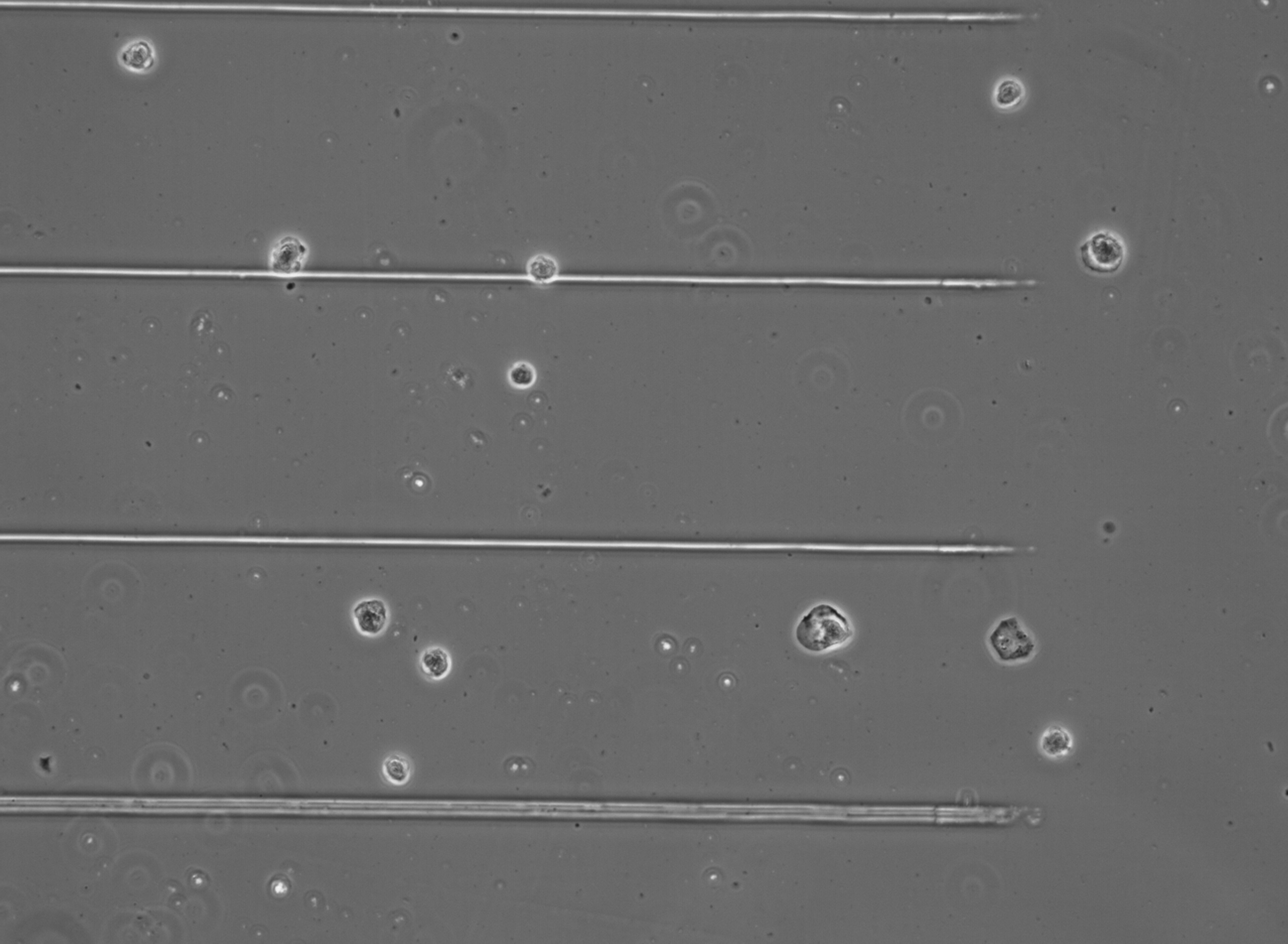

Supplement: S4 File — (ZIP) [file pone.0329484.s004.zip › S4 File - l-CSC 1/l-CSC 1/untitled045.tif]

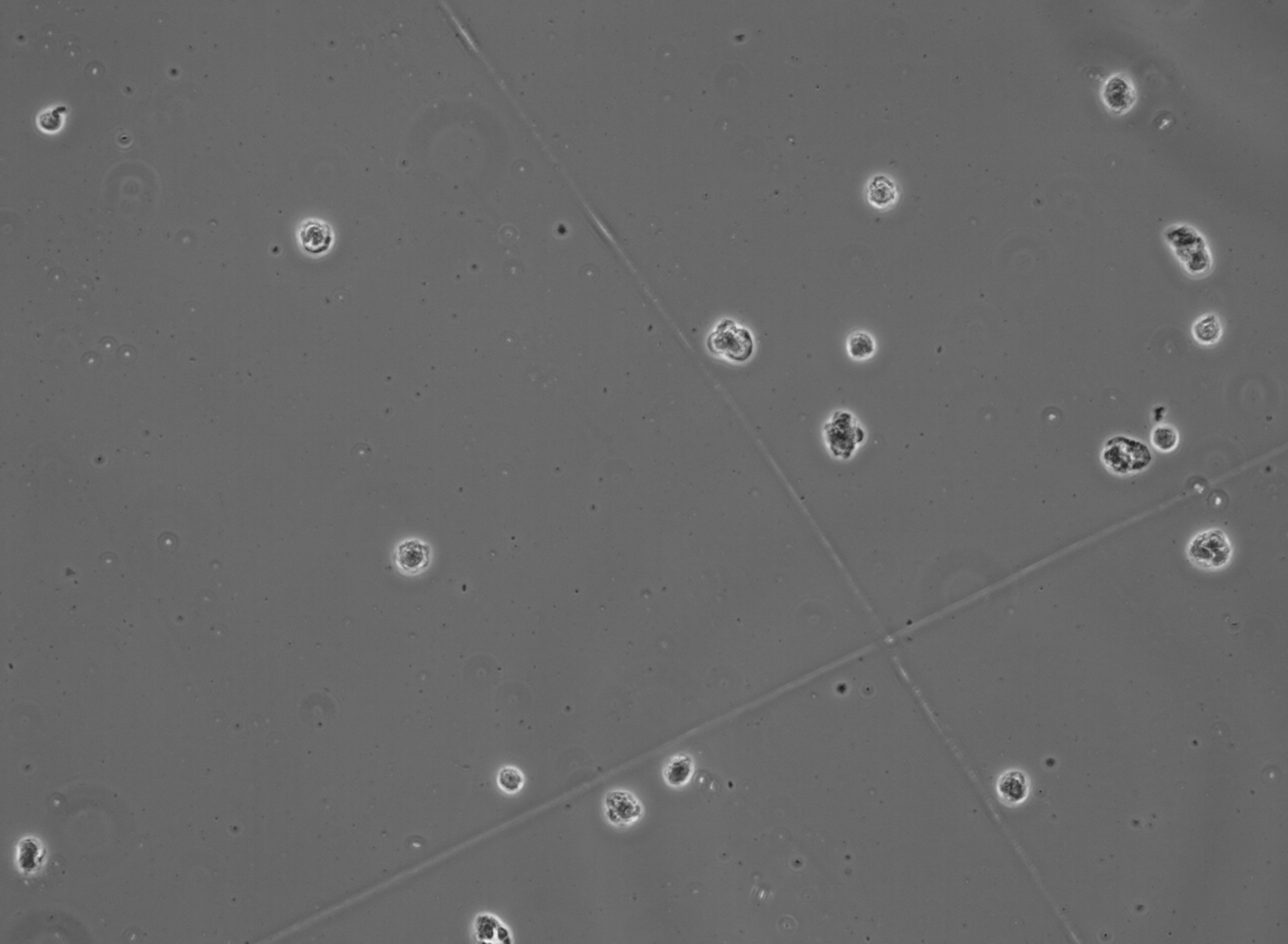

Supplement: S4 File — (ZIP) [file pone.0329484.s004.zip › S4 File - l-CSC 1/l-CSC 1/untitled046.tif]

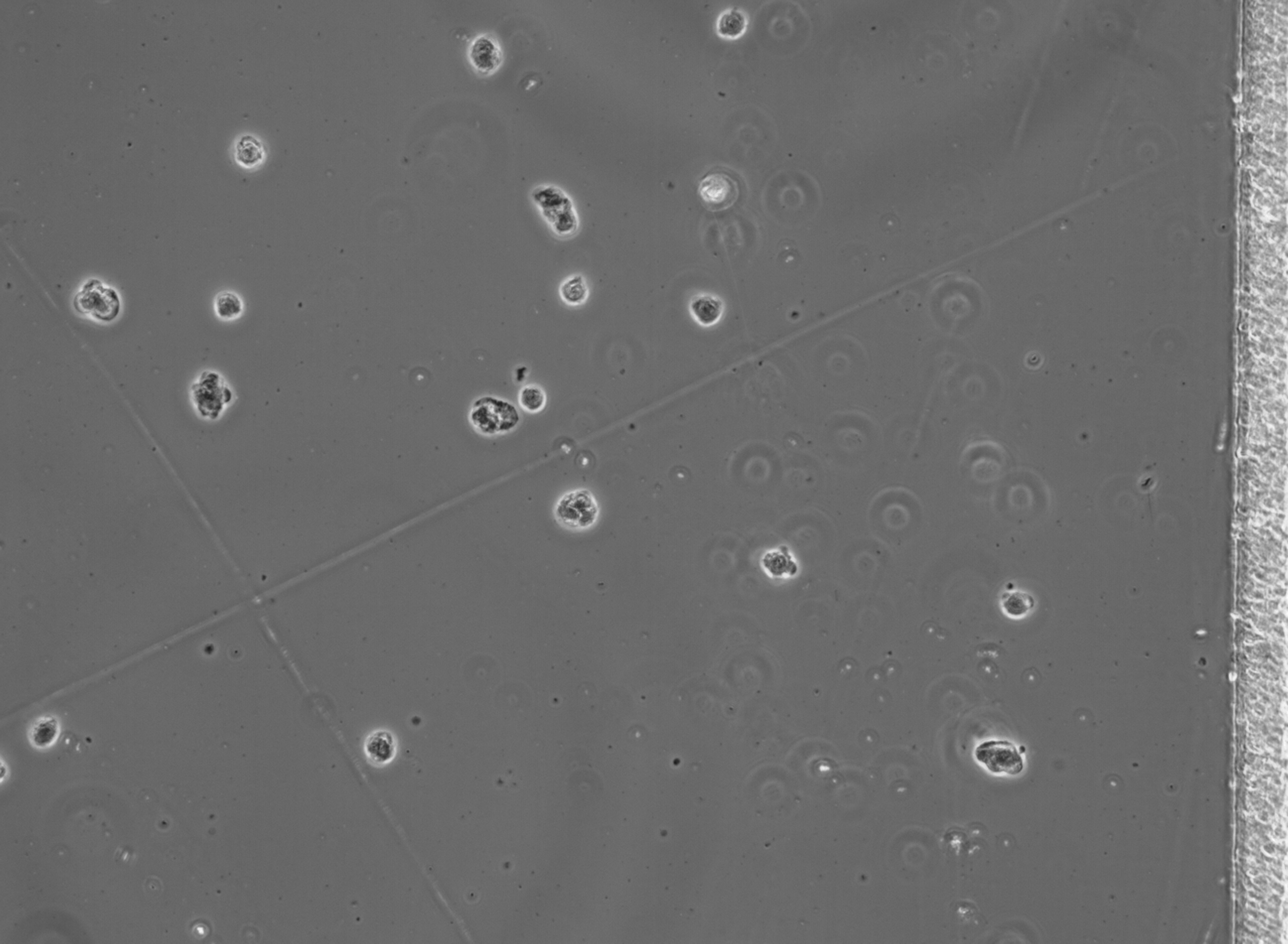

Supplement: S4 File — (ZIP) [file pone.0329484.s004.zip › S4 File - l-CSC 1/l-CSC 1/untitled047.tif]

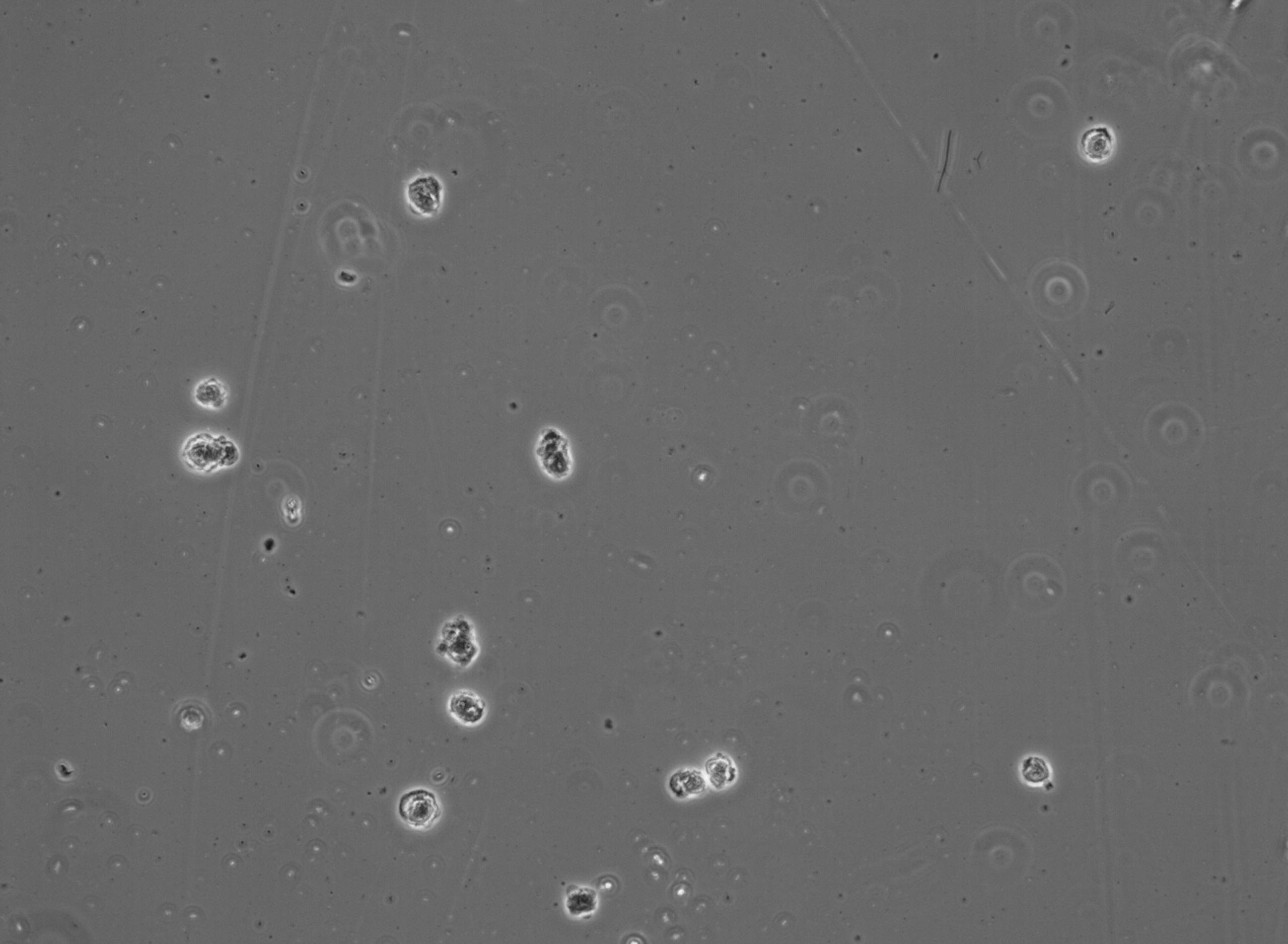

Supplement: S4 File — (ZIP) [file pone.0329484.s004.zip › S4 File - l-CSC 1/l-CSC 1/untitled048.tif]

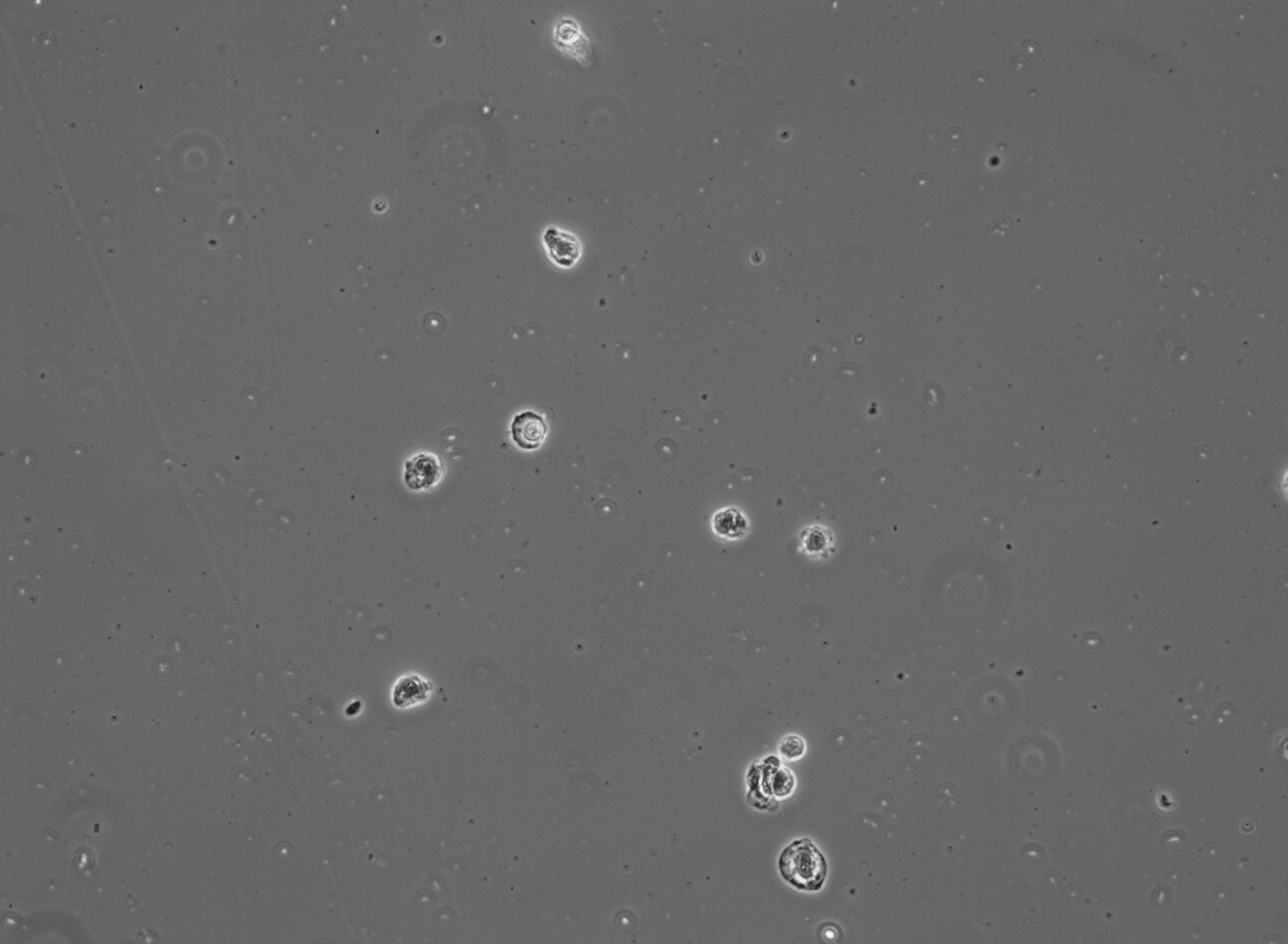

Supplement: S4 File — (ZIP) [file pone.0329484.s004.zip › S4 File - l-CSC 1/l-CSC 1/untitled049.tif]

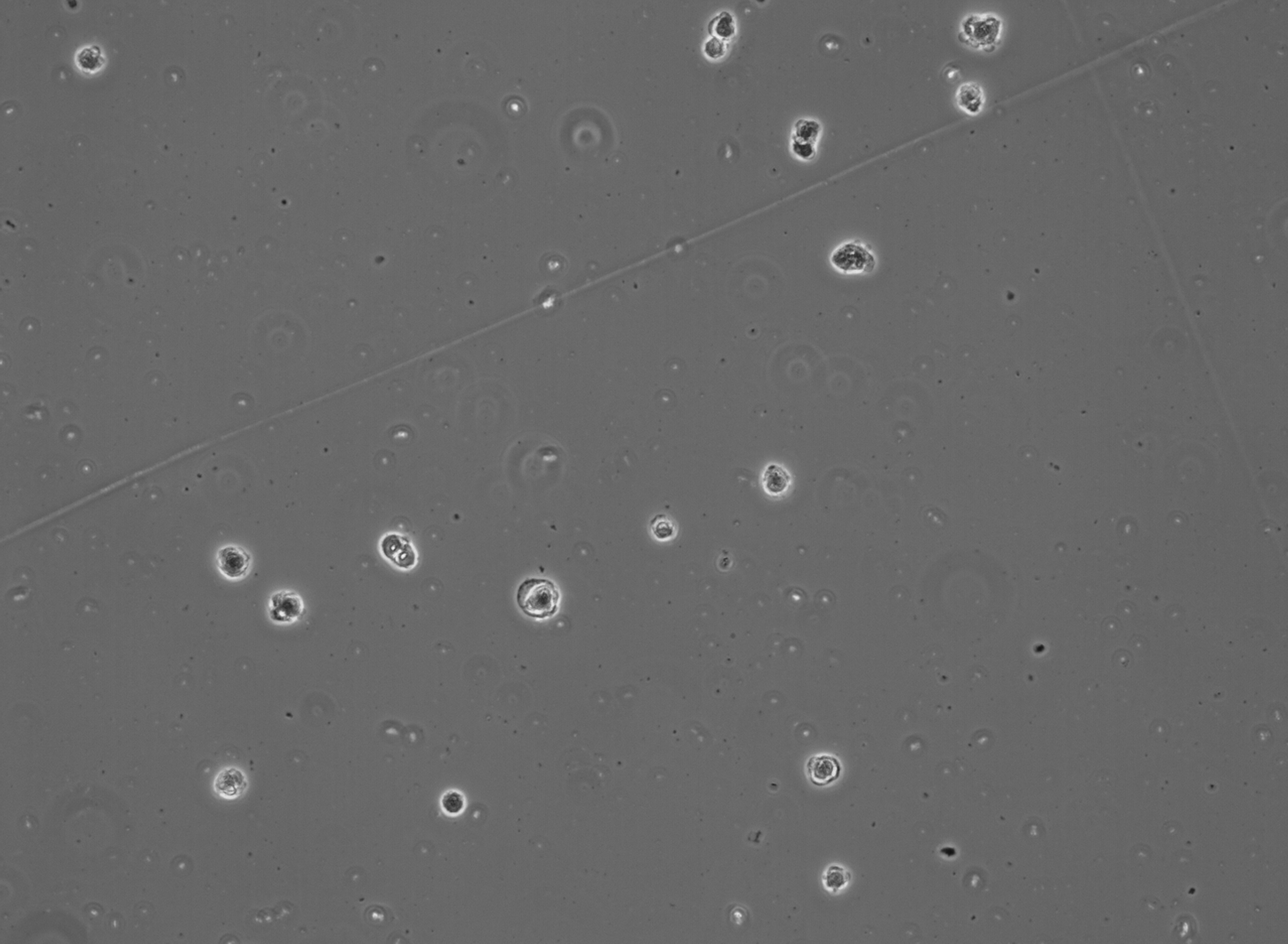

Supplement: S4 File — (ZIP) [file pone.0329484.s004.zip › S4 File - l-CSC 1/l-CSC 1/untitled050.tif]

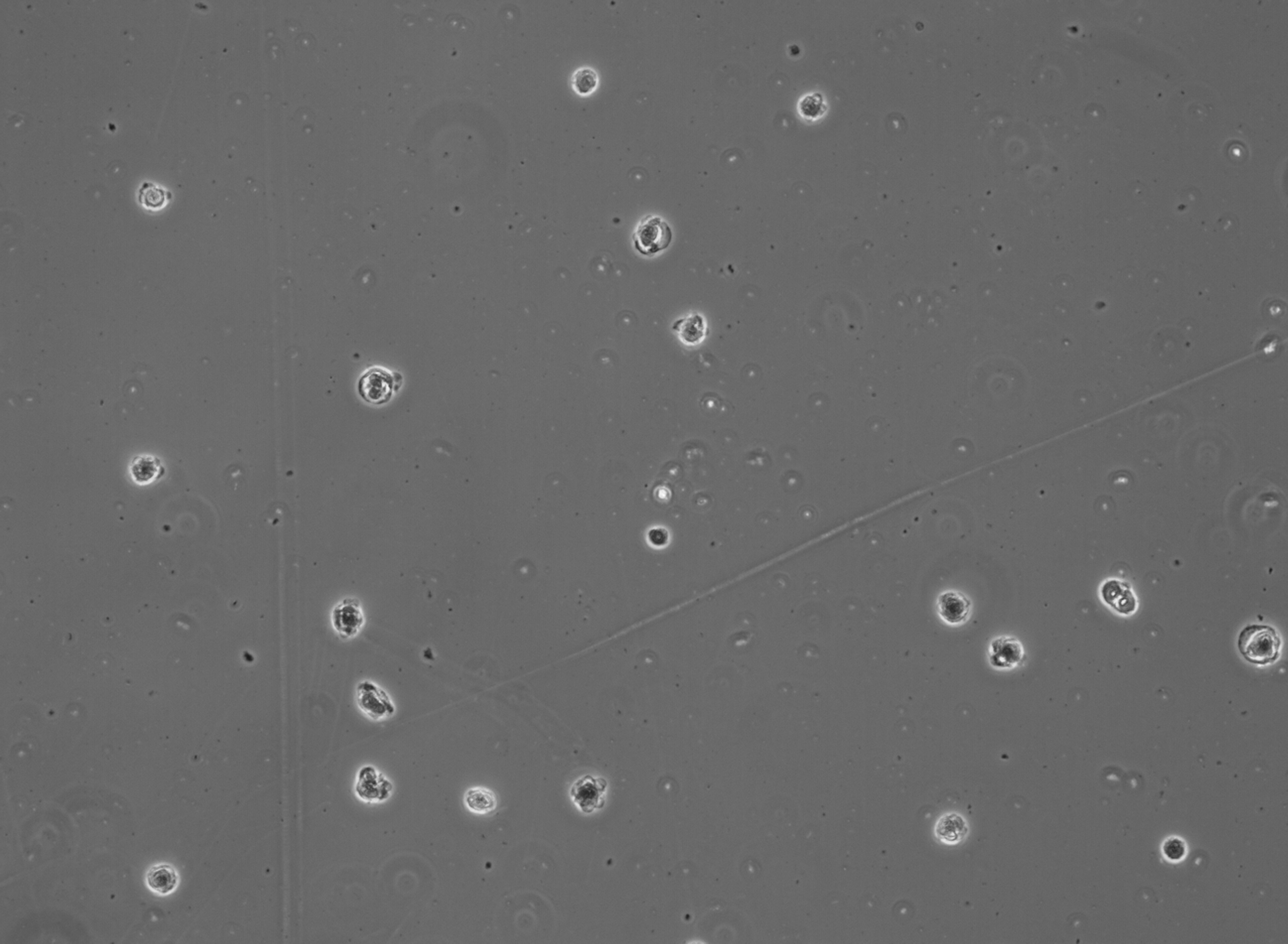

Supplement: S4 File — (ZIP) [file pone.0329484.s004.zip › S4 File - l-CSC 1/l-CSC 1/untitled051.tif]

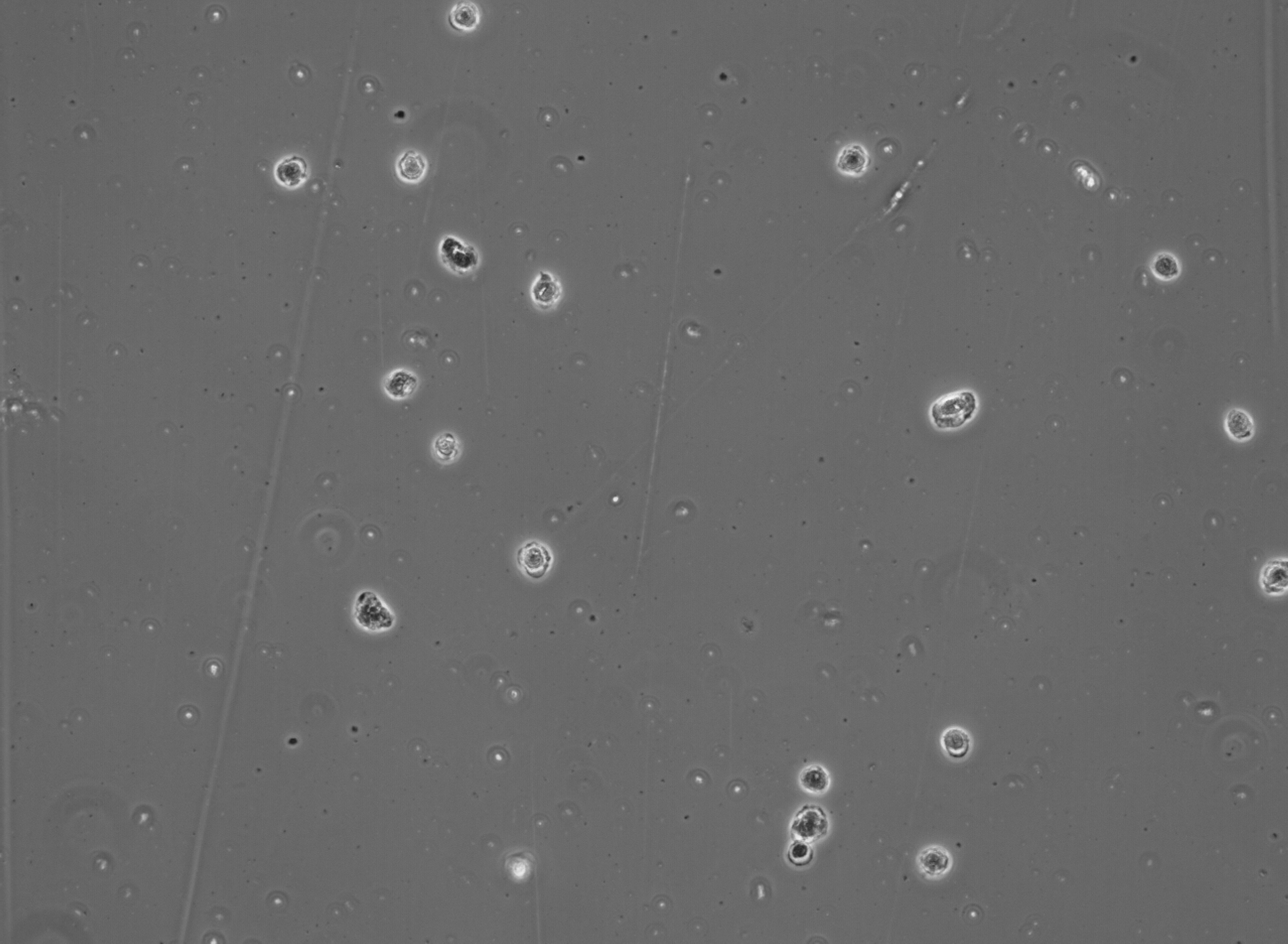

Supplement: S4 File — (ZIP) [file pone.0329484.s004.zip › S4 File - l-CSC 1/l-CSC 1/untitled052.tif]

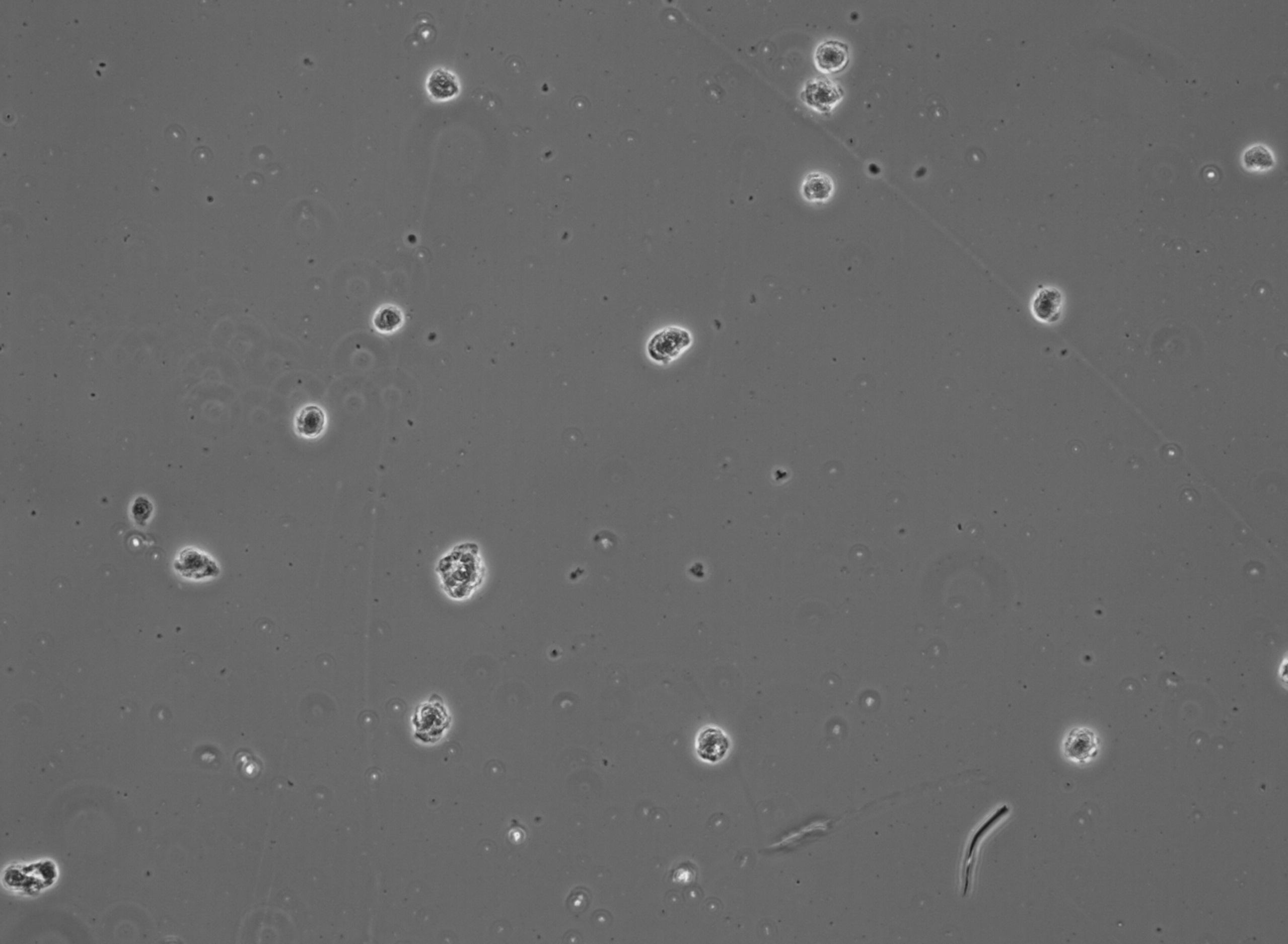

Supplement: S4 File — (ZIP) [file pone.0329484.s004.zip › S4 File - l-CSC 1/l-CSC 1/untitled053.tif]

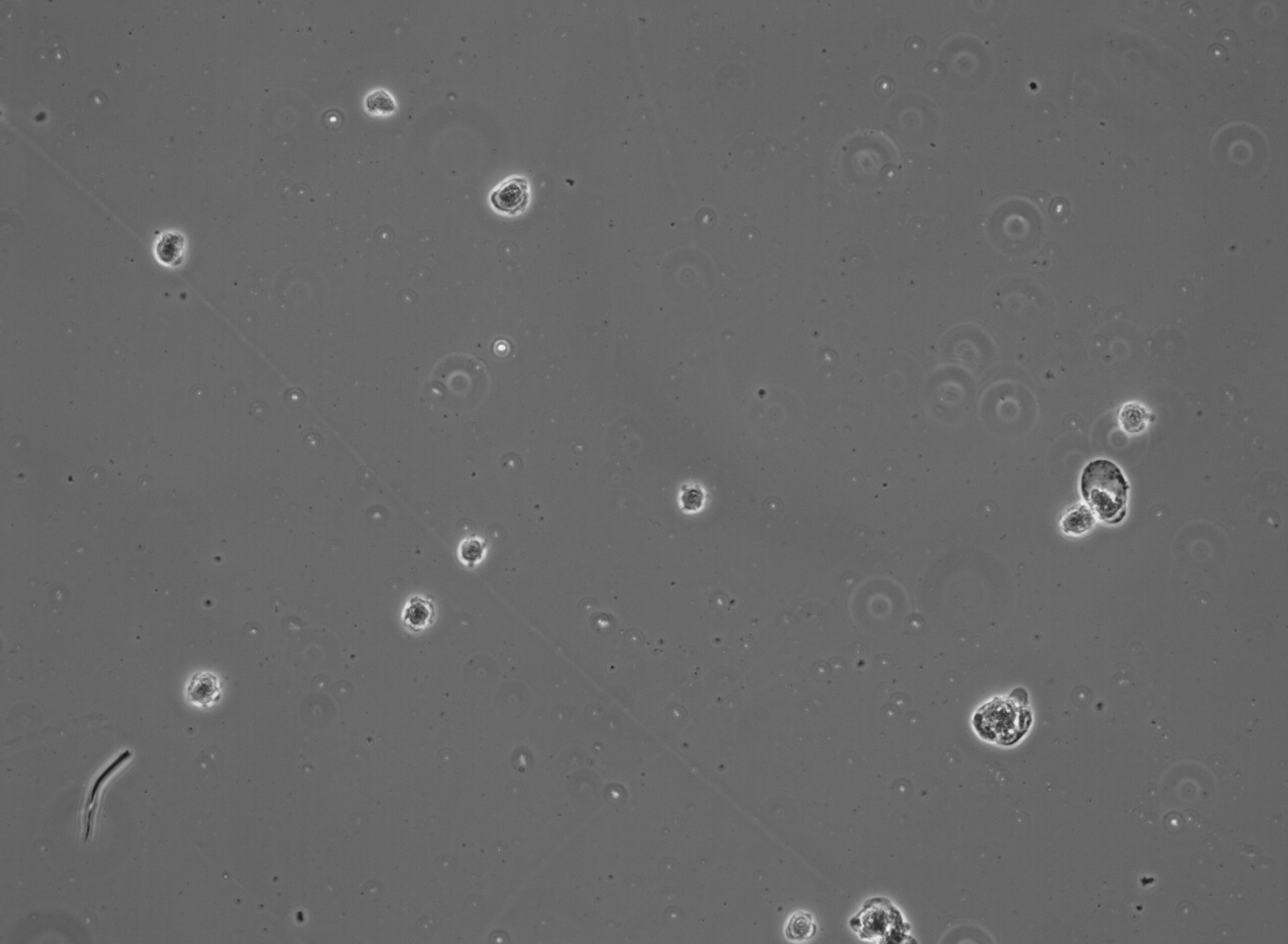

Supplement: S4 File — (ZIP) [file pone.0329484.s004.zip › S4 File - l-CSC 1/l-CSC 1/untitled054.tif]

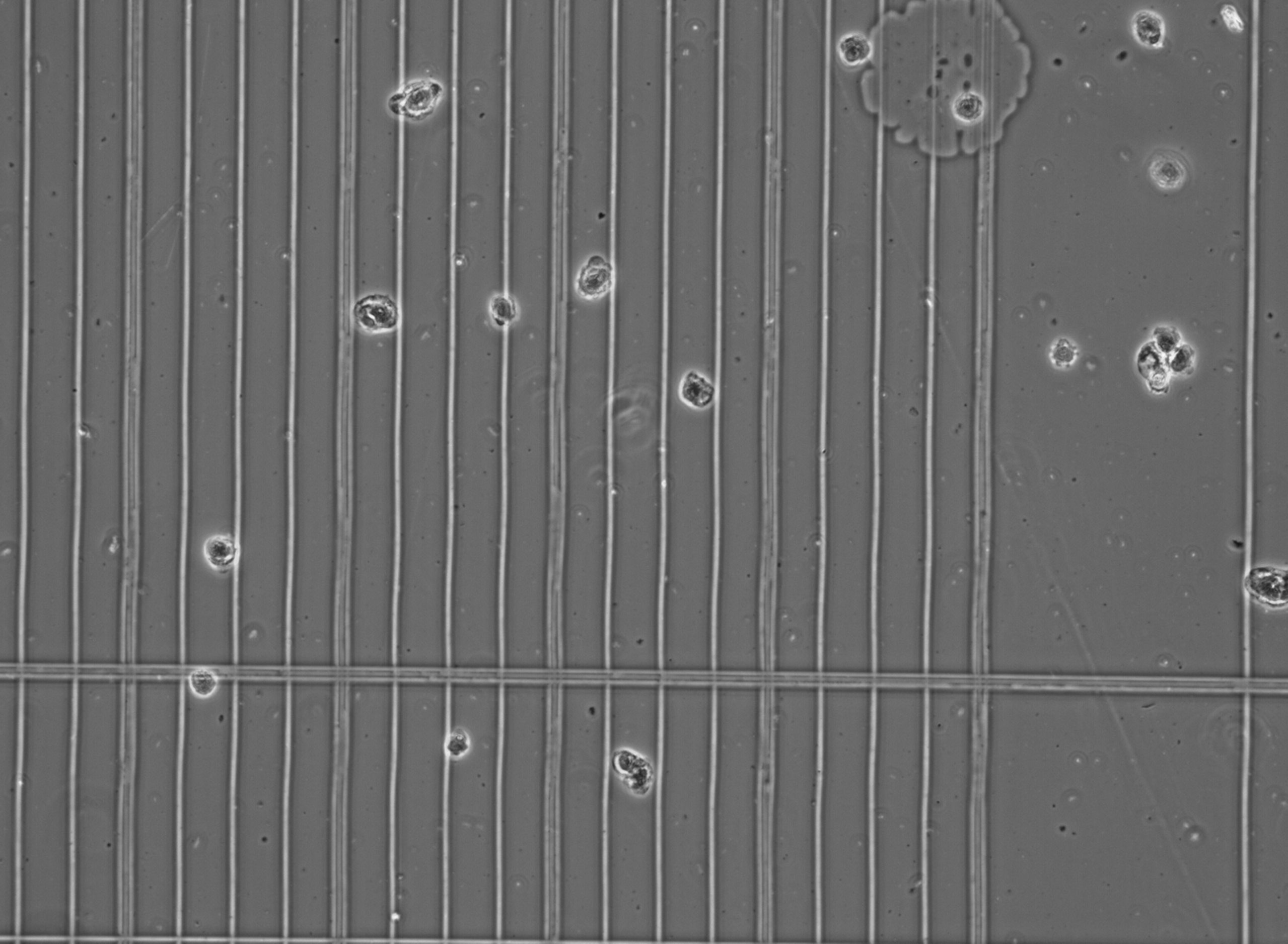

Supplement: S4 File — (ZIP) [file pone.0329484.s004.zip › S4 File - l-CSC 1/l-CSC 1/untitled055.tif]

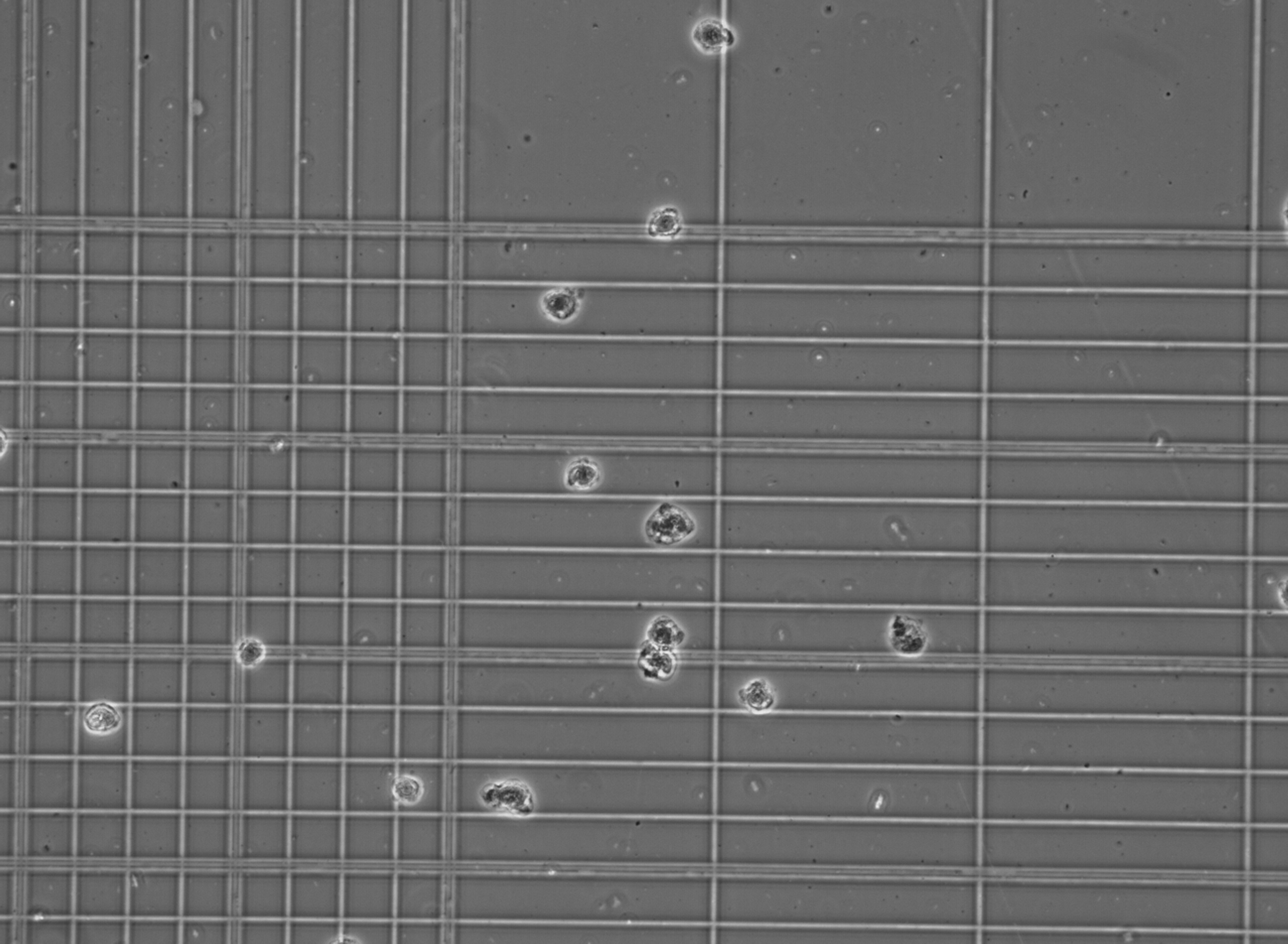

Supplement: S4 File — (ZIP) [file pone.0329484.s004.zip › S4 File - l-CSC 1/l-CSC 1/untitled056.tif]

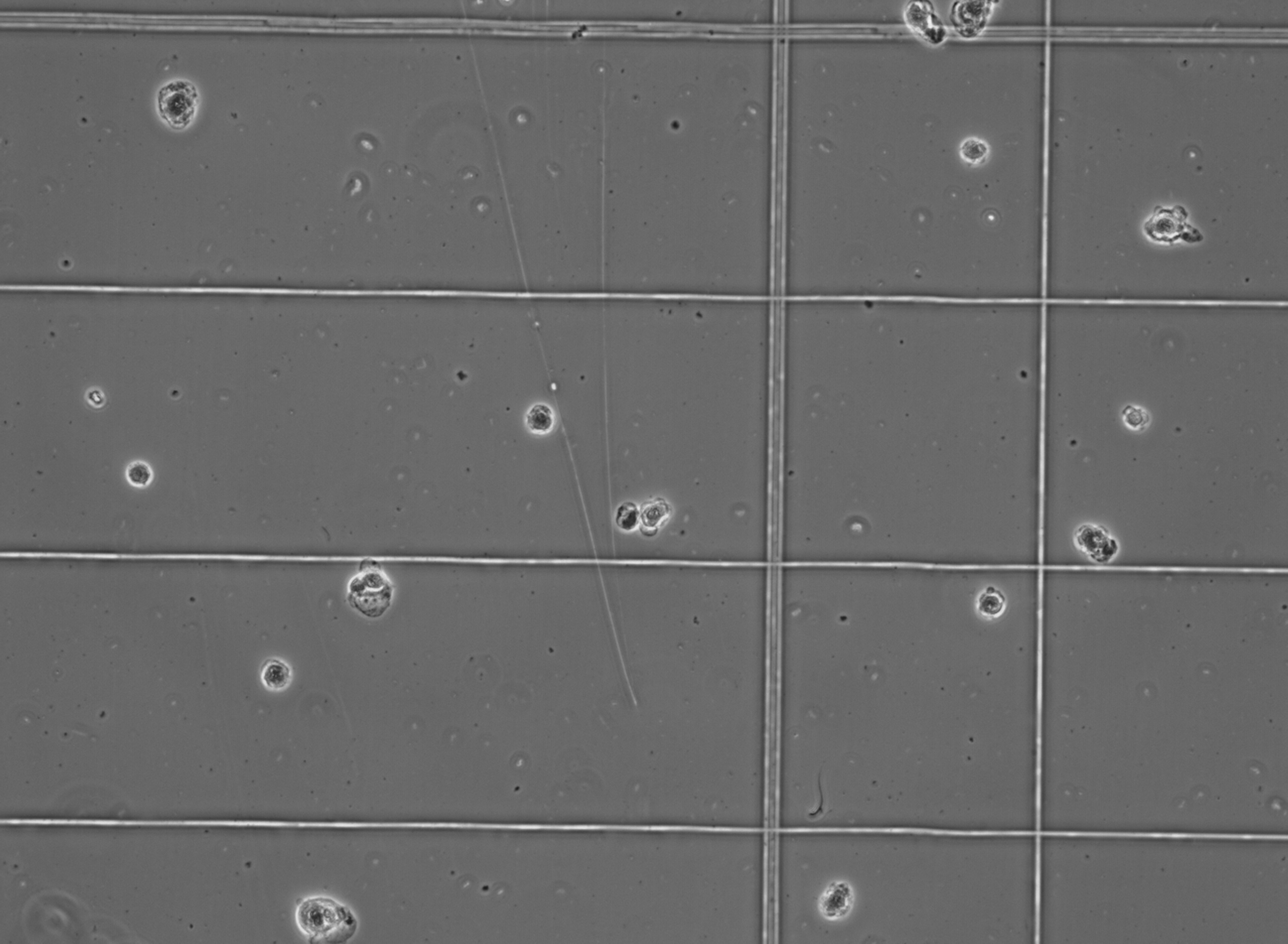

Supplement: S4 File — (ZIP) [file pone.0329484.s004.zip › S4 File - l-CSC 1/l-CSC 1/untitled057.tif]

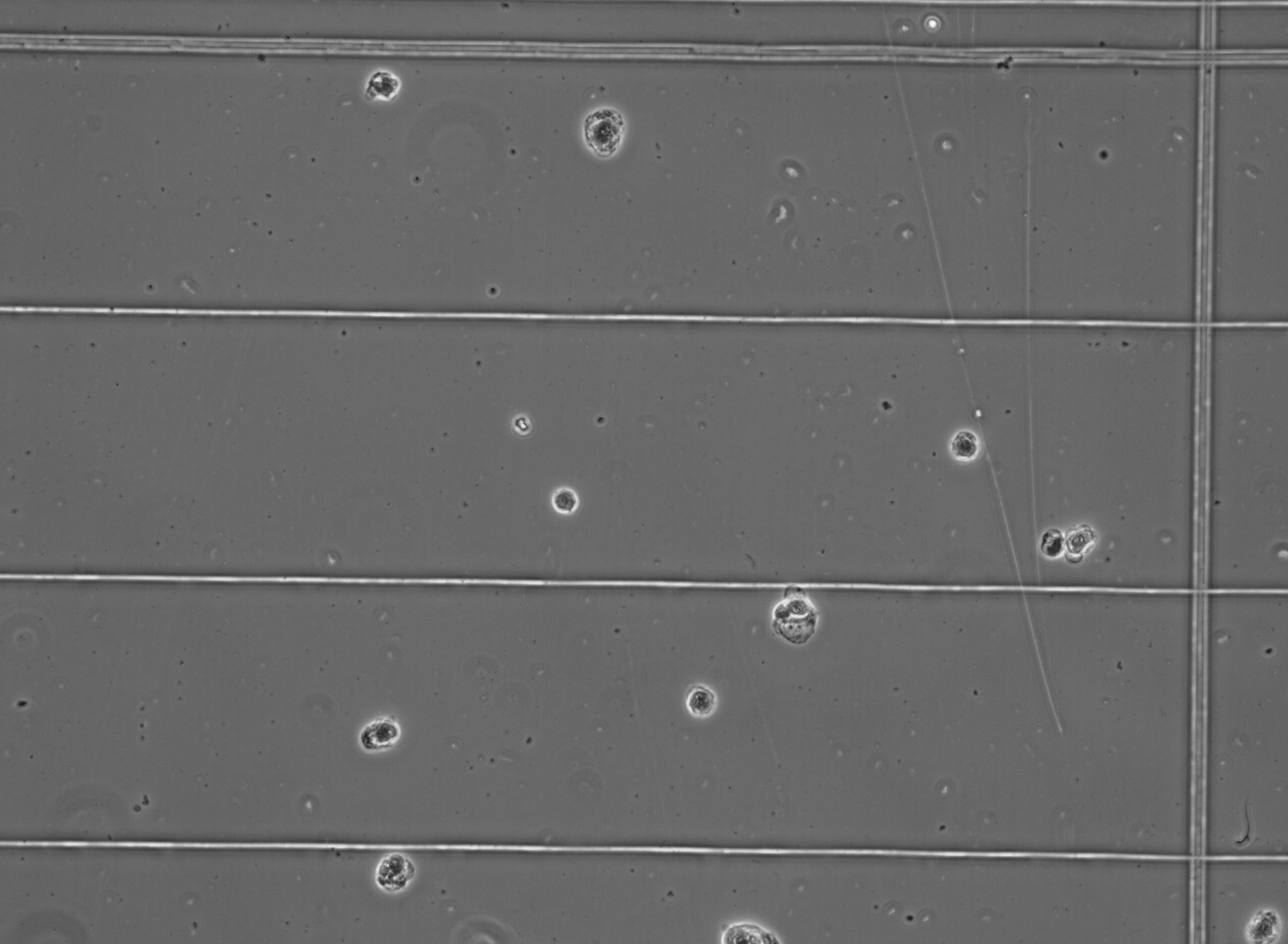

Supplement: S4 File — (ZIP) [file pone.0329484.s004.zip › S4 File - l-CSC 1/l-CSC 1/untitled058.tif]

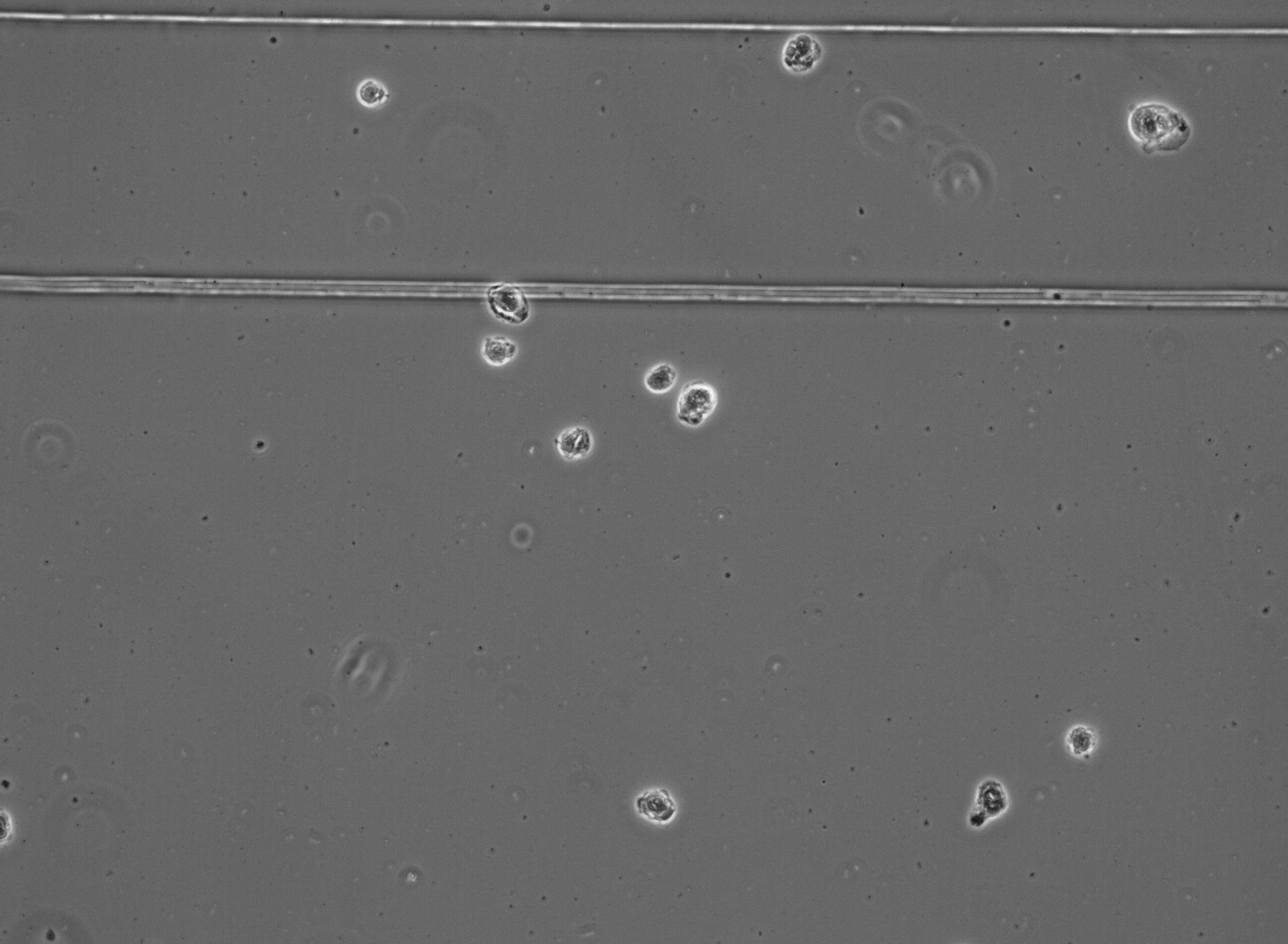

Supplement: S4 File — (ZIP) [file pone.0329484.s004.zip › S4 File - l-CSC 1/l-CSC 1/untitled059.tif]

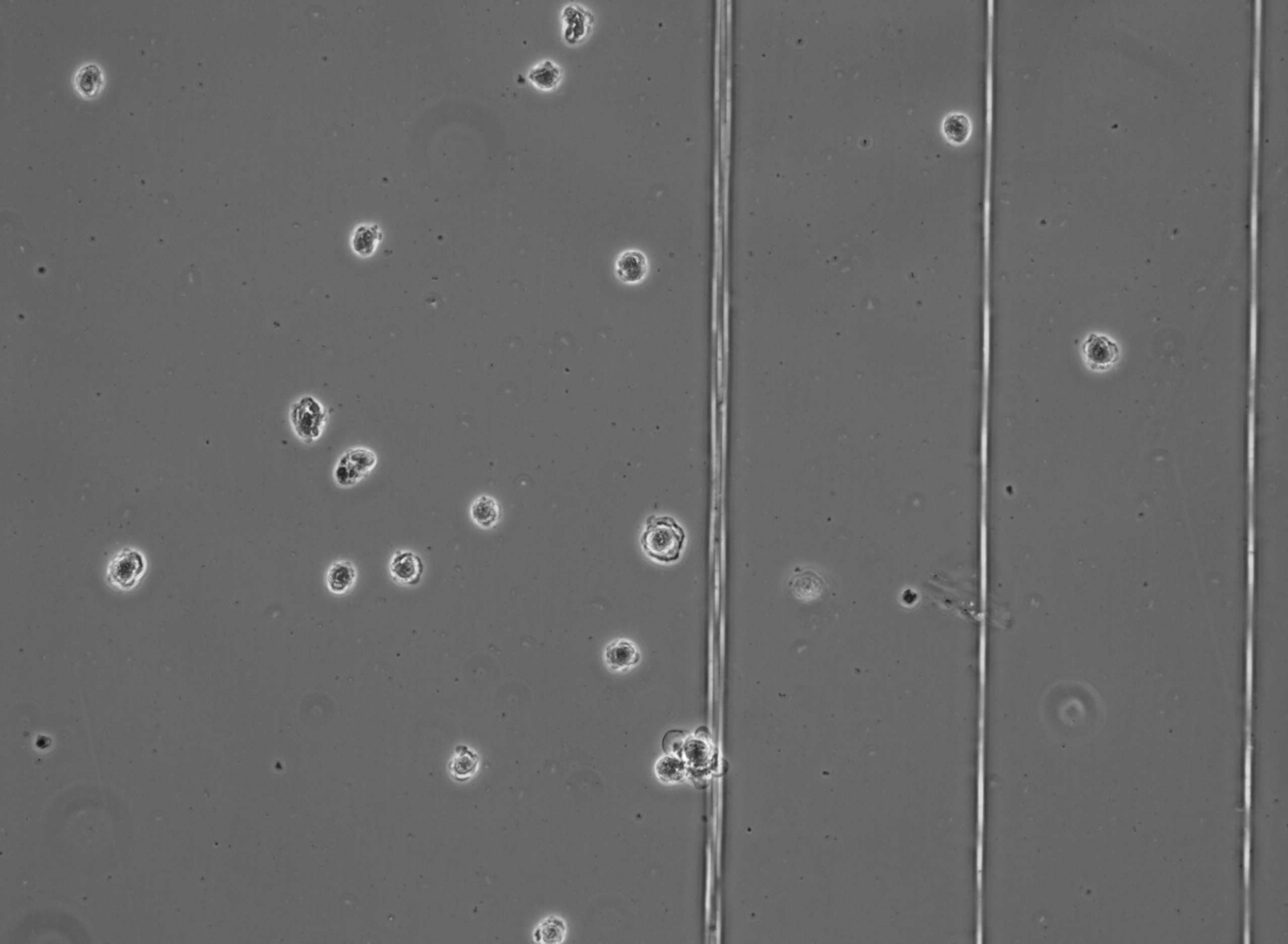

Supplement: S4 File — (ZIP) [file pone.0329484.s004.zip › S4 File - l-CSC 1/l-CSC 1/untitled060.tif]

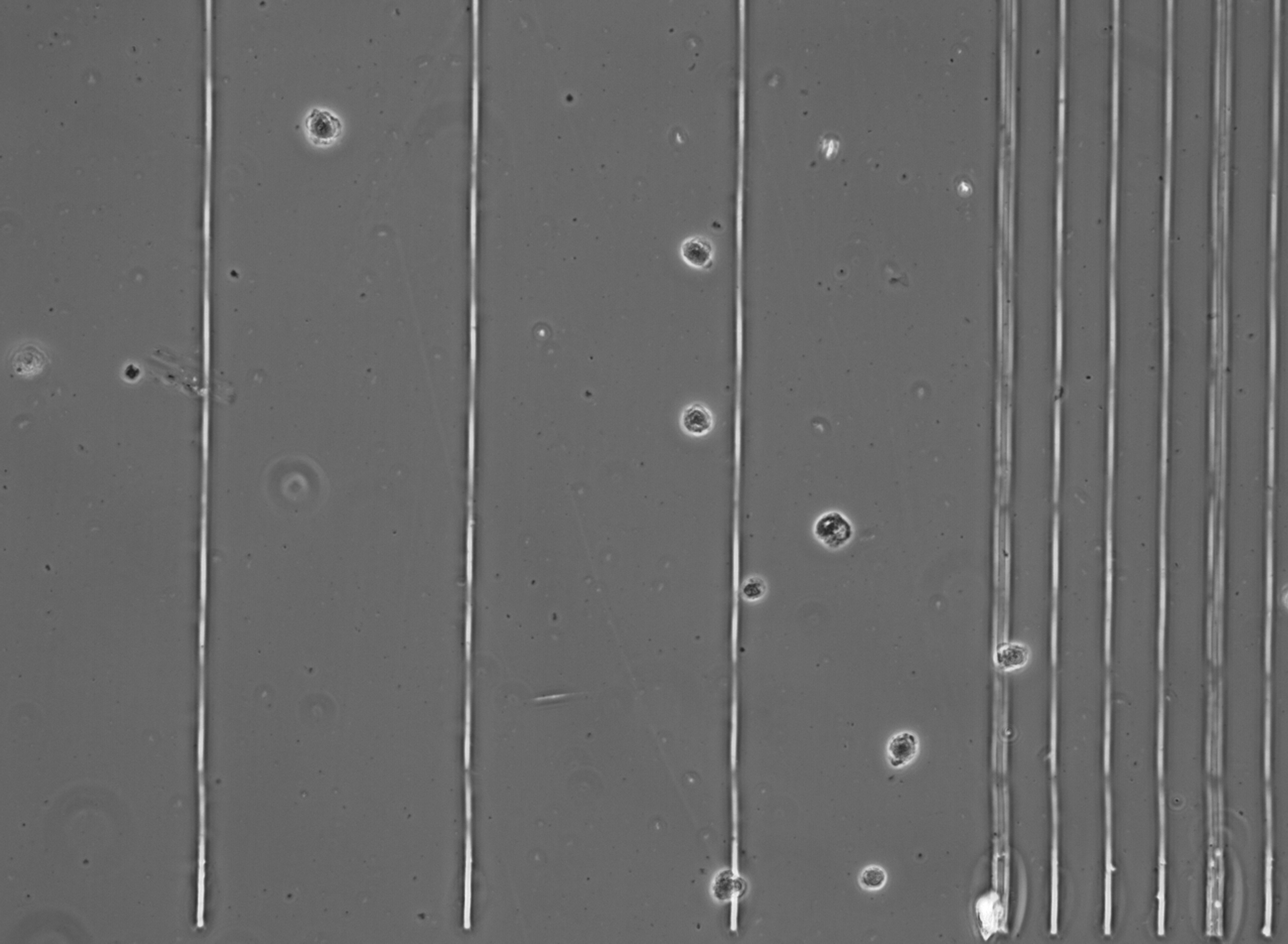

Supplement: S4 File — (ZIP) [file pone.0329484.s004.zip › S4 File - l-CSC 1/l-CSC 1/untitled061.tif]

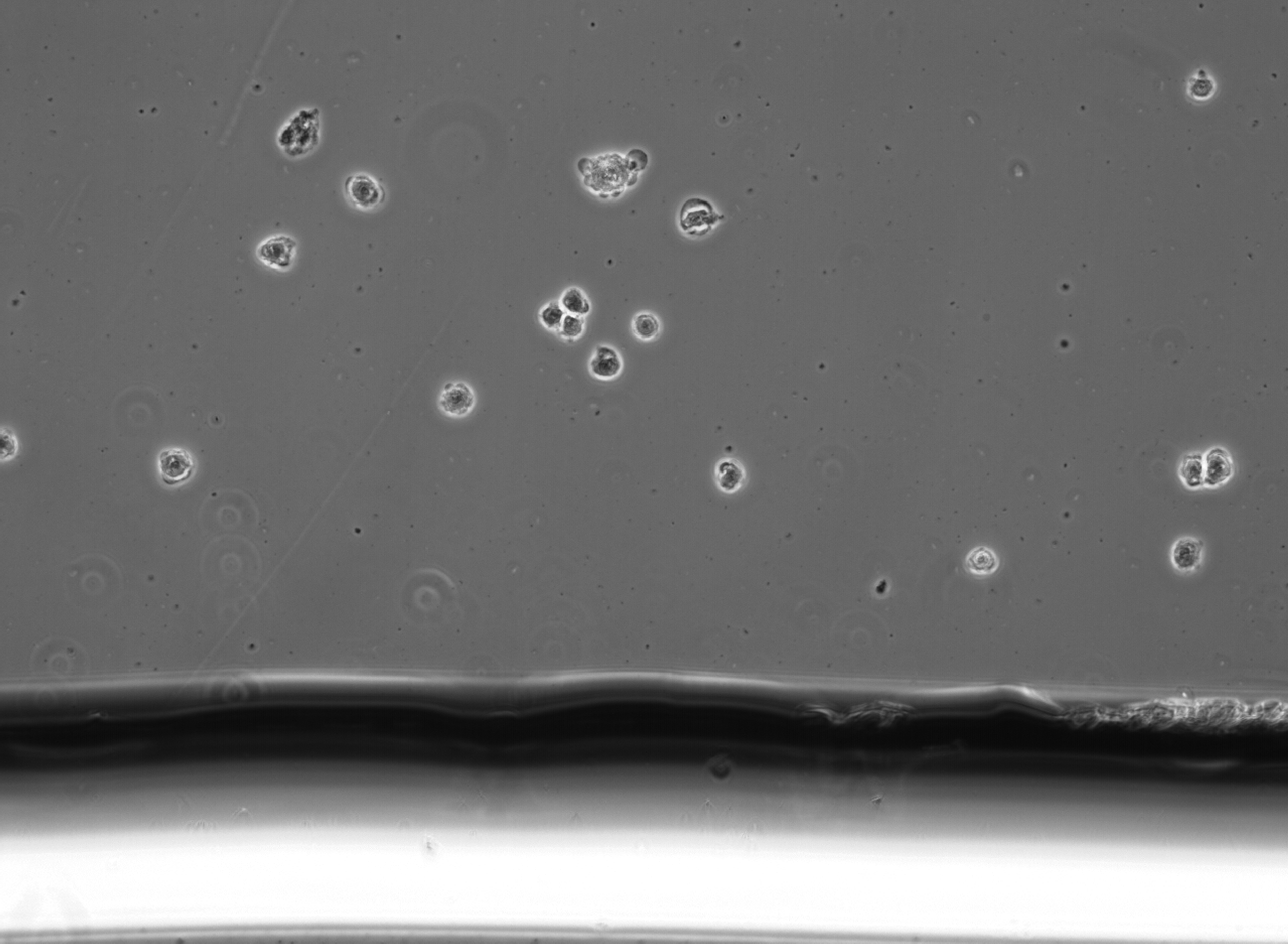

Supplement: S4 File — (ZIP) [file pone.0329484.s004.zip › S4 File - l-CSC 1/l-CSC 1/untitled062.tif]

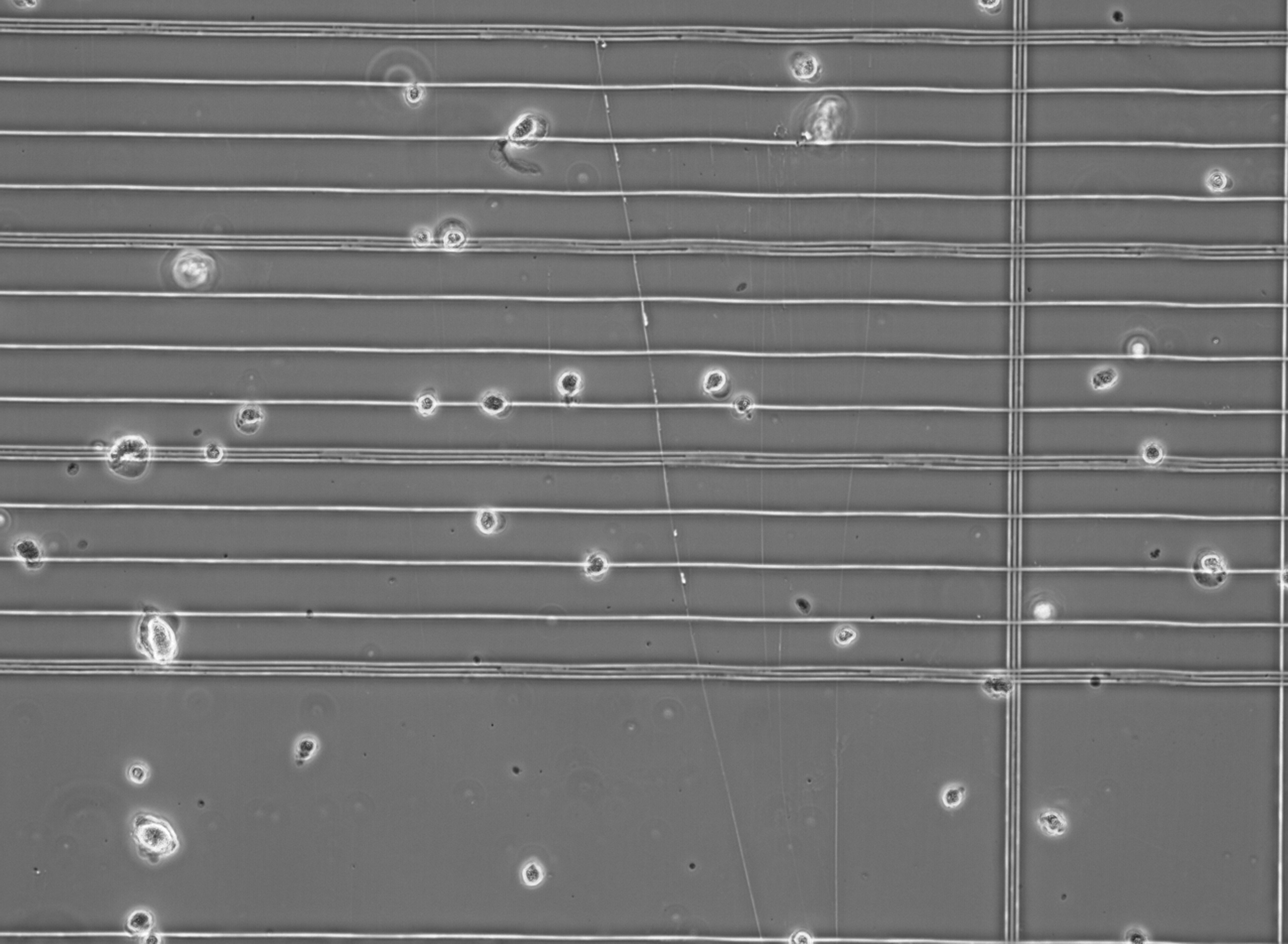

Supplement: S5 File — (ZIP) [file pone.0329484.s005.zip › S5 File - l-CSC 2/l-CSC 2/untitled063.tif]

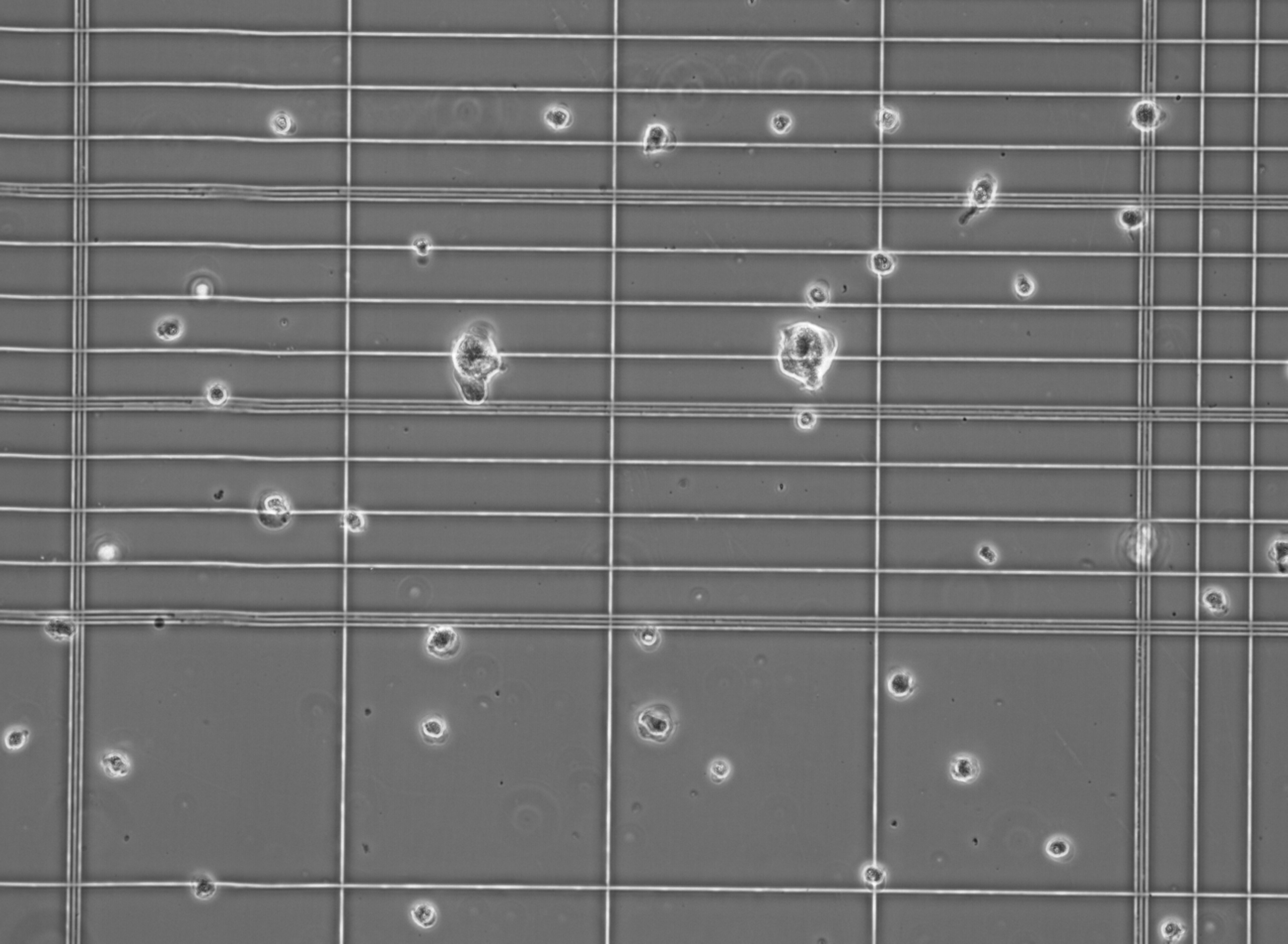

Supplement: S5 File — (ZIP) [file pone.0329484.s005.zip › S5 File - l-CSC 2/l-CSC 2/untitled064.tif]

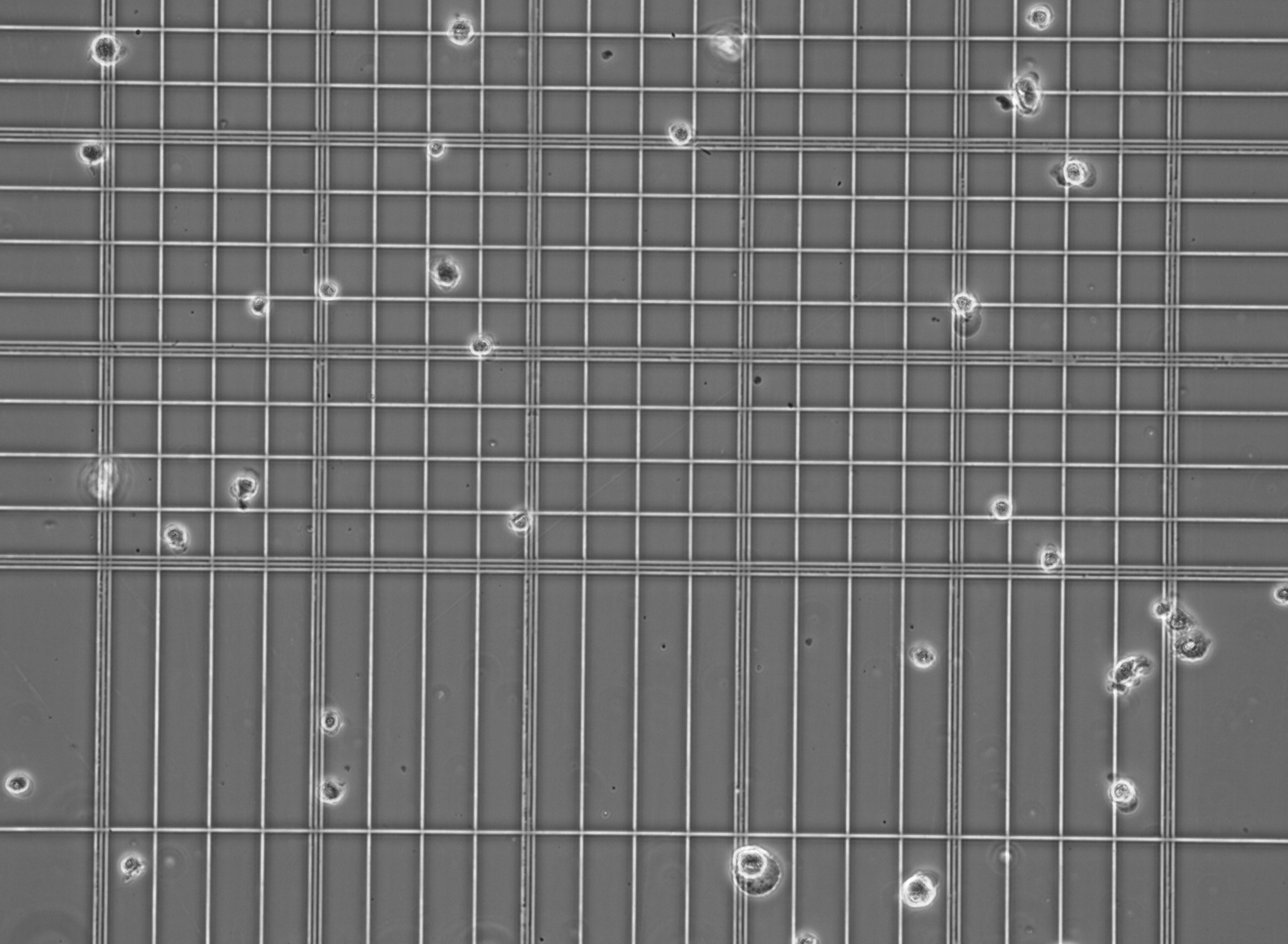

Supplement: S5 File — (ZIP) [file pone.0329484.s005.zip › S5 File - l-CSC 2/l-CSC 2/untitled065.tif]

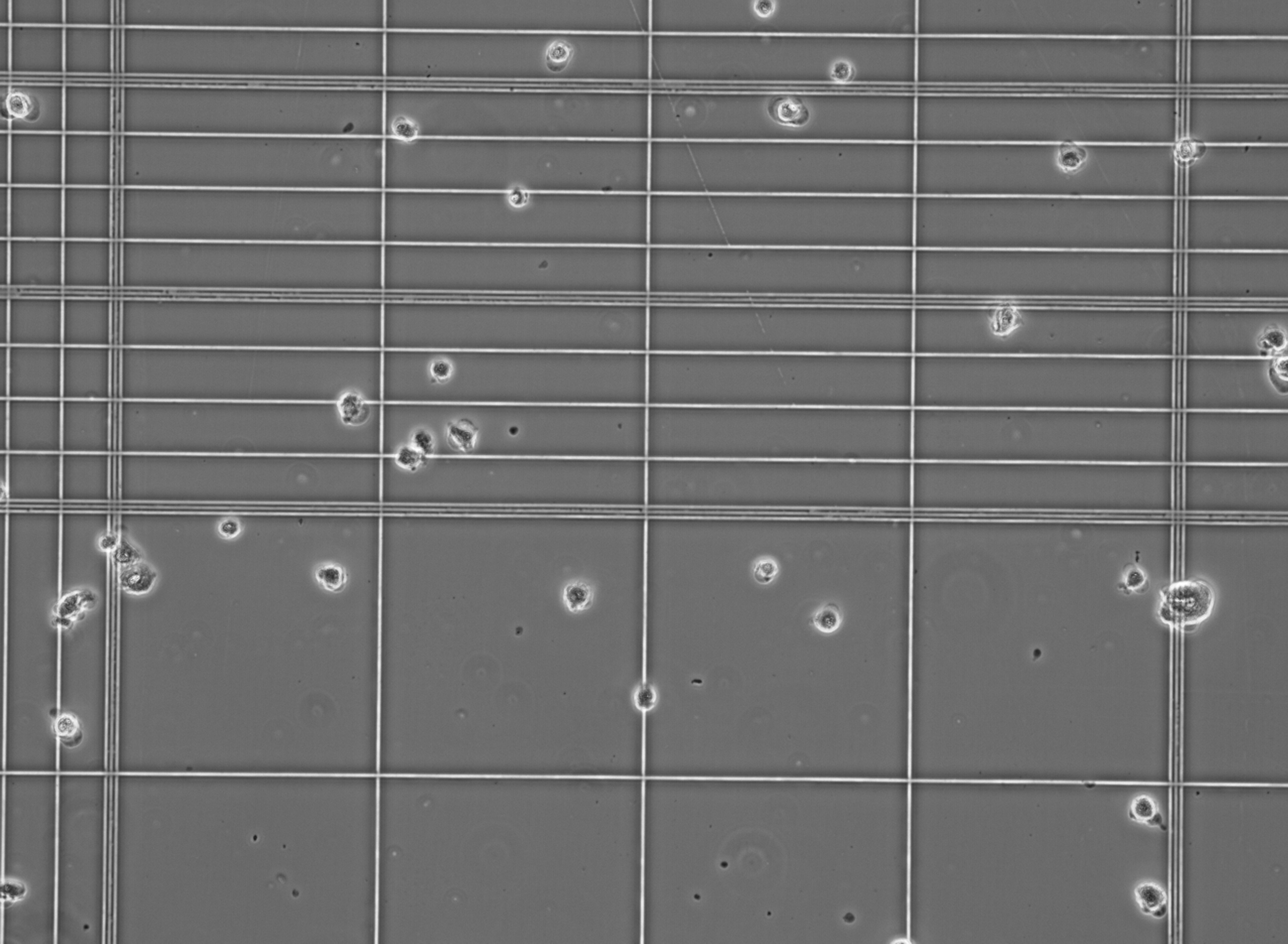

Supplement: S5 File — (ZIP) [file pone.0329484.s005.zip › S5 File - l-CSC 2/l-CSC 2/untitled066.tif]

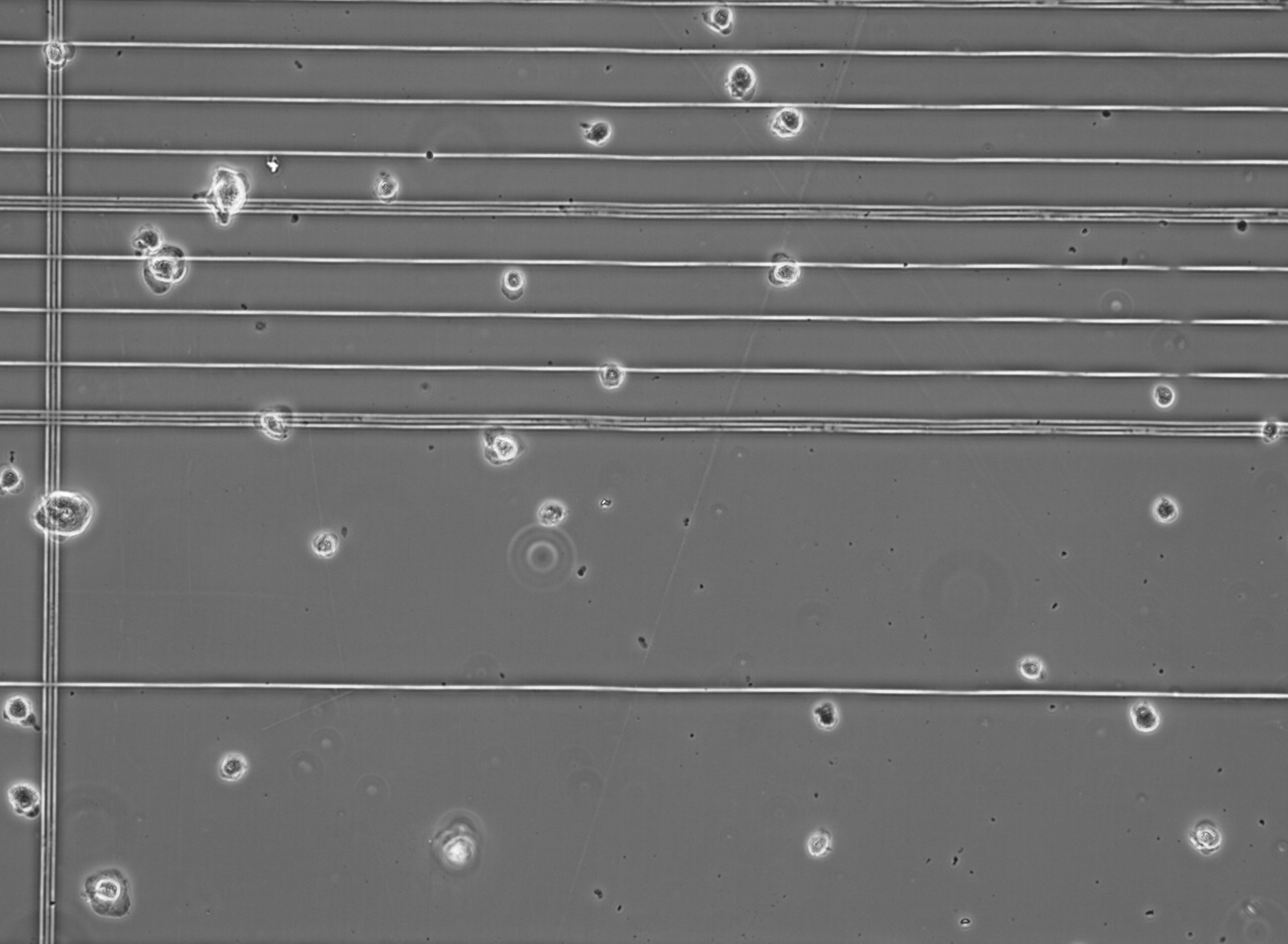

Supplement: S5 File — (ZIP) [file pone.0329484.s005.zip › S5 File - l-CSC 2/l-CSC 2/untitled067.tif]

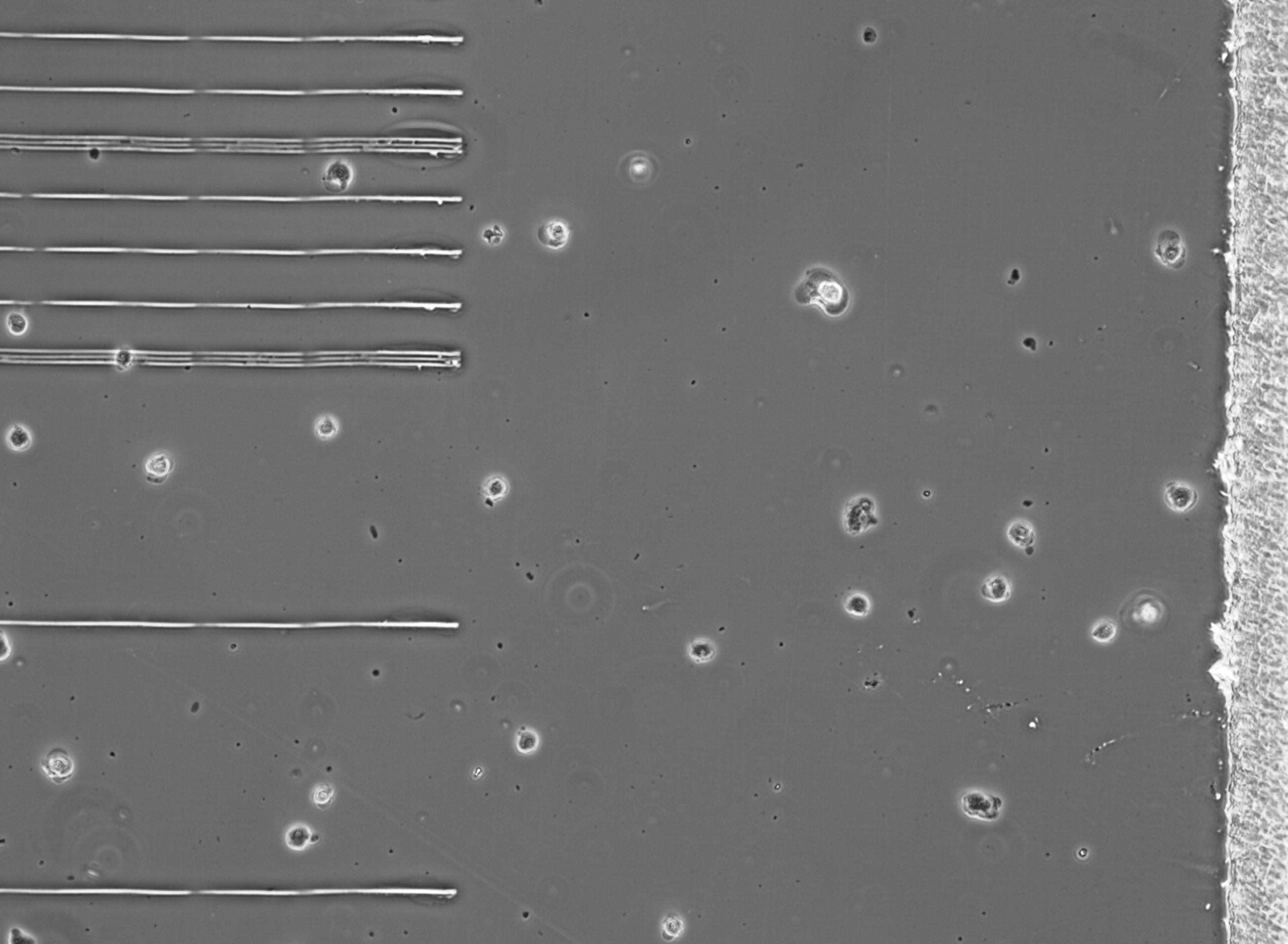

Supplement: S5 File — (ZIP) [file pone.0329484.s005.zip › S5 File - l-CSC 2/l-CSC 2/untitled068.tif]

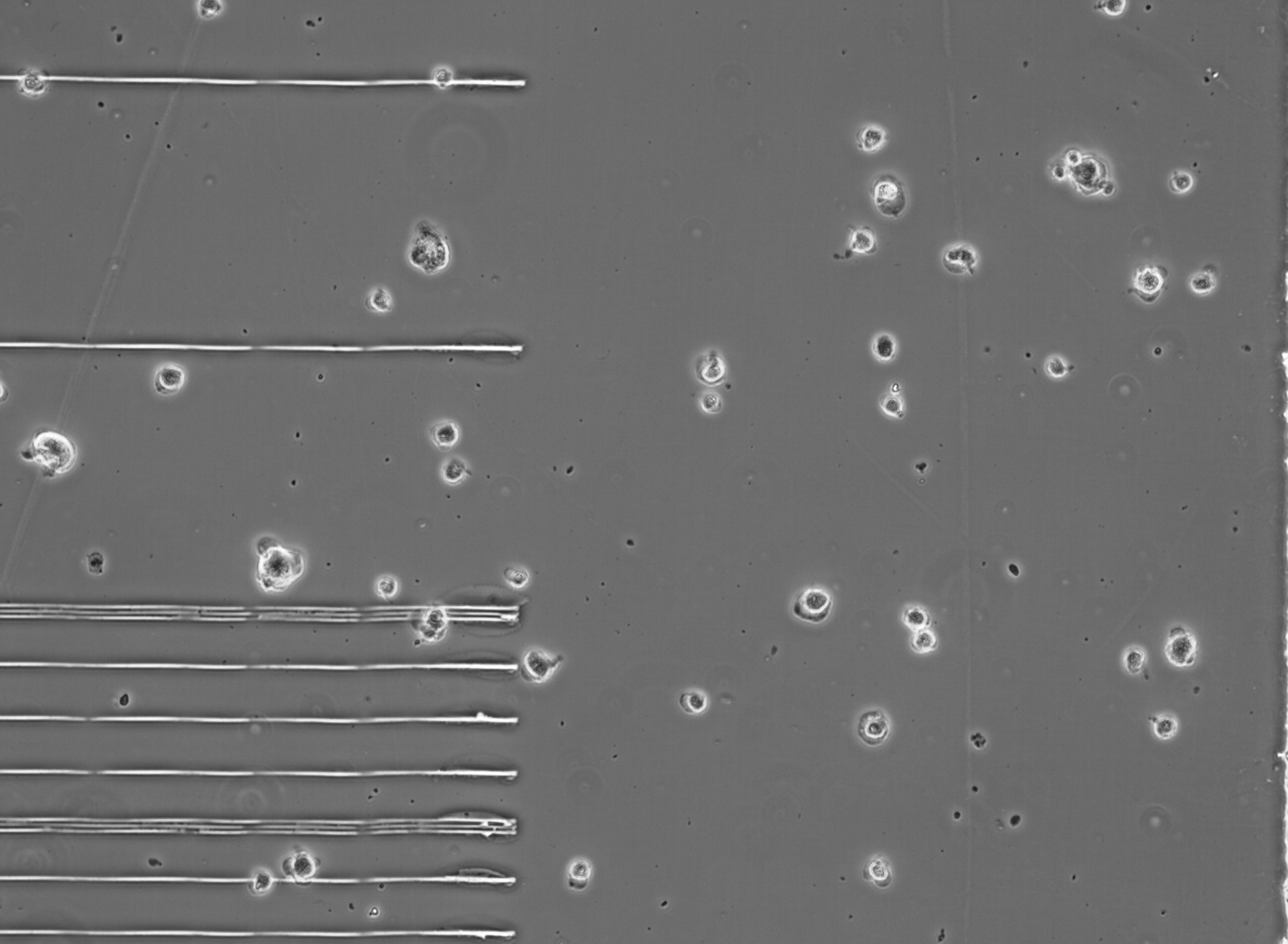

Supplement: S5 File — (ZIP) [file pone.0329484.s005.zip › S5 File - l-CSC 2/l-CSC 2/untitled069.tif]

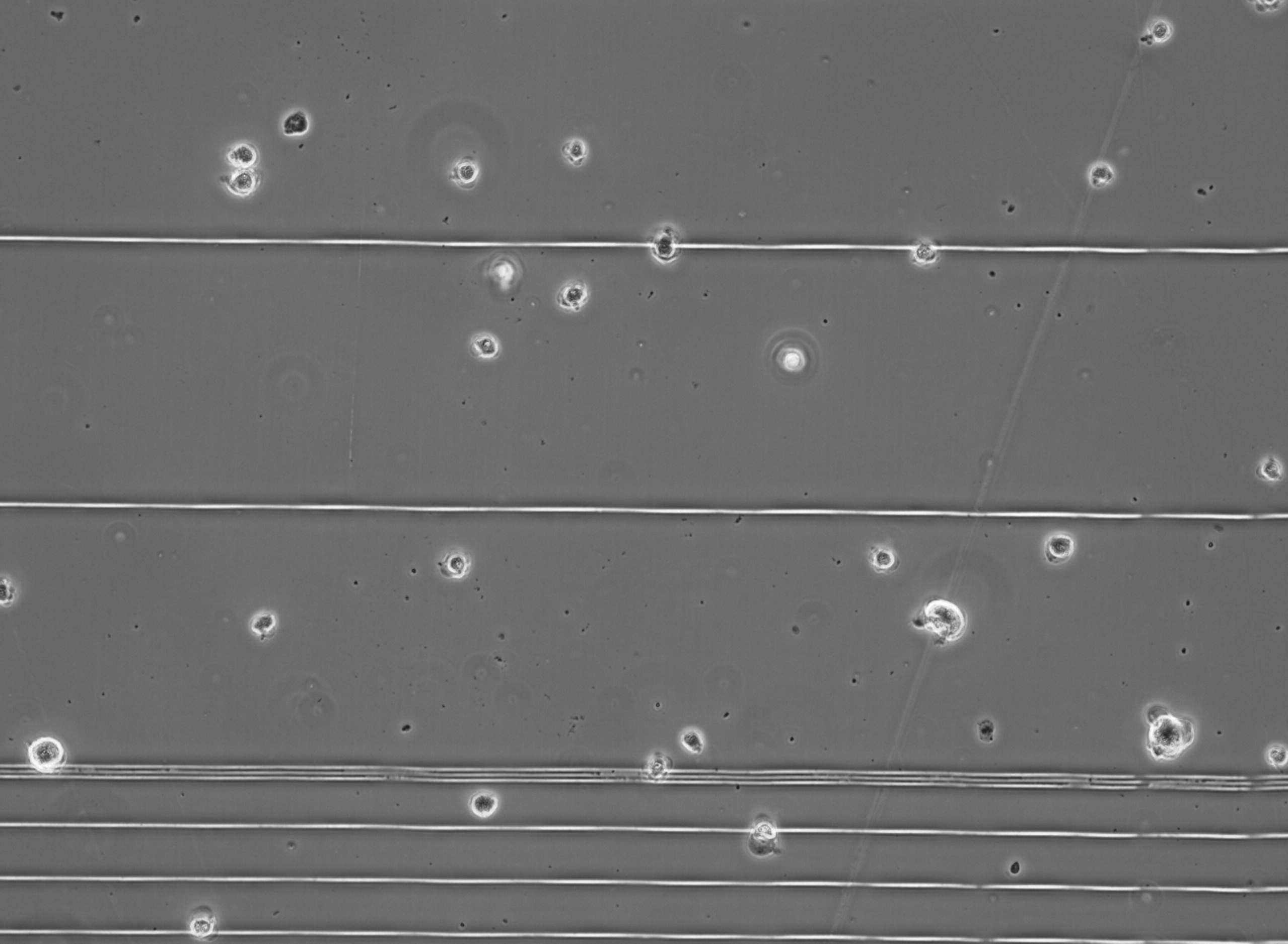

Supplement: S5 File — (ZIP) [file pone.0329484.s005.zip › S5 File - l-CSC 2/l-CSC 2/untitled070.tif]

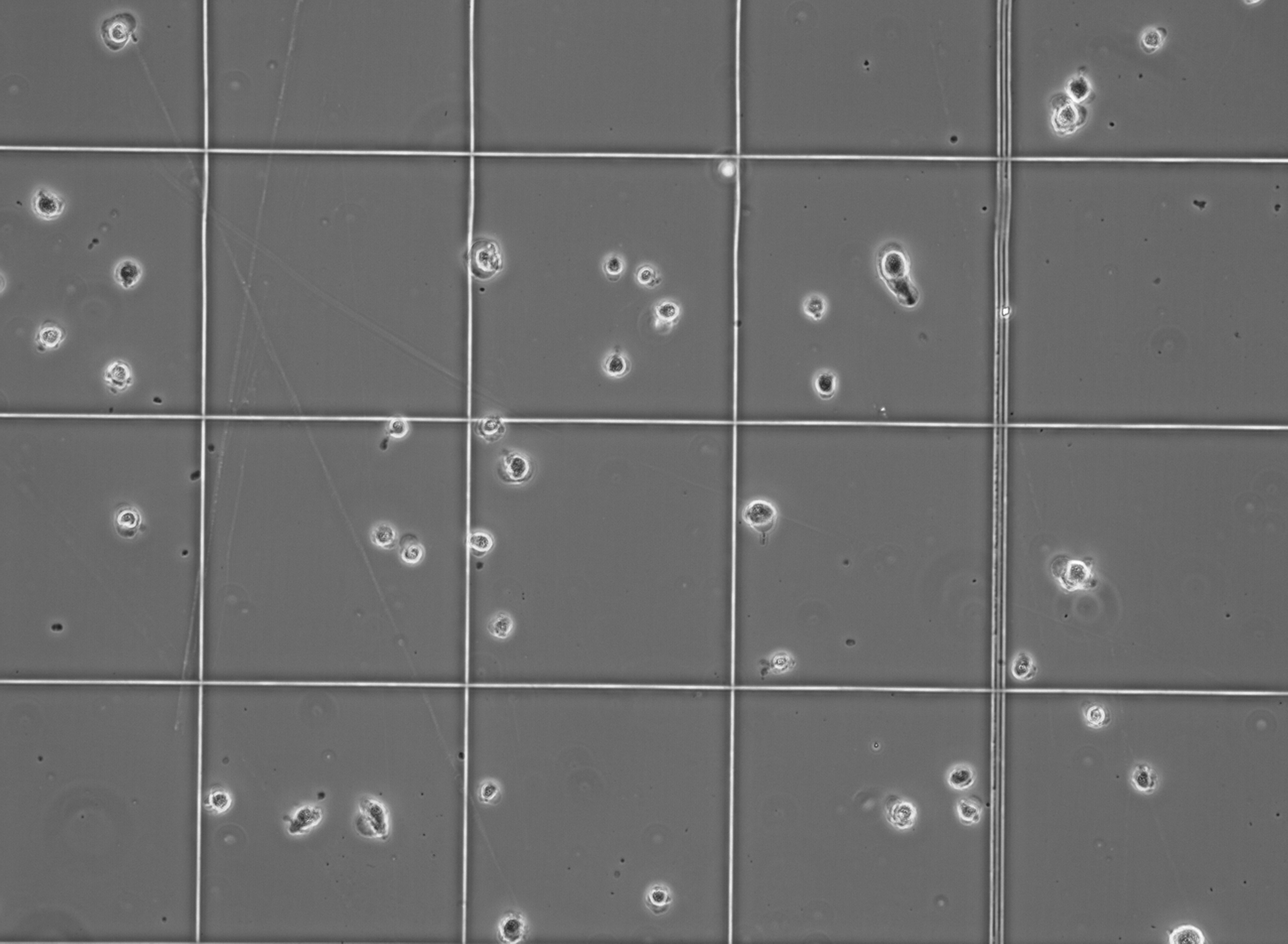

Supplement: S5 File — (ZIP) [file pone.0329484.s005.zip › S5 File - l-CSC 2/l-CSC 2/untitled071.tif]

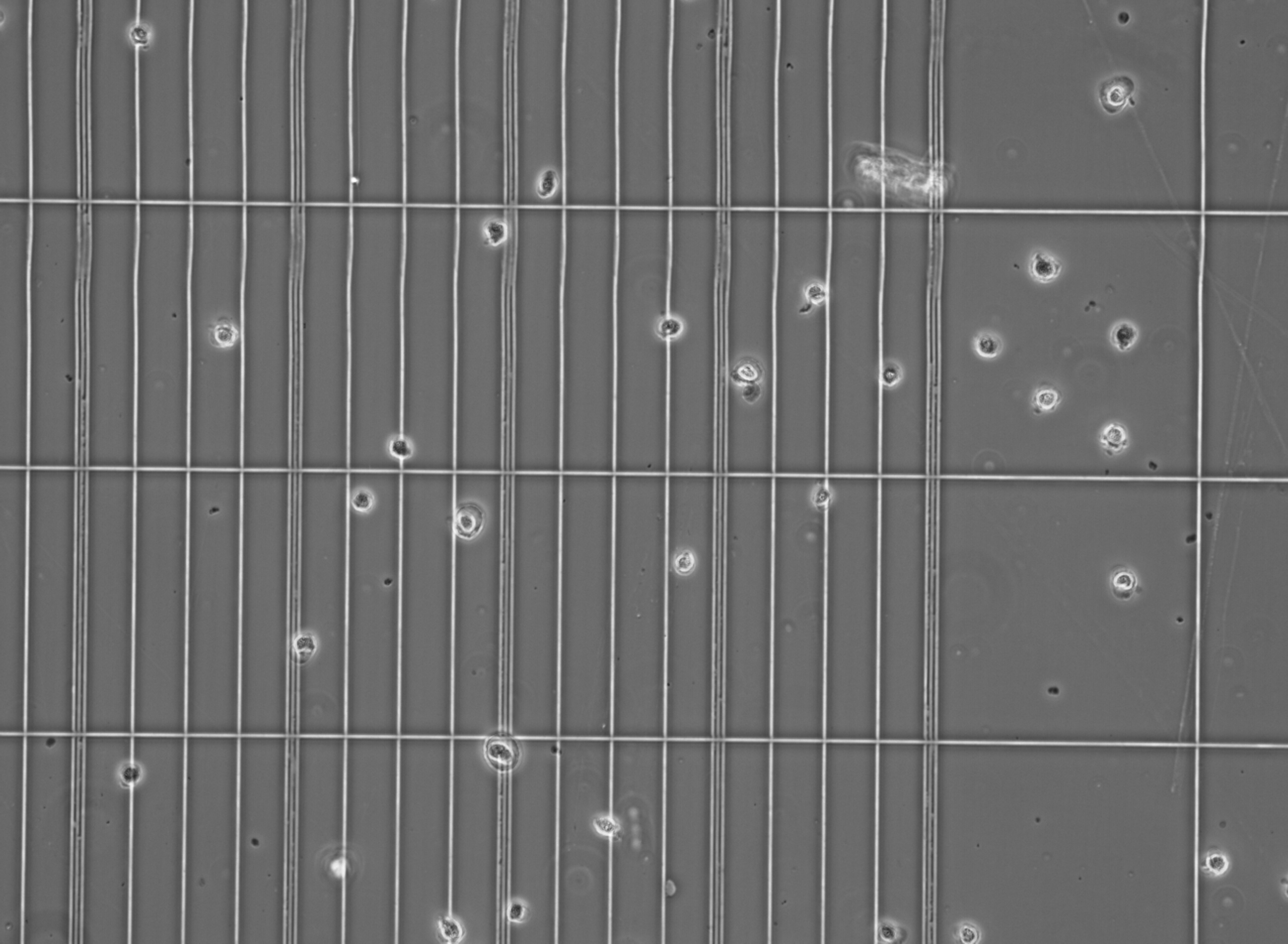

Supplement: S5 File — (ZIP) [file pone.0329484.s005.zip › S5 File - l-CSC 2/l-CSC 2/untitled072.tif]

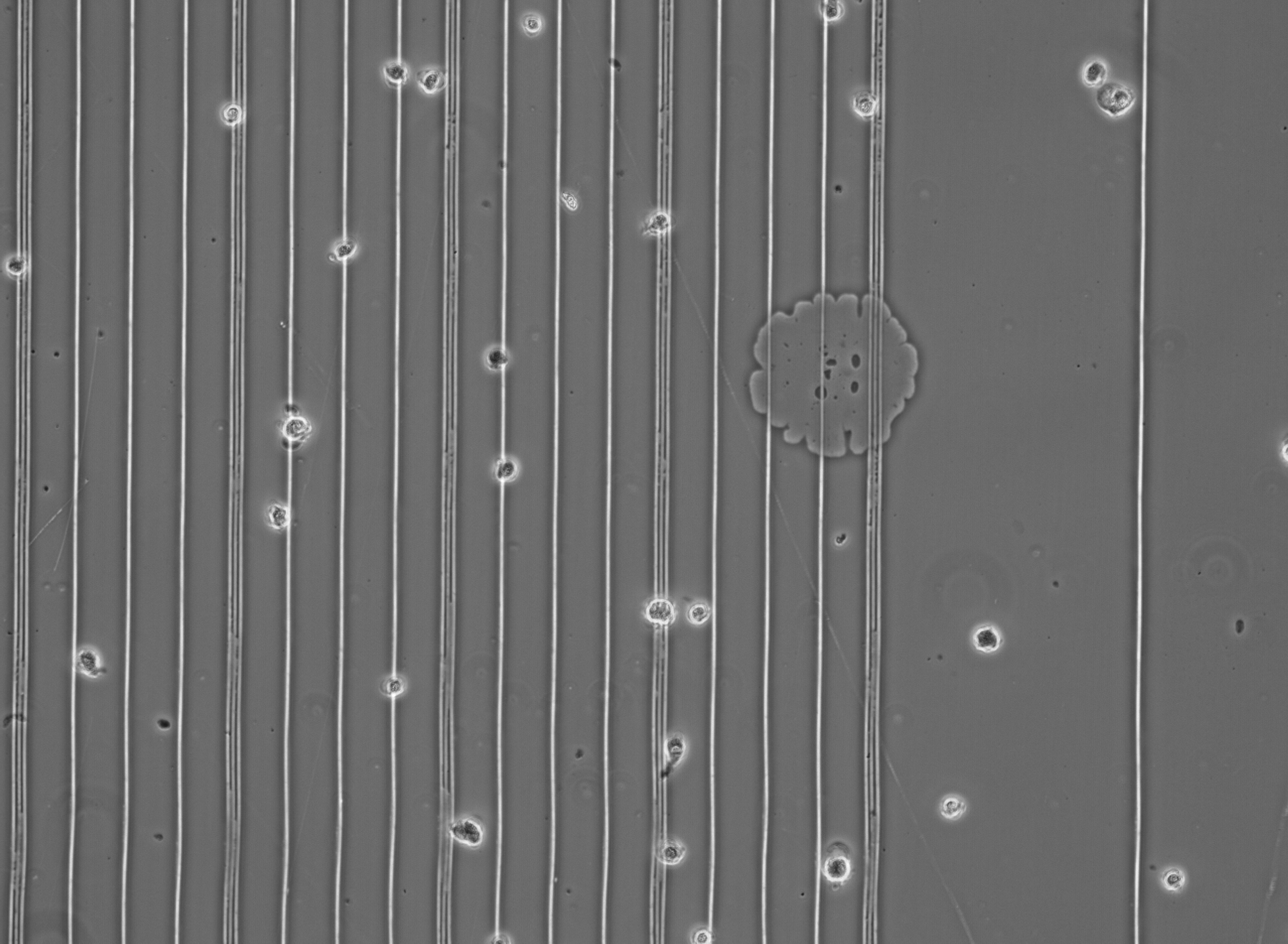

Supplement: S5 File — (ZIP) [file pone.0329484.s005.zip › S5 File - l-CSC 2/l-CSC 2/untitled073.tif]

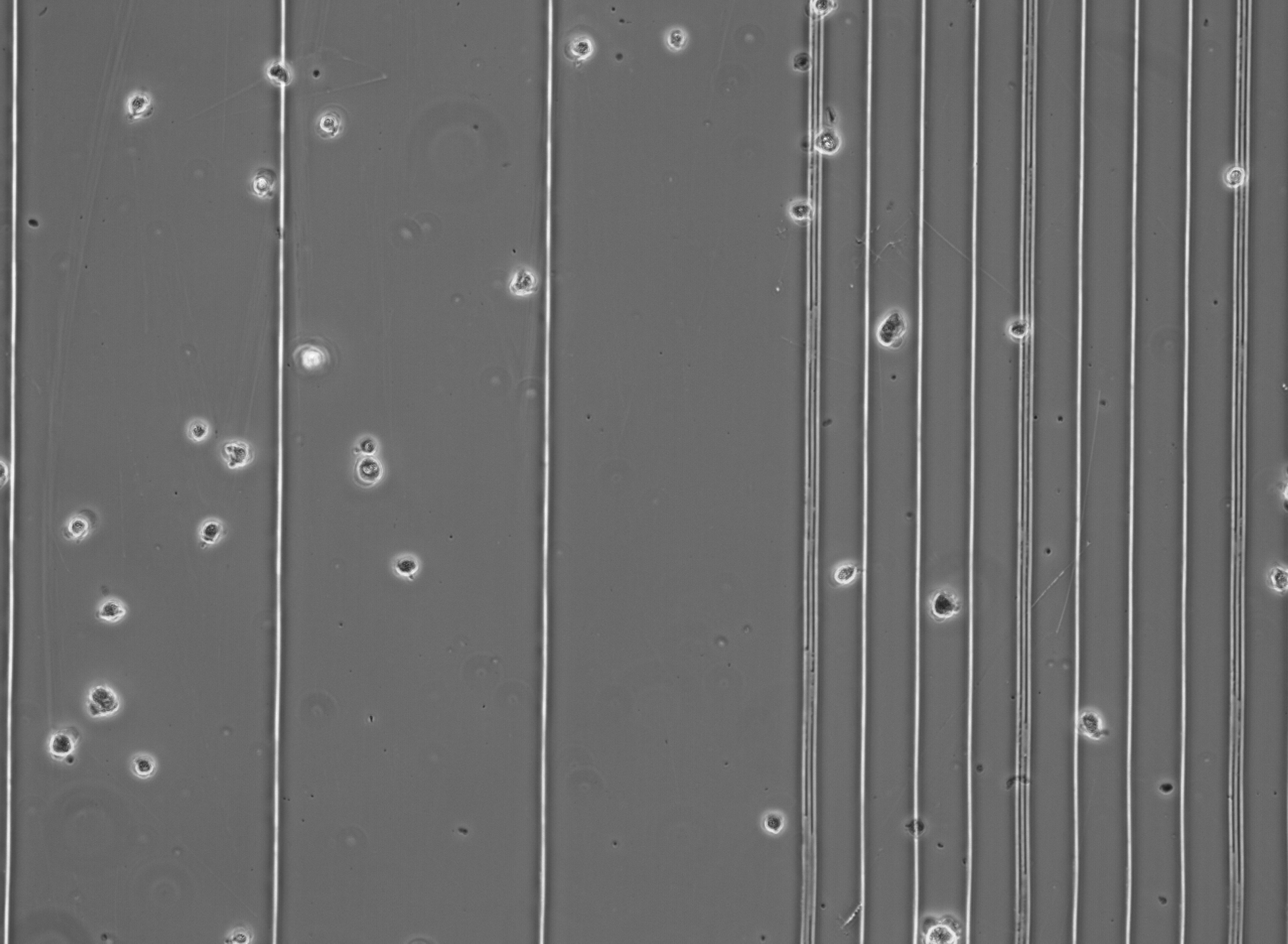

Supplement: S5 File — (ZIP) [file pone.0329484.s005.zip › S5 File - l-CSC 2/l-CSC 2/untitled074.tif]

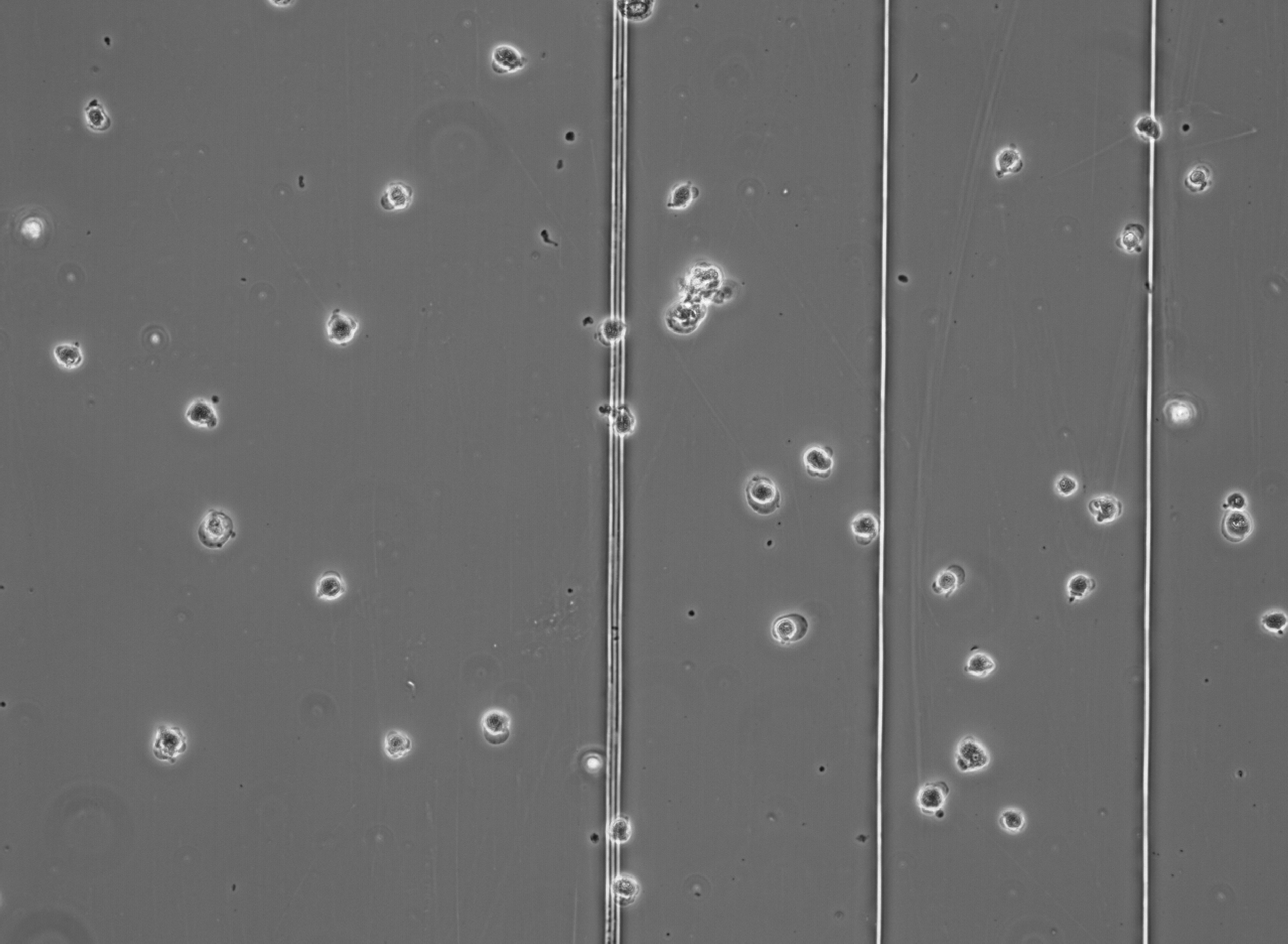

Supplement: S5 File — (ZIP) [file pone.0329484.s005.zip › S5 File - l-CSC 2/l-CSC 2/untitled075.tif]

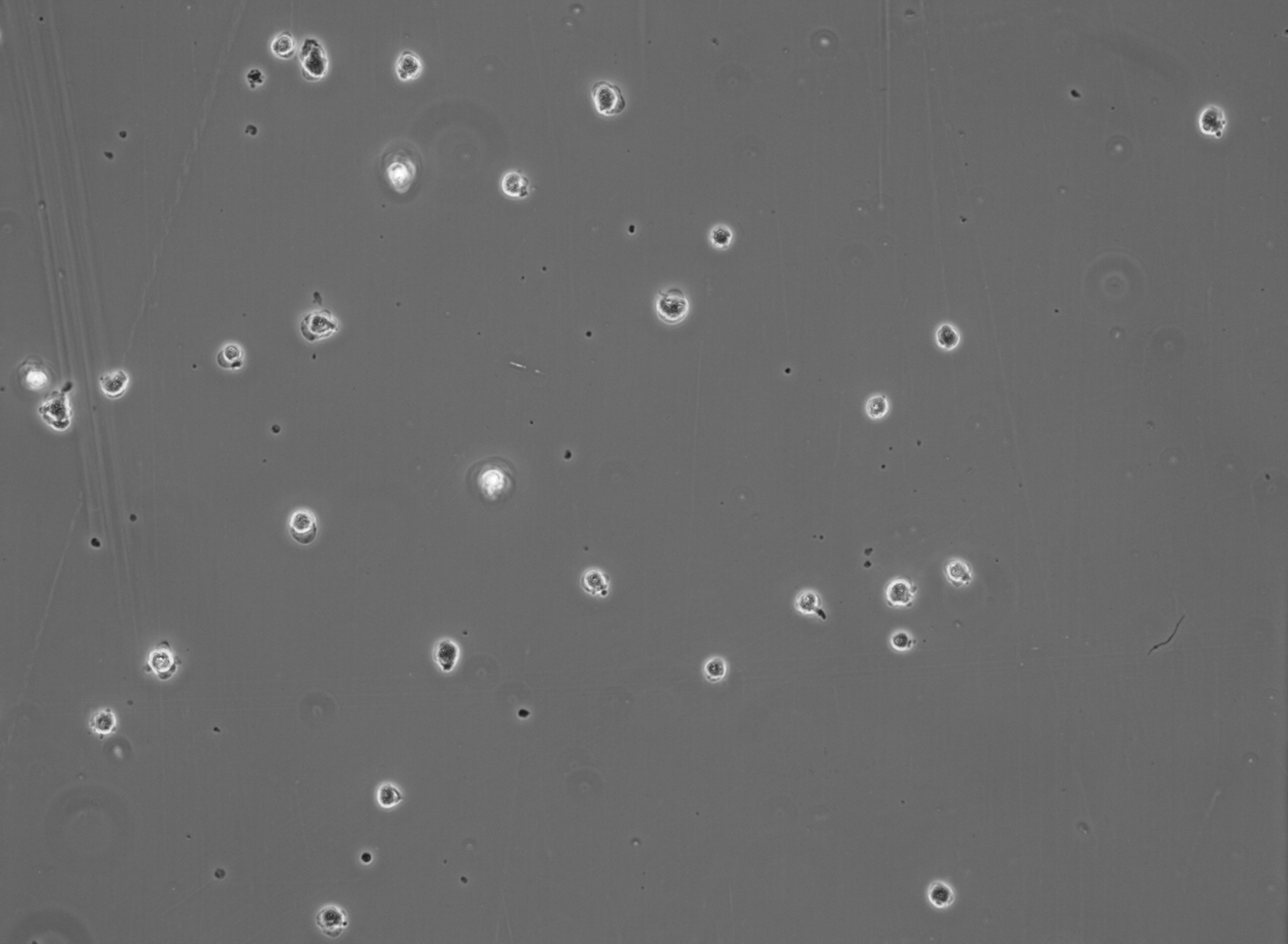

Supplement: S5 File — (ZIP) [file pone.0329484.s005.zip › S5 File - l-CSC 2/l-CSC 2/untitled076.tif]

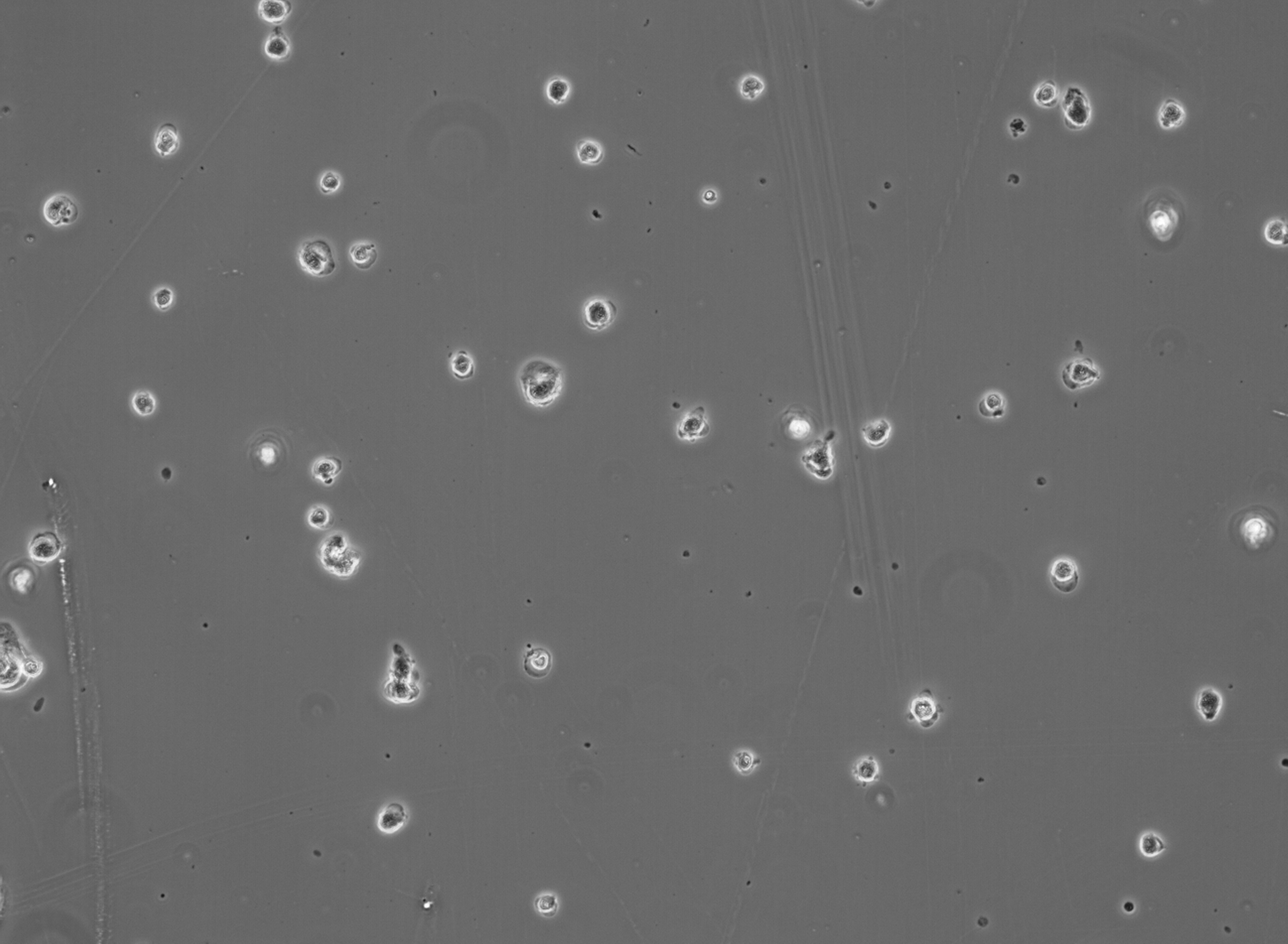

Supplement: S5 File — (ZIP) [file pone.0329484.s005.zip › S5 File - l-CSC 2/l-CSC 2/untitled077.tif]

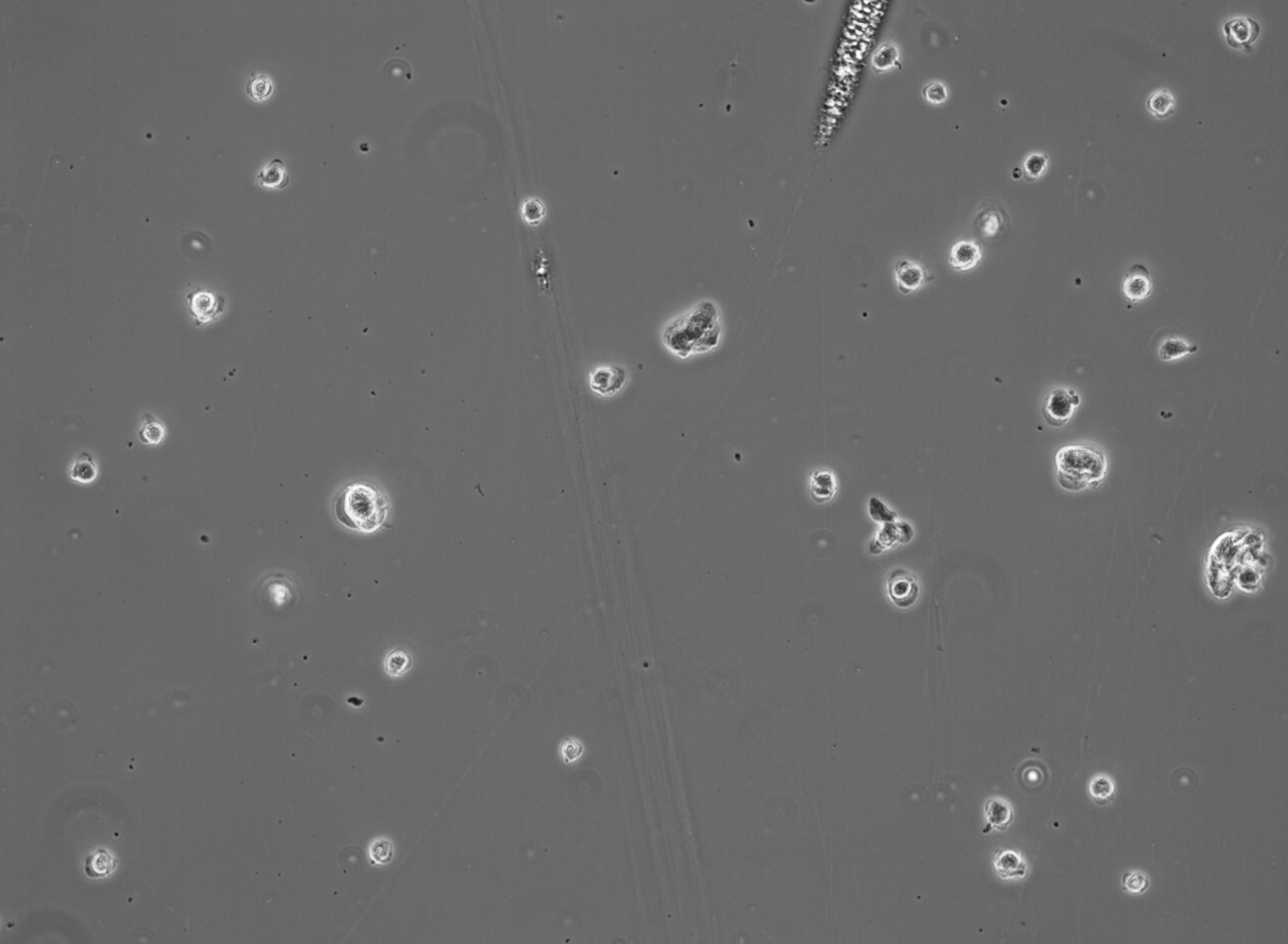

Supplement: S5 File — (ZIP) [file pone.0329484.s005.zip › S5 File - l-CSC 2/l-CSC 2/untitled078.tif]

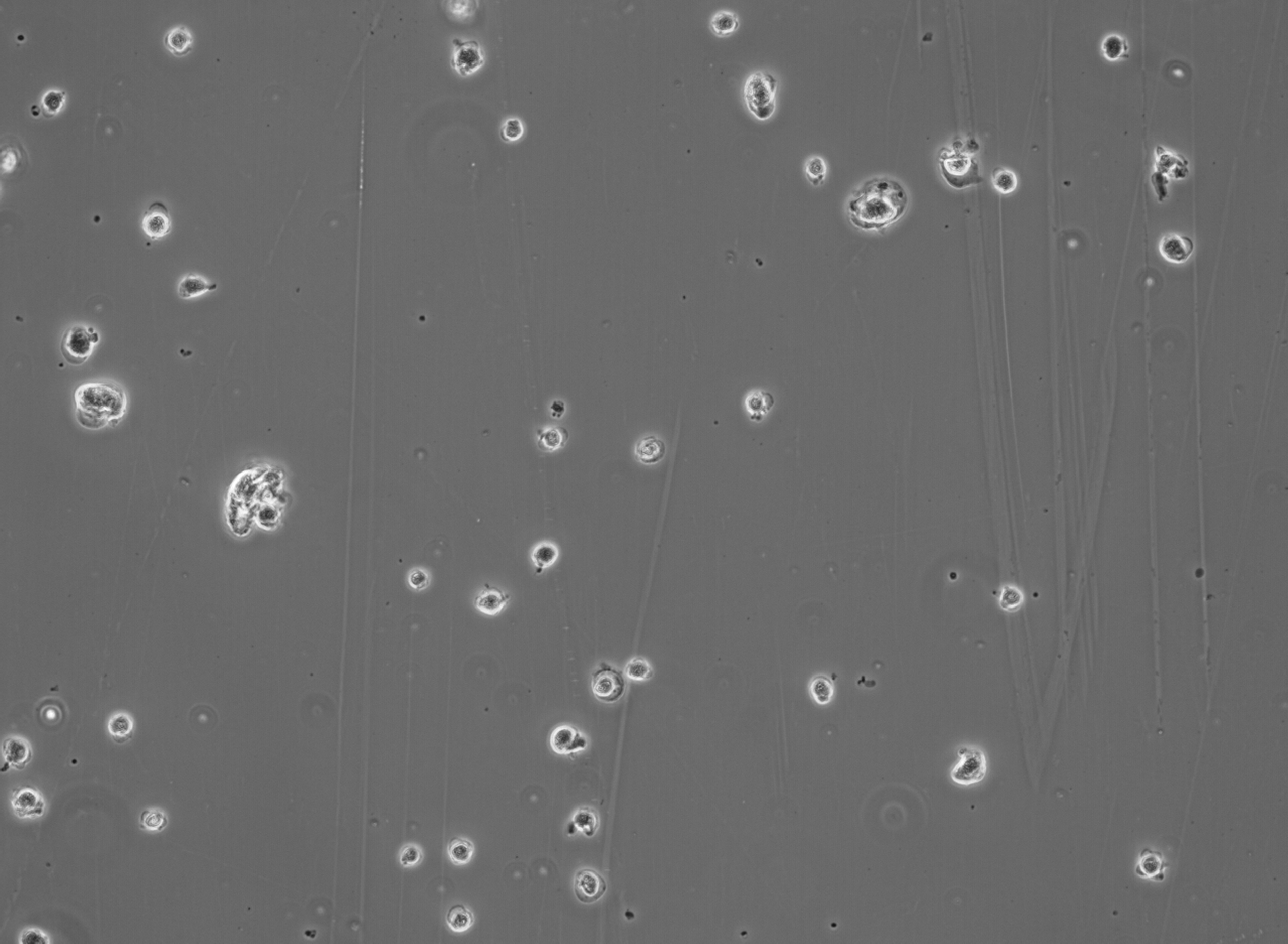

Supplement: S5 File — (ZIP) [file pone.0329484.s005.zip › S5 File - l-CSC 2/l-CSC 2/untitled079.tif]

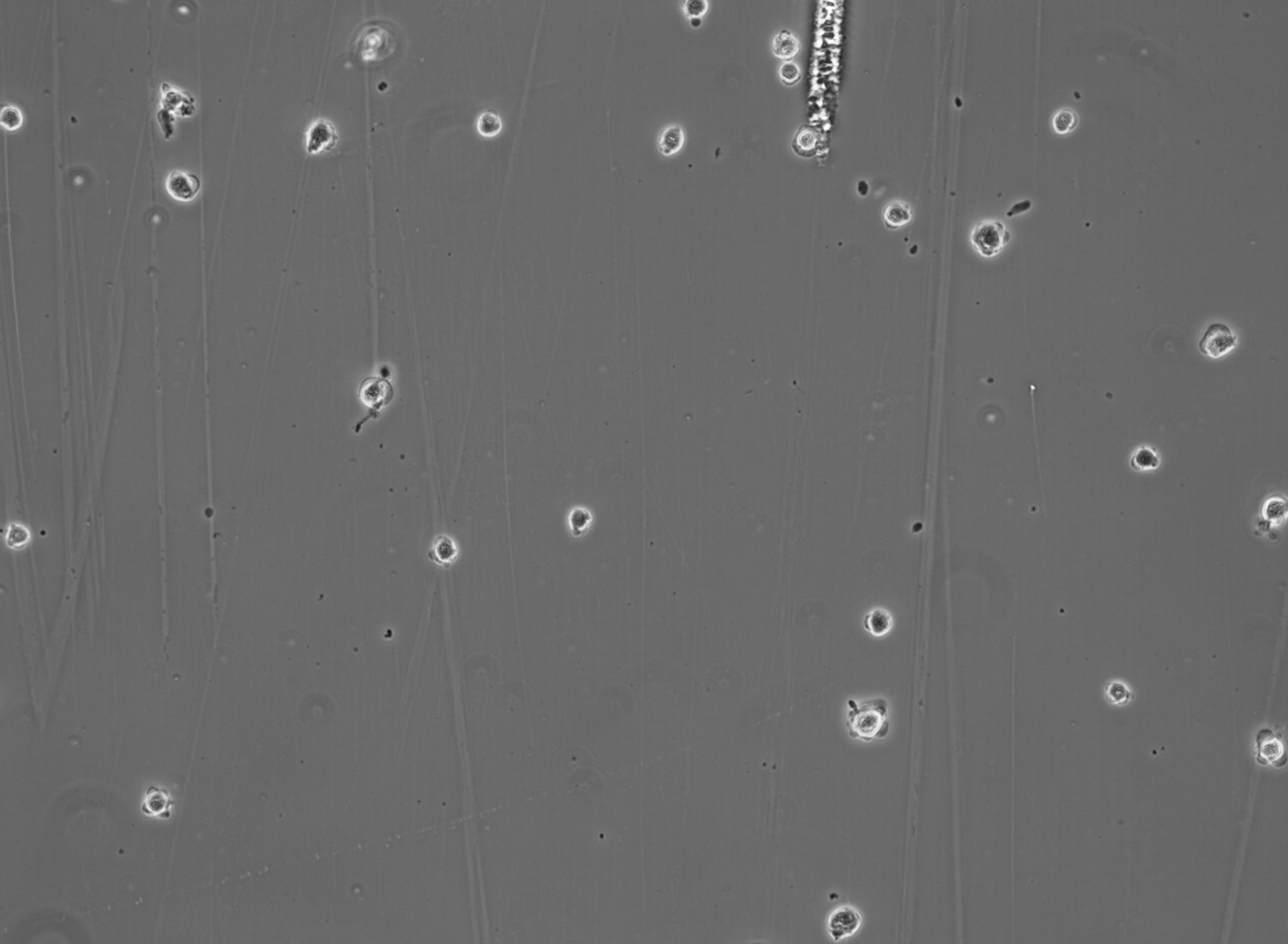

Supplement: S5 File — (ZIP) [file pone.0329484.s005.zip › S5 File - l-CSC 2/l-CSC 2/untitled080.tif]

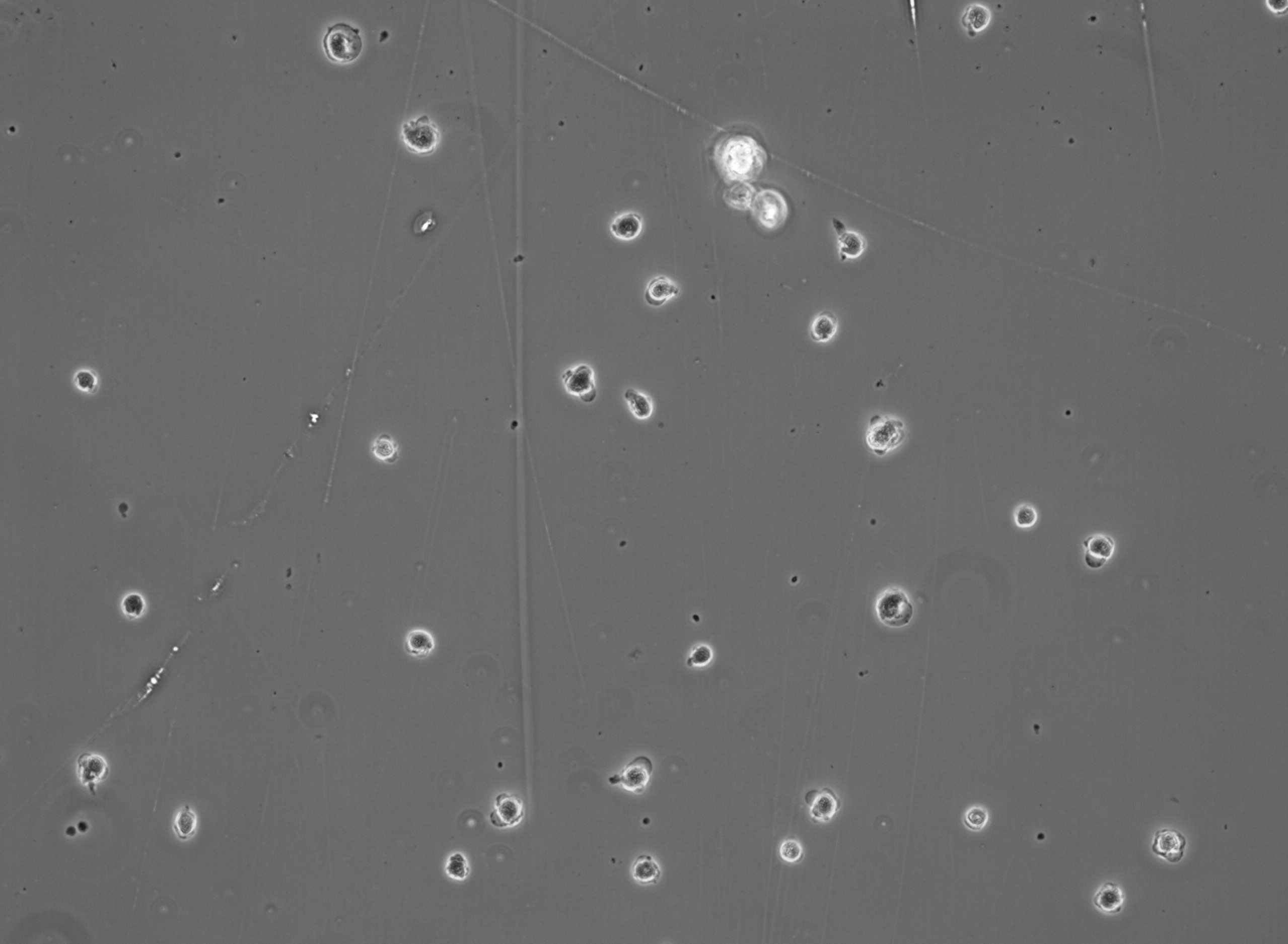

Supplement: S5 File — (ZIP) [file pone.0329484.s005.zip › S5 File - l-CSC 2/l-CSC 2/untitled081.tif]

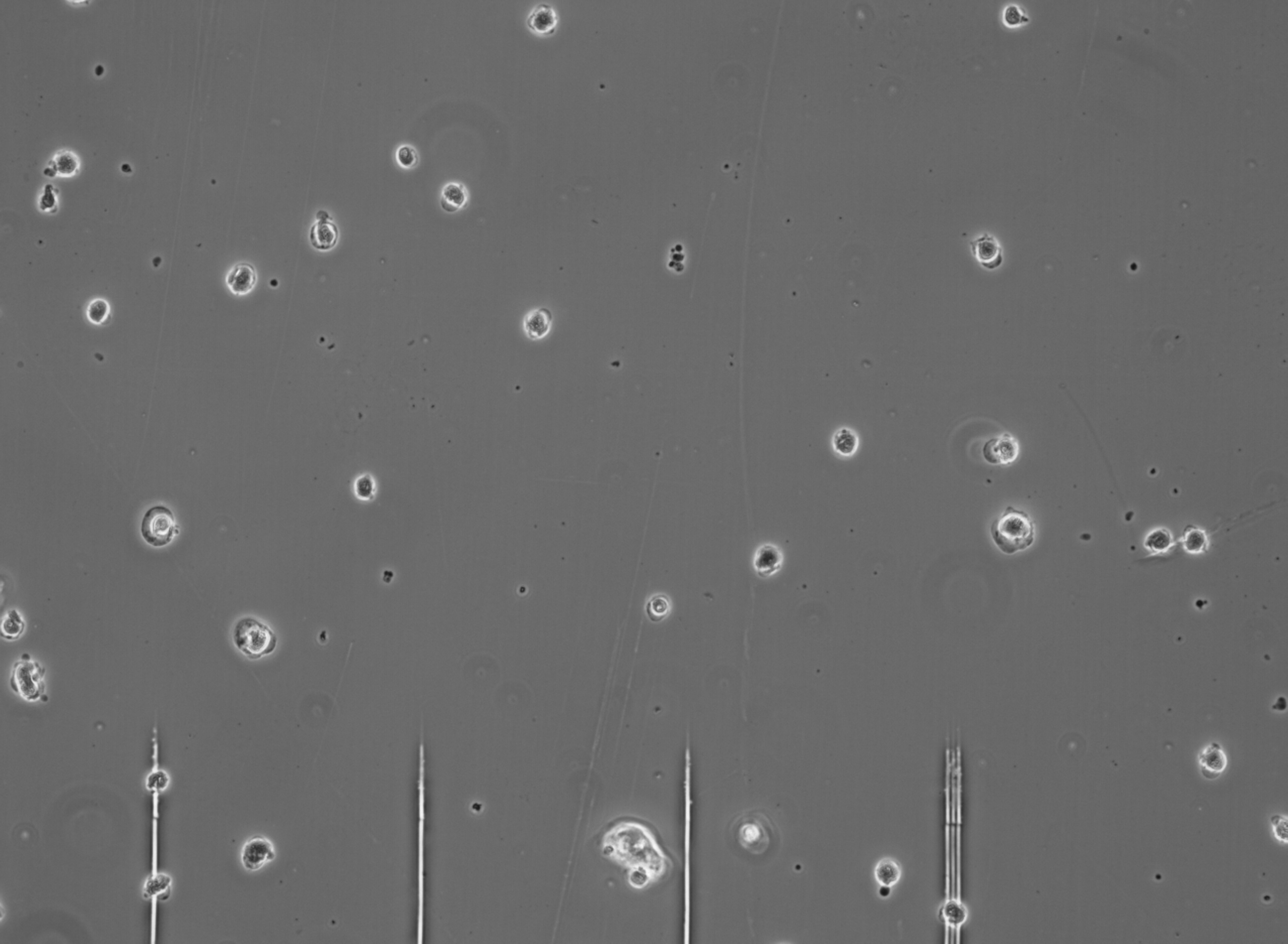

Supplement: S5 File — (ZIP) [file pone.0329484.s005.zip › S5 File - l-CSC 2/l-CSC 2/untitled082.tif]

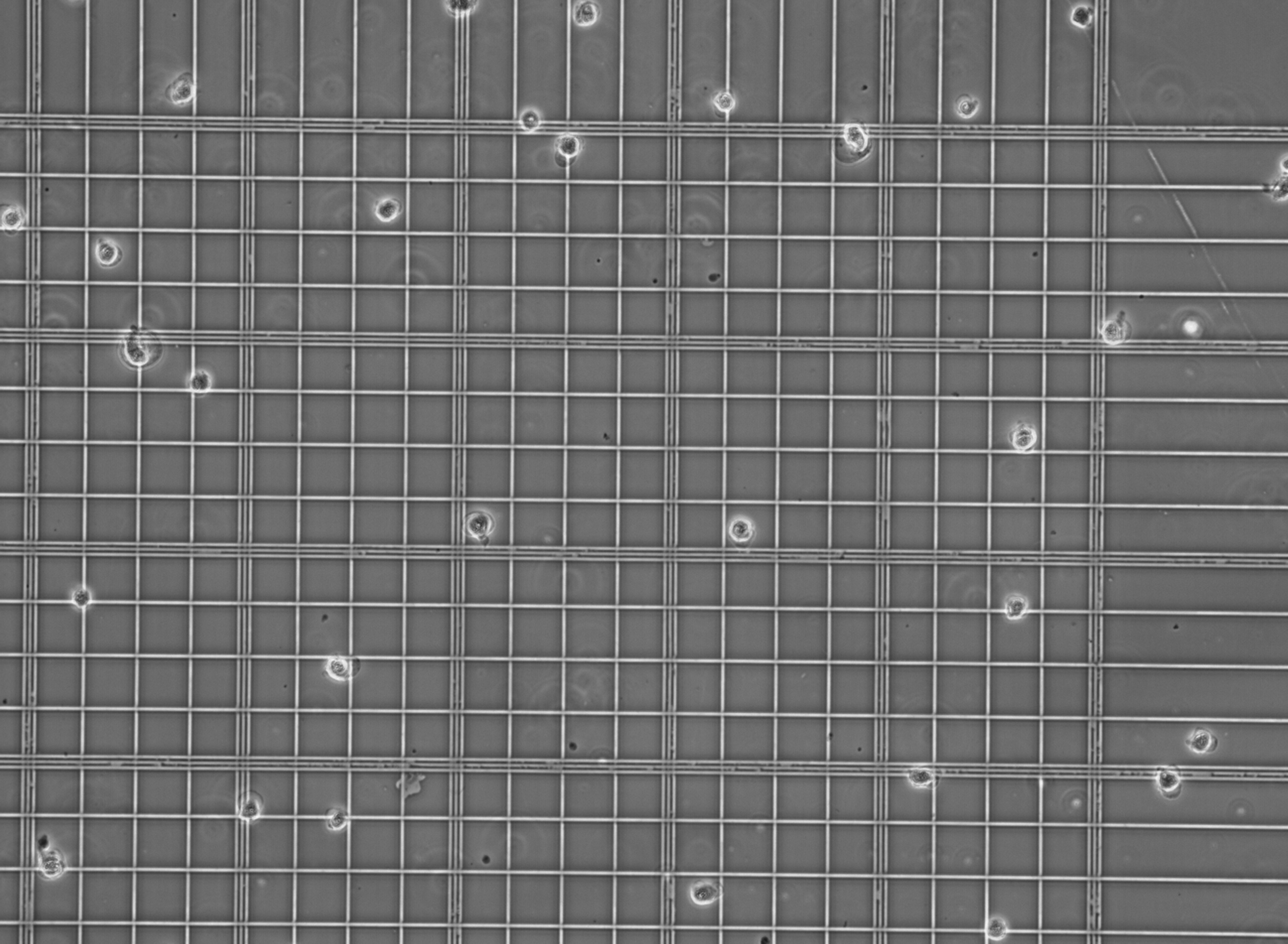

Supplement: S5 File — (ZIP) [file pone.0329484.s005.zip › S5 File - l-CSC 2/l-CSC 2/untitled083.tif]

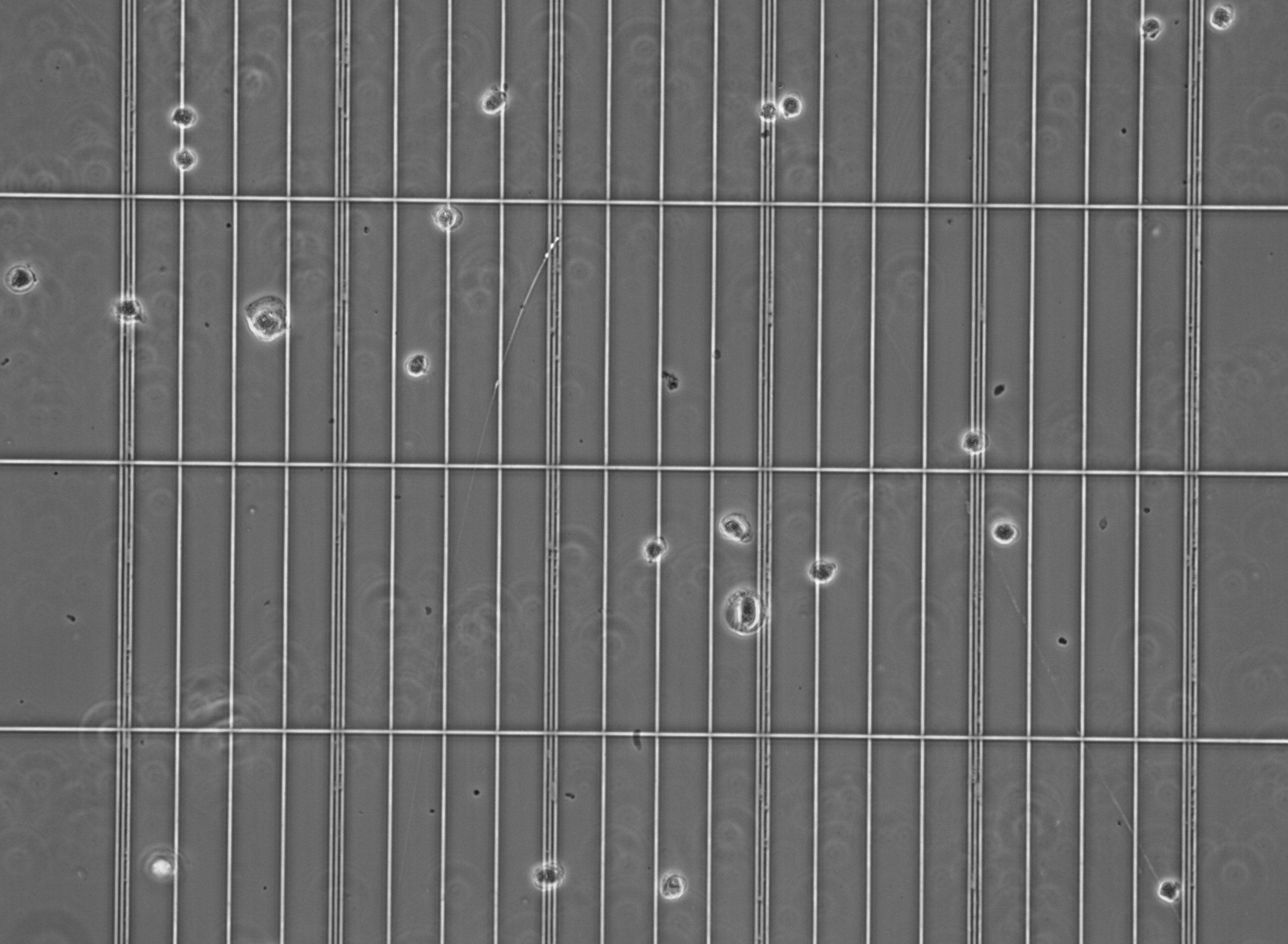

Supplement: S5 File — (ZIP) [file pone.0329484.s005.zip › S5 File - l-CSC 2/l-CSC 2/untitled084.tif]

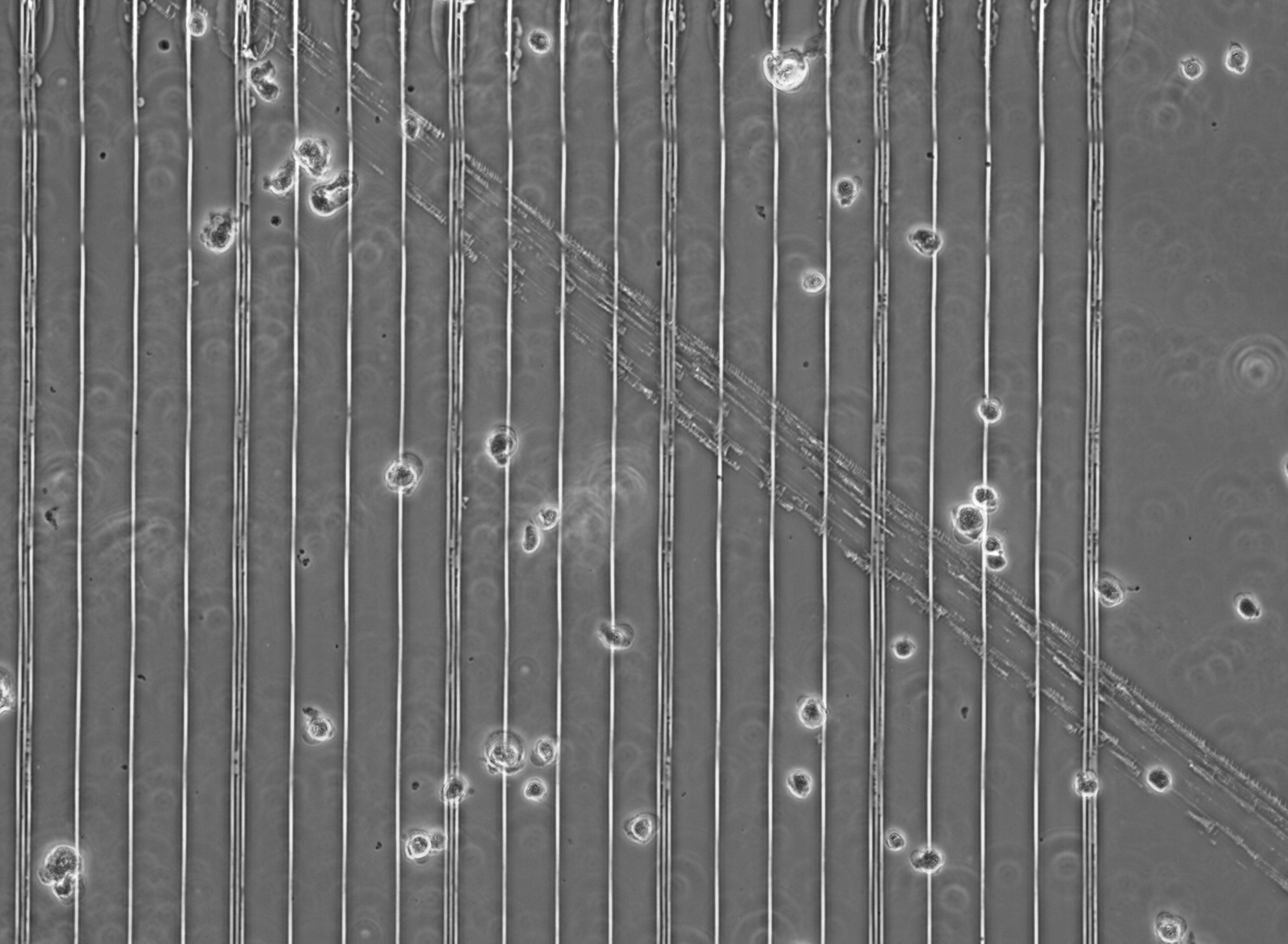

Supplement: S5 File — (ZIP) [file pone.0329484.s005.zip › S5 File - l-CSC 2/l-CSC 2/untitled085.tif]

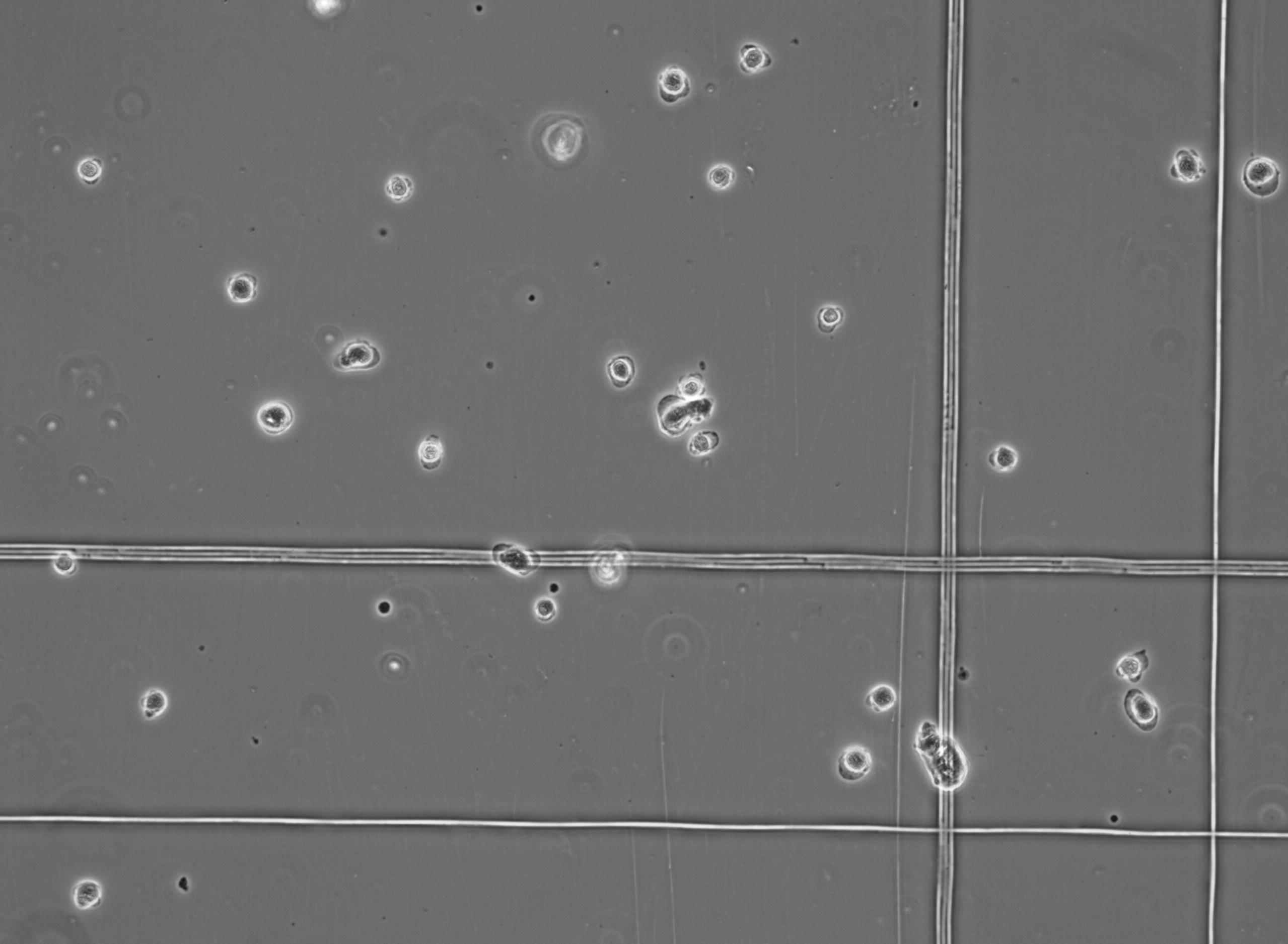

Supplement: S6 File — (ZIP) [file pone.0329484.s006.zip › S6 File - l-CSC 3/l-CSC 3/untitled086.tif]

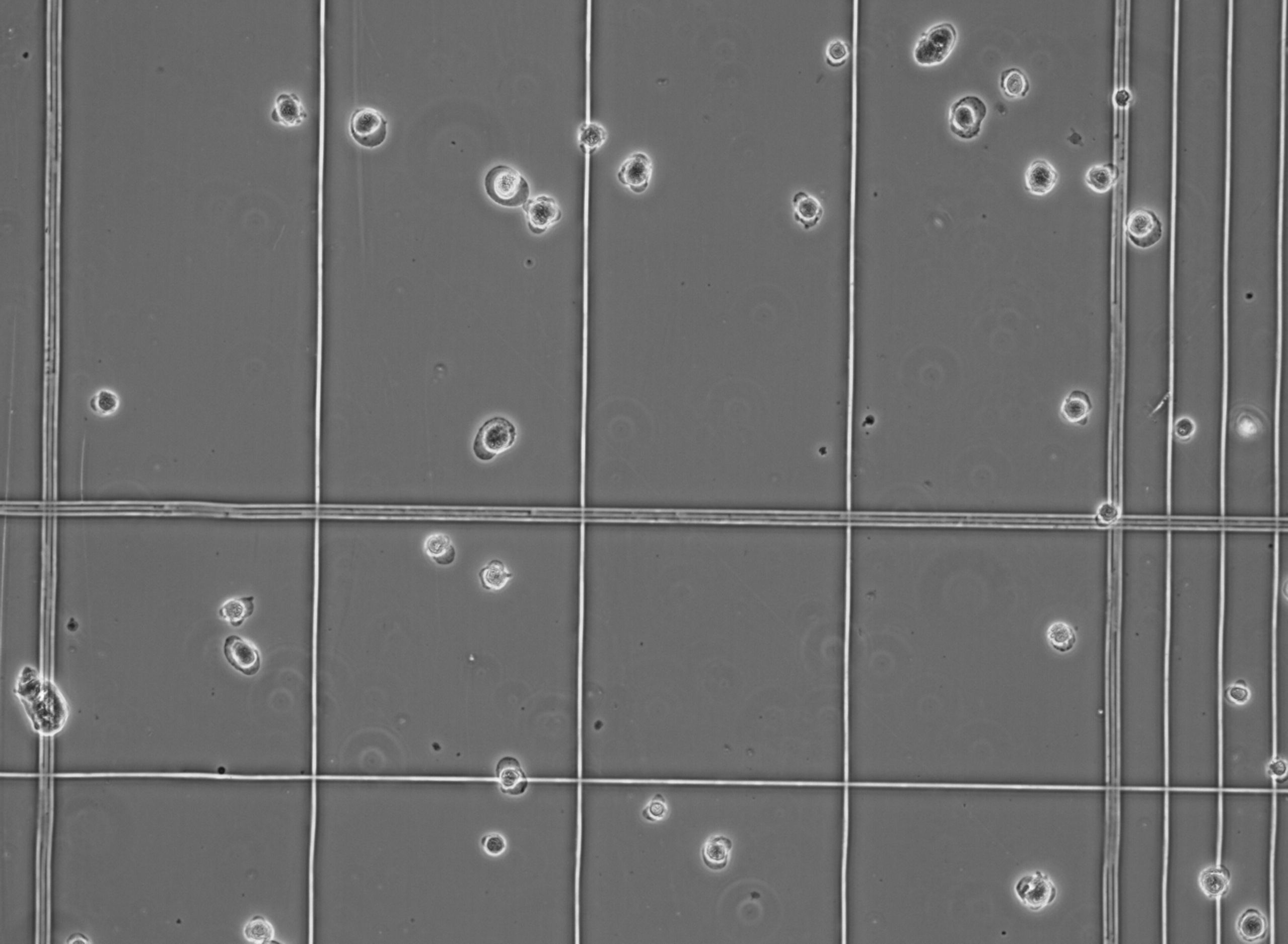

Supplement: S6 File — (ZIP) [file pone.0329484.s006.zip › S6 File - l-CSC 3/l-CSC 3/untitled087.tif]

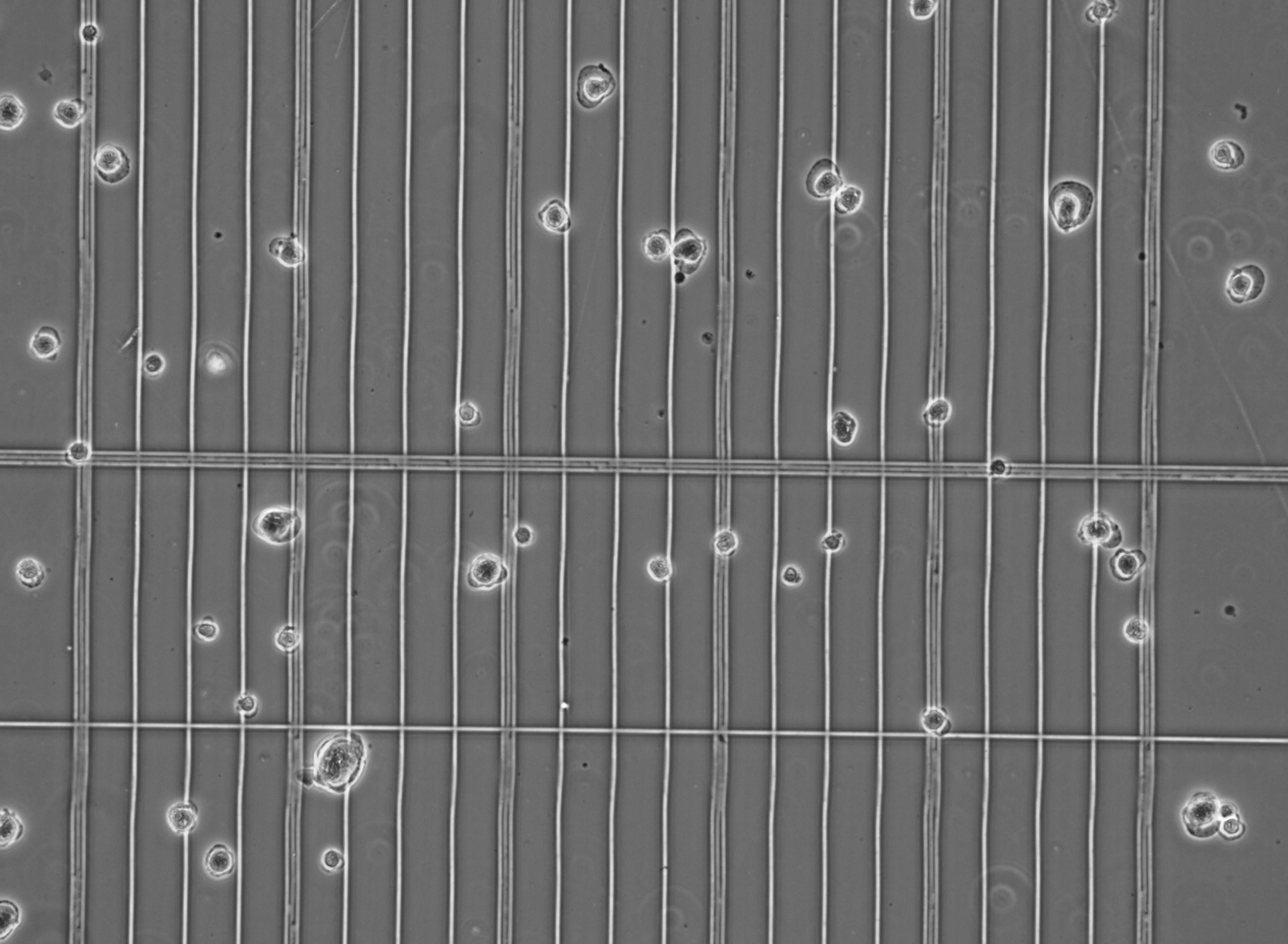

Supplement: S6 File — (ZIP) [file pone.0329484.s006.zip › S6 File - l-CSC 3/l-CSC 3/untitled088.tif]

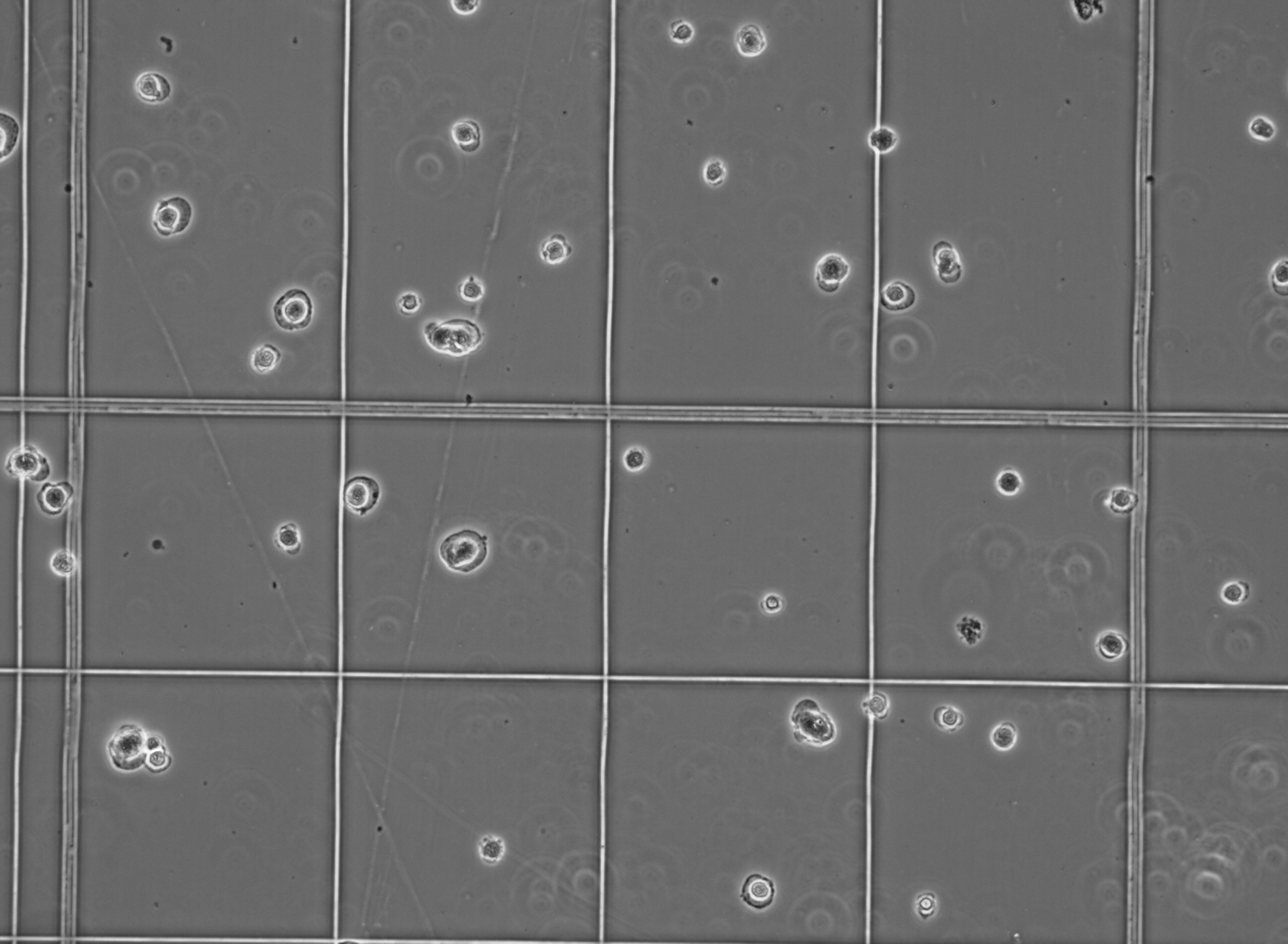

Supplement: S6 File — (ZIP) [file pone.0329484.s006.zip › S6 File - l-CSC 3/l-CSC 3/untitled089.tif]

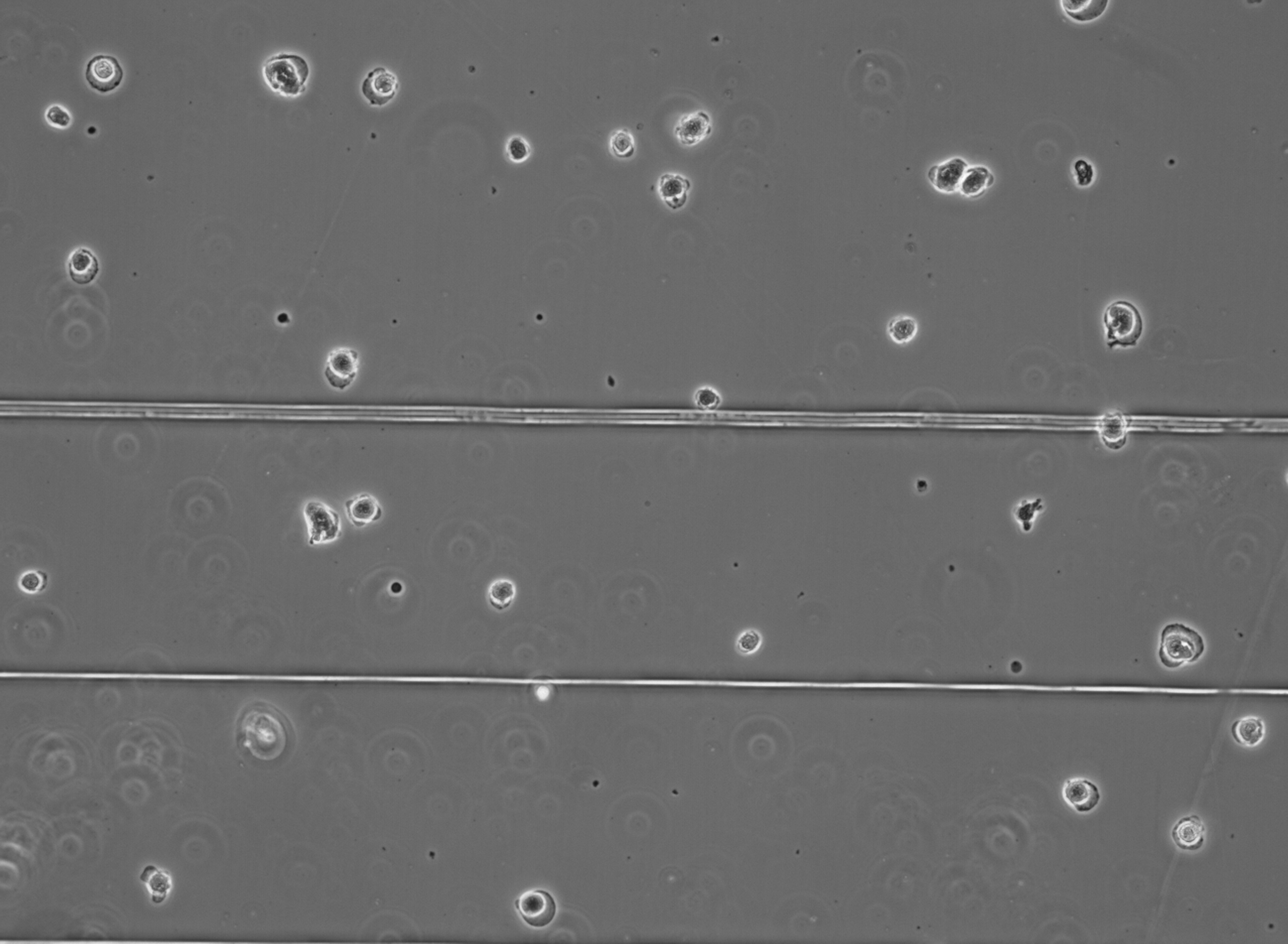

Supplement: S6 File — (ZIP) [file pone.0329484.s006.zip › S6 File - l-CSC 3/l-CSC 3/untitled090.tif]

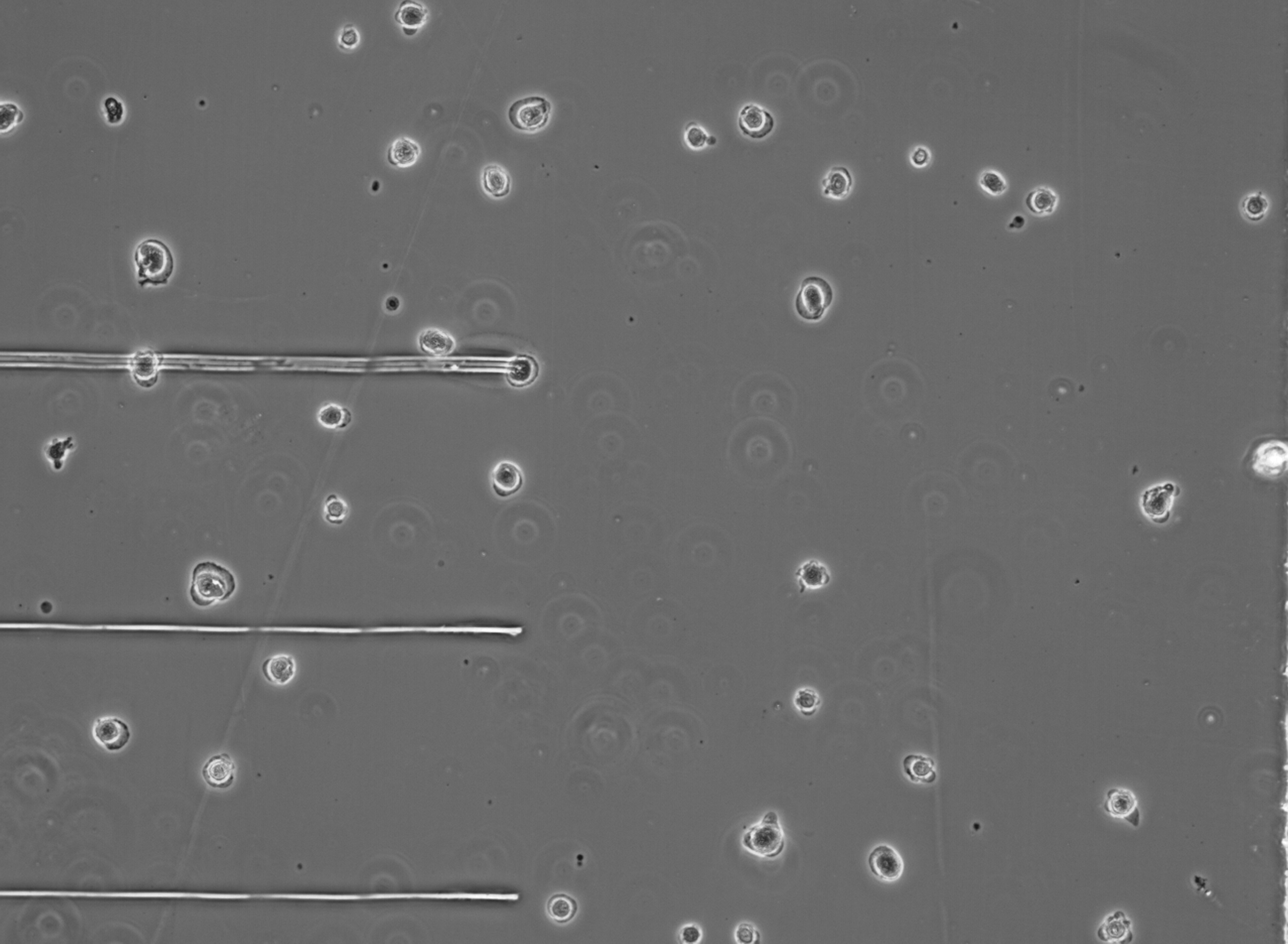

Supplement: S6 File — (ZIP) [file pone.0329484.s006.zip › S6 File - l-CSC 3/l-CSC 3/untitled091.tif]

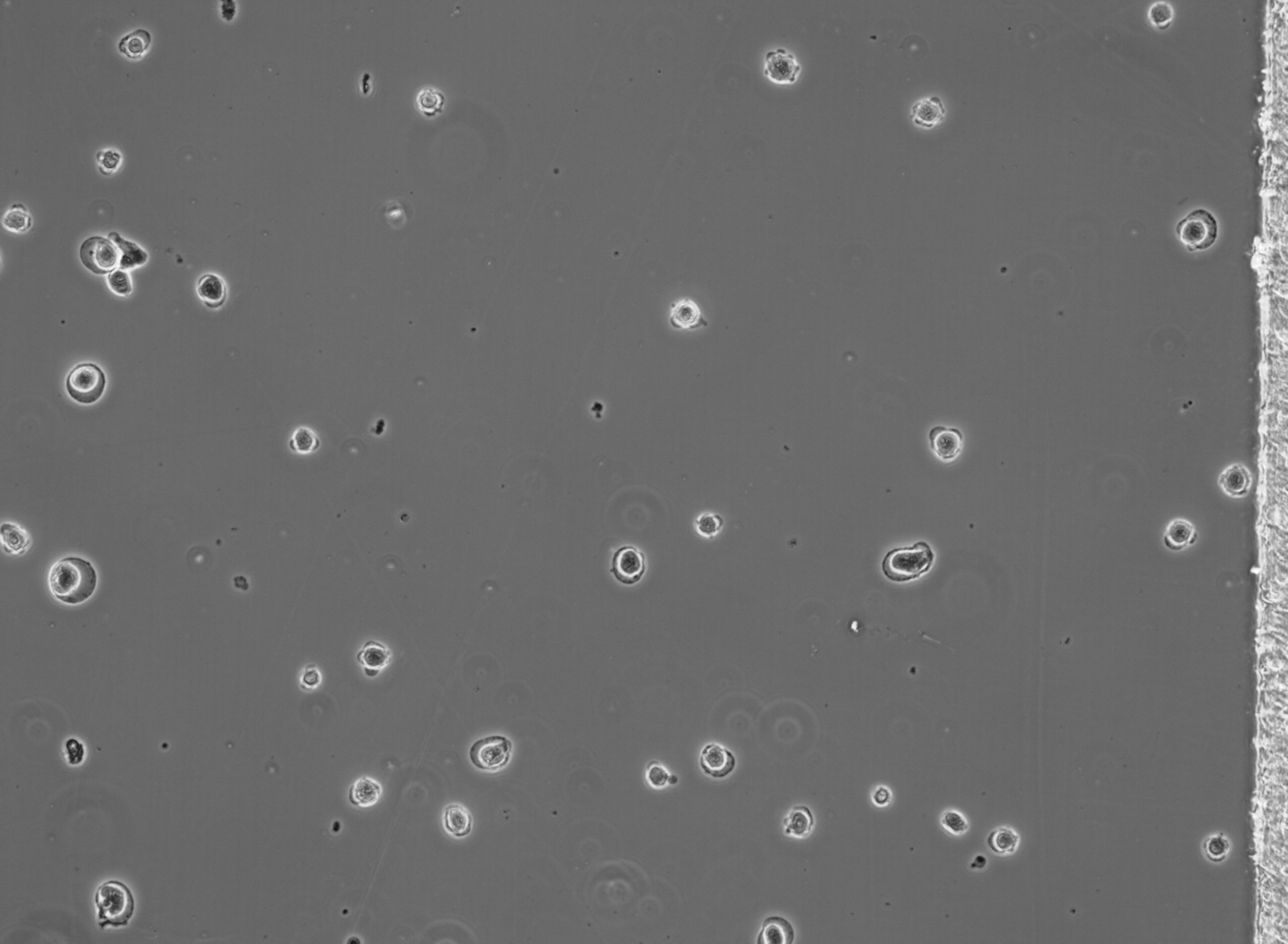

Supplement: S6 File — (ZIP) [file pone.0329484.s006.zip › S6 File - l-CSC 3/l-CSC 3/untitled092.tif]

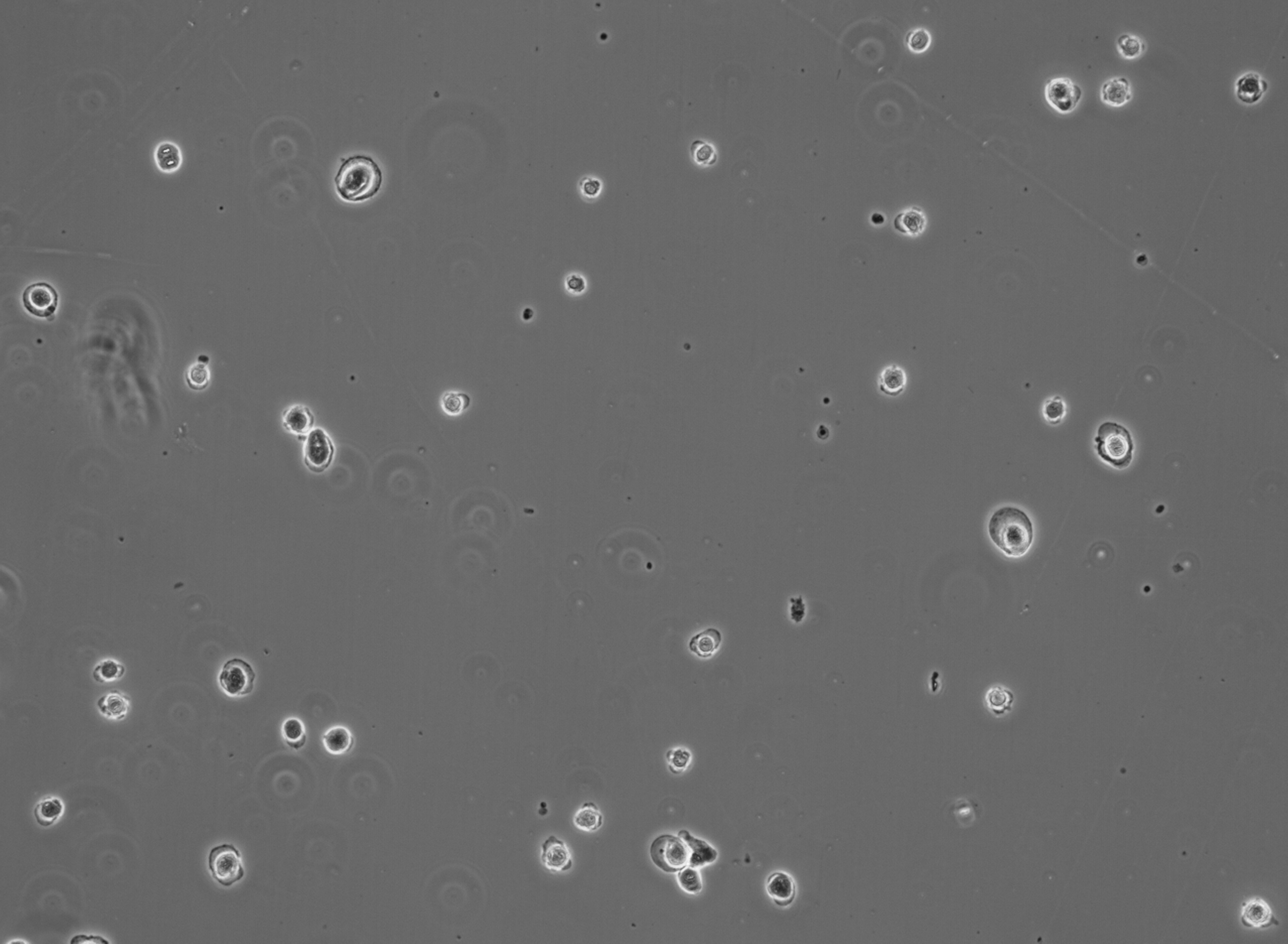

Supplement: S6 File — (ZIP) [file pone.0329484.s006.zip › S6 File - l-CSC 3/l-CSC 3/untitled093.tif]
