# Supplementary figures and images for: Expression of Concern: Ontological Differences in First Compared to Third Trimester Human Fetal Placental Chorionic Stem Cells (part 2 of 2)
Source: PLoS One. 2025 Aug 1;20(8):e0329484. doi: 10.1371/journal.pone.0329484 (PMC12316261; doi:10.1371/journal.pone.0329484)

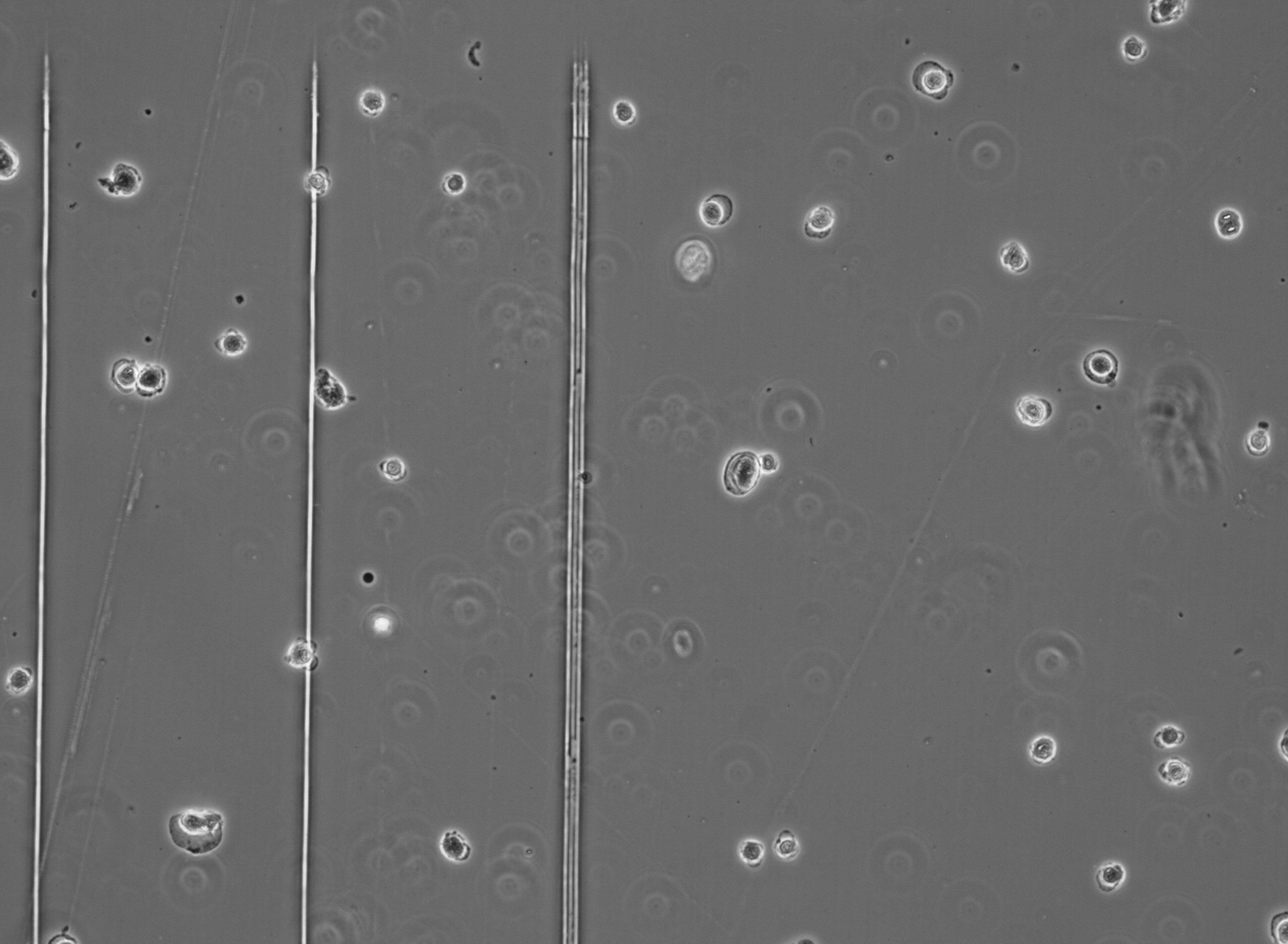

Supplement: S6 File — (ZIP) [file pone.0329484.s006.zip › S6 File - l-CSC 3/l-CSC 3/untitled094.tif]

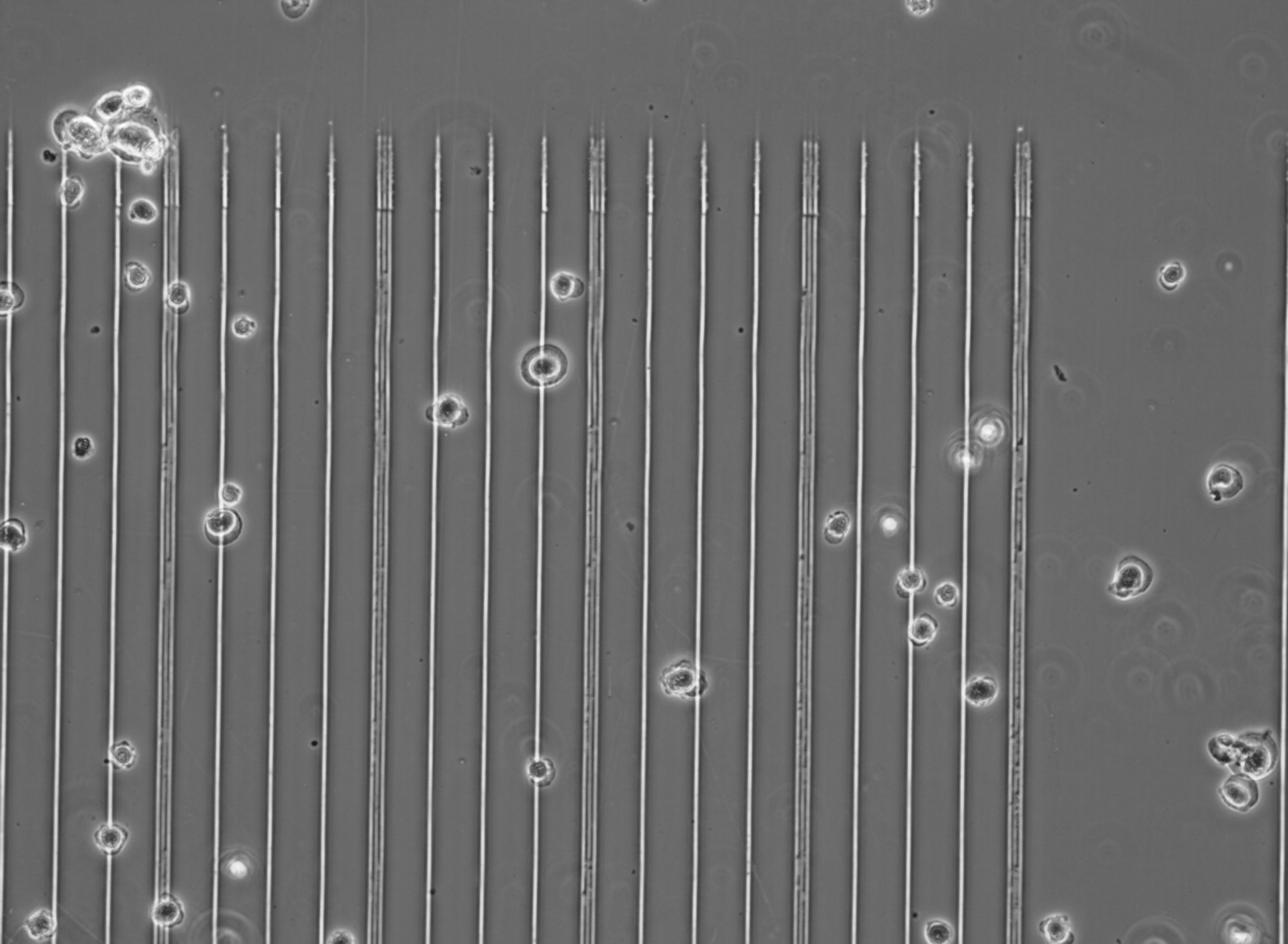

Supplement: S6 File — (ZIP) [file pone.0329484.s006.zip › S6 File - l-CSC 3/l-CSC 3/untitled095.tif]

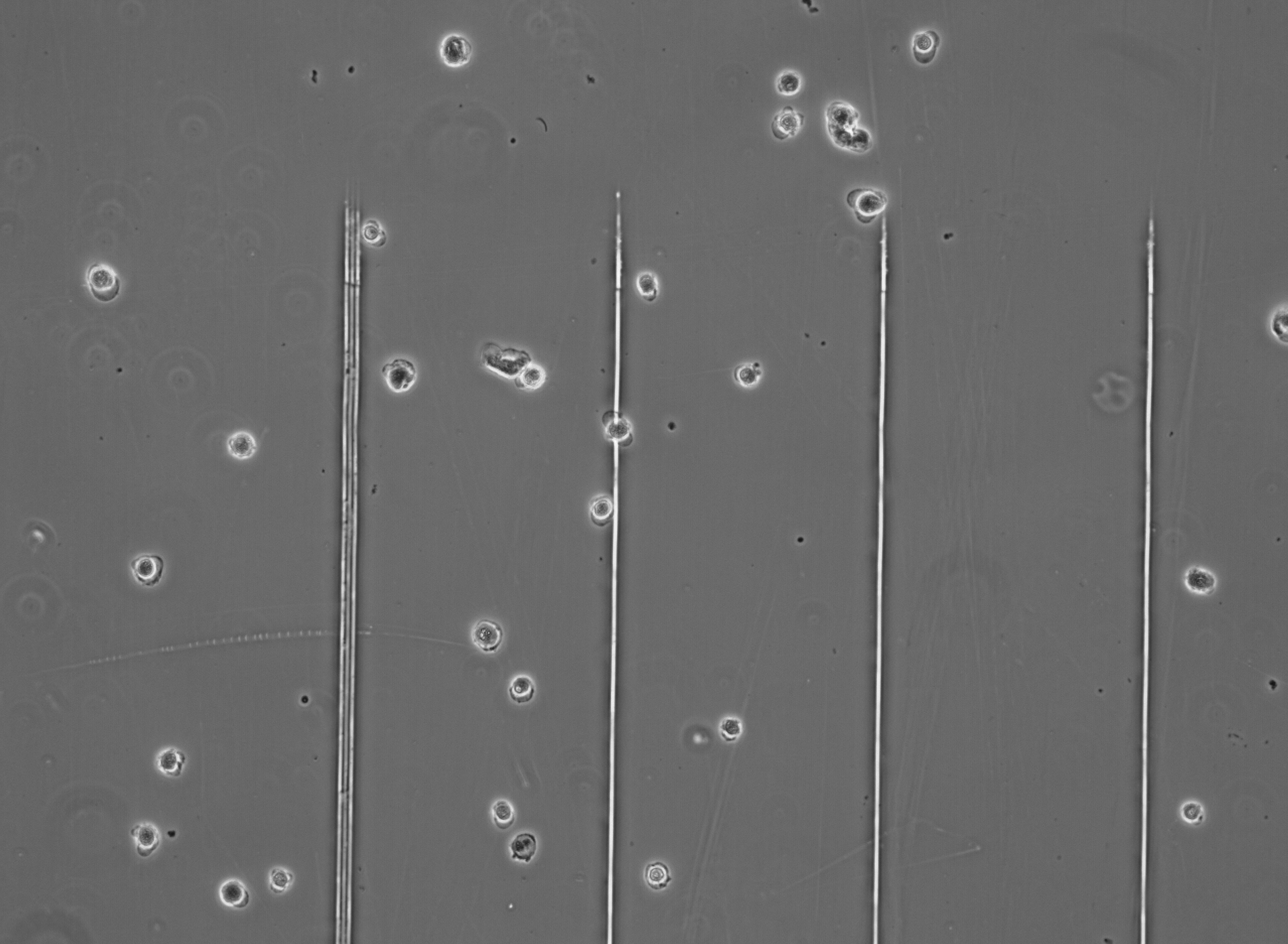

Supplement: S6 File — (ZIP) [file pone.0329484.s006.zip › S6 File - l-CSC 3/l-CSC 3/untitled096.tif]

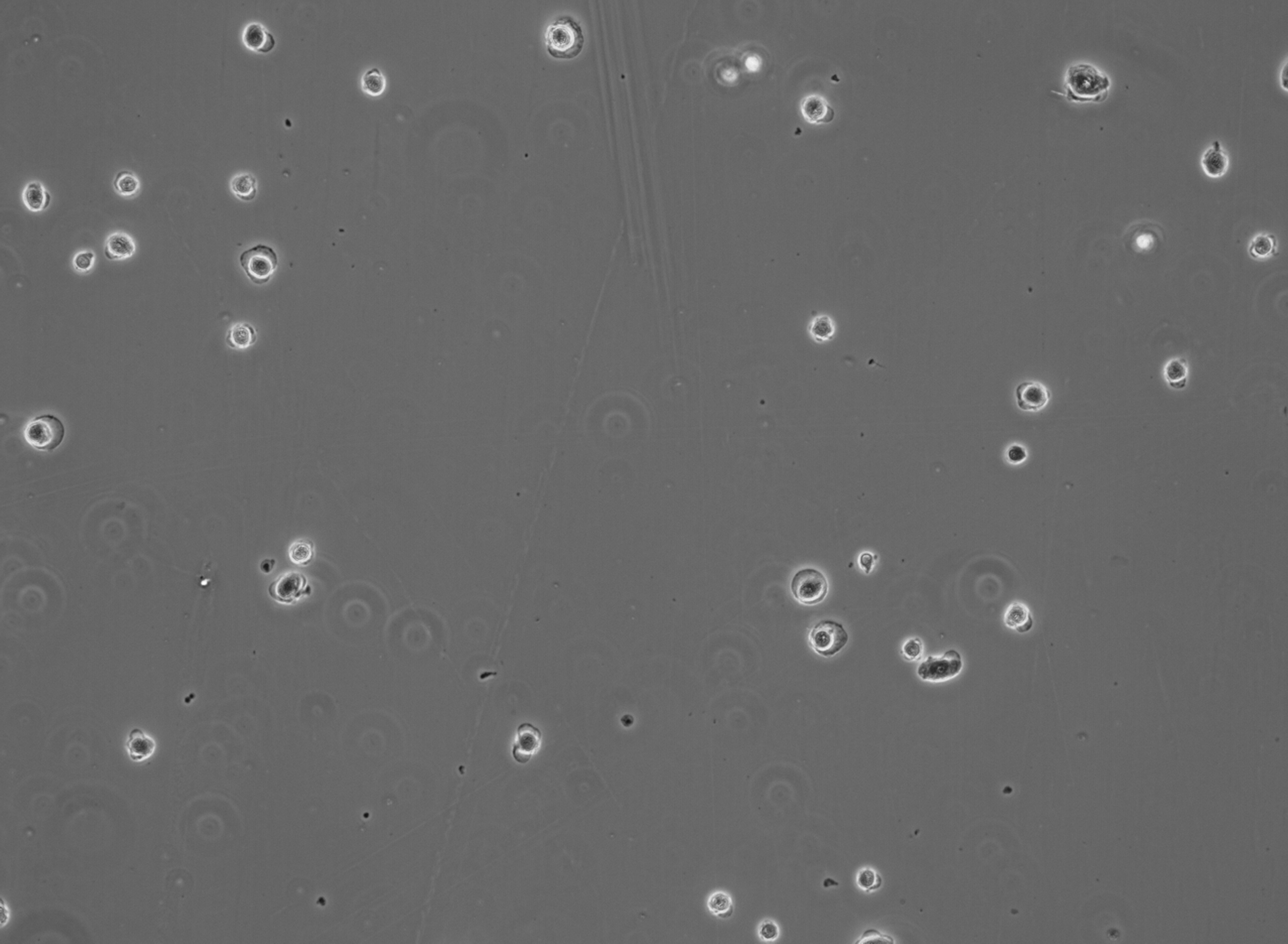

Supplement: S6 File — (ZIP) [file pone.0329484.s006.zip › S6 File - l-CSC 3/l-CSC 3/untitled097.tif]

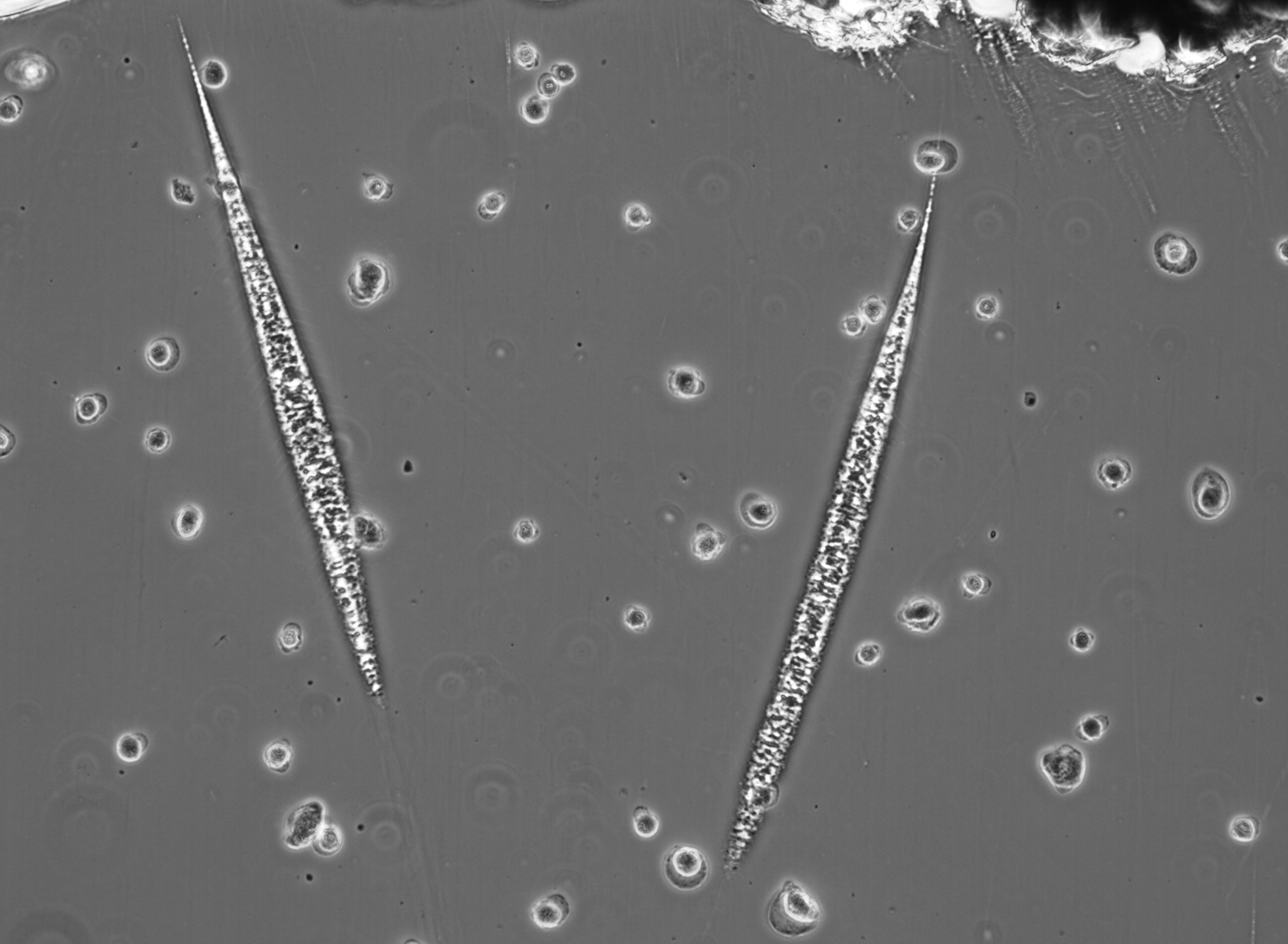

Supplement: S6 File — (ZIP) [file pone.0329484.s006.zip › S6 File - l-CSC 3/l-CSC 3/untitled098.tif]

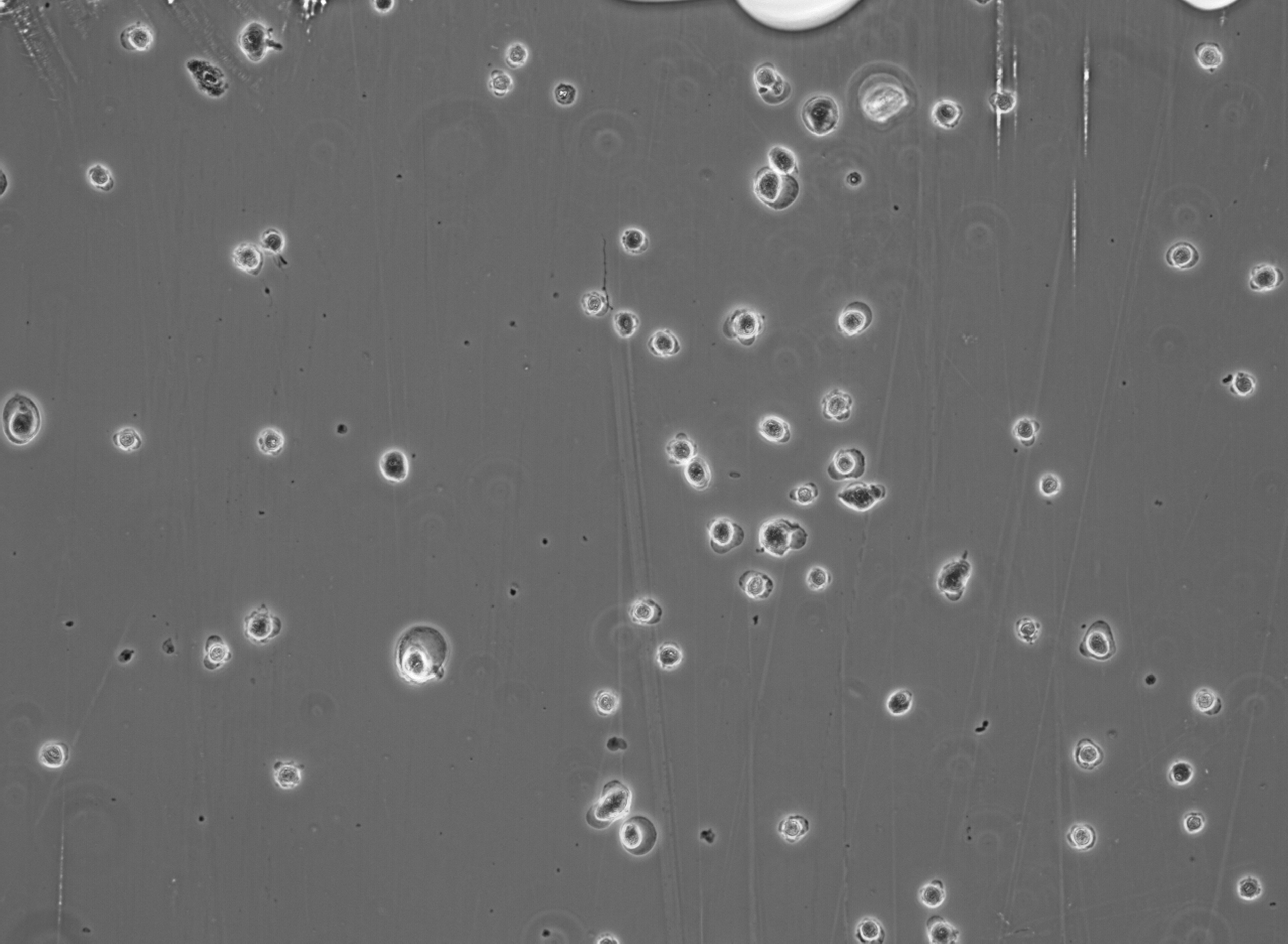

Supplement: S6 File — (ZIP) [file pone.0329484.s006.zip › S6 File - l-CSC 3/l-CSC 3/untitled099.tif]

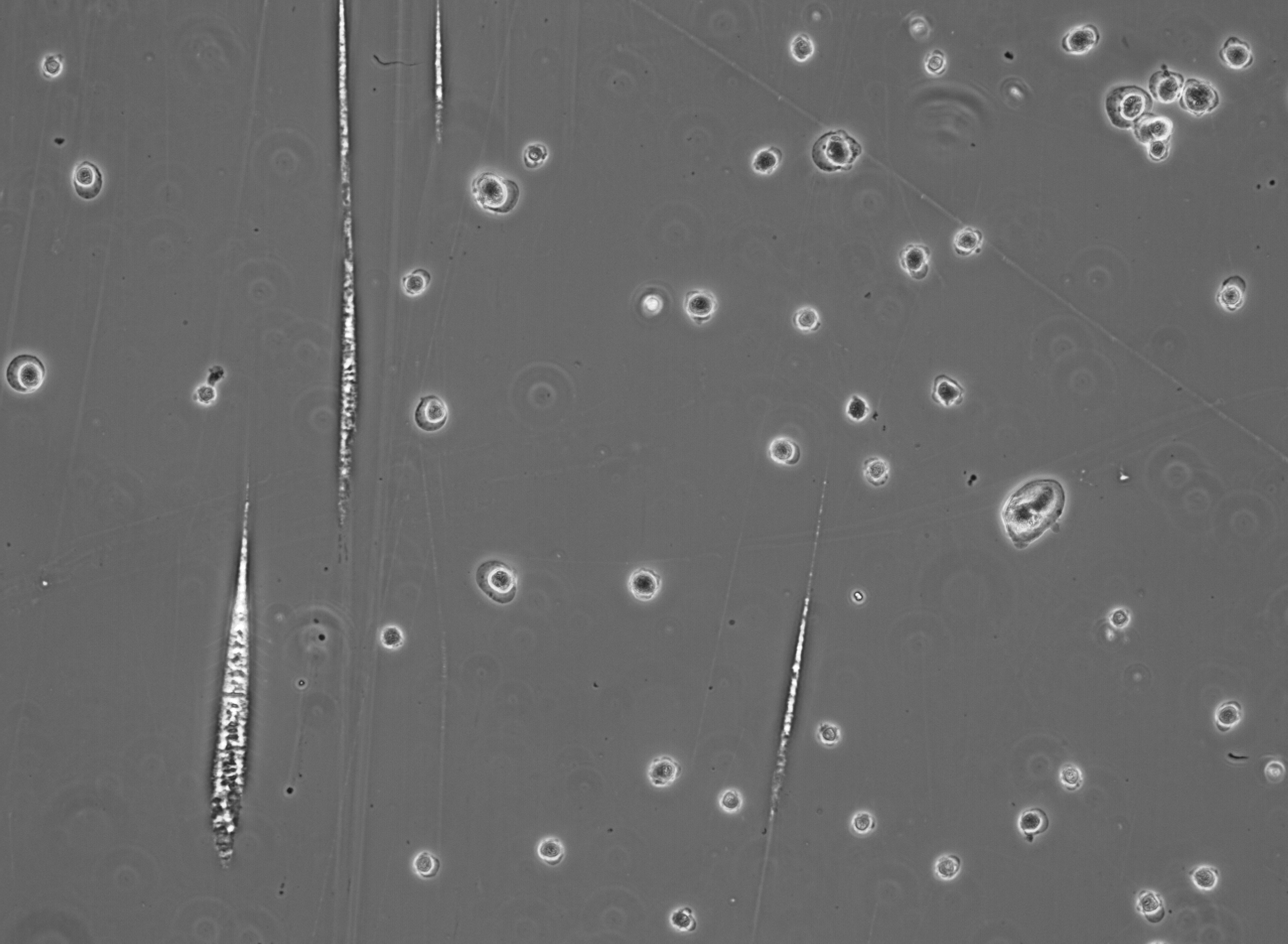

Supplement: S6 File — (ZIP) [file pone.0329484.s006.zip › S6 File - l-CSC 3/l-CSC 3/untitled100.tif]

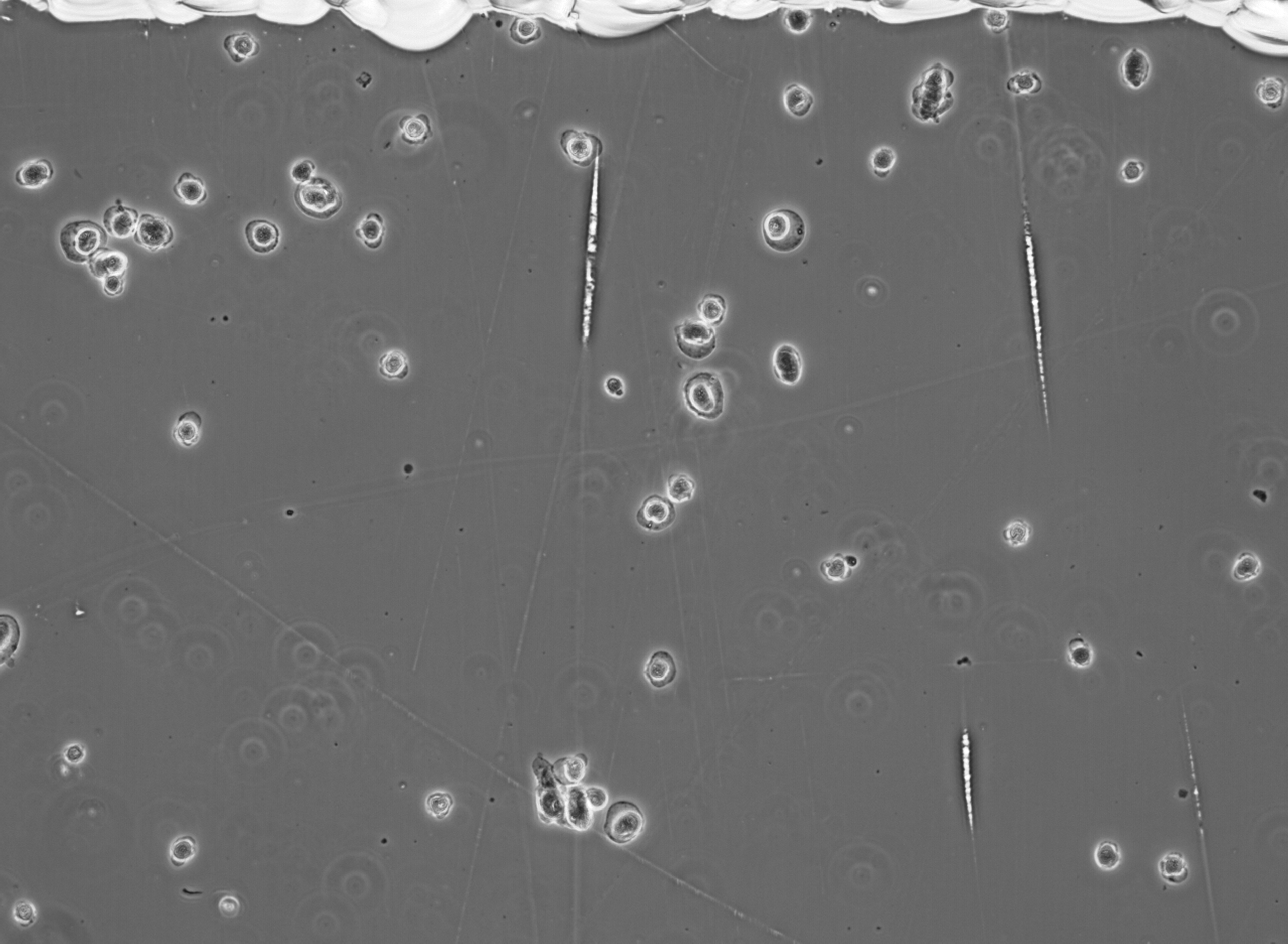

Supplement: S6 File — (ZIP) [file pone.0329484.s006.zip › S6 File - l-CSC 3/l-CSC 3/untitled101.tif]

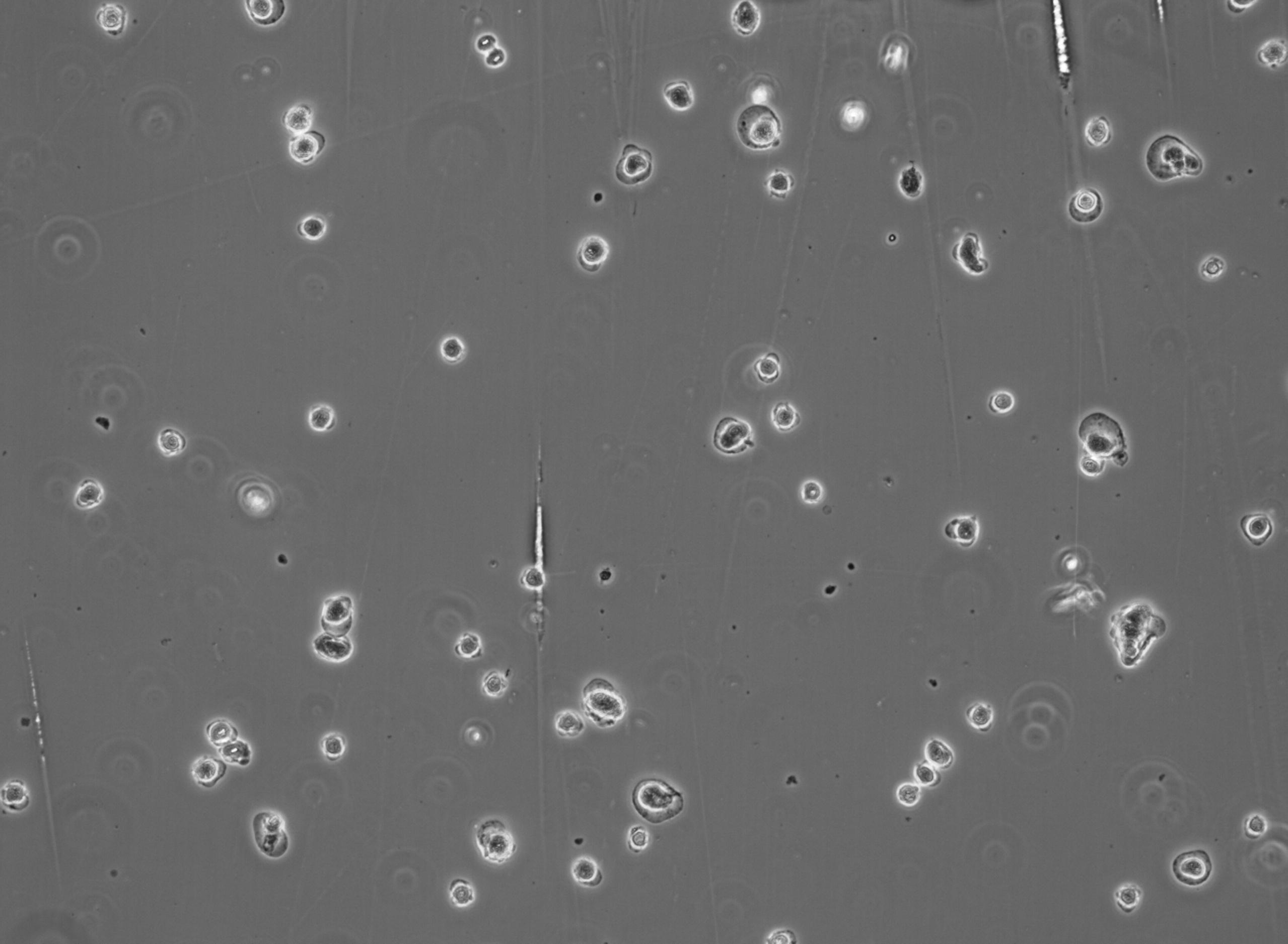

Supplement: S6 File — (ZIP) [file pone.0329484.s006.zip › S6 File - l-CSC 3/l-CSC 3/untitled102.tif]

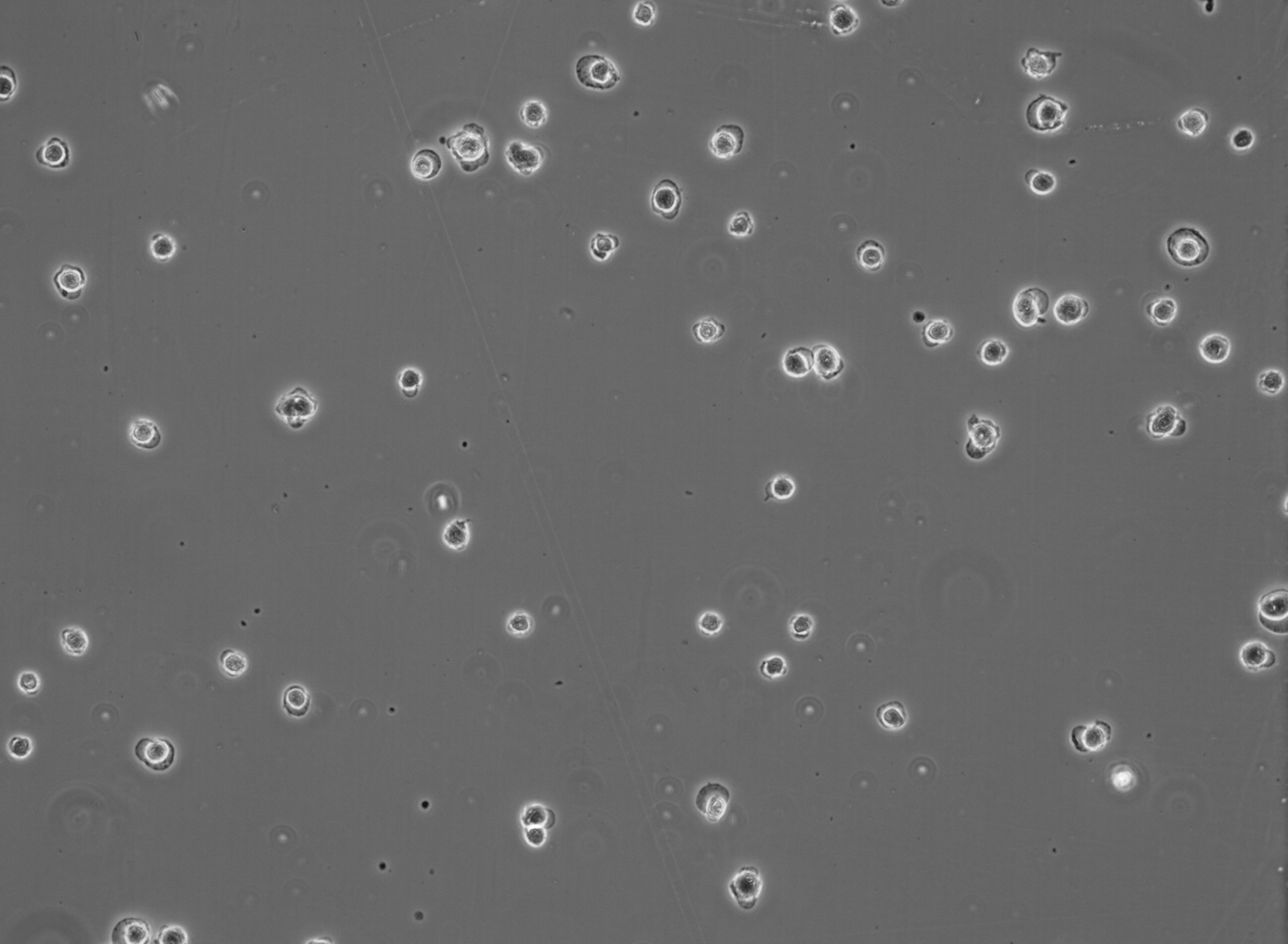

Supplement: S6 File — (ZIP) [file pone.0329484.s006.zip › S6 File - l-CSC 3/l-CSC 3/untitled103.tif]

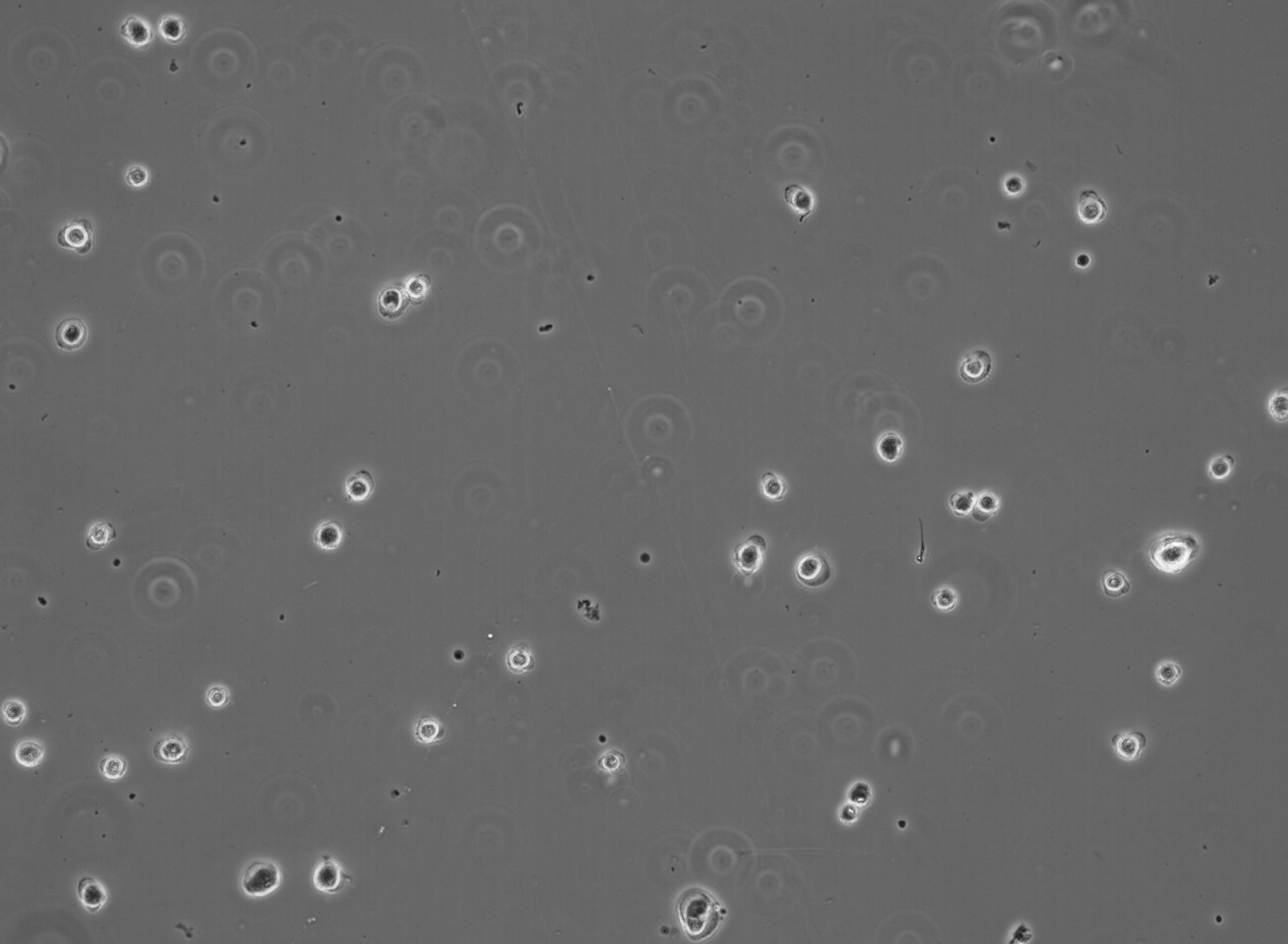

Supplement: S6 File — (ZIP) [file pone.0329484.s006.zip › S6 File - l-CSC 3/l-CSC 3/untitled104.tif]

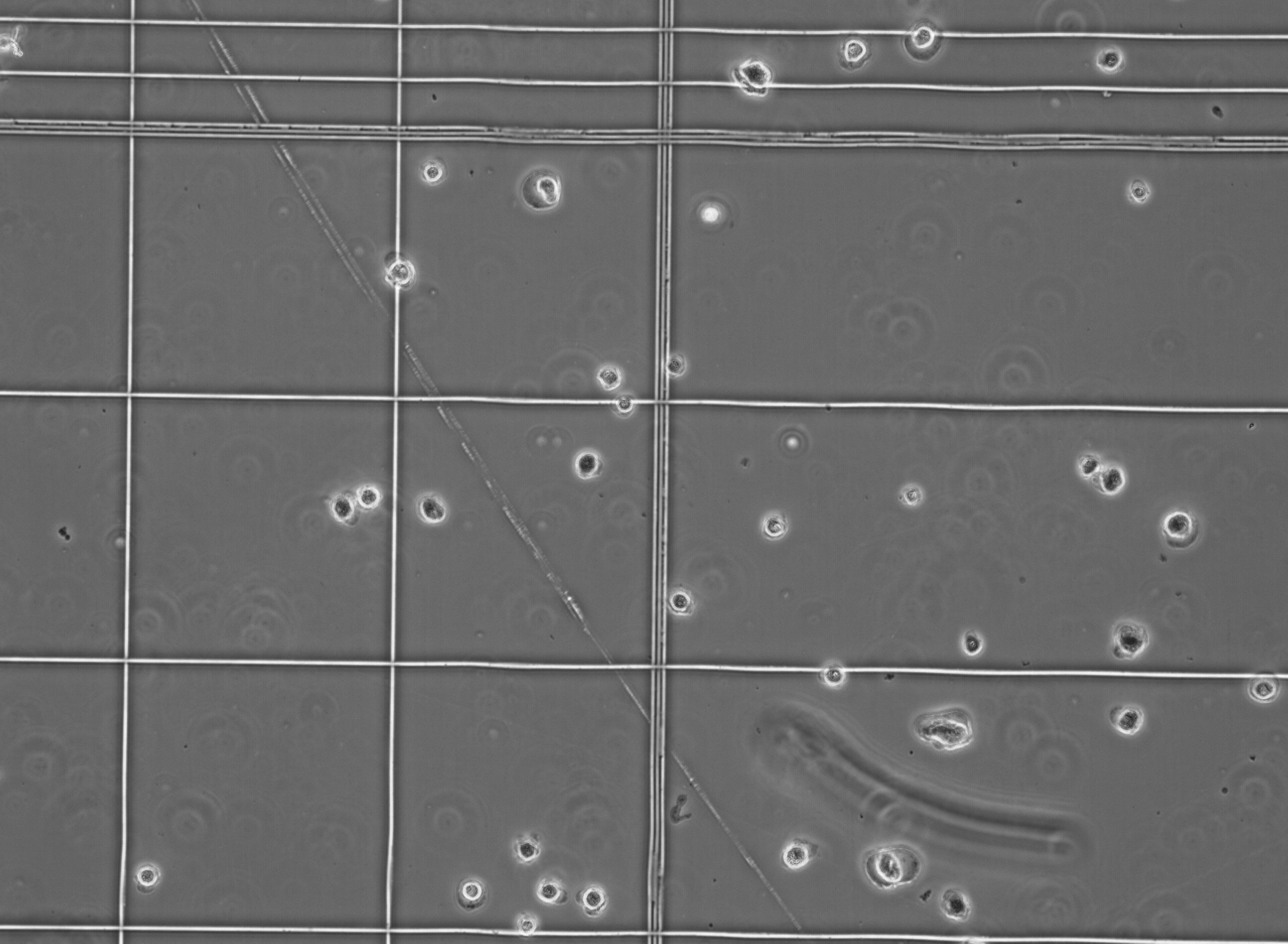

Supplement: S6 File — (ZIP) [file pone.0329484.s006.zip › S6 File - l-CSC 3/l-CSC 3/untitled105.tif]

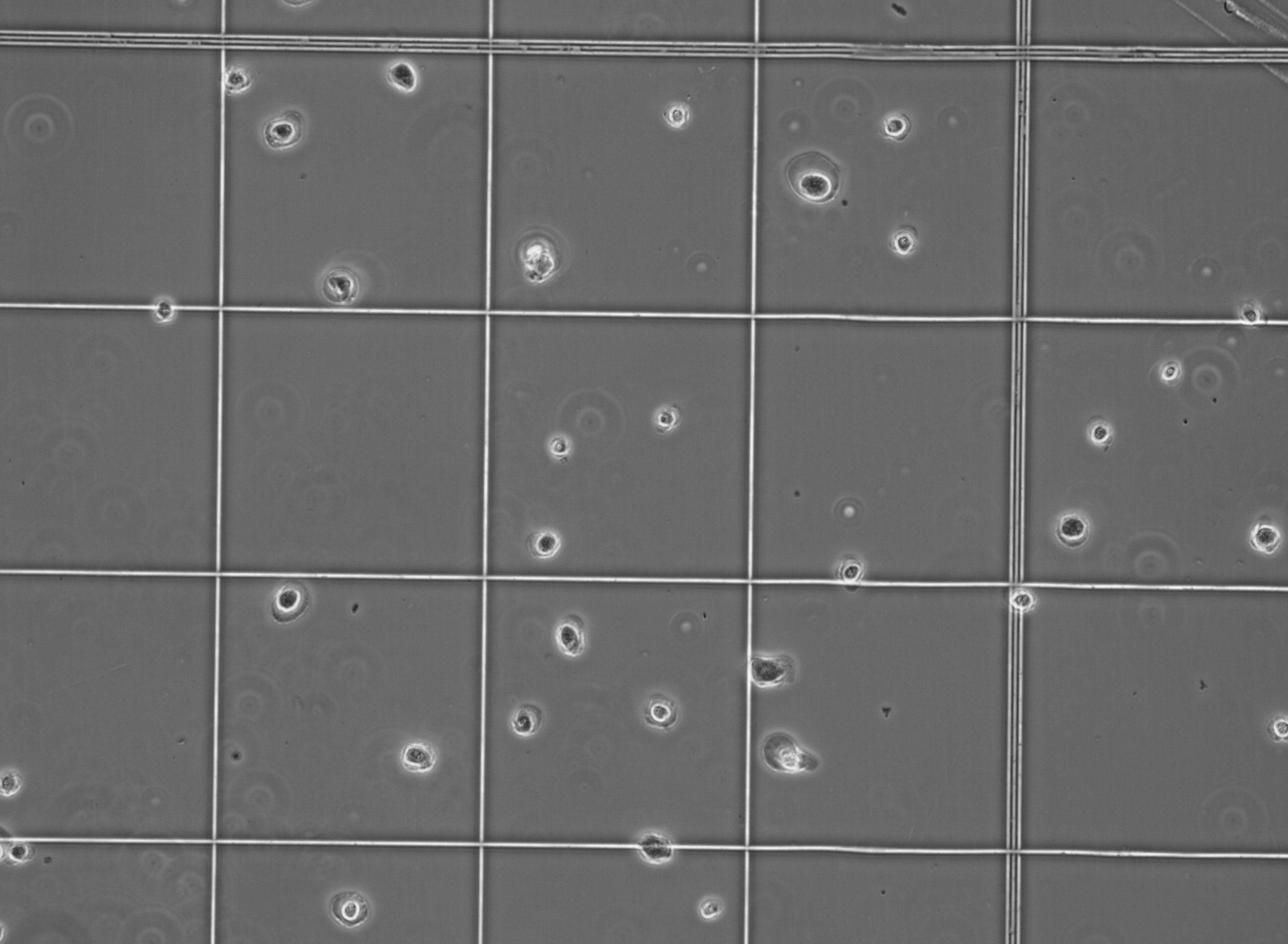

Supplement: S6 File — (ZIP) [file pone.0329484.s006.zip › S6 File - l-CSC 3/l-CSC 3/untitled106.tif]

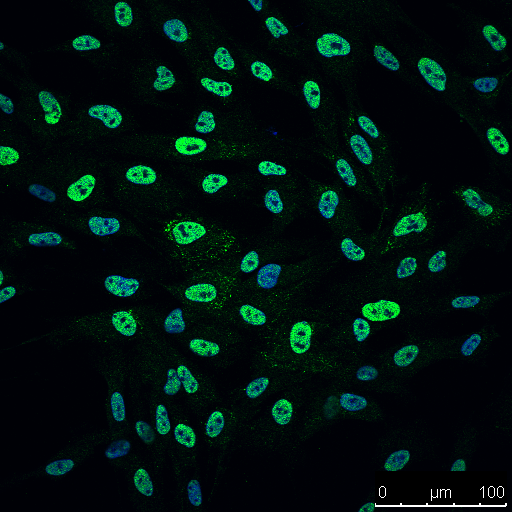

Supplement: S8 File — (ZIP) [file pone.0329484.s008.zip › S8 File/S8 File/Klf4/eCSC/Klf4 eCSC.tif]

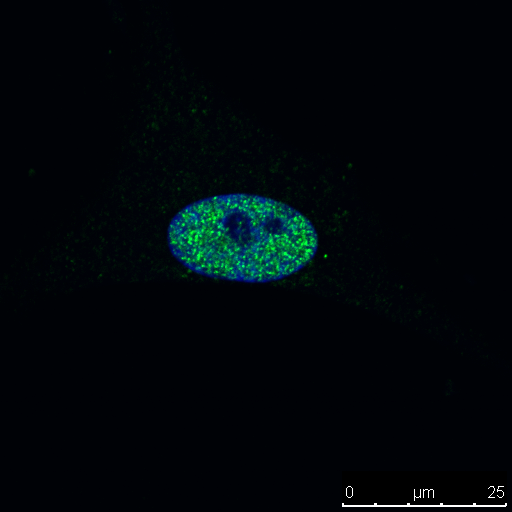

Supplement: S8 File — (ZIP) [file pone.0329484.s008.zip › S8 File/S8 File/Klf4/eCSC/Klfe eCSC sc.tif]

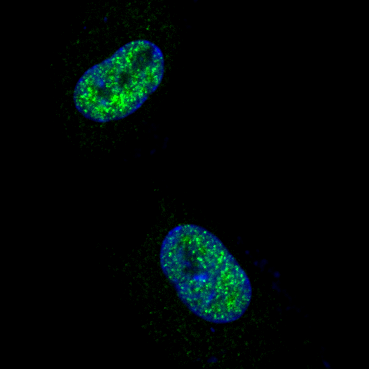

Supplement: S8 File — (ZIP) [file pone.0329484.s008.zip › S8 File/S8 File/Klf4/lCSC/KLF4 lCSC SC.tif]

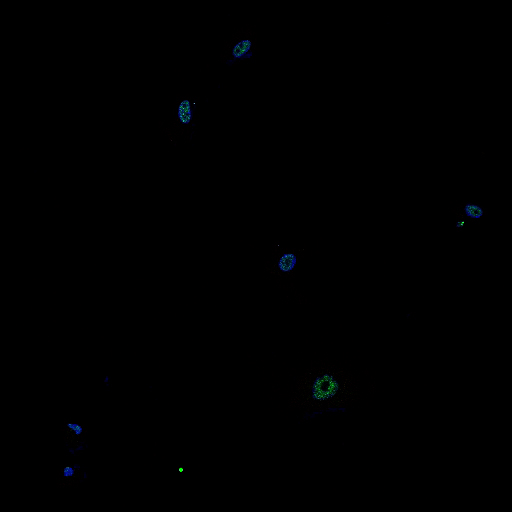

Supplement: S8 File — (ZIP) [file pone.0329484.s008.zip › S8 File/S8 File/Klf4/lCSC/KLF4 lCSC.jpg]

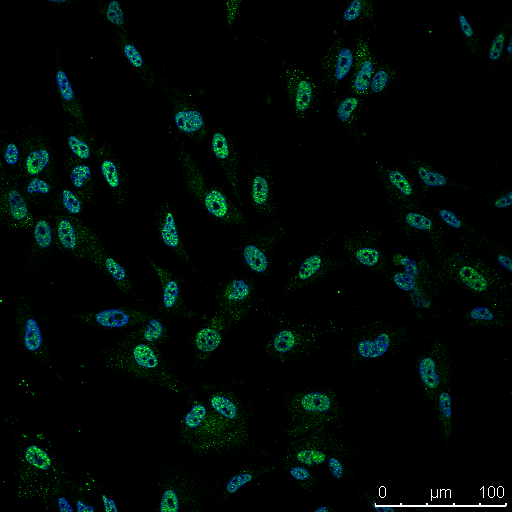

Supplement: S8 File — (ZIP) [file pone.0329484.s008.zip › S8 File/S8 File/Nanog/eCSC/Nanog CSC.tif]

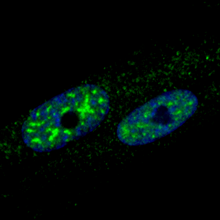

Supplement: S8 File — (ZIP) [file pone.0329484.s008.zip › S8 File/S8 File/Nanog/eCSC/Nanog eCSC SC.tif]

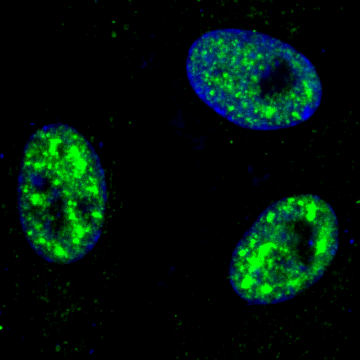

Supplement: S8 File — (ZIP) [file pone.0329484.s008.zip › S8 File/S8 File/Nanog/lCSC/Nanog lCSC SC.tif]

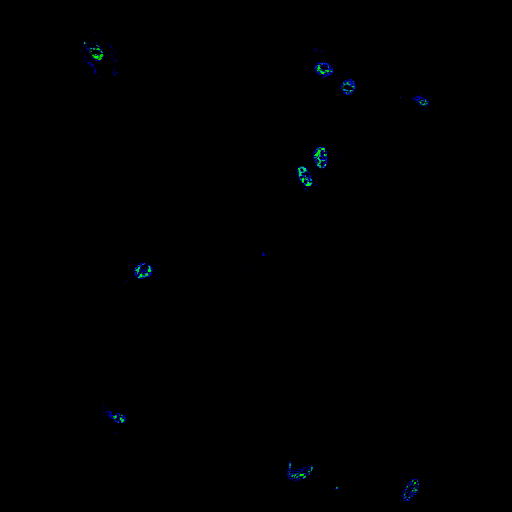

Supplement: S8 File — (ZIP) [file pone.0329484.s008.zip › S8 File/S8 File/Nanog/lCSC/Nanog lCSC.jpg]

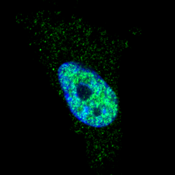

Supplement: S8 File — (ZIP) [file pone.0329484.s008.zip › S8 File/S8 File/Oct4/eCSC/OCT4 eCSC SC.tif]

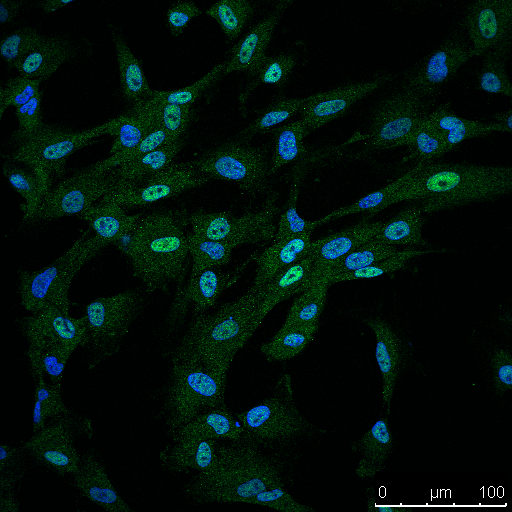

Supplement: S8 File — (ZIP) [file pone.0329484.s008.zip › S8 File/S8 File/Oct4/eCSC/OCT4 eCSC.tif]

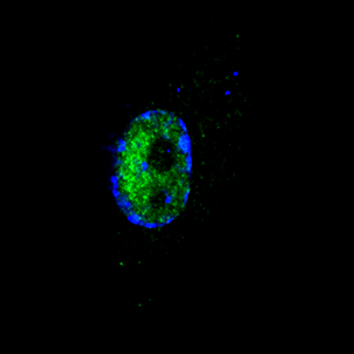

Supplement: S8 File — (ZIP) [file pone.0329484.s008.zip › S8 File/S8 File/Oct4/lCSC/OCT4 lCSC SC.jpg]

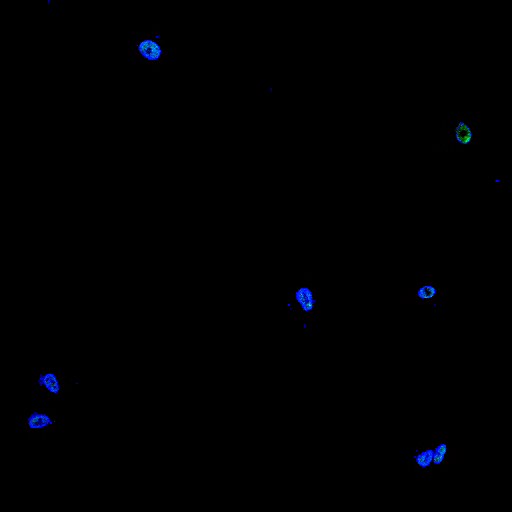

Supplement: S8 File — (ZIP) [file pone.0329484.s008.zip › S8 File/S8 File/Oct4/lCSC/OCT4 lCSC.jpg]

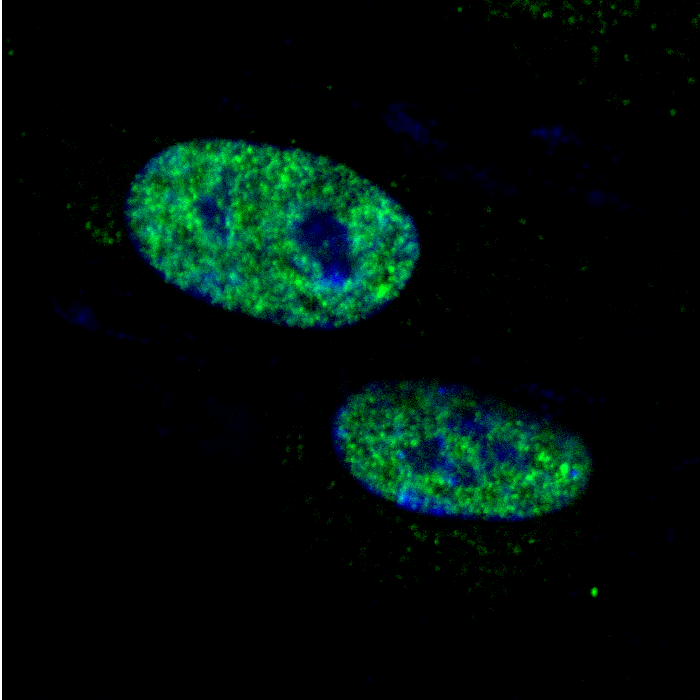

Supplement: S8 File — (ZIP) [file pone.0329484.s008.zip › S8 File/S8 File/Rex1/eCSC/Rex1 eCSC SC.tif]

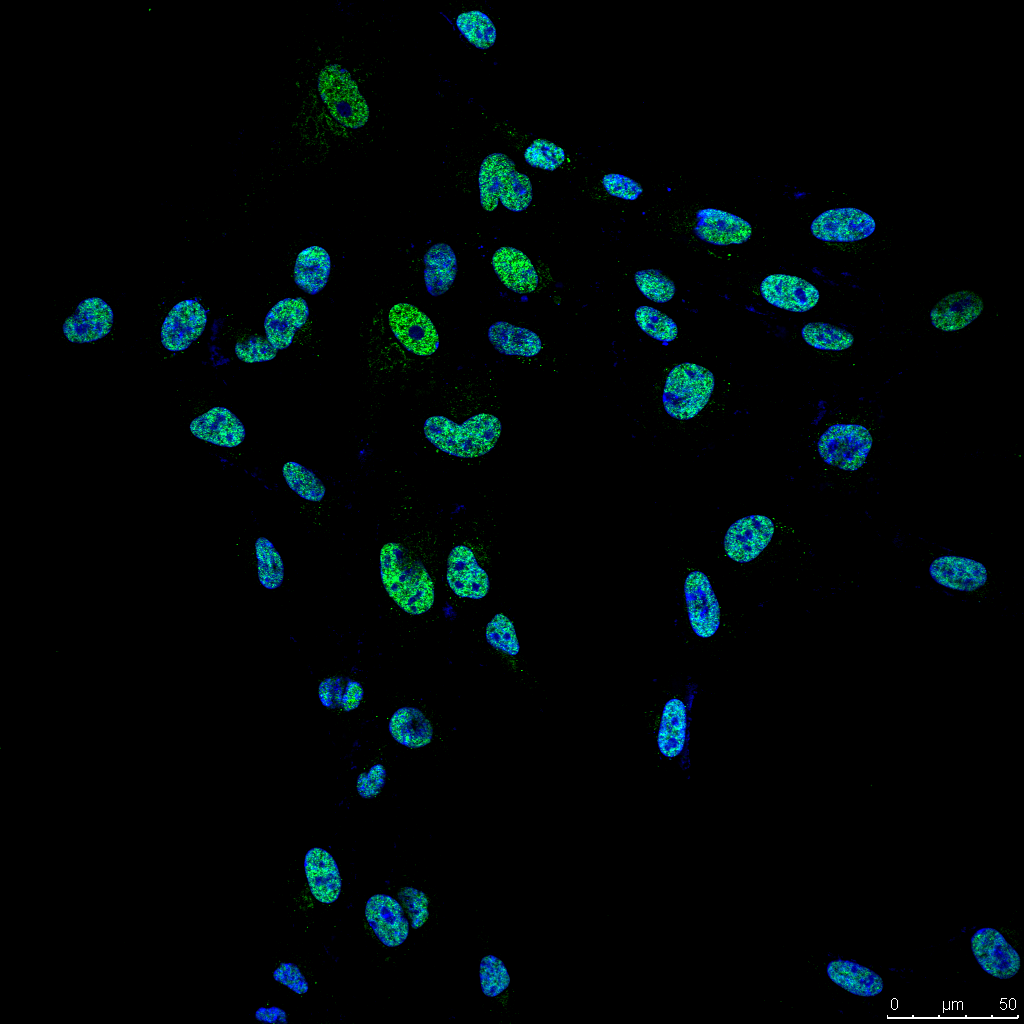

Supplement: S8 File — (ZIP) [file pone.0329484.s008.zip › S8 File/S8 File/Rex1/eCSC/Rex1 eCSC.tif]

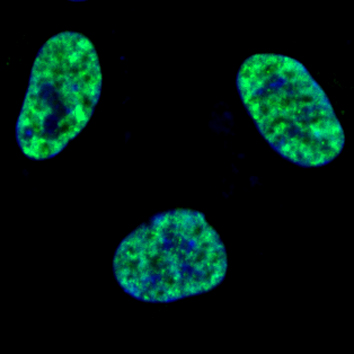

Supplement: S8 File — (ZIP) [file pone.0329484.s008.zip › S8 File/S8 File/Rex1/lCSC/Rex1 lCSC SC.jpg]

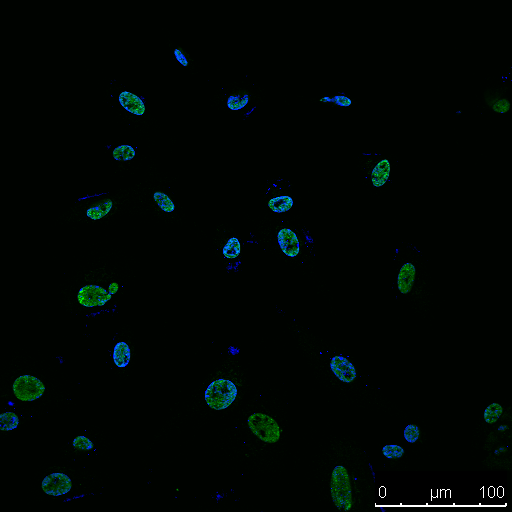

Supplement: S8 File — (ZIP) [file pone.0329484.s008.zip › S8 File/S8 File/Rex1/lCSC/Rex1 lCSC.tif]

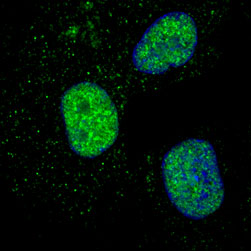

Supplement: S8 File — (ZIP) [file pone.0329484.s008.zip › S8 File/S8 File/Sox2/eCSC/Sox2 eCSC SC.jpg]

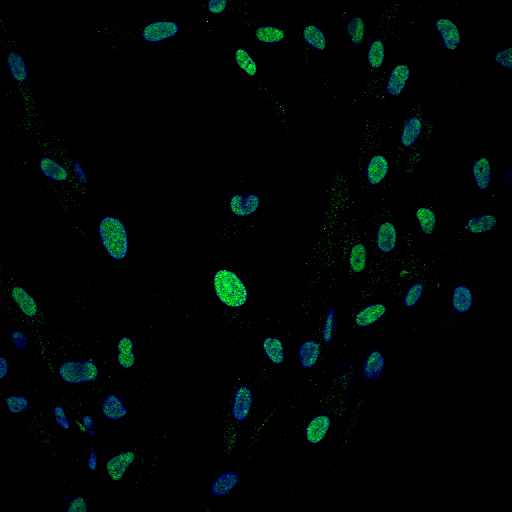

Supplement: S8 File — (ZIP) [file pone.0329484.s008.zip › S8 File/S8 File/Sox2/eCSC/Sox2 eCSC.jpg]

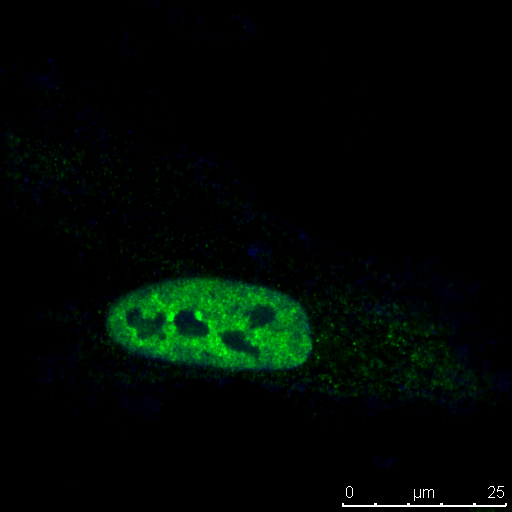

Supplement: S8 File — (ZIP) [file pone.0329484.s008.zip › S8 File/S8 File/Sox2/lCSC/Sox2 lCSC SC.tif]

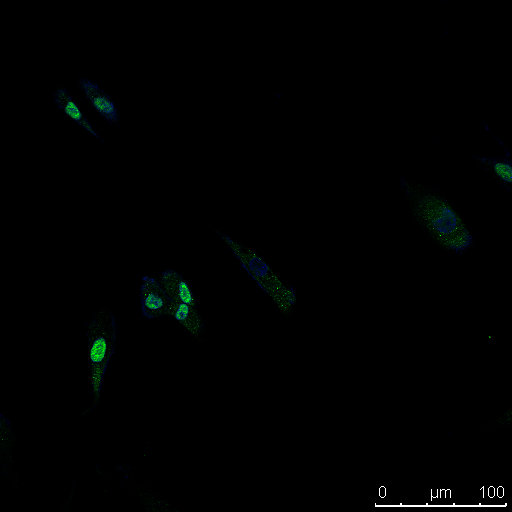

Supplement: S8 File — (ZIP) [file pone.0329484.s008.zip › S8 File/S8 File/Sox2/lCSC/Sox2 lCSC.tif]

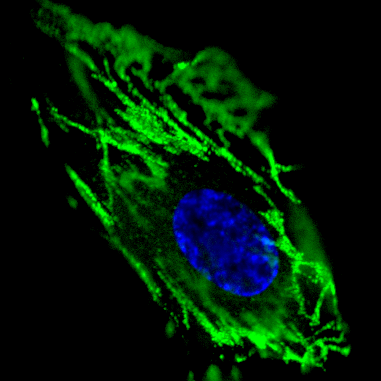

Supplement: S8 File — (ZIP) [file pone.0329484.s008.zip › S8 File/S8 File/SSEA-3/eCSC/SSEA3 eCSC SC.tif]

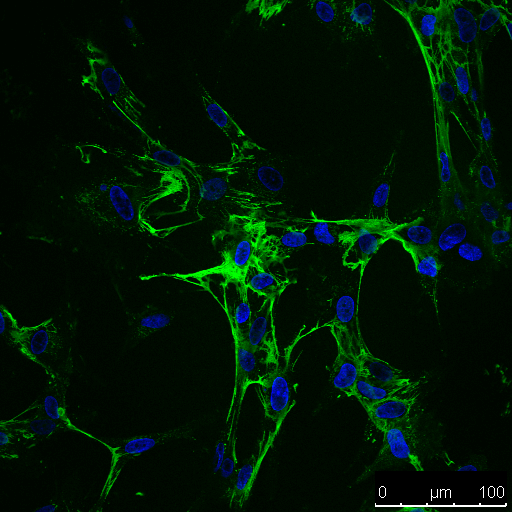

Supplement: S8 File — (ZIP) [file pone.0329484.s008.zip › S8 File/S8 File/SSEA-3/eCSC/SSEA3 eCSC.tif]

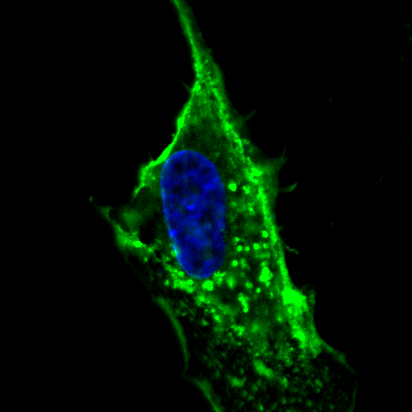

Supplement: S8 File — (ZIP) [file pone.0329484.s008.zip › S8 File/S8 File/SSEA-3/lCSC/SSEA3 lCSC SC.tif]

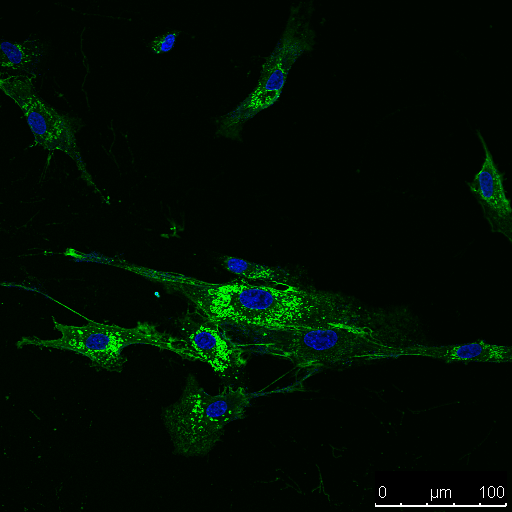

Supplement: S8 File — (ZIP) [file pone.0329484.s008.zip › S8 File/S8 File/SSEA-3/lCSC/SSEA3 lCSC.tif]

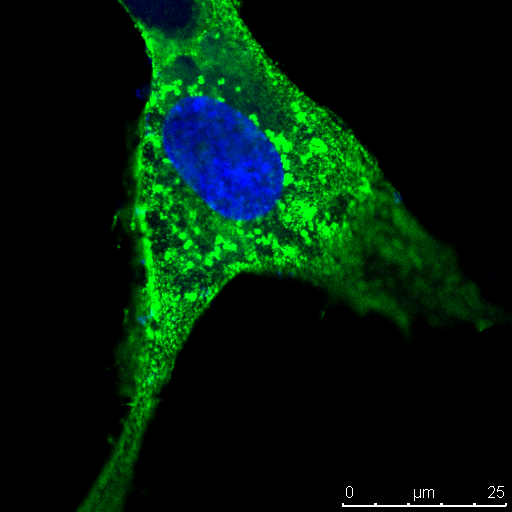

Supplement: S8 File — (ZIP) [file pone.0329484.s008.zip › S8 File/S8 File/SSEA-4/eCSC/SSEA4 eCSC SC.tif]

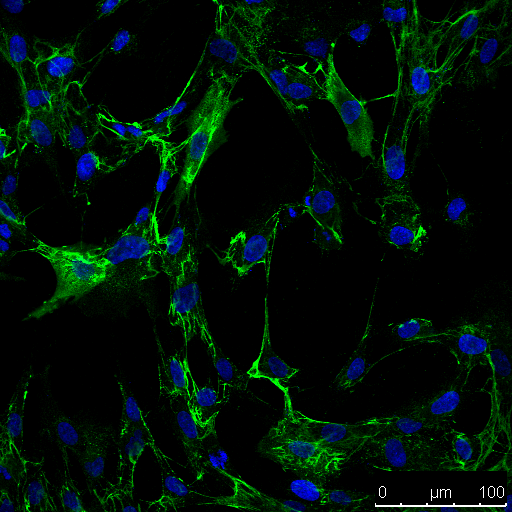

Supplement: S8 File — (ZIP) [file pone.0329484.s008.zip › S8 File/S8 File/SSEA-4/eCSC/SSEA4 eCSC.tif]

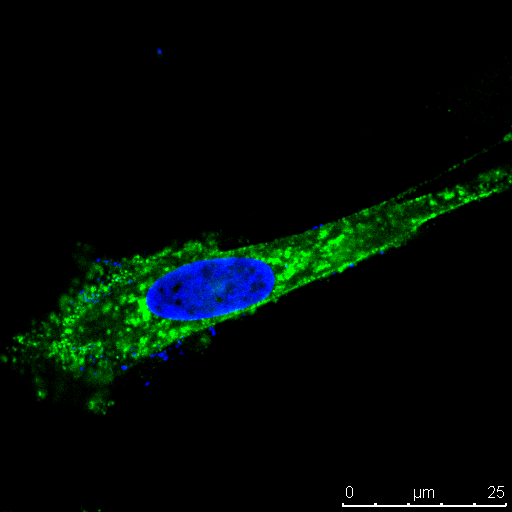

Supplement: S8 File — (ZIP) [file pone.0329484.s008.zip › S8 File/S8 File/SSEA-4/lCSC/Copy of Series172_z0.tif]

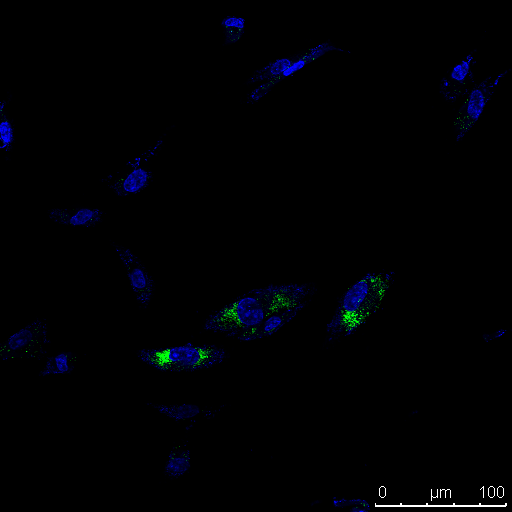

Supplement: S8 File — (ZIP) [file pone.0329484.s008.zip › S8 File/S8 File/SSEA-4/lCSC/SSEA4 low res overlay (2).tif]

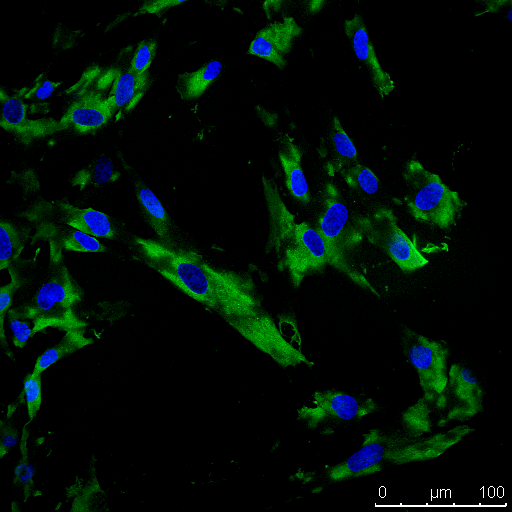

Supplement: S8 File — (ZIP) [file pone.0329484.s008.zip › S8 File/S8 File/Tra-1-60/eCSC/TRA-1-60 eCSC.tif]

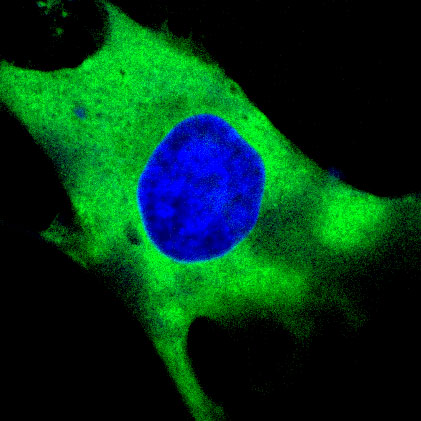

Supplement: S8 File — (ZIP) [file pone.0329484.s008.zip › S8 File/S8 File/Tra-1-60/eCSC/TRA1-1-60 eCSC SC.jpg]

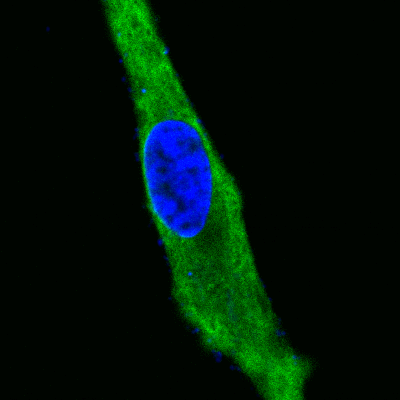

Supplement: S8 File — (ZIP) [file pone.0329484.s008.zip › S8 File/S8 File/Tra-1-60/lCSC/TRA-1-60 lCSC SC.tif]

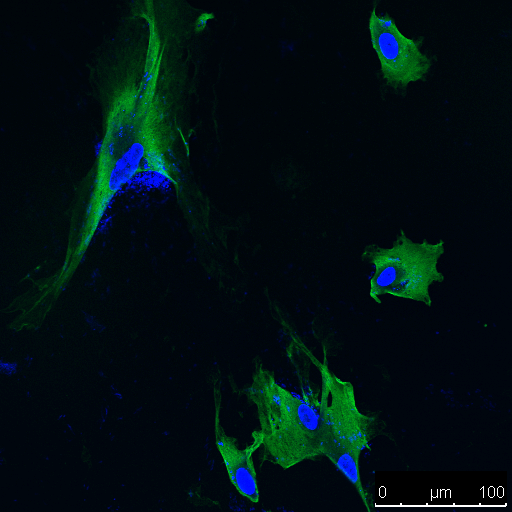

Supplement: S8 File — (ZIP) [file pone.0329484.s008.zip › S8 File/S8 File/Tra-1-60/lCSC/TRA-1-60 lCSC.tif]

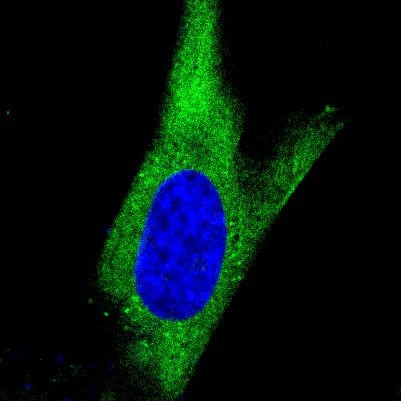

Supplement: S8 File — (ZIP) [file pone.0329484.s008.zip › S8 File/S8 File/Tra-1-81/eCSC/TRA-1-81 eCSC SC.tif]

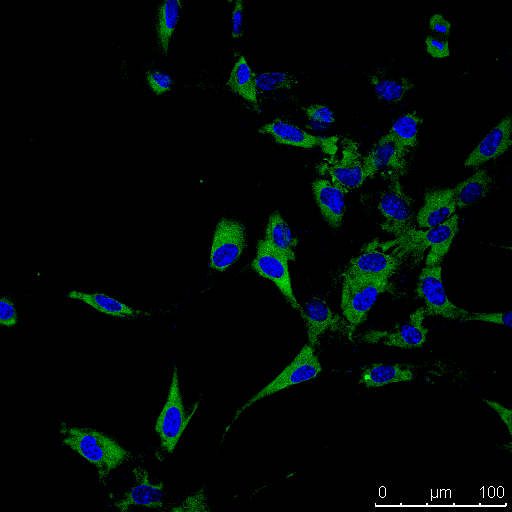

Supplement: S8 File — (ZIP) [file pone.0329484.s008.zip › S8 File/S8 File/Tra-1-81/eCSC/TRA-1-81 eCSC.tif]

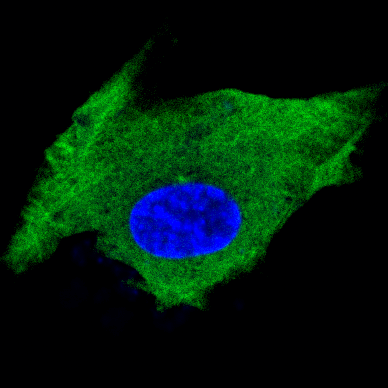

Supplement: S8 File — (ZIP) [file pone.0329484.s008.zip › S8 File/S8 File/Tra-1-81/lCSC/TRA-1-81 lCSC SC.tif]

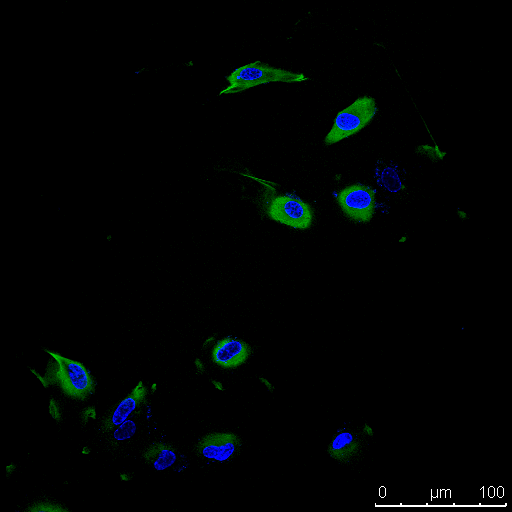

Supplement: S8 File — (ZIP) [file pone.0329484.s008.zip › S8 File/S8 File/Tra-1-81/lCSC/TRA-1-81 lCSC.tif]
